# Visible Light‐Mediated Preparation of a Key Intermediate Employed in the Synthesis of Zolpidem and Several Analogs

**DOI:** 10.1002/asia.202500455

**Published:** 2025-05-13

**Authors:** Ronei M. S. Souza, Tales A. C. Goulart, Roberto do C. Pinheiro, Felipe F. do C. Sonaglio, Deborah de A. Simoni, Rodrigo A. Cormanich, Igor D. Jurberg

**Affiliations:** ^1^ Department of Organic Chemistry Institute of Chemistry State University of Campinas Rua Monteiro Lobato 270, 13083–862 Campinas SP Brazil; ^2^ Institutional X‐Ray Laboratory, Institute of Chemistry State University of Campinas Rua Monteiro Lobato 270, 13083–862 Campinas SP Brazil

**Keywords:** Diazo compound, Imidazo[1,2‐a]pyrimidine, Imidazo[1,2‐a]pyridine, Visible light, Zolpidem

## Abstract

This work describes a protocol being only promoted by visible light for the C3‐alkylation of imidazo[1,2‐a]pyrimidines and imidazo[1,2‐a]pyridines with aryldiazoacetates. Several known and new analogs of a key intermediate employed in previous syntheses of zolpidem have been accessed in good yields. The preparation of this key intermediate was also achieved using this method, but an adjustment of the nature of the alkylating agent was required.

## Graphical abstract

A new visible light‐induced method is reported for the C3‐alkylation of imidazo[1,2‐a]pyrimidines and ‐pyridines with aryldiazoacetates. This protocol can be performed in open air and serves as a convenient entry to several known and new molecules. A formal synthesis of zolpidem is achieved by changing the alkylating agent to an appropriate hypervalent iodine compound followed by a Krapcho decarboxylation step.

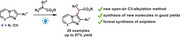

## 1 Introduction

**Figure 1:**
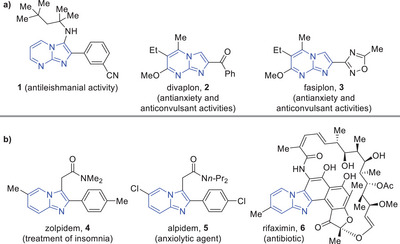
a) Examples of bioactive imidazo[1,2‐a]pyrimidines. b) Examples of bioactive imidazo[1,2‐a]pyridines.

Both imidazo[1,2*‐a*]pyrimidines^[^ ^1^, ^2^ ^]^ and imidazo[1,2*‐a*]pyridines^[^ ^3^, ^4^ ^]^ are highly relevant cores in medicinal chemistry. The imidazo[1,2*‐a*]pyrimidine motif can be found in molecules displaying antiviral,^[^ ^5^ ^]^ antibacterial,^[^ ^6^, ^7^ ^]^ antifungal,^[^ ^6^, ^8^, ^9^ ^]^ antileishmanial (**1**),^[^ ^10^ ^]^ and anti‐inflammatory^[^ ^11^ ^]^ activities, among others.^[^ ^1^, ^2^ ^]^ Furthermore, some of these *aza*‐arene structures have also shown antianxiety and anticonvulsant properties, which led to the development of drug candidates, such as divaplon **2** (RU 32698)^[^ ^12^, ^13^ ^]^ and fasiplon **3** (RU 33203)^[^ ^14^, ^15^ ^]^ (Figure 1a). Similarly, the imidazo[1,2*‐a*]pyridine core has been also associated with several biological activities, such as antibacterial,^[^ ^16^ ^]^ anthelmintic and antifungal,^[^ ^17^ ^]^ antiviral,^[^ ^5^, ^18^, ^19^ ^]^ antiulcer,^[^ ^20^ ^]^ and anti‐cancer.^[^ ^21^ ^]^ Numerous examples of drugs containing this *aza‐*arene core have been developed, such as zolpidem **4** (treatment of insomnia),^[^ ^22^ ^]^ alpidem **5** (anxiolytic agent),^[^ ^23^ ^]^ and rifaximin **6** (antibiotic),^[^ ^24^ ^]^ among others.^[^ ^3^, ^4^ ^]^ (Figure 1b).

Various synthetic routes were designed to afford zolpidem and/ or other similar compounds.^[^ ^25^, ^26^, ^27^, ^28^, ^29^, ^30^, ^31^, ^32^, ^33^, ^34^, ^35^ ^]^ Among these routes, several strategies take advantage of a C3‐alkylation step as a key synthetic event.^[^ ^29^, ^30^, ^31^, ^32^, ^33^, ^34^, ^35^ ^]^ For instance, Wang, Li and co‐workers^[^ ^29^ ^]^ and Miranda and co‐workers^[^ ^30^ ^]^ have both reported thermal reactions employing xanthate derivatives as alkylating agents in the presence of dilauroyl peroxide. Rao, Xu and co‐workers described a thermal Fe‐catalyzed dehydrogenative coupling employing acetonitrile as the alkylating agent in the presence of dicumyl peroxide.^[^ ^31^ ^]^

Liu, Sun and co‐workers disclosed a photoredox‐catalyzed protocol using *fac*‐Ir(ppy)_3_ and employing bromoacetonitrile as the alkylating agent.^[^ ^32^ ^]^ Yu, Tan, Deng and co‐workers revealed the use of ethyl diazoacetate as the alkylating agent in the presence of Ru(bpy)_3_Cl_2_ as the photoredox catalyst.^[^ ^33^ ^]^ On the other hand, Wang and co‐workers have also taken advantage of the use of the same alkylating agent, but in a thermal reaction catalyzed by Rh_2_(oct)_4_.^[^ ^34^ ^]^ Finally, Chaubey and co‐workers have explored a photochemical protocol promoted by rose bengal as the photocatalyst and B_2_(pin)_2_ as an additive, using dialkyl 2‐bromomalonate as the alkylating agent.^[^ ^35^ ^]^

Overall, it seems reasonable to assume that alkylation strategies can play an important role in the functionalization of a number of *aza*‐arenes; thus possibly allowing the development of drug discovery programs based on them. In this context, building on our own previous research findings^[^ ^36^, ^37^ ^]^ and of other groups^[^ ^38^, ^39^, ^40^, ^41^ ^]^ on the development of pure photochemical reactions employing diazo compounds, we became interested in the potential development of a new visible light‐induced C3‐alkylation strategy of imidazo[1,2*‐a*]pyrimidines and ‐pyridines **7** ^[^ ^42^, ^43^ ^]^ with diazo compounds **8**.

## 2 Results and Discussion

We started our investigation by employing imidazo[1,2*‐a*]pyrimidine **7a** and aryldiazoacetate **8a** as reagents for our model reaction under blue light irradiation (using two LED lamps, each 15 W, *λ*_max_ = 452 nm). The irradiation of an equimolar mixture of these reagents in CHCl_3_ at rt under air for 24 h afforded the corresponding alkylated compound **9aa** in 50% yield (Entry 1, Table 1). Variations of the stoichiometry employed for reagents **7a**:**8a** showed that a 3:1 ratio (Entry 3, Table 1) or the opposite ratio (Entry 6, Table 1) both produced the same 83% yield of the desired product **9aa**, which can be considered the optimal outcomes of this series (Entries 1–7, Table 1). Interrogation on the use of other solvents, DCM, 1,2‐DCE, MeCN, AcOEt or toluene, showed inferior results when compared to CHCl_3_ (Entries 8–12, Table 1). A shorter reaction time of 15 h also led to a lower 65% yield of **9aa** (Entry 13, Table 1). Then, control experiments performed in the absence of blue light irradiation (but in the presence of ambient light) did not produce any trace amount of the desired compound **9aa** when reacting at rt (Entry 14, Table 1), or when heating at 40 °C (Entry 15, Table 1). Only when the thermal reaction was performed at 80 °C (in 1,2‐DCE) was the target product **9aa** obtained in a good 70% yield (Entry 16, Table 1). However, this yield was still lower than when using the blue light irradiation. Finally, we have also investigated the effect of reaction concentration on the yield of **9aa**. A more concentrated reaction of 0.2 M (calculated in relation to the *aza‐*arene **7a**) produced essentially the same optimal yield, 80% (Entry 17, Table 1), but a more diluted concentration of 0.05 M led to a lower 56% yield of **9aa** (Entry 18, Table 1).

**Table 1:** Optimization studies.

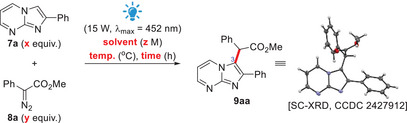						
Entry	x	y	Solvent (z M)	Temp. (°C)	Time (h)	Yield 9aa (%)^a)^
1	1	1	CHCl_3_ (0.1)	Rt	24	50
2	2	1	CHCl_3_ (0.1)	rt	24	68
**3**	**3**	**1**	**CHCl_3_ (0.1)**	**rt**	**24**	**83 (80)** ^b)^
4	4	1	CHCl_3_ (0.1)	rt	24	72
5	1	2	CHCl_3_ (0.1)	rt	24	70
**6**	**1**	**3**	**CHCl_3_ (0.1)**	**rt**	**24**	**83 (81)** ^b)^
7	1	4	CHCl_3_ (0.1)	rt	24	67
8	1	3	DCM (0.1)	rt	24	70
9	1	3	1,2‐DCE (0.1)	rt	24	74
10	1	3	MeCN (0.1)	rt	24	75
11	1	3	AcOEt (0.1)	rt	24	71
12	1	3	toluene (0.1)	rt	24	50
13	1	3	CHCl_3_ (0.1)	rt	15	65
14^c)^	1	3	CHCl_3_ (0.1)	rt	24	<10
15^c)^	1	3	CHCl_3_ (0.1)	40	24	<10
16^c)^	1	3	1,2‐DCE (0.1)	80	24	70
17	1	3	CHCl_3_ (0.2)	rt	24	80
18	1	3	CHCl_3_ (0.05)	rt	24	56

^a)^ Estimated yield based on ^1^H NMR analysis of the crude reaction mixture using 1,3,5‐trimethoxybenzene as an internal standard.

^b)^ Isolated yield.

^c)^ Performed under ambient light.

**Scheme 1:**
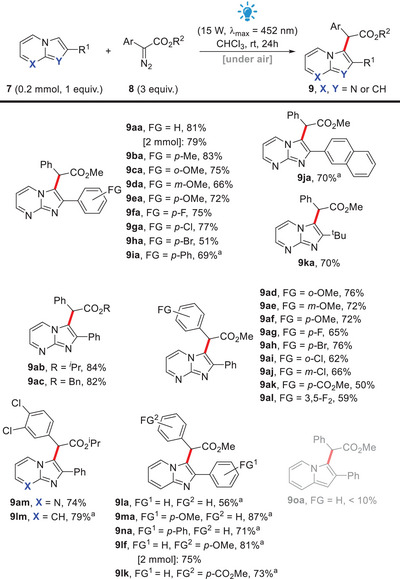
Substrate scope (Notation: reaction of **7x** with **8y** leads to product **9xy**). ^a)^ Reaction performed with 4 equiv. of the diazo compound **8**.

With the optimal reaction condition in hand, we moved forward to evaluate the scope of this transformation (Scheme 1). We first examined the reaction of different C2‐arylated imidazo[1,2‐*a*]pyrimidines **7b**–**7j** and the C2‐alkylated derivative **7k** with aryldiazoacetate **8a**, and yields in the range of 51%–83% for the corresponding compounds **9ba**–**9ka** were obtained. Then, we treated imidazo[1,2*‐a*]pyrimidine **7a** with different aryldiazoacetates **8b**–**8m** aiming at the synthesis of the corresponding C3‐alkylated imidazo[1,2*‐a*]pyrimidines **9ab**–**9am**, which could be successfully prepared in the range of 50%–84% yields (Scheme 1).

At this point, we became interested in investigating the potential C3‐alkylation of other *aza‐*arenes, such as imidazo[1,2*‐a*]pyridines and indolizines. The use of the same photochemical protocol for the C3‐alkylation of C2‐arylated imidazo[1,2*‐a*]pyridines also proceeded well. Compounds **9** **lm**–**9lk** could be prepared in a somewhat similar range of yields, 56 – 87%. However, to our surprise, our attempts to alkylate indolizine **7o** with aryldiazoacetate **8a** (presumably aiming at product **9oa**) failed, leading to a complex mixture (Scheme 1).

During the investigation of the reaction scope, when moving from imidazo[1,2*‐a*]pyrimidines to ‐pyridines, we observed more impurities in the crude reaction mixture and a lower conversion for the desired alkylated compounds. This could be somewhat compensated for by using a larger excess of the diazo compound (4 equiv.), which led to improved yields of **9**.

Notwithstanding, even more degradation was observed when attempting the alkylation of indolizine **7o** with aryldiazoacetate **8a** and the desired compound **9oa** could not be observed by ^1^H NMR in any significant extension in the crude reaction mixture.

In order to better understand this difference in reactivity among the three *aza*‐arenes, we decided to look into the frontier molecular orbitals of representative compounds **7a**, **7l,** and **7o** and the carbene **10a**, derived from diazo compound **8a**, which presumably reacts via a singlet state^[^ ^44^ ^]^ (see the Supporting Information for more details).

All molecules were optimized and had their frequencies calculated at standard temperature and pressure using the M06‐2X/aug‐cc‐pVTZ level in Gaussian 16.^[^ ^45^ ^]^ The lack of imaginary frequencies was used to characterize true minima. Solvent effects were included using the IEFPCM method^[^ ^46^ ^]^ with parameters of CHCl_3_.

**Figure 2:**
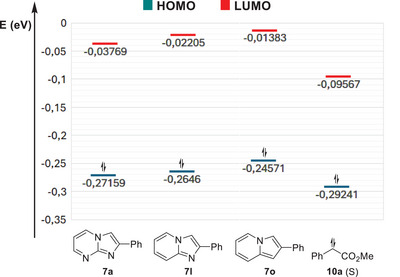
Energy levels of frontier molecular orbitals of imidazo[1,2*‐a*]pyrimidine **7a**, imidazo[1,2*‐a*]pyridine **7l**, indolizine **7o,** and singlet carbene **10a**.

In terms of energy, the smaller gaps were found between the HOMOs of the *aza*‐arenes **7a**, **7l,** and **7o** and the LUMO of the free carbene **10a**, thus suggesting that these are the main frontier molecular orbitals involved in the C‐C bond forming event, and that these *aza*‐arenes are reacting as nucleophiles, while the carbene is initially reacting as an electrophile. The energy level of the HOMO of the indolizine **7o** was higher than the HOMO of the imidazo[1,2*‐a*]pyridine **7l**, which in turn was higher than the HOMO of imidazo[1,2*‐a*]pyrimidine **7a** (Figure 2). Furthermore, it was possible to observe more pronounced C3 coefficients in the HOMO of all three *aza*‐arenes (See the Supporting Information). Our current working hypothesis is that indolizine **7o** is too reactive and more readily leads to other compounds (thus forming a complex mixture), while imidazo[1,2*‐a*]pyridine **7l** is somewhat less reactive, and imidazo[1,2*‐a*]pyrimidine **7a** is the least reactive of the three compounds. Consequently, imidazo[1,2*‐a*]pyrimidine **7a** seems to be more compatible with the highly reactive carbene intermediate formed, thus affording an overall more well‐behaved reaction system. In addition, we have also remarked that the three *aza*‐arenes **7a**, **7l,** and **7o** are not perfectly photostable under blue light irradiation and some minor degradation was produced after 24 h for each of them, only by allowing them to stir at rt in CHCl_3_ under blue light irradiation (See the Supporting Information for details).

**Scheme 2:**
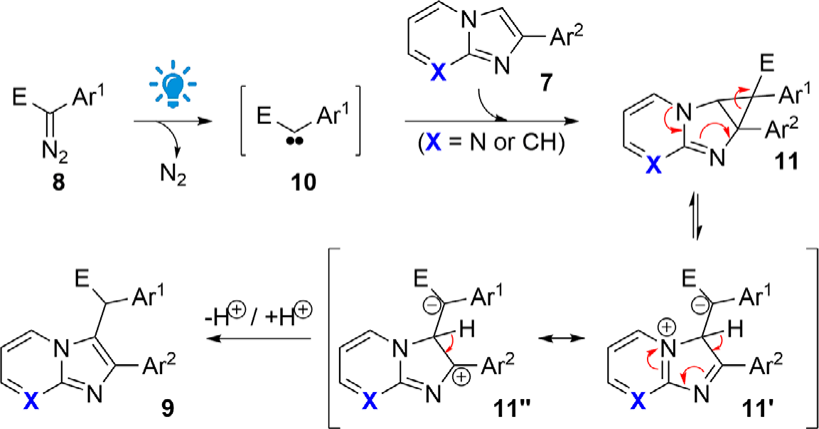
Proposed reaction mechanism. E = ester group.

In terms of reaction mechanism, during our investigations, we did not observe any experimental evidence of a cyclopropane intermediate being formed during the transformation, but DFT calculations suggest an easy cyclopropanation event (see the Supporting Information for more details). Our proposal for the reaction mechanism is that it starts with the blue light‐promoted photolysis of the aryldiazoacetate **8** to afford a free carbene intermediate **10**.^[^ ^47^ ^]^ This intermediate reacts with the *aza*‐arene **7**, thus presumably leading to the cyclopropanated compound **11**, which is likely in equilibrium with the zwitterions **11'** /**11''**. Then, a proton transfer event involving **11'**/ **11''** should lead to the observed product **9** (Scheme 2).

Finally, we became interested in evaluating other types of diazo compounds under our blue light‐promoted protocol, while also investigating the possibility of a synthetic application involving the preparation of zolpidem. While this drug molecule has been previously prepared by several different strategies,^[^ ^25^, ^26^, ^27^, ^28^, ^29^, ^30^, ^31^, ^32^, ^33^, ^34^, ^35^ ^]^ including different photocatalyzed alkylation steps,^[^ ^32^, ^33^, ^35^ ^]^ no pure photochemical transformation relying only on visible light has been previously reported. Therefore, we imagined that our strategy could be an useful complementary approach to these previous methods.

**Scheme 3:**
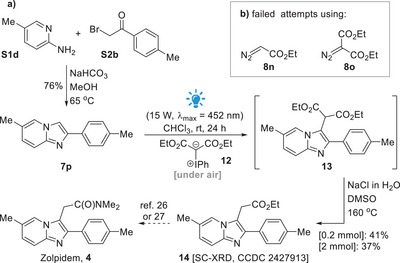
a) Formal synthesis of zolpidem. b) Diazo compounds investigated for the photochemically‐promoted key C3‐alkylation step aiming at intermediate **14**.

The required imidazo[1,2*‐a*]pyridine precursor **7p** could be readily accessed in one step from 5‐methylpyridin‐2‐amine **S1d** and 2‐bromo‐1‐(*p‐*tolyl)ethan‐1‐one **S2b** in 76% yield (Scheme 3a, see also the Supporting Information). As opposed to aryldiazoacetates **8a**–**8m**, which are donor–acceptor diazo compounds known to approximately absorb in the region of 380–480 nm, the only‐acceptor diazo compound ethyl diazoacetate **8n** and the acceptor–acceptor diazo compound diethyl 2‐diazomalonate **8o** are known to absorb at shorter wavelength regions, approximately at 340–440 nm and 320–400 nm, respectively.^[^ ^47^ ^]^ In our hands, all our attempts to perform C3‐alkylations of compound **7p** with diazo compounds **8n** or **8o** under blue light irradiation failed (Scheme 3b, see also the Supporting Information).

At this point, we turned our attention to the use of the hypervalent iodine compound **12**, which is known to also react under blue light irradiation^[^ ^48^ ^]^ and we were delighted to observe that the desired alkylation event could be successfully performed to access intermediate **13**. Then, a telescoped Krapcho decarboxylation step^[^ ^35^ ^]^ allowed the preparation of the key intermediate **14** in a combined 41% isolated yield for these two steps. Of note, a 51% yield was estimated for **13** in the first step using an internal reference (while 40% of **7p** remained unreacted). This alkylation could be also promoted in the absence of the light, at rt, but with a slight decrease to 44% for the estimated yield of **13** (see the Supporting Information for more details). This approach represents a formal synthesis of zolpidem, as intermediate **14** has been previously converted to the target drug following procedures described by Namboothiri and co‐workers,^[^ ^26^ ^]^ as well as Ley and co‐workers^[^ ^27^ ^]^ (Scheme 3).

In terms of mechanism, we speculate that this key alkylation step could possibly proceed also via a free carbene intermediate^[^ ^49^, ^50^ ^]^ or via an excited diradical intermediate derived from **12**.^[^ ^48^, ^51^ ^]^ Notwithstanding, an electron donor–acceptor (EDA) complex seems to be formed in the reaction mixture of **7p** with **12**, as a new absorption band can be observed^[^ ^52^ ^]^ (see Supporting Information for UV–Vis absorbance spectra). This EDA complex could also possibly play a role in the reaction mechanism. Spectroscopic and EPR analyses, as well as kinetic measurements could be important to better ascertain the mechanistic scenario involved in this step. We did not investigate this mechanism in greater detail.

## 3 Conclusions

In summary, we have developed a new photochemical method for the formal C─H insertion of imidazo[1,2*‐a*]pyrimidines and imidazo[1,2*‐a*]pyridines onto aryldiazoacetates solely based on the use of blue light as the promoting agent. This transformation can be performed in a convenient manner under air and at rt to afford the corresponding C3‐alkylated products in good yields. These compounds represent analogs of a key intermediate previously reported in the preparation of zolpidem. Furthermore, this key intermediate can be also accessed by using a hypervalent iodine compound as the alkylating agent and performing a sequential Krapcho decarboxylation step.

The main points of the current work were to show how one can take advantage of the presented strategies to perform C3‐alkylation of imidazo[1,2*‐a*]pyrimidines and ‐pyridines with aryldiazoacetates and to understand the difference in reactivity observed between the *aza*‐arenes employed (also including indolizines, by means of a representative member of this class, **7o**). Possibly, these new synthetic technologies could be valuable in future applications in material science or in chemical biology programs.

## Supporting Information

The authors have cited additional references within the Supporting Information.^[^ ^1^, ^2^, ^3^, ^4^, ^5^, ^6^, ^7^, ^8^, ^9^, ^10^, ^11^, ^12^, ^13^, ^14^, ^15^, ^16^, ^17^, ^18^, ^19^, ^20^, ^21^, ^22^, ^23^, ^24^ ^]^ FID files associated with the NMR spectra of all synthesized compounds are available at Zenodo at https://doi.org/10.5281/zenodo.15001166. Crystallographic data associated with the single crystal X‐ray diffraction (SC‐XRD) of **7a**, **9aa,** and **14** have been deposited at the Cambridge Crystallographic Data Centre (CCDC 2427911 ‐ 2427913, respectively) and can be accessed via https://www.ccdc.cam.ac.uk/structures.

## Conflict of Interests

The authors declare no conflict of interest.

## Supporting information



Supporting Information

Supporting Information
