# Supplementary material for: Visible Light‐Mediated Preparation of a Key Intermediate Employed in the Synthesis of Zolpidem and Several Analogs
Source: Chem Asian J. 2025 May 13;20(16):e00455. doi: 10.1002/asia.202500455 (PMC12392708; doi:10.1002/asia.202500455)

Supporting Information for:

Visible Light-Mediated Preparation of a Key Intermediate Employed in the Synthesis of Zolpidem and Several Analogs

Ronei M. S. Souza,[a] Tales A. C. Goulart,[a] Roberto do C. Pinheiro,[a] Felipe F. do C. Sonaglio,[a] Deborah de A. Simoni,[b] Rodrigo A. Cormanich,[a] Igor D. Jurberg[a]*

[a]Institute of Chemistry, State University of Campinas, 13083-862, Campinas, SP, Brazil
[b]Institutional X-Ray Laboratory, Institute of Chemistry, State University of Campinas, 13083-862, Campinas, SP, Brazil

*E-mail: ijurberg@unicamp.br

Table of Contents

1. Materials and Methods S3
1.1. Starting Substrates Employed in this Work S4
1.2. Synthesis of Molecules Reported in this Work S5
1.2.1. Imidazo[1,2-*a*]pyrimidines and Imidazo[1,2-*a*]pyridines **7** S5
General Procedure A S5
1.2.2. Diazo Compounds **8** S14
1.2.3. Hypervalent Iodine Compound **12** S16
1.2.4. Formal C-H Insertion of *aza*-Arenes **7** onto Aryldiazoacetates **8** S17
General Procedure B S17
1.2.5. Preliminary Investigations Aiming at the Blue Light-Mediated Alkylation of Indolizine **7o** with Aryldiazoacetate **8a** S36
1.2.6. Formal Synthesis of Zolpidem S37
2. Theoretical Calculations S41
2.1. Frontier Molecular Orbitals S41
2.2. Cyclopropanation of *aza*-Arenes **7a** or **7l** with Aryldiazoacetate **8a** S44
3. UV/ Vis Absorbance Spectra of Isolated Compounds and Mixtures S63
4. Evaluation of the Photostability of *aza*-Arenes **7a**, **7l** and **7o** under Blue Light Irradiation in $CHCl_3$ S69
5. Copies of ^{1}H, ^{13}C{^{1}H} and ^{19}F{^{1}H} NMR Spectra of Compounds S71

1. Materials and Methods

All reactions were carried out under air, in oven dried glassware with magnetic stirring, unless otherwise noted. All reagents employed in this work were purchased from Sigma-Aldrich/Merck or Oakwood and used as such without further purification. All solvents employed in the reactions were distilled from appropriate drying agents prior to use. Organic solutions were concentrated under reduced pressure on rotary evaporators IKA RV-10 Control or Buchi R-100. Reactions were monitored by thin-layer chromatography (TLC) on Silica gel 60 F_{254} plastic plates (Merck). Chromatograms were visualized by fluorescence quenching with UV light at 254 nm and/or by staining using phosphomolybdic acid. Flash column chromatography was performed using Merck silica gel 60 (particle size 35-70µm). ^{1}H and $^{13}C\{^{1}H\}$ NMR spectra were recorded on Bruker AV–250, 300, 400, 500 or 600. Chemical shifts (δ) are given in parts per million, referenced to the residual peak of $CDCl_3$, δ = 7.26 (^{1}H NMR) and δ = 77.16 ($^{13}C\{^{1}H\}$ NMR) or of d_6-DMSO, δ = 2.50 (^{1}H NMR) and δ = 39.52 ($^{13}C\{^{1}H\}$ NMR) as internal references.[1] The following abbreviations were used to designate chemical shift multiplicities: s = singlet, d = doublet, t = triplet, q = quartet, sept. = septet, m = multiplet, br s = broad singlet. High-resolution mass spectra were recorded on Q Exactive Orbitrap spectrometer working with an electrospray ionization (ESI). Infrared spectra were performed on the Agilent Cary 630 FTIR spectrometer. Melting points were measured on Metler Toledo MP50 Melting Point System and are uncorrected. Compounds **7a**, **9aa** and **14** had their structures confirmed by single crystal X-ray diffraction technique, performed in a Bruker APEX II duo CCD area detector diffractometer. The crystallographic information files were validated by the CheckCIF server and deposited in Cambridge Structural Database."

The blue LED lamps employed in this work for photochemical reactions were bought from manufacturer Westinghouse: model PAR38, 15W, 120V, Blue E26 (Medium), Base Outdoor LED Flood Reflector Light Bulb 33151.

[1] G. R. Fulmer, A. J. M. Miller, N. H. Sherden, H. E. Gottlieb, A. Nudelman, B. M. Stoltz, J. E. Bercaw, K. I. Goldberg, *Organometallics* **2010**, *29*, 2176 – 2179.

1.1. Starting Substrates Employed in this Work

The following *aza*-arenes were purchased from commercial sources and used directly from their bottles without any treatment or additional purification step: pyrimidin-2-amine (**S1a**), pyridin-2-amine (**S1b**), 2-methylpyridine (**S1c**) and 5-methylpyridin-2-amine (**S1d**) (Figure S1).

Figure S1. Commercially available *aza*-arenes employed in this work.

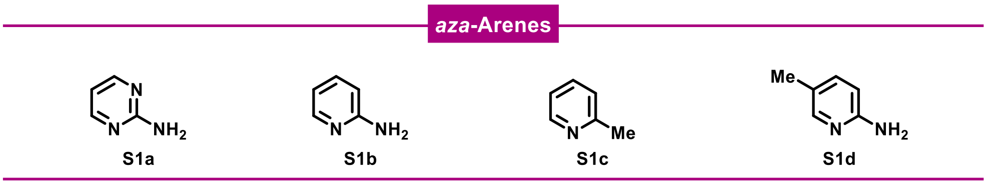

The following 2-bromoacetophenones were purchased from commercial sources and used directly from their bottles without any treatment or additional purification step: 2-bromo-1-phenylethan-1-one (**S2a**), 2-bromo-1-(p-tolyl)ethan-1-one (**S2b**), 2-bromo-1-(2-methoxyphenyl)ethan-1-one (**S2c**), 2-bromo-1-(3-methoxyphenyl)ethan-1-one (**S2d**), 2-bromo-1-(4-methoxyphenyl)ethan-1-one (**S2e**), 2-bromo-1-(4-fluorophenyl)ethan-1-one (**S2f**), 2-bromo-1-(4-chlorophenyl)ethan-1-one (**S2g**), 2-bromo-1-(4-bromophenyl)ethan-1-one (**S2h**), 1-([1,1'-biphenyl]-4-yl)-2-bromoethan-1-one (**S2i**), 2-bromo-1-(naphthalen-2-yl)ethan-1-one (**S2j**), 1-bromo-3,3-dimethylbutan-2-one (**S2k**) (Figure S2).

Figure S2. Commercially available 2-bromo-ketones employed in this work.

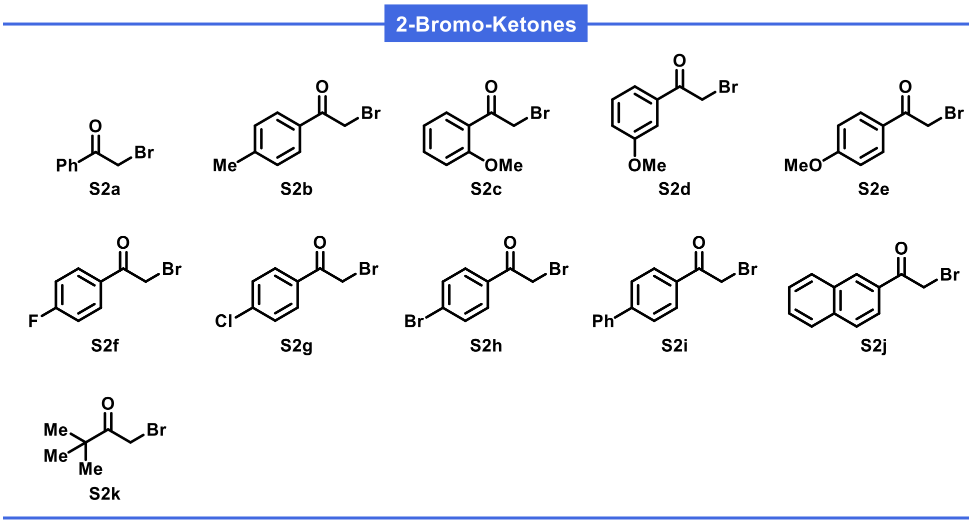

1.2. Synthesis of Molecules Reported in this Work

1.2.1. Imidazo[1,2-a]pyrimidines and Imidazo[1,2-a]pyridines 7

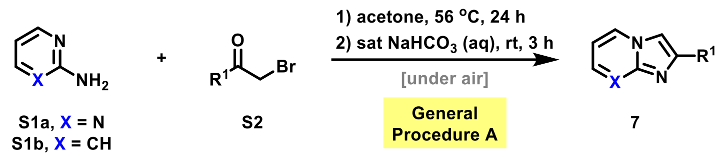

General Procedure A: *Synthesis of aza-arenes imidazo[1,2-a]pyrimidines and imidazo[1,2-a]pyridines* ***7***

Under air, at room temperature, a round bottom flask equipped with a reflux condenser is charged with pyrimidin-2-amine **S1a** or pyridin-2-amine **S1b** (1 equiv.), 2-bromo-ketones **S2** (2.5 equiv.), and acetone (0.2 M in relation to **S1a** or **S1b**). Then, the reaction mixture is heated at reflux (~ 56 °C) and stirred until the starting *aza*-heterocycles **S1a** or **S1b** is fully consumed (as indicated by TLC analysis, which typically occurred in 24 hours). At this point, the reaction mixture is allowed to cool down to room temperature, and a precipitate is usually observed. This precipitate is filtered off and thoroughly washed with cold AcOEt. This solid is then added to a saturated aqueous solution of $NaHCO_3$ and stirred at room temperature for 3 hours. Finally, the resulting mixture is extracted with $CHCl_3$ (3x), dried (Na_2SO_4), and concentrated under reduced pressure to afford the desired compound **7**. In most cases, this desired compound is obtained clean. However, if necessary, a purification step by flash column chromatography can be performed.

2-phenylimidazo[1,2-a]pyrimidine[2] (**7a**)

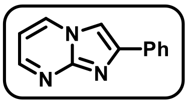

The **General Procedure A** was employed with pyrimidin-2-amine **S1a** (190 mg, 2 mmol), 2-bromo-1-phenylethan-1-one **S2a** (995 mg, 5 mmol) in acetone (10 mL). The title compound was obtained as a white solid: 288 mg, 74%.

^{1}H NMR (500 MHz, CDCl$_3$) δ: 8.50 (dd, *J* = 4.0 Hz, *J* = 2.0 Hz, 1H), 8.42 (dd, *J* = 6.5 Hz, *J* = 2.0 Hz, 1H), 8.03 – 8.00 (m, 2H), 7.81 (s, 1H), 7.45 – 7.22 (m, 2H), 7.35 (tt, *J* = 7.3 Hz, *J* = 1.2 Hz, 1H), 6.83 (dd, *J* = 6.5 Hz, *J* = 4.0 Hz, 1H).

^{13}C{^{1}H} NMR (125 MHz, CDCl$_3$) δ: 149.9, 148.8, 147.5, 133.2, 133.1, 128.9, 128.7, 126.4, 108.9, 106.3.

[2] ^{1}H and ^{13}C{^{1}H} NMR data are in good agreement with the literature. See: H. Su, L. Wang, H. Rao, H. Xu, *Org. Lett.* **2017**, *19*, 2226-2229.

Structure defined by single crystal X-ray diffraction (SC-XRD) of **7a** (CCDC 2427911)

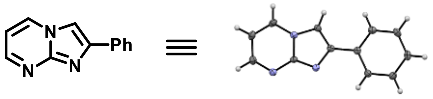

(ORTEP representation with ellipsoids showing 50% of probability)

Crystal data

$C_{12}H_9N_3$	$F(000) = 408$
$M_r = 195.22$	$D_x = 1.407$ Mg m^{-3}
Monoclinic, $P2_1/n$	Mo $K\alpha$ radiation, $\lambda = 0.71073$ Å
$a = 5.9586$ (6) Å	Cell parameters from 3696 reflections
$b = 8.8232$ (9) Å	$\theta = 2.3–27.4°$
$c = 17.5380$ (15) Å	$\mu = 0.09$ mm^{-1}
$\beta = 91.648$ (5)°	$T = 120$ K
$V = 921.66$ (15) Å^3	Plate, orange
$Z = 4$	0.17 × 0.13 × 0.02 mm

Data collection

Bruker APEX CCD detector diffractometer	2038 independent reflections
Radiation source: fine-focus sealed tube	1720 reflections with $I > 2\sigma(I)$
Detector resolution: 8.3333 pixels mm^{-1}	$R_{int} = 0.029$
phi and ω scans	$\theta_{max} = 27.1°$, $\theta_{min} = 2.3°$
Absorption correction: multi-scan *SADABS* (Bruker, 2010)	$h = -7\rightarrow7$
$T_{min} = 0.703$, $T_{max} = 0.746$	$k = -11\rightarrow11$
10837 measured reflections	$l = -22\rightarrow17$

Refinement

Refinement on F^2	Primary atom site location: structure-invariant direct methods
Least-squares matrix: full	Hydrogen site location: inferred from neighbouring sites
$R[F^2 > 2\sigma(F^2)] = 0.036$	H-atom parameters constrained
$wR(F^2) = 0.088$	$w = 1/[\sigma^2(F_o^2) + (0.0356P)^2 + 0.3647P]$ where $P = (F_o^2 + 2F_c^2)/3$
$S = 1.07$	$(\Delta/\sigma)_{max} < 0.001$
2038 reflections	$\Delta\rangle_{max} = 0.22$ e Å^{-3}
136 parameters	$\Delta\rangle_{min} = -0.22$ e Å^{-3}

0 restraints	

2-(p-tolyl)imidazo[1,2-a]pyrimidine[3] (**7b**)

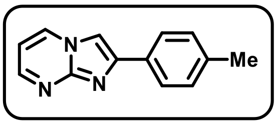

The **General Procedure A** was employed with pyrimidin-2-amine **S1a** (190 mg, 2 mmol), 2-bromo-1-(*p*-tolyl)ethan-1-one **S2b** (1.07 g, 5 mmol) and acetone (10 mL). The title compound was obtained as a white solid: 353 mg, 84%.

^{1}H NMR (500 MHz, $CDCl_3$) δ: 8.47 (dd, *J* = 4.0 Hz, *J* = 2.0 Hz, 1H), 8.40 (dd, *J* = 6.5 Hz, *J* = 2.0 Hz, 1H), 7.90 (d, *J* = 8.0 Hz, 2H), 7.75 (s, 1H), 7.24 (d, *J* = 8.0 Hz, 2H), 6.80 (dd, *J* = 6.5 Hz, *J* = 4.0 Hz, 1H), 2.38 (s, 3H).

^{13}C{^{1}H} NMR (125 MHz, $CDCl_3$) δ: 149.6, 148.8, 147.6, 138.6, 133.0, 130.4, 129.6, 126.3, 108.7, 105.9, 21.5.

M.P.: 223 – 225 °C

IR (ATR, cm^{-1}): 3119, 1613, 1522, 1502, 1487, 1354, 1226, 1220, 1187.

HRMS (ESI+, Orbitrap), calcd. for [$C_{13}H_{11}N_3$ + H^+]: 210.1026, found: 210.1024.

2-(2-methoxyphenyl)imidazo[1,2-a]pyrimidine (**7c**)

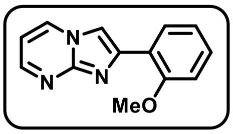

The **General Procedure A** was employed with pyrimidin-2-amine **S1a** (190 mg, 2 mmol), 2-bromo-1-(2-methoxyphenyl)ethan-1-one **S2c** (1.15 g, 5 mmol) and acetone (10 mL). The title compound was obtained as a dark red solid: 377 mg, 84%.

^{1}H NMR (400 MHz, $CDCl_3$) δ: 8.55 (dd, *J* = 7.8 Hz, *J* = 2.0 Hz, 1H), 8.48 (dd, *J* = 4.2 Hz, *J* = 2.0 Hz, 1H), 8.40 (dd, *J* = 6.5 Hz, *J* = 2.0 Hz, 1H), 8.13 (s, 1H), 7.32 (ddd, *J* = 8.4 Hz, *J* = 7.4 Hz, *J* = 2.0 Hz, 1H), 7.11 (td, *J* = 7.4 Hz, *J* = 1.2 Hz, 1H), 6.98 (dd, *J* = 8.4 Hz, *J* = 1.2 Hz, 1H), 6.79 (dd, *J* = 6.5 Hz, *J* = 4.2 Hz, 1H), 3.98 (s, 3H).

^{13}C{^{1}H} NMR (100 MHz, $CDCl_3$) δ: 157.2, 149.8, 147.7, 143.0, 133.0, 129.7, 129.4, 121.8, 121.2, 110.9, 110.7, 108.4, 55.5.

M.P.: 139 – 141 °C.

IR (ATR, cm^{-1}): 3027, 1611, 1524, 1483, 1451, 1346, 1297, 1273,1218, 1192, 1176.

HRMS (ESI+, Orbitrap), calcd. for $C_{13}H_{11}N_3O$ + H^+]: 226.0975, found: 226.0972.

[3] ^{1}H NMR is in good agreement with the literature. See: Y.-Y. Xie, *Synth. Commun.* **2005**, *35*, 1741-1746.

2-(3-methoxyphenyl)imidazo[1,2-a]pyrimidine (**7d**)

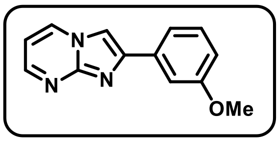

The **General Procedure A** was employed with pyrimidin-2-amine **S1a** (190 mg, 2 mmol), 2-bromo-1-(3-methoxyphenyl)ethan-1-one **S2d** (1.15 g, 5 mmol) and acetone (10 mL). The title compound was obtained as a light brown solid: 295 mg, 66%.

^{1}H NMR (400 MHz, CDCl$_3$) δ: 8.48 (ddd, *J* = 4.2 Hz, *J* = 2.0 Hz, *J* = 1.1 Hz, 1H), 8.41 (dd, *J* = 6.5 Hz, *J* = 2.0 Hz, 1H), 7.78 (s, 1H), 7.65 (t, *J* = 2.0 Hz, 1H), 7.51 (d, *J* = 7.6 Hz, 1H), 7.31 (t, *J* = 8.0 Hz, 1H), 6.89 (dd, *J* = 8.2 Hz, *J* = 2.6 Hz, 1H), 6.81 (ddd, *J* = 6.5 Hz, *J* = 4.0 Hz, *J* = 1.1 Hz, 1H), 3.87 (s, 3H).

^{13}C{^{1}H} NMR (100 MHz, CDCl$_3$) δ: 160.2, 149.9, 148.7, 147.2, 134.6, 133.1, 129.8, 118.6, 115.0, 111.3, 108.9, 106.5, 55.5.

M.P.: 141 – 143 ^{o}C.

IR (ATR, cm^{-1}): 3063, 1609, 1581, 1504, 1482, 1354, 1282, 1168, 1041.

HRMS (ESI+, Orbitrap), calcd. for[$C_{13}H_{11}N_3O$ + H^+]: 226.0975, found: 226.0972.

2-(4-methoxyphenyl)imidazo[1,2-a]pyrimidine[3] (**7e**)

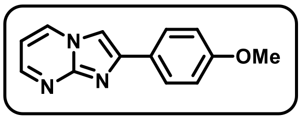

The **General Procedure A** was employed with pyrimidin-2-amine **S1a** (190 mg, 2 mmol), 2-bromo-1-(4-methoxyphenyl)ethan-1-one **S2e** (1.15 g, 5 mmol) and acetone (10 mL). The title compound was obtained as a white solid: 363 mg, 81%.

^{1}H NMR (500 MHz, CDCl$_3$) δ: 8.48 (dd, *J* = 4.0 Hz, *J* = 2.0 Hz, 1H), 8.40 (dd, *J* = 6.7 Hz, *J* = 2.0 Hz, 1H), 7.95 (d, *J* = 9.0 Hz, 2H), 7.72 (s, 1H), 6.98 (d, *J* = 9.0 Hz, 2H), 6.82 (dd, *J* = 6.7 Hz, *J* = 4.0 Hz, 1H), 3.85 (s, 3H).

^{13}C{^{1}H} NMR (125 MHz, CDCl$_3$) δ: 160.2, 149.6, 148.8, 147.5, 132.8, 127.7, 125.9, 114.3, 108.7, 105.3, 55.5.

M.P.: 203 – 205 ^{o}C.

IR (ATR, cm^{-1}): 3058, 1619, 1520, 1488, 1239, 1224, 1115, 1080.

HRMS (ESI+, Orbitrap), calcd. for[$C_{13}H_{11}N_3O$ + H^+]: 226.0975, found: 226.0972.

2-(4-fluorophenyl)imidazo[1,2-a]pyrimidine[4] (**7f**)

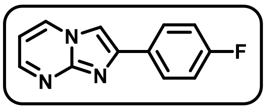

The **General Procedure A** was employed with pyrimidin-2-amine **S1a** (190 mg, 2 mmol), 2-bromo-1-(4-fluorophenyl)ethan-1-one **S2f** (1.09 g, 5 mmol) and acetone (10 mL). The title compound was obtained as a white solid: 190 mg, 45%.

^{1}H NMR (500 MHz, *d*$_6$-DMSO) δ: 8.96 (dd, *J* = 6.7 Hz, *J* = 2.1 Hz, 1H), 8.53 (dd, *J* = 4.0 Hz, *J* = 2.1 Hz, 1H), 8.36 (s, 1H), 8.05 (dd, *J* = 8.9 Hz, *J* = 5.8 Hz, 2H), 7.30 (t, *J* = 8.9 Hz, 2H), 7.05 (dd, *J* = 6.7 Hz, *J* = 4.0 Hz, 1H).

^{13}C{^{1}H} NMR (125 MHz, *d*$_6$-DMSO) δ: 162.1 (d, *J* = 243.8 Hz), 150.4, 148.0, 144.4, 135.1, 130.0 (d, *J* = 2.5 Hz), 127.8 (d, *J* = 8.8 Hz), 115.7 (d, *J* = 22.5 Hz), 108.9, 107.4.

^{19}F{^{1}H} NMR (470 MHz, *d*$_6$-DMSO) δ: -113.7.

2-(4-chlorophenyl)imidazo[1,2-a]pyrimidine[5] (**7g**)

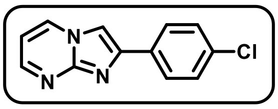

The **General Procedure A** was employed with pyrimidin-2-amine **S1a** (190 mg, 2 mmol), 2-bromo-1-(4-chlorophenyl)ethan-1-one **S2g** (1.17 g, 5 mmol) and acetone (10 mL). The title compound was obtained as a white solid: 311 mg, 68%.

^{1}H NMR (300 MHz, *d*$_6$-DMSO) δ: 8.93 (dd, *J* = 6.8 Hz, *J* = 2.1 Hz, 1H), 8.53 (dd, *J* = 4.2 Hz, *J* = 2.1 Hz, 1H), 8.34 (s, 1H), 8.01 (d, *J* = 8.6 Hz, 2H), 7.51 (d, *J* = 8.6 Hz, 2H), 7.04 (dd, *J* = 6.8 Hz, *J* = 4.2 Hz, 1H).

^{13}C{^{1}H} NMR (75 MHz, *d*$_6$-DMSO) δ: 150.2, 147.9, 144.0, 134.7, 132.5, 132.2, 128.5, 127.2, 108.7, 107.5.

M.P.: 279 - 281 ^{o}C.

IR (ATR, cm^{-1}): 3113, 1614, 1522, 1501, 1476, 1354, 1014.

HRMS (ESI+, Orbitrap), calcd. for[$C_{12}H_8ClN_3$ + H^+]: 230.0480, found: 230.0479.

[4] ^{1}H and ^{13}C{^{1}H} NMR data are in good agreement with the literature. See: B. Mu, J. Li, D. Zou, Y. Wu, J. Chang, Y. Wu, *Tetrahedron Lett.* **2017**, *58*, 4816-4821.
[5] ^{1}H NMR data is in good agreement with the literature. See: Z. Liu, Z.-C. Chen, Q.-G. Zheng, *J. Heterocyclic Chem.* **2003**, *40*, 909-911.

2-(4-bromophenyl)imidazo[1,2-a]pyrimidine[5] (**7h**)

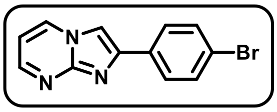

The **General Procedure A** was employed with pyrimidin-2-amine **S1a** (190 mg, 2 mmol), 2-bromo-1-(4-bromophenyl)ethan-1-one **S2h** (1.39 g, 5 mmol) and acetone (10 mL). The title compound was obtained as a white solid: 407 mg, 74%.

^{1}H NMR (300 MHz, *d$_6$*-DMSO) δ: 8.93 (dd, *J* = 6.7 Hz, *J* = 2.0 Hz, 1H), 8.53 (dd, *J* = 4.2 Hz, *J* = 2.0 Hz, 1H), 8.36 (s, 1H), 7.95 (d, *J* = 8.6 Hz, 2H), 7.65 (d, *J* = 8.6 Hz, 2H), 7.04 (dd, *J* = 6.7 Hz, *J* = 4.2 Hz, 1H).

^{13}C{^{1}H} NMR (75 MHz, *d$_6$*-DMSO) δ: 150.2, 147.8, 144.0, 134.6, 132.5, 131.3, 127.4, 120.9, 108.6, 107.5.

M.P.: 280 – 282 ºC.

IR (ATR, cm^{-1}): 3111, 1614, 1521, 1500, 1354, 1237, 1069, 1010.

HRMS (ESI+, Orbitrap), calcd. for [$C_{12}H_8BrN_3$ + H^+]: 273.9974, found: 273.9974.

2-([1,1'-biphenyl]-4-yl)imidazo[1,2-a]pyrimidine[6] (**7i**)

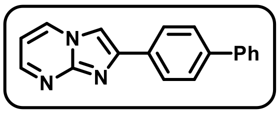

The **General Procedure A** was employed with pyrimidin-2-amine **S1a** (190 mg, 2 mmol) in acetone (10 mL). 1-([1,1'-biphenyl]-4-yl)-2-bromoethan-1-one **S2i** (1.38 g, 5 mmol) and acetone (10 mL). The title compound was obtained as a white solid: 493 mg, 91%.

^{1}H NMR (600 MHz, *d$_6$*-DMSO) δ: 8.93 (dd, *J* = 6.8 Hz, *J* = 2.0 Hz, 1H), 8.53 (dd, *J* = 4.2 Hz, *J* = 2.0 Hz, 1H), 8.36 (s, 1H), 8.09 (d, *J* = 8.4 Hz, 2H), 7.77 (d, *J* = 8.4 Hz, 2H), 7.72 (d, *J* = 7.8 Hz, 2H), 7.49 (t, *J* = 7.8 Hz, 2H), 7.38 (t, *J* = 7.8 Hz, 1H), 7.04 (dd, *J* = 6.8 Hz, *J* = 4.2 Hz, 1H).

^{13}C{^{1}H} NMR (150 MHz, *d$_6$*-DMSO) δ: 149.9, 147.9, 144.8, 139.7, 139.5, 134.5, 132.3, 128.6, 127.1, 126.6, 126.2, 126.0, 108.5, 107.3.

M.P.: 274 – 275 ºC.

IR (ATR, cm^{-1}): 3102, 1615, 1521, 1500, 1477, 1378, 1351, 1232, 1182.

HRMS (ESI+, Orbitrap), calcd. for[$C_{18}H_{13}N_3$ + H]$^+$: 272.1182, found: 272.1180.

[6] ^{1}H NMR data is in good agreement with the literature. See: D. Xu, B. Liu, M. Zheng, *J. Chem. Res.* **2003**, *2003*, 645-647.

2-(naphthalen-2-yl)imidazo[1,2-a]pyrimidine (**7j**)

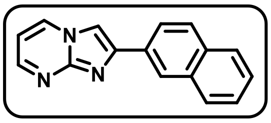

The **General Procedure A** was employed with pyrimidin-2-amine **S1a** (190 mg, 2 mmol), 2-bromo-1-(naphthalen-2-yl)ethan-1-one **S2j** (1.25 g, 5 mmol) and acetone (10 mL). The title compound was obtained as a white solid: 265 mg, 54%.

^{1}H NMR (500 MHz, CDCl$_3$) δ: 8.61 (s, 1H), 8.55 (dd, *J* = 4.4 Hz, *J* = 2.0 Hz, 1H), 8.44 (dd, *J* = 6.7 Hz, *J* = 2.0 Hz, 1H), 8.06 (dd, *J* = 8.5 Hz, *J* = 2.0 Hz, 1H), 7.94 – 7.89 (m, 3H), 7.86 – 7.84 (m, 1H), 7.52 – 7.47 (m, 2H), 6.85 (dd, *J* = 6.7 Hz, *J* = 4.4 Hz, 1H).

^{13}C{^{1}H} NMR (125 MHz, CDCl$_3$) δ: 150.1, 149.0, 147.5, 133.8, 133.7, 133.0, 130.5, 128.7, 128.5, 127.9, 126.5, 126.4, 125.7, 124.1, 108.9, 106.7.

M.P.: 240 – 241 ^{o}C.

IR (ATR, cm^{-1}): 3143, 3074, 1612, 1519, 1510, 1492, 1347, 1225, 1179.

HRMS (ESI+, Orbitrap), calcd. for [$C_{16}H_{11}N_3$ + H^+]: 246.1026, found: 246.1022.

2-(tert-butyl)imidazo[1,2-a]pyrimidine[7] (**7k**)

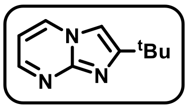

The **General Procedure A** was employed with pyrimidin-2-amine **S1a** (190 mg, 2 mmol), 1-bromo-3,3-dimethylbutan-2-one **S2k** (895 mg, 5 mmol) and acetone (10 mL). The title compound was isolated as a light brown solid: 215 mg, 61%.

^{1}H NMR (300 MHz, CDCl$_3$) δ: 8.44 (dd, *J* = 4.2 Hz, *J* = 2.0 Hz, 1H), 8.35 (dd, *J* = 6.7 Hz, *J* = 2.0 Hz, 1H), 7.29 (s, 1H), 6.76 (dd, *J* = 6.7 Hz, *J* = 4.2 Hz, 1H), 1.40 (s, 9H).

^{13}C{^{1}H} NMR (75 MHz, CDCl$_3$) δ: 159.4, 149.0, 148.3, 132.9, 108.2, 105.0, 32.8, 30.1.

M.P.: 189 – 191 ^{o}C.

IR (ATR, cm^{-1}): 2958, 1616, 1502, 1234.

HRMS (ESI+, Orbitrap), Calcd. for [$C_{10}H_{13}N_3$ + H]$^+$: 176.1182, found: 176.1179.

2-phenylimidazo[1,2-a]pyridine[2] (**7l**)

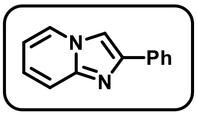

The **General Procedure A** was employed with pyridin-2-amine **S1b** (470 mg, 5 mmol), 2-bromo-1-phenylethan-1-one **S2a** (2.49 g, 12.5

[7] ^{1}H and ^{13}C{^{1}H} NMR data are in good agreement with the literature. See: S. K. Ghosh, D. Ghosh, R. Maitra, Y.-T. Kuo, H. M. Lee, *Eur. J. Org. Chem.* **2016**, *2016*, 5722-5731.

mmol) and acetone (10 mL). The title compound was obtained as a white solid: 735 mg, 76%.

^{1}H NMR (600 MHz, CDCl$_3$) δ: 8.02 (d, *J* = 6.6 Hz, 1H), 7.93 (d, *J* = 7.2 Hz, 2H), 7.78 (s, 1H), 7.62 (d, *J* = 9.0 Hz, 1H), 7.40 (t, *J* = 7.6 Hz, 2H), 7.30 (t, *J* = 7.6 Hz, 1H), 7.12 (dd, *J* = 9.0 Hz, *J* = 7.2 Hz, 1H), 6.70 (t, *J* = 6.6 Hz, 1H).

^{13}C{^{1}H} NMR (150 MHz, CDCl$_3$) δ: 145.7 (x2), 133.8, 128.7, 128.0, 126.1, 125.7, 124.7, 117.5, 112.5, 108.2.

2-(4-methoxyphenyl)imidazo[1,2-a]pyridine[2] (**7m**)

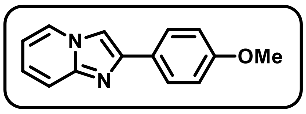

The **General Procedure A** was employed with pyridin-2-amine **S1b** (188 mg, 2 mmol), 2-bromo-1-(4-methoxyphenyl)ethan-1-one **S2e** (1.15 g, 5 mmol) and acetone (10 mL). The title compound was obtained as a grayish solid: 273 mg, 61%.

^{1}H NMR (400 MHz, CDCl$_3$) δ: 8.02 (dt, *J* = 6.8 Hz, *J* = 1.2 Hz, 1H), 7.86 (d, *J* = 8.8 Hz, 2H), 7.70 (s, 1H), 7.58 (d, *J* = 9.2 Hz, 1H), 7.10 (ddd, *J* = 9.2 Hz, *J* = 6.8 Hz, *J* = 1.2 Hz, 1H), 6.95 (d, *J* = 8.8 Hz, 2H), 6.69 (td, *J* = 6.8 Hz, *J* = 1.2 Hz, 1H), 3.82 (s, 3H).

^{13}C{^{1}H} NMR (100 MHz, CDCl$_3$) δ: 159.6, 145.8, 145.7, 127.3, 126.6, 125.5, 124.5, 117.3, 114.2, 112.2, 107.3, 55.4.

2-([1,1'-biphenyl]-4-yl)imidazo[1,2-a]pyridine[8] (**7n**)

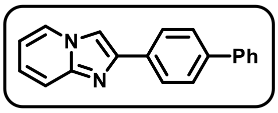

The **General Procedure A** was employed with pyridin-2-amine **S1b** (188 mg, 2 mmol), 1-([1,1'-biphenyl]-4-yl)-2-bromoethan-1-one **S2i** (1.38 g, 5 mmol) and acetone (10 mL). The title compound was obtained as a white solid: 448 mg, 83%.

^{1}H NMR (400 MHz, CDCl$_3$) δ: 8.08 (d, *J* = 6.8 Hz, 1H), 8.03 (d, *J* = 8.0 Hz, 2H), 7.86 (s, 1H), 7.69 – 7.63 (m, 5H), 7.45 (t, *J* = 7.4 Hz, 2H), 7.35 (t, *J* = 7.4 Hz, 1H), 7.16 (t, *J* = 8.0 Hz, 1H), 6.75 (t, *J* = 6.8 Hz, 1H).

^{13}C{^{1}H} NMR (100 MHz, CDCl$_3$) δ: 145.9, 145.6, 140.8, 140.7, 132.9, 128.9, 127.5, 127.4, 127.1, 126.5, 125.7, 124.8, 117.6, 112.5, 108.3.

[8] ^{1}H and ^{13}C{^{1}H} NMR data are in good agreement with the literature. See: G. Cao, Z. Chen, J. Song, J. Xu, M. Miao, H. Ren, *Adv. Synth. Catal.* **2018**, *360*, 881-886.

2-phenylindolizine[9] (**7o**)

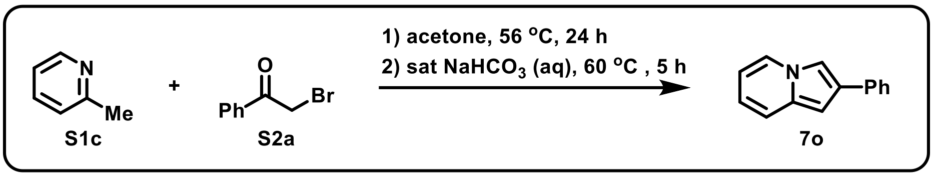

Under air, at room temperature, a round bottom flask equipped with a reflux condenser was charged with 2-methylpyridine **S1c** (186 mg, 2 mmol, 1 equiv.), 2-bromo-1-phenylethan-1-one **S2a** (995 mg, 5 mmol, 2.5 equiv.), and acetone (10 mL, 0.2 M in relation to **S1c**). Then, the reaction mixture was heated to reflux (~ 56 °C) and stirred at this temperature until the complete consumption of **S1c** (as indicated by TLC analysis, typically occurring in 24 hours). At this point, the reaction mixture was allowed to cool down to room temperature, and a precipitate was observed. This precipitate was filtered off and thoroughly washed with cold AcOEt. This solid was added to an aqueous saturated solution of $NaHCO_3$ and stirred at 60 °C for 5 hours. Then, the mixture was extracted with $CHCl_3$ (3x), dried (Na_2SO_4), and concentrated under reduced pressure to afford the title product as a white solid: 343 mg, 89%.

^{1}H NMR (600 MHz, *d$_6$*-DMSO) δ: 8.21 (dd, *J* = 7.2 Hz, *J* = 1.2 Hz, 1H), 7.96 (d, *J* = 1.2 Hz, 1H), 7.70 – 7.69 (m, 2H), 7.40 – 7.37 (m, 3H), 7.23 (tt, *J* = 7.2 Hz, *J* = 1.2 Hz, 1H), 6.76 (s, 1H), 6.68 (ddd, *J* = 9.0 Hz, *J* = 6.0 Hz, *J* = 1.2 Hz, 1H), 6.53 (td, *J* = 6.6 Hz, *J* = 1.2 Hz, 1H).

^{13}C{^{1}H} NMR (150 MHz, *d$_6$*-DMSO) δ: 134.9, 132.9, 128.8, 128.2, 126.4, 125.7, 125.6, 118.6, 117.5, 110.4, 109.8, 96.1.

6-methyl-2-(p-tolyl)imidazo[1,2-a]pyridine[2] (**7p**)

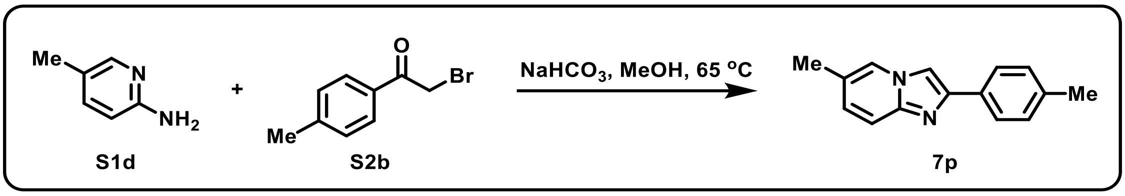

A three-neck round-bottom flask, under a nitrogen atmosphere, at room temperature, was charged with 5-methylpyridin-2-amine **S1d** (1.08 g, 10 mmol, 1 equiv.), $NaHCO_3$ (882

[9] ^{1}H and ^{13}C{^{1}H} data are in good agreement with the literature. See: W. Kim, H. Y. Kim, K. Oh, *J. Org. Chem.* **2021**, *86*, 15973-15991.

mg, 10.5 mmol, 1.05 equiv.) and MeOH (10 mL, 1M in relation to **S1d**). Then, a solution of 2-bromo-1-(*p*-tolyl)ethan-1-one **S2b** (2.24 g, 10.5 mmol, 1.05 equiv.) in MeOH (10 mL, 1M in relation to **S1d**) was slowly added. The resulting reaction mixture was heated to reflux (~ 65 ^{o}C) and stirred at this temperature until the complete consumption of **S1d** (as indicated by TLC analysis, which typically occurred in 24 h). Then, the reaction mixture was cooled down to room temperature, diluted in H_2O, extracted with AcOEt (3x), dried (Na_2SO_4) and concentrated under reduced pressure. Then, the solid formed is dissolved in a minimum amount of AcOEt and left in the fridge (- 20 ^{o}C) for 1h. The title product precipitates and is filtered off as a white solid: 1.68 g, 76%.

^{1}H NMR (600 MHz, $CDCl_3$) δ: 7.88 (d, *J* = 1.8 Hz, 1H), 7.83 (d, *J* = 8.1 Hz, 2H), 7.73 (s, 1H), 7.53 (d, *J* = 9.0 Hz, 1H), 7.23 (d, *J* = 8.1 Hz, 2H), 7.00 (dd, *J* = 9.0 Hz, *J* = 1.8 Hz, 1H), 2.38 (s, 3H), 2.31 (s, 3H).

^{13}C{^{1}H} NMR (150 MHz, $CDCl_3$) δ: 145.7, 144.8, 137.8, 131.1, 129.5, 127.9, 126.0, 123.4, 122.1, 116.9, 107.6, 21.4, 18.3.

.

1.2.2. Diazo compounds 8

A list of diazo compounds used in this work is presented, following their order of appearance in the manuscript (Figure S3). The following diazo compounds were synthesized as previously described in the literature: methyl 2-diazo-2-phenylacetate[10] (**8a**), isopropyl 2-diazo-2-phenylacetate[10] (**8b**), benzyl 2-diazo-2-phenylacetate[10] (**8c**), methyl 2-diazo-2-(2-methoxyphenyl)acetate[10] (**8d**), methyl 2-diazo-2-(3-methoxyphenyl)acetate[10] (**8e**), methyl 2-diazo-2-(4-methoxyphenyl)acetate[10] (**8f**), methyl 2-diazo-2-(4-fluorophenyl)acetate[11] (**8g**), methyl 2-(4-bromophenyl)-2-diazoacetate[11] (**8h**), methyl 2-(2-chlorophenyl)-2-diazoacetate[10] (**8i**), methyl 2-(3-chlorophenyl)-2-diazoacetate[10] (**8j**), methyl 4-(1-diazo-2-methoxy-2-oxoethyl)benzoate[10] (**8k**), methyl 2-diazo-2-(3,5-difluorophenyl)acetate[12] (**8l**), diethyl 2-diazomalonate (**8o**).[13]

[10] G. Cariello, R. D. C. Gallo, V. M. Deflon, R. A. Cormanich, I. D. Jurberg, *Chem. Commun.*, **2025**, *61*, 2044-2047.
[11] A. F. da Silva, M. A. S. Afonso, R. A. Cormanich, I. D. Jurberg, *Chem. Eur. J.*, **2020**, *26*, 5648-5653.
[12] M. L. Stivanin, A. A. G. Fernandes, A. F. da Silva, C. Y. Okada Jr, I. D. Jurberg, *Adv. Synth. Catal.*, **2020**, *362*, 1106-1111.
[13] G. A. Kadam, T. Singha, S. Rawat, D. P. Hari, *ACS Catal.* **2024**, *14*, 12225-12233.

Figure S3. Diazo compounds used in this work.

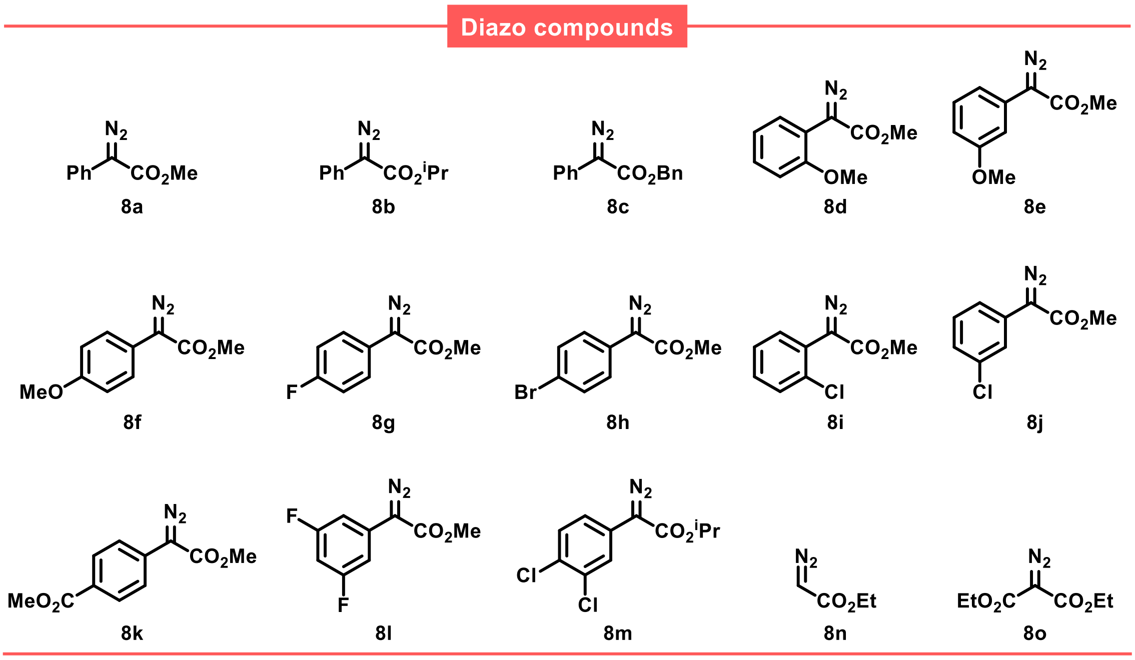

Diazo compound isopropyl 2-diazo-2-(3,4-dichlorophenyl)acetate (**8m**) is new. Diazo compound ethyl 2-diazoacetate (**8n**) (15% in toluene) was purchased from a commercial source and used directly without any treatment or additional purification step.

isopropyl 2-diazo-2-(3,4-dichlorophenyl)acetate (**8m**)

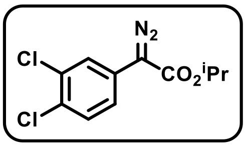

This diazo compound was prepared in 2 steps.

<u>*Step 1:*</u> A solution of 2-(3,4-dichlorophenyl)acetic acid (1.03 g, 5 mmol, 1 equiv.) in isopropanol (10 mL, 0.5 M in relation to the carboxylic acid) and H_2SO_4 (100 µL, 1.88 mmol, 0.375 equiv.) was heated under reflux (~ 82 °C) and stirred at this temperature until the starting carboxylic acid was completely consumed (reaction time ~ 24 h). Then, the reaction mixture was quenched with a saturated aqueous solution of $NaHCO_3$ and extracted with AcOEt (3x). The combined organic layers were dried (Na_2SO_4), filtered and concentrated under reduced pressure, yielding the intermediate ester isopropyl 2-(3,4-dichlorophenyl)acetate (**pre-8m**) in good purity, as a colorless oil: 1.09 g, 88%.

^{1}H NMR (600 MHz, $CDCl_3$) δ: 7.38 – 7.36 (m, 2H), 7.12 (dd, *J* = 8.4 Hz, *J* = 1.8 Hz, 1H), 5.01 (sept, *J* = 6.3 Hz, 1H), 3.52 (s, 2H), 1.23 (d, *J* = 6.3 Hz, 6H).

$^{13}C\{^1H\}$ NMR (150 MHz, $CDCl_3$) δ: 170.2, 134.5, 132.5, 131.3 (x2), 130.5, 128.8, 68.8, 40.7, 21.8.
IR (ATR, cm^{-1}): 2982, 1778, 1473, 1221, 1104, 1035.
HRMS (ESI+, Orbitrap), calcd. for $[C_{11}H_{12}Cl_2O_2 + H]^+$: 247.0287, found: 247.0287.

Step 2: Then, a solution of the previously prepared isopropyl 2-(3,4-dichlorophenyl)acetate **pre-8m** (741 mg, 3 mmol, 1 equiv.) and *p*-TsN_3 (709 mg, 3.6 mmol, 1.2 equiv.) in MeCN (10 mL, 0.33 M in relation to the ester) was cooled to 0 °C, and 1,8-diazabicyclo[5.4.0]undec-7-ene (DBU) (730 mg, 4.8 mmol, 1.6 equiv.) was added dropwise. The resulting reaction mixture was stirred at room temperature until complete consumption of the initial ester (reaction time ~ 24 h). Then, the reaction mixture was extracted with AcOEt (3x), and the combined organic layers were washed with a saturated aqueous solution of NH_4Cl, dried (Na_2SO_4), filtered and concentrated under reduced pressure. The residue was purified by flash column chromatography (SiO_2, Hex – 99:1 Hex:AcOEt) to afford the title compound as an orange solid: 552 mg, 67%.
^1H NMR (600 MHz, $CDCl_3$) δ: 7.65 (d, *J* = 2.4 Hz, 1H), 7.42 (d, *J* = 8.4 Hz, 1H), 7.29 (dd, *J* = 8.4 Hz, *J* = 2.4 Hz, 1H), 5.20 (sept, *J* = 6.3 Hz, 1H), 1.33 (d, *J* = 6.3 Hz, 6H).
$^{13}C\{^1H\}$ NMR (150 MHz, $CDCl_3$) (1C could not be unambiguously assigned) δ: 164.1, 133.4, 130.8, 129.4, 126.5, 125.5, 122.8, 69.4, 22.2.
IR (ATR, cm^{-1}): 2969, 2078, 1699, 1478, 1338, 1172, 1105, 1022.
M.P.: 62 – 64 °C.
HRMS (ESI+, Orbitrap), calcd. for $[C_{11}H_{10}Cl_2N_2O_2 + H]^+$: 273.0192, found: 273.0192.

1.2.3. Hypervalent Iodine Compound 12

The hypervalent iodine compound diethyl 2-(phenyl-λ^3-iodanylidene)malonate (**12**) was synthesized as previously described in the literature.[14]

[14] S. R. Goudreau, D. Marcoux, A. B. Charette, *J. Org. Chem.* **2009**, *74*, 470-473.

Figure S4. Hypervalent iodine compound used in this work.

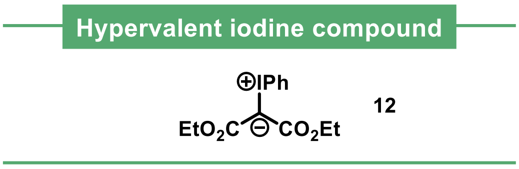

1.2.4. Formal C-H Insertion of *aza*-Arenes 7 onto Aryldiazoacetates 8

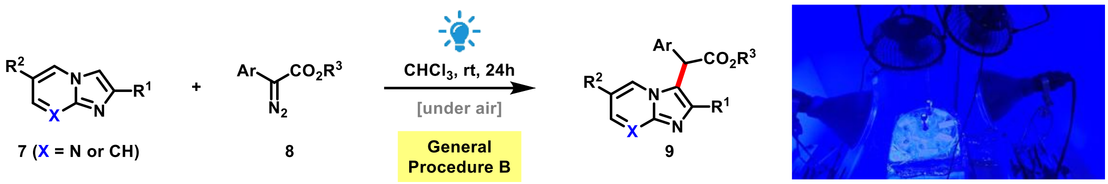

General Procedure B: *Visible light-mediated C-H insertion of imidazo[1,2-a]pyrimidines or imidazo[1,2-a]pyridines* **7** *onto aryldiazoacetates* **8**

Under air, at room temperature, a 4-mL glass vial was charged with aryldiazoacetate **8** (0.6 or 0.8 mmol, 3 or 4 equiv.), $CHCl_3$ (2 mL, 0.1 M in relation to **7**) and imidazo[1,2-a]pyrimidine or imidazo[1,2-a]pyridine **7** (0.2 mmol, 1 equiv.). The reaction mixture was irradiated by 2 blue LED lamps (15W each, λ_{max} = 452 nm,[12] distance ~10 cm) while stirring at room temperature (*ca.* 30 °C) until the complete consumption of the starting heterocycle **7** (as indicated by TLC analysis, which typically occurred in 24 h). Then, the reaction mixture was concentrated under reduced pressure and purified by flash column chromatography to afford the C3-alkylated compound **9** in the stated yield.

methyl 2-phenyl-2-(2-phenylimidazo[1,2-a]pyrimidin-3-yl)acetate (**9aa**)

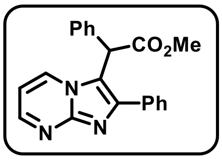

The **General Procedure B** was employed with 2-phenylimidazo[1,2-a]pyrimidine **7a** (39 mg, 0.2 mmol), methyl 2-diazo-2-phenylacetate **8a** (106 mg, 0.6 mmol) and $CHCl_3$ (2 mL). Purification by flash column chromatography (SiO_2 pre-treated with 60:1 Hex:Et_3N, then gradient: Hex – 9:1 Hex:AcOEt – 8:2 Hex:AcOEt – 7:3 Hex:AcOEt – 6:4 Hex:AcOEt) afforded the title product as a yellow solid: 56 mg, 81%.

This reaction was also performed in a 2-mmol scale, according to **General Procedure B** employing 2-phenylimidazo[1,2-a]pyrimidine **7a** (390 mg, 2 mmol), methyl 2-diazo-2-phenylacetate **8a** (1.06 g, 6 mmol) and $CHCl_3$ (20 mL). Purification by flash column chromatography (SiO_2 pre-treated with 60:1 Hex:Et_3N, then gradient: Hex – 9:1 Hex:AcOEt – 8:2 Hex:AcOEt – 7:3 Hex:AcOEt – 6:4 Hex:AcOEt) afforded the title product as a yellow solid: 542 mg, 79%.

^{1}H NMR (500 MHz, $CDCl_3$) δ: 8.53 (dd, *J* = 4.0 Hz, *J* = 2.0 Hz, 1H), 8.30 (dd, *J* = 7.0 Hz, *J* = 2.0 Hz, 1H), 7.85 – 7.83 (m, 2H), 7.51 – 7.48 (m, 2H), 7.44 – 7.40 (m, 1H), 7.31 – 7.28 (m, 3H), 7.04 – 7.02 (m, 2H), 6.68 (dd, *J* = 7.0 Hz, *J* = 4.0 Hz, 1H), 5.94 (s, 1H), 3.84 (s, 3H).
^{13}C{^{1}H} NMR (125 MHz, $CDCl_3$) δ: 171.4, 150.3, 148.8, 147.6, 134.2, 133.9, 133.5, 129.3, 129.2, 128.9, 128.7, 128.0, 127.2, 114.3, 108.2, 53.0, 47.3.
M.P.: 171 – 173 °C.
IR (ATR, cm^{-1}): 3031, 2953, 1733, 1619, 1501, 1496, 1430, 1305, 1195, 1153.
HRMS (ESI+, Orbitrap), calcd. for $[C_{21}H_{17}N_3O_2 + H]^+$: 344.1394, found: 344.1389.

Structure defined by single crystal X-ray diffraction (SC-XRD) of **9aa** (CCDC 2427912)

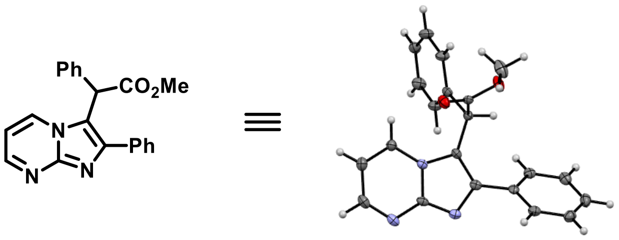

(ORTEP representation with ellipsoids showing 50% of probability)

Crystal data

$C_{21}H_{17}N_3O_2$	*Z* = 4
M_r = 343.38	*F*(000) = 720
Triclinic, $P\bar{1}$	D_x = 1.363 mg m^{-3}
a = 9.6988 (8) Å	Mo *K*α radiation, λ = 0.71073 Å
b = 10.5794 (8) Å	Cell parameters from 4226 reflections
c = 17.9789 (15) Å	θ = 4.8–55.8°
α = 87.716 (2)°	μ = 0.09 mm^{-1}

β = 81.254 (2)°	T = 120 K
γ = 66.599 (2)°	Irregular, colorless
V = 1672.9 (2) Å^3	0.30 × 0.15 × 0.15 mm

Data collection

Bruker APEX CCD detector diffractometer	7969 independent reflections
Radiation source: fine-focus sealed tube	5819 reflections with $I > 2\sigma(I)$
Detector resolution: 8.3333 pixels mm^{-1}	R_{int} = 0.034
phi and ω scans	θ_{max} = 27.9°, θ_{min} = 2.1°
Absorption correction: multi-scan *SADABS* (Bruker, 2010)	h = -12→12
T_{min} = 0.711, T_{max} = 0.746	k = -13→13
20801 measured reflections	l = -23→23

Refinement

Refinement on F^2	Primary atom site location: structure-invariant direct methods
Least-squares matrix: full	Hydrogen site location: inferred from neighbouring sites
$R[F^2 > 2\sigma(F^2)]$ = 0.044	H-atom parameters constrained
$wR(F^2)$ = 0.101	$w = 1/[\sigma^2(F_o^2) + (0.0385P)^2 + 0.5161P]$ where $P = (F_o^2 + 2F_c^2)/3$
S = 1.01	$(\Delta/\sigma)_{max}$ < 0.001
7969 reflections	$\Delta\rangle_{max}$ = 0.31 e Å^{-3}
471 parameters	$\Delta\rangle_{min}$ = -0.25 e Å^{-3}
0 restraints	

methyl 2-phenyl-2-(2-(p-tolyl)imidazo[1,2-a]pyrimidin-3-yl)acetate (**9ba**)

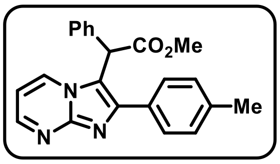

The **General Procedure B** was employed with 2-(*p*-tolyl)imidazo[1,2-a]pyrimidine **7b** (42 mg, 0.2 mmol), methyl 2-diazo-2-phenylacetate **8a** (106 mg, 0.6 mmol) and $CHCl_3$ (2 mL). Purification by flash column chromatography (SiO_2 pre-treated with 60:1 Hex:Et_3N, then gradient: Hex – 9:1 Hex:AcOEt – 7:3 Hex:AcOEt – 1:1 Hex:AcOEt) afforded the title product as a yellow solid: 59 mg, 83%.

^{1}H NMR (250 MHz, $CDCl_3$) δ: 8.56 (dd, *J* = 4.1 Hz, *J* = 1.9 Hz, 1H), 8.34 (dd, *J* = 6.9, *J* = 1.9 Hz, 1H), 7.79 (d, *J* = 8.0 Hz, 2H), 7.39 – 7.31 (m, 5H), 7.11 – 7.03 (m, 2H), 6.72 (dd, *J* = 6.9 Hz, *J* = 4.1 Hz, 1H), 5.98 (s, 1H), 3.88 (s, 3H), 2.45 (s, 3H).
$^{13}C\{^1H\}$ NMR (62.5 MHz, $CDCl_3$) δ: 171.3, 150.1, 148.7, 147.7, 138.6, 134.0, 133.9, 130.5, 129.6, 129.2, 129.0, 128.0, 127.2, 114.0, 108.1, 52.9, 47.2, 21.4.
IR (ATR, cm^{-1}): 3029, 2954, 1726, 1497, 1329, 1157, 837, 771, 711, 515.
M.P.: 194 – 196 °C.
HRMS (ESI+, Orbitrap), calcd. for $[C_{22}H_{19}N_3O_2 + H]^+$: 358.1550, found: 358.1546.

methyl 2-(2-(2-methoxyphenyl)imidazo[1,2-a]pyrimidin-3-yl)-2-phenylacetate (**9ca**)

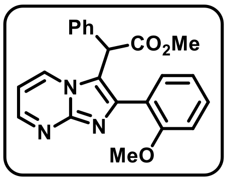

The **General Procedure B** was employed with 2-(2-methoxyphenyl)imidazo[1,2-*a*]pyrimidine **7c** (45 mg, 0.2 mmol), methyl 2-diazo-2-phenylacetate **8a** (106 mg, 0.6 mmol) and $CHCl_3$ (2 mL). Purification by flash column chromatography (SiO_2 pre-treated with 60:1 Hex:Et_3N, then gradient: Hex – 9:1 Hex:AcOEt – 7:3 Hex:AcOEt – 1:1 Hex:AcOEt) afforded the title product as a light yellow solid: 56 mg, 75%.
^{1}H NMR (500 MHz, $CDCl_3$) δ: 8.51 (dd, *J* = 4.0 Hz, *J* = 2.0 Hz, 1H), 8.22 (dd, *J* = 6.5 Hz, *J* = 2.0 Hz, 1H), 7.78 (dd, *J* = 7.5 Hz, *J* = 2.0 Hz, 1H), 7.42 (td, *J* = 8.5 Hz, *J* = 2.0 Hz, 1H), 7.33 – 7.28 (m, 3H), 7.13 (t, *J* = 7.5 Hz, 1H), 7.08 – 7.07 (m, 2H), 7.00 (d, *J* = 8.5 Hz, 1H), 6.66 (dd, *J* = 7.0 Hz, *J* = 4.0 Hz, 1H), 5.62 (s, 1H), 3.82 (s, 3H), 3.66 (s, 3H).
$^{13}C\{^1H\}$ NMR (125 MHz, $CDCl_3$) δ: 171.6, 156.6, 149.5, 148.7, 144.1, 134.4, 133.6, 132.7, 130.2, 129.1, 127.7, 127.3, 122.5, 121.2, 116.8, 111.0, 107.9, 55.3, 52.5, 48.0.
M.P.: 174 – 176 °C.
IR (ATR, cm^{-1}): 3005, 2948, 1735, 1616, 1498, 1433, 1244, 1221, 1197, 1155.
HRMS (ESI+, Orbitrap), calcd. for $[C_{22}H_{19}N_3O_3 + H]^+$: 374.1499, found: 374.1490.

methyl 2-(2-(3-methoxyphenyl)imidazo[1,2-a]pyrimidin-3-yl)-2-phenylacetate (**9da**)

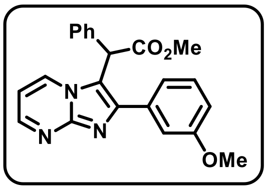

The **General Procedure B** was employed with 2-(3-methoxyphenyl)imidazo[1,2-*a*]pyrimidine **7d** (45 mg, 0.2 mmol) methyl 2-diazo-2-phenylacetate **8a** (106 mg, 0.6 mmol) and $CHCl_3$ (2 mL). Purification by flash column chromatography (SiO_2 pre-

treated with 60:1 Hex:Et_3N, then gradient: Hex – 9:1 Hex:AcOEt – 7:3 Hex:AcOEt – 1:1 Hex:AcOEt) afforded the title product as a yellow solid: 49 mg, 66%.

^{1}H NMR (600 MHz CDCl$_3$) δ: 8.54 (dd, *J* = 4.2 Hz, *J* = 1.8 Hz, 1H), 8.31 (dd, *J* = 6.9 Hz, *J* = 2.1 Hz, 1H), 7.46 (d, *J* = 1.8 Hz, 1H), 7.40 – 7.38 (m, 2H), 7.32 – 7.28 (m, 3H), 7.04 – 7.02 (m, 2H), 6.99 – 6.96 (m, 1H), 6.68 (dd, *J* = 6.9 Hz, *J* = 4.2 Hz, 1H), 5.98 (s, 1H), 3.88 (s, 3H), 3.85 (s, 3H).

^{13}C{^{1}H} NMR (150 MHz, CDCl$_3$) δ: 171.4, 160.0, 150.3, 148.7, 147.6, 134.8, 134.2, 133.9, 129.9, 129.3, 128.1, 127.2, 121.4, 115.3, 114.4, 114.1, 108.2, 55.5, 53.0, 47.3.

M.P.: 140 – 142 °C

IR (ATR, cm^{-1}): 2953, 1731, 1605, 1498, 1452, 1433, 1246, 1153, 1034.

HRMS (ESI+, Orbitrap), calcd. for [$C_{22}H_{19}N_3O_3$ + H]$^+$: 374.1499, found: 374.1492.

methyl 2-(2-(4-methoxyphenyl)imidazo[1,2-a]pyrimidin-3-yl)-2-phenylacetate (**9ea**)

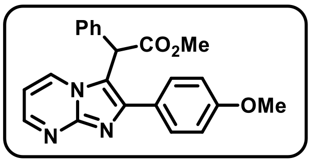

The **General Procedure B** was employed with 2-(4-methoxyphenyl)imidazo[1,2-*a*]pyrimidine **7e** (45 mg, 0.2 mmol), methyl 2-diazo-2-phenylacetate **8a** (106 mg, 0.6 mmol) and $CHCl_3$ (2 mL). Purification by flash column chromatography (SiO_2 pre-treated with 60:1 Hex:Et_3N, then gradient: Hex – 9:1 Hex:AcOEt – 7:3 Hex:AcOEt – 1:1 Hex:AcOEt – 4:6 Hex:AcOEt) afforded the title product as a yellow solid: 54 mg, 72%.

^{1}H NMR (250 MHz, CDCl$_3$) δ: 8.51 (dd, *J* = 4.0 Hz, *J* = 2.0 Hz, 1H), 8.28 (dd, *J* = 7.0 Hz, *J* = 2.0 Hz, 1H), 7.79 (d, *J* = 8.8 Hz, 2H), 7.35 – 7.28 (m, 3H), 7.05 (d, *J* = 8.8 Hz, 4H), 6.66 (dd, *J* = 7.0 Hz, *J* = 4.0 Hz, 1H), 5.91 (s, 1H), 3.85 (s, 3H), 3.83 (s, 3H).

^{13}C{^{1}H} NMR (62.5 MHz, CDCl$_3$) δ: 171.4, 160.1, 150.0, 148.7, 147.5, 134.0, 133.9, 130.5, 129.3, 128.0, 127.2, 125.9, 114.4, 113.7, 108.1, 55.5, 53.0, 47.3.

IR (ATR, cm^{-1}): 2957, 2838, 1725, 1616, 1497, 1244, 1156, 1029.

M.P.: 181 – 183 °C.

HRMS (ESI+, Orbitrap), calcd. for [$C_{22}H_{19}N_3O_3$ + H]$^+$: 374.1499, found: 374.1496.

methyl 2-(2-(4-fluorophenyl)imidazo[1,2-a]pyrimidin-3-yl)-2-phenylacetate (**9fa**)

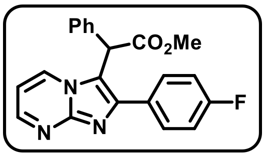

The **General Procedure B** was employed with 2-(4-fluorophenyl)imidazo[1,2-*a*]pyrimidine **7f** (43 mg, 0.2 mmol), methyl 2-diazo-2-phenylacetate **8a** (106 mg, 0.6 mmol) and $CHCl_3$ (2 mL). Purification by flash column chromatography (SiO_2 pre-treated with 60:1 Hex:Et_3N, then gradient: Hex – 9:1 Hex:AcOEt – 8:2 Hex:AcOEt – 7:3 Hex:AcOEt) afforded the title product as a white solid: 54 mg, 75%.

^1H NMR (500 MHz, $CDCl_3$) δ 8.53 (dd, *J* = 4.0 Hz, *J* = 2.0 Hz, 1H), 8.29 (dd, *J* = 7.0 Hz, *J* = 2.0 Hz, 1H), 7.82 (dd, *J* = 8.8 Hz, *J* = 5.5 Hz, 2H), 7.32 – 7.28 (m, 3H), 7.18 (t, *J* = 8.8 Hz, 2H), 7.03 – 7.01 (m, 2H), 6.69 (dd, *J* = 7.0 Hz, *J* = 4.0 Hz, 1H), 5.86 (s, 1H), 3.83 (s, 3H).

$^{13}C\{^1H\}$ NMR (125 MHz, $CDCl_3$) δ: 171.2, 163.1 (d, *J* = 247.5 Hz), 150.4, 148.7, 146.6, 134.2, 133.7, 131.0 (d, *J* = 7.5 Hz), 129.5 (d, *J* = 2.5 Hz), 129.3, 128.1, 127.2, 115.9 (d, *J* = 22.5 Hz), 114.2, 108.3, 53.0, 47.2.

$^{19}F\{^1H\}$ NMR (470 MHz, $CDCl_3$) δ: -112.9.

IR (ATR, cm^{-1}): 3054, 3004, 2954, 1731, 1492, 1220, 1155.

M.P.: 148 – 150 °C.

HRMS (ESI+, Orbitrap), calcd. for $[C_{21}H_{16}FN_3O_2 + H]^+$: 362.1299, found: 362.1290.

methyl 2-(2-(4-chlorophenyl)imidazo[1,2-a]pyrimidin-3-yl)-2-phenylacetate (**9ga**)

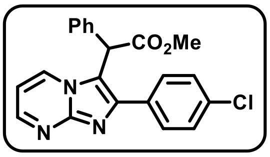

The **General Procedure B** was employed with 2-(4-chlorophenyl)imidazo[1,2-*a*]pyrimidine **7g** (46 mg, 0.2 mmol), methyl 2-diazo-2-phenylacetate **8a** (106 mg, 0.6 mmol) and $CHCl_3$ (2 mL). Purification by flash column chromatography (SiO_2 pre-treated with 60:1 Hex:Et_3N, then gradient: Hex – 9:1 Hex:AcOEt – 8:2 Hex:AcOEt – 7:3 Hex:AcOEt – 6:4 Hex:AcOEt) afforded the title product as a yellow solid: 58 mg, 77%.

^1H NMR (250 MHz, $CDCl_3$) δ: 8.55 (dd, *J* = 4.0 Hz, *J* = 2.0 Hz, 1H), 8.30 (dd, *J* = 7.0 Hz, *J* = 2.0 Hz, 1H), 7.79 (d, *J* = 8.5 Hz, 2H), 7.47 (d, *J* = 8.5 Hz, 2H), 7.34 – 7.28 (m, 3H), 7.04 – 7.00 (m, 2H), 6.70 (dd, *J* = 7.0 Hz, *J* = 4.0 Hz, 1H), 5.87 (s, 1H), 3.85 (s, 3H).

$^{13}C\{^1H\}$ NMR (62.5 MHz, $CDCl_3$) δ: 171.2, 150.6, 148.8, 146.5, 134.9, 134.3, 133.7, 132.0, 130.5, 129.4, 129.2, 128.2, 127.2, 114.5, 108.4, 53.1, 47.3.

IR (ATR, cm^{-1}): 3060, 3048, 2952, 1727, 1496, 1153.

M.P.: 201 – 203 °C.

HRMS (ESI+, Orbitrap), calcd. for [$C_{21}H_{16}ClN_3O_2$ + H]$^+$: 378.1004, found: 378.0996.

methyl 2-(2-(4-bromophenyl)imidazo[1,2-a]pyrimidin-3-yl)-2-phenylacetate (**9ha**)

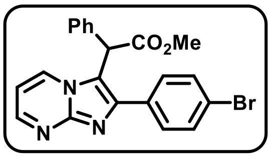

The **General Procedure B** was employed with 2-(4-bromophenyl)imidazo[1,2-*a*]pyrimidine **7h** (55 mg, 0.2 mmol), methyl 2-diazo-2-phenylacetate **8a** (106 mg, 0.6 mmol) and $CHCl_3$ (2 mL). Purification by flash column chromatography (SiO_2 pre-treated with 60:1 Hex:Et_3N, then gradient: Hex – 9:1 Hex:AcOEt – 8:2 Hex:AcOEt – 6:4 Hex:AcOEt – 4:6 Hex:AcOEt) afforded the title product as a yellow solid: 43 mg, 51%.

^{1}H NMR (500 MHz, $CDCl_3$) δ: 8.55 (dd, *J* = 4.0 Hz, *J* = 2.0 Hz, 1H), 8.31 (dd, *J* = 7.0 Hz, *J* = 2.0 Hz, 1H), 7.72 (d, *J* = 8.5 Hz, 2H), 7.63 (d, *J* = 8.5 Hz, 2H), 7.34 – 7.29 (m, 3H), 7.03 – 7.01 (m, 2H), 6.70 (dd, *J* = 7.0 Hz, *J* = 4.0 Hz, 1H), 5.86 (s, 1H), 3.84 (s, 3H).

^{13}C{^{1}H} NMR (125 MHz, $CDCl_3$) δ: 171.2, 150.6, 148.8, 146.5, 134.3, 133.6, 132.4, 132.1, 130.7, 129.4, 128.2, 127.2, 123.2, 114.5, 108.4, 53.1, 47.3.

IR (ATR, cm^{-1}): 3048, 2952, 2844, 1727, 1496, 1153.

M.P.: 210 – 212 °C.

HRMS (ESI+, Orbitrap), calcd. for [$C_{21}H_{16}BrN_3O_2$ + H]$^+$: 422.0499, found: 422.0492.

methyl 2-(2-([1,1'-biphenyl]-4-yl)imidazo[1,2-a]pyrimidin-3-yl)-2-phenylacetate (**9ia**)

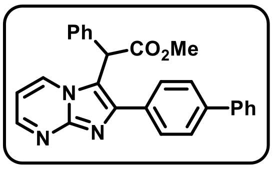

The **General Procedure B** was employed with 2-([1,1'-biphenyl]-4-yl)imidazo[1,2-a]pyrimidine **7i** (54 mg, 0.2 mmol), methyl 2-diazo-2-phenylacetate **8a** (141 mg, 0.8 mmol) and $CHCl_3$ (2 mL). Purification by flash column chromatography (SiO_2 pre-treated with 60:1 Hex:Et_3N, then gradient: Hex – 9:1 Hex:AcOEt – 8:2 Hex:AcOEt – 6:4 Hex:AcOEt – 1:1 Hex:AcOEt) afforded the title product as a yellow solid: 58 mg, 69%.

^{1}H NMR (600 MHz, $CDCl_3$) δ: 8.55 (dd, *J* = 3.9 Hz, *J* = 2.1 Hz, 1H), 8.32 (dd, *J* = 6.9 Hz, *J* = 2.1 Hz, 1H), 7.94 (d, *J* = 8.1 Hz, 2H), 7.74 (d, *J* = 8.1 Hz, 2H), 7.67 – 7.65 (m, 2H), 7.47 (t, *J* = 7.8 Hz, 2H), 7.37 (tt, *J* = 7.2 Hz, *J* = 1.5 Hz, 1H), 7.34 – 7.30 (m, 3H), 7.07 – 7.05 (m, 2H), 6.69 (dd, *J* = 6.9 Hz, *J* = 3.9 Hz, 1H), 6.00 (s, 1H), 3.87 (s, 3H).

$^{13}C\{^1H\}$ NMR (150 MHz, CDCl$_3$) δ: 171.4, 150.3, 148.9, 147.3, 141.5, 140.7, 134.2, 133.9, 132.5, 129.6, 129.4, 129.0, 128.1, 127.7, 127.6, 127.3 (x2), 114.4, 108.2, 53.0, 47.4.

IR (ATR, cm^{-1}): 2953, 1731, 1493, 1451, 1195, 1184, 1079, 1043.

M.P.: 224 – 226 °C.

HRMS (ESI+, Orbitrap), calcd. for [$C_{27}H_{21}N_3O_2$ + H]$^+$: 420.1707, found: 420.1699.

methyl 2-(2-(naphthalen-2-yl)imidazo[1,2-a]pyrimidin-3-yl)-2-phenylacetate (**9ja**)

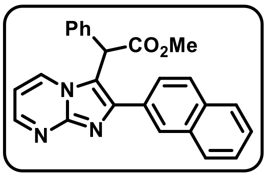

The **General Procedure B** was employed with 2-(naphthalen-2-yl)imidazo[1,2-*a*]pyrimidine **7j** (49 mg, 0.2 mmol), methyl 2-diazo-2-phenylacetate **8a** (141 mg, 0.8 mmol) and CHCl$_3$ (2 mL). Purification by flash column chromatography (SiO$_2$ pre-treated with 60:1 Hex:Et$_3$N, then gradient: Hex – 9:1 Hex:AcOEt – 8:2 Hex:AcOEt – 6:4 Hex:AcOEt) afforded the title product as a yellow solid: 55 mg, 70%.

^1H NMR (500 MHz, CDCl$_3$) δ: 8.56 (dd, *J* = 4.0 Hz, *J* = 2.0 Hz, 1H), 8.35 (dd, *J* = 7.0 Hz, *J* = 2.0 Hz, 1H), 8.33 (d, *J* = 1.5 Hz, 1H), 8.01 (dd, *J* = 8.5 Hz, *J* = 1.5 Hz, 1H), 7.98 (d, *J* = 8.5 Hz, 1H), 7.94 – 7.87 (m, 2H), 7.54-7.51 (m, 2H), 7.34 – 7.28 (m, 3H), 7.08 - 7.06 (m, 2H), 6.71 (dd, *J* = 7.0 Hz, *J* = 4.0 Hz, 1H), 6.05 (s, 1H), 3.87 (s, 3H).

$^{13}C\{^1H\}$ NMR (125 MHz, CDCl$_3$) δ: 171.4, 150.4, 148.9, 147.6, 134.2, 133.9, 133.5, 133.3, 130.9, 129.4, 128.6 (x3), 128.1, 127.9, 127.2, 126.8, 126.7, 126.5, 114.7, 108.3, 53.0, 47.4.

IR (ATR, cm^{-1}): 3006, 2956, 1731, 1494, 1326, 1149.

M.P.: 211 – 213 °C

HRMS (ESI+, Orbitrap), calcd. for [$C_{25}H_{19}N_3O_2$ + H]$^+$: 394.1550, found: 394.1542.

methyl 2-(2-(tert-butyl)imidazo[1,2-a]pyrimidin-3-yl)-2-phenylacetate (**9ka**)

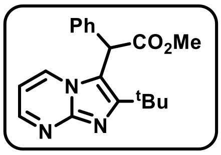

The **General Procedure B** was employed with 2-(*tert*-butyl)imidazo[1,2-*a*]pyrimidine **7k** (35 mg, 0.2 mmol), methyl 2-diazo-2-phenylacetate **8a** (106 mg, 0.6 mmol) and CHCl$_3$ (2 mL). Purification by flash column chromatography (SiO$_2$ pre-treated with 60:1 Hex:Et$_3$N, then gradient: Hex – 9:1 Hex:AcOEt – 8:2 Hex:AcOEt – 6:4 Hex:AcOEt – 1:1 Hex:AcOEt) afforded the title product as a yellow solid: 45 mg, 70%.

^{1}H NMR (500 MHz, CDCl$_3$) δ: 8.42 (dd, *J* = 4.0 Hz, *J* = 2.0 Hz, 1H), 8.13 (dd, *J* = 7.0 Hz, *J* = 2.0 Hz, 1H), 7.34 – 7.28 (m, 3H), 7.03 – 7.01 (m, 2H), 6.56 (dd, *J* = 7.0 Hz, *J* = 4.0 Hz, 1H), 6.06 (s, 1H), 3.81 (s, 3H), 1.56 (s, 9H).
^{13}C{^{1}H} NMR (125 MHz, CDCl$_3$) δ: 171.5, 156.4, 149.0, 147.4, 134.1, 133.8, 129.3, 128.0, 127.0, 112.8, 107.6, 52.8, 47.7, 33.9, 31.2.
IR (ATR, cm^{-1}): 2965, 2921, 1729, 1492, 1160, 1000.
M.P.: 156 – 158 °C.
HRMS (ESI+, Orbitrap), calcd. for [$C_{19}H_{21}N_3O_2$ + H]$^+$: 324.1707, found: 324.1698.

isopropyl 2-phenyl-2-(2-phenylimidazo[1,2-a]pyrimidin-3-yl)acetate (**9ab**)

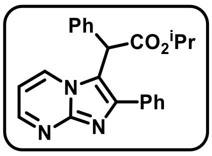

The **General Procedure B** was employed with 2-phenylimidazo[1,2-a]pyrimidine **7a** (39 mg, 0.2 mmol), isopropyl 2-diazo-2-phenylacetate **8b** (122 mg, 0.6 mmol) and $CHCl_3$ (2 mL). Purification by flash column chromatography (SiO_2 pre-treated with 60:1 Hex:Et_3N, then gradient: Hex – 9:1 Hex:AcOEt – 8:2 Hex:AcOEt – 6:4 Hex:AcOEt) afforded the title product as a brown solid: 62 mg, 84%.
^{1}H NMR (600 MHz, CDCl$_3$) δ: 8.53 (dd, *J* = 4.2 Hz, *J* = 1.8 Hz, 1H), 8.33 (dd, *J* = 7.0 Hz, *J* = 2.0 Hz, 1H), 7.88 – 7.86 (m, 2H), 7.52 – 7.48 (m, 2H), 7.44 – 7.41 (m, 1H), 7.32 – 7.28 (m, 3H), 7.05 – 7.03 (m, 2H), 6.67 (dd, *J* = 7.0 Hz, *J* = 4.2 Hz, 1H), 5.85 (s, 1H), 5.17 (sept, *J* = 6.3 Hz, 1H), 1.34 (d, *J* = 6.3 Hz, 3H), 1.28 (d, *J* = 6.3 Hz, 3H).
^{13}C{^{1}H} NMR (150 MHz, CDCl$_3$) δ: 170.5, 150.3, 148.8, 147.7, 134.4, 134.2, 133.6, 129.3 (x2), 128.8, 128.7, 127.9, 127.2, 114.7, 108.0, 70.2, 47.7, 22.0, 21.9.
M.P.: 46 – 48 °C.
IR (ATR, cm^{-1}): 2979, 1726, 1616, 1524, 1498, 1348, 1304, 1216, 1190, 1154, 1101.
HRMS (ESI+, Orbitrap), calcd. for [$C_{23}H_{21}N_3O_2$ + H$^+$]: 372.1707, found: 372.1701.

benzyl 2-phenyl-2-(2-phenylimidazo[1,2-a]pyrimidin-3-yl)acetate (**9ac**)

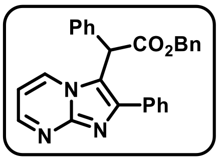

The **General Procedure B** was employed with 2-phenylimidazo[1,2-*a*]pyrimidine **7a** (39 mg, 0.2 mmol), benzyl 2-diazo-2-phenylacetate **8c** (151 mg, 0.6 mmol) and $CHCl_3$ (2 mL). Purification by flash column chromatography (SiO_2 pre-treated with 60:1 Hex:Et_3N, then

gradient: Hex – 9:1 Hex:AcOEt – 8:2 Hex:AcOEt – 6:4 Hex:AcOEt – 1:1 Hex:AcOEt) afforded the title product as a yellow solid: 69 mg, 82%.

^{1}H NMR (500 MHz, $CDCl_3$) δ: 8.43 (dd, *J* = 4.0 Hz, *J* = 2.0 Hz, 1H), 8.14 (dd, *J* = 7.0 Hz, *J* = 2.0 Hz, 1H), 7.78 – 7.76 (m, 2H), 7.41 – 7.38 (m, 2H), 7.36 – 7.32 (m, 1H), 7.27 – 7.24 (m, 3H), 7.23 – 7.19 (m, 5H), 6.95 – 6.92 (m, 2H), 6.52 (dd, *J* = 7.0 Hz, *J* = 4.0 Hz, 1H), 5.88 (s, 1H), 5.21 (d, *J* = 12.0 Hz, 1H), 5.17 (d, *J* = 12.0 Hz, 1H).

$^{13}C\{^1H\}$ NMR (125 MHz, $CDCl_3$) δ: 170.6, 150.2, 148.7, 147.7, 135.0, 134.0, 133.8, 133.4, 129.2 (x2), 128.8 (x3), 128.7, 128.5, 128.0, 127.2, 114.3, 108.0, 67.8, 47.4.

IR (ATR, cm^{-1}): 3065, 1740, 1496, 1142.

M.P.: 116 – 118 °C.

HRMS (ESI+, Orbitrap), calcd. for [$C_{27}H_{21}N_3O_2$ + H]$^+$: 420.1707, found: 420.1703.

methyl 2-(2-methoxyphenyl)-2-(2-phenylimidazo[1,2-a]pyrimidin-3-yl)acetate (**9ad**)

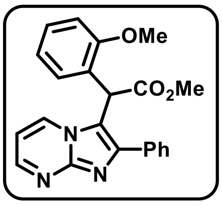

The **General Procedure B** was employed with 2-phenylimidazo[1,2-*a*]pyrimidine **7a** (39 mg, 0.2 mmol), methyl 2-diazo-2-(2-methoxyphenyl)acetate **8d** (124 mg, 0.6 mmol) and $CHCl_3$ (2 mL). Purification by flash column chromatography (SiO_2 pre-treated with 60:1 Hex:Et_3N, then gradient: Hex – 9:1 Hex:AcOEt – 8:2 Hex:AcOEt – 6:4 Hex:AcOEt – 4:6 Hex:AcOEt – 3:7 Hex:AcOEt) afforded the title product as a yellow solid: 57 mg, 76%.

^{1}H NMR (250 MHz, $CDCl_3$) δ: 8.60 (dd, *J* = 6.9 Hz, *J* = 2.0 Hz, 1H), 8.56 (dd, *J* = 4.3 Hz, *J* = 2.0 Hz, 1H), 7.82 – 7.79 (m, 2H), 7.50 – 7.35 (m, 3H), 7.33 – 7.26 (m, 1H), 6.93 (dd, *J* = 8.3 Hz, *J* = 1.2 Hz, 1H), 6.83 (td, *J* = 7.4 Hz, *J* = 1.2 Hz, 1H), 6.77 (dd, *J* = 6.9 Hz, *J* = 4.3 Hz, 1H), 6.68 (dt, *J* = 7.4 Hz, *J* = 1.2 Hz, 1H), 5.89 (s, 1H), 3.76 (s, 3H), 3.71 (s, 3H).

$^{13}C\{^1H\}$ NMR (62.5 MHz, $CDCl_3$) δ: 171.0, 157.2, 150.0, 148.6, 147.7, 134.2, 133.8, 129.6, 129.2, 128.7, 128.5, 128.0, 122.9, 120.8, 113.5, 111.4, 108.3, 55.7, 52.7, 43.5.

IR (ATR, cm^{-1}): 3083, 2952, 1741, 1490, 1245, 1152.

M.P.: 76 – 78 °C.

HRMS (ESI+, Orbitrap), calcd. for [$C_{22}H_{19}N_3O_3$ + H]$^+$: 374.1499, found: 374.1495.

methyl 2-(3-methoxyphenyl)-2-(2-phenylimidazo[1,2-a]pyrimidin-3-yl)acetate (**9ae**)

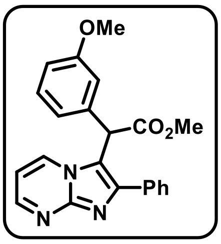

The **General Procedure B** was employed with 2-phenylimidazo[1,2-*a*]pyrimidine **7a** (39 mg, 0.2 mmol), methyl 2-diazo-2-(3-methoxyphenyl)acetate **8e** (124 mg, 0.6 mmol) and $CHCl_3$ (2 mL). Purification by flash column chromatography (SiO_2 pre-treated with 60:1 Hex:Et_3N, then gradient: Hex – 9:1 Hex:AcOEt – 8:2 Hex:AcOEt – 6:4 Hex:AcOEt – 1:1 Hex:AcOEt) afforded the title product as a yellow solid: 54 mg, 72%.

^{1}H NMR (300 MHz, $CDCl_3$) δ: 8.55 (dd, *J* = 4.0 Hz, *J* = 2.0 Hz, 1H), 8.34 (dd, *J* = 6.9 Hz, *J* = 2.0 Hz, 1H), 7.86-7.82 (m, 2H), 7.53 – 7.40 (m, 3H), 7.23 (t, *J* = 8.0 Hz, 1H), 6.82 (dd, *J* = 8.0 Hz, *J* = 2.0 Hz, 1H), 6.70 (dd, *J* = 6.9 Hz, *J* = 4.0 Hz, 1H), 6.63 - 6.59 (m, 2H), 5.90 (s, 1H), 3.83 (s, 3H), 3.71 (s, 3H).

^{13}C{^{1}H} NMR (75 MHz, $CDCl_3$) δ: 171.2, 160.3, 150.3, 148.8, 147.6, 135.5, 134.2, 133.5, 130.3, 129.2, 128.9, 128.7, 119.4, 114.3, 113.7, 112.8, 108.2, 55.4, 53.0, 47.2.

IR (ATR, cm^{-1}): 2951, 2837, 1734, 1601, 1492, 1432, 1148, 1047.

M.P.: 47 – 49 °C.

HRMS (ESI+, Orbitrap), calcd. for [$C_{22}H_{19}N_3O_3$ + H]$^+$: 374.1499, found: 374.1492.

methyl 2-(4-methoxyphenyl)-2-(2-phenylimidazo[1,2-a]pyrimidin-3-yl)acetate (**9af**)

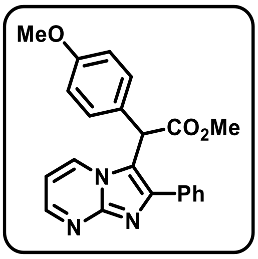

The **General Procedure B** was employed with 2-phenylimidazo[1,2-*a*]pyrimidine **7a** (39 mg, 0.2 mmol), methyl 2-diazo-2-(4-methoxyphenyl)acetate **8f** (124 mg, 0.6 mmol) and $CHCl_3$ (2 mL). Purification by flash column chromatography (SiO_2 pre-treated with 60:1 Hex:Et_3N, then gradient: Hex – 9:1 Hex:AcOEt – 8:2 Hex:AcOEt – 6:4 Hex:AcOEt – 1:1 Hex:AcOEt) afforded the title product as a yellow solid: 54 mg, 72%.

^{1}H NMR (300 MHz, $CDCl_3$) δ: 8.54 (dd, *J* = 4.1 Hz, *J* = 2.0 Hz, 1H), 8.32 (dd, *J* = 6.9 Hz, *J* = 2.0 Hz, 1H), 7.85 – 7.81 (m, 2H), 7.52 – 7.38 (m, 3H), 6.95 (d, *J* = 8.6 Hz, 2H), 6.82 (d, *J* = 8.6 Hz, 2H), 6.69 (dd, *J* = 6.9 Hz, *J* = 4.1 Hz, 1H), 5.87 (s, 1H), 3.82 (s, 3H), 3.76 (s, 3H).

^{13}C{^{1}H} NMR (75 MHz, $CDCl_3$) δ: 171.6, 159.2, 150.2, 148.7, 147.5, 134.2, 133.5, 129.2, 128.9, 128.7, 128.4, 125.7, 114.6 (x2), 108.1, 55.4, 52.9, 46.6.

IR (ATR, cm^{-1}): 2953, 1736, 1611, 1512, 1435, 1249, 1171, 1154, 1027.

M.P.: 134 – 136 °C.

HRMS (ESI+, Orbitrap), calcd. for [$C_{22}H_{19}N_3O_3$ + H]$^+$: 374.1499, found: 374.1492.

methyl 2-(4-fluorophenyl)-2-(2-phenylimidazo[1,2-a]pyrimidin-3-yl)acetate (**9ag**)

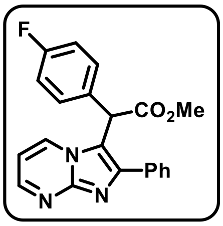

The **General Procedure B** was employed with 2-phenylimidazo[1,2-a]pyrimidine **7a** (39 mg, 0.2 mmol), methyl 2-diazo-2-(4-fluorophenyl)acetate **8g** (116 mg, 0.6 mmol) and $CHCl_3$ (2 mL). Purification by flash column chromatography (SiO_2 pre-treated with 60:1 Hex:Et_3N, then gradient: Hex – 9:1 Hex:AcOEt – 8:2 Hex:AcOEt – 6:4 Hex:AcOEt – 4:6 Hex:AcOEt) afforded the title product as a yellow solid: 47 mg, 65%.

^{1}H NMR (250 MHz, $CDCl_3$) δ: 8.56 (dd, *J* = 4.2 Hz, *J* = 2.0 Hz, 1H), 8.29 (dd, *J* = 7.0 Hz, *J* = 2.0 Hz, 1H), 7.85 – 7.80 (m, 2H), 7.53 – 7.39 (m, 3H), 7.02 – 6.99 (m, 4H), 6.72 (dd, *J* = 7.0 Hz, *J* = 4.2 Hz, 1H), 5.88 (d, *J* = 1.3 Hz, 1H), 3.83 (s, 3H).

^{13}C{^{1}H} NMR (62.5 MHz, $CDCl_3$) δ: 171.2, 162.3 (d, *J* = 246.3 Hz), 150.4, 148.8, 147.7, 133.9, 133.3, 129.6 (d, *J* = 3.8 Hz), 129.2, 129.1 (d, *J* = 8.1 Hz), 128.9, 128.8, 116.3 (d, *J* = 21.3 Hz), 114.1, 108.3, 53.1, 46.5.

^{19}F{^{1}H} NMR (235 MHz, $CDCl_3$) δ: -113.8.

IR (ATR, cm^{-1}): 2957, 1733, 1504, 1221, 1152.

M.P.: 159 – 161 °C.

HRMS (ESI+, Orbitrap), calcd. for [$C_{21}H_{16}FN_3O_2$ + H]$^+$: 362.1299, found: 362.1295.

methyl 2-(4-bromophenyl)-2-(2-phenylimidazo[1,2-a]pyrimidin-3-yl)acetate (**9ah**)

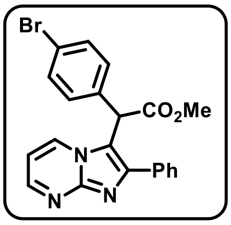

The **General Procedure B** was employed with 2-phenylimidazo[1,2-*a*]pyrimidine **7a** (39 mg, 0.2 mmol), methyl 2-(4-bromophenyl)-2-diazoacetate **8h** (153 mg, 0.6 mmol) and $CHCl_3$ (2 mL). Purification by flash column chromatography (SiO_2 pre-treated with 60:1 Hex:Et_3N, then gradient: Hex – 9:1 Hex:AcOEt – 8:2 Hex:AcOEt – 6:4 Hex:AcOEt – 4:6 Hex:AcOEt) afforded the title product as a yellow solid: 64 mg, 76%.

^{1}H NMR (250 MHz, $CDCl_3$) δ: 8.56 (dd, *J* = 4.1 Hz, *J* = 2.0 Hz, 1H), 8.28 (dd, *J* = 6.9 Hz, *J* = 2.0 Hz, 1H), 7.84 – 7.79 (m, 2H), 7.53 – 7.39 (m, 5H), 6.91 (d, *J* = 7.8 Hz, 2H), 6.73 (dd, *J* = 6.9 Hz, *J* = 4.1 Hz, 1H), 5.84 (s, 1H), 3.82 (s, 3H).

$^{13}C\{^1H\}$ NMR (62.5 MHz, $CDCl_3$) δ: 170.9, 150.5, 148.8, 147.8, 133.9, 133.3, 132.9, 132.4, 129.2, 129.1, 128.9 (x2), 122.2, 113.7, 108.5, 53.1, 46.7.

IR (ATR, cm^{-1}): 2951, 1731, 1490, 1152, 1011.

M.P.: 145 – 147 °C.

HRMS (ESI+, Orbitrap), calcd. for $[C_{21}H_{16}BrN_3O_2+H]^+$: 422.0499, found: 422.0497.

methyl 2-(2-chlorophenyl)-2-(2-phenylimidazo[1,2-a]pyrimidin-3-yl)acetate (**9ai**)

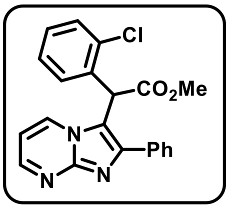

The **General Procedure B** was employed with 2-phenylimidazo[1,2-*a*]pyrimidine **7a** (39 mg, 0.2 mmol), methyl 2-(2-chlorophenyl)-2-diazoacetate **8i** (126 mg, 0.6 mmol) and $CHCl_3$ (2 mL). Purification by flash column chromatography (SiO_2 pre-treated with 60:1 Hex:Et_3N, then gradient: Hex – 9:1 Hex:AcOEt – 8:2 Hex:AcOEt – 6:4 Hex:AcOEt – 4:6 Hex:AcOEt) afforded the title product as a yellow solid: 47 mg, 62%.

^{1}H NMR (500 MHz, $CDCl_3$) δ: 8.60 (dd, *J* = 4.0 Hz, *J* = 2.0 Hz, 1H), 8.46 (dd, *J* = 7.0 Hz, *J* = 2.0 Hz, 1H), 7.73 – 7.71 (m, 2H), 7.48 - 7.44 (m, 3H), 7.42 – 7.39 (m, 1H), 7.30 (td, *J* = 7.5 Hz, *J* = 1.0 Hz, 1H), 7.18 (t, *J* = 7.5 Hz, *J* = 1.0 Hz, 1H), 6.86 (d, *J* = 7.5 Hz, 1H), 6.83 (dd, *J* = 7.0 Hz, *J* = 4.0 Hz, 1H), 5.96 (s, 1H), 3.68 (s, 3H).

$^{13}C\{^1H\}$ NMR (125 MHz, $CDCl_3$) δ: 169.8, 150.2, 148.6, 148.0, 134.6, 133.4, 133.1, 132.6, 130.7, 129.8, 129.1, 128.9, 128.8 (2x), 127.4, 113.0, 108.8, 53.2, 46.4.

IR (ATR, cm^{-1}): 3060, 2949, 1741, 1444, 1344, 1177.

M.P.: 141 – 143 °C.

HRMS (ESI+, Orbitrap), calcd. for $[C_{21}H_{16}ClN_3O_2 + H]^+$: 378.1004, found: 378.1001.

methyl 2-(3-chlorophenyl)-2-(2-phenylimidazo[1,2-a]pyrimidin-3-yl)acetate (**9aj**)

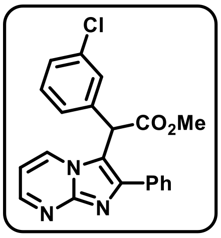

The **General Procedure B** was employed with 2-phenylimidazo[1,2-*a*]pyrimidine **7a** (39 mg, 0.2 mmol), methyl 2-(3-chlorophenyl)-2-diazoacetate **8j** (127 mg, 0.6 mmol) and $CHCl_3$ (2 mL). Purification by flash column chromatography (SiO_2 pre-treated with 60:1 Hex:Et_3N, then gradient: Hex – 9:1 Hex:AcOEt – 8:2 Hex:AcOEt –

6:4 Hex:AcOEt – 1:1 Hex:AcOEt) afforded the title product as a yellow solid: 50 mg, 66%.

^{1}H NMR (300 MHz, $CDCl_3$) δ: 8.62 (dd, *J* = 4.2 Hz, *J* = 2.0 Hz, 1H), 8.36 (dd, *J* = 6.9 Hz, *J* = 2.0 Hz, 1H), 7.90 - 7.86 (m, 2H), 7.59 – 7.46 (m, 3H), 7.35 – 7.26 (m, 2H), 7.12 (app q, *J* = 1.5 Hz, 1H), 6.95 (app dq, *J* = 7.2 Hz, *J* = 1.5 Hz, 1H), 6.80 (dd, *J* = 6.9 Hz, *J* = 4.2 Hz, 1H), 5.94 (s, 1H), 3.89 (s, 3H).

$^{13}C\{^1H\}$ NMR (75 MHz, $CDCl_3$) δ: 170.7, 150.5, 148.8, 147.9, 136.0, 135.4, 133.9, 133.3, 130.5, 129.2, 129.0, 128.9, 128.4, 127.5, 125.5, 113.6, 108.5, 53.2, 46.9.

IR (ATR, cm^{-1}): 2953, 1736, 1500, 1154.

M.P.: 56 – 58 °C.

HRMS (ESI+, Orbitrap), calcd. for [$C_{21}H_{16}ClN_3O_2$ + H]$^+$: 378.1004, found: 378.0998.

methyl 4-(2-methoxy-2-oxo-1-(2-phenylimidazo[1,2-a]pyrimidin-3-yl)ethyl)benzoate (**9ak**)

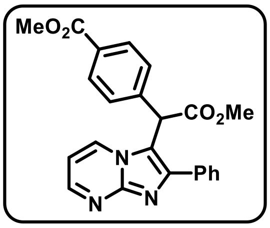

The **General Procedure B** was employed with 2-phenylimidazo[1,2-*a*]pyrimidine **7a** (39 mg, 0.2 mmol), methyl 4-(1-diazo-2-methoxy-2-oxoethyl)benzoate **8k** (140 mg, 0.6 mmol) and $CHCl_3$ (2 mL). Purification by flash column chromatography (SiO_2 pre-treated with 60:1 Hex:Et_3N, then gradient: Hex – 9:1 Hex:AcOEt – 8:2 Hex:AcOEt – 6:4 Hex:AcOEt – 1:1 Hex:AcOEt) afforded the title product as a yellow solid: 40 mg, 50%.

^{1}H NMR (300 MHz, $CDCl_3$) δ: 8.56 (dd, *J* = 4.1 Hz, *J* = 2.0 Hz, 1H), 8.25 (dd, *J* = 6.9 Hz, *J* = 2.0 Hz, 1H), 7.97 (d, *J* = 8.1 Hz, 2H), 7.86 – 7.82 (m, 2H), 7.53 – 7.40 (m, 3H), 7.12 (d, *J* = 8.1 Hz, 2H), 6.70 (dd, *J* = 6.9 Hz, *J* = 4.1 Hz, 1H), 5.95 (s, 1H), 3.89 (s, 3H), 3.86 (s, 3H).

$^{13}C\{^1H\}$ NMR (75 MHz, $CDCl_3$) δ: 170.8, 166.5, 150.5, 148.8, 148.0, 139.0, 133.9, 133.3, 130.5, 130.1, 129.2, 129.0, 128.9, 127.4, 113.7, 108.4, 53.2, 52.4, 47.3.

IR (ATR, cm^{-1}): 2952, 1721, 1614, 1500, 1436, 1279, 1111.

M.P.: 63 – 65 °C.

HRMS (ESI+, Orbitrap), calcd. for [$C_{23}H_{19}N_3O_4$ + H]$^+$: 402.1448, found: 402.1440.

methyl 2-(3,5-difluorophenyl)-2-(2-phenylimidazo[1,2-a]pyrimidin-3-yl)acetate (**9al**)

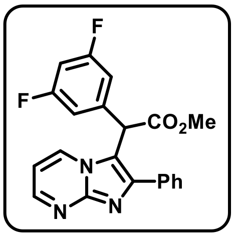

The **General Procedure B** was employed with 2-phenylimidazo[1,2-*a*]pyrimidine **7a** (39 mg, 0.2 mmol), methyl 2-diazo-2-(3,5-difluorophenyl)acetate **8l** (127 mg, 0.6 mmol) and $CHCl_3$ (2 mL). Purification by flash column chromatography (SiO_2 pre-treated with 60:1 Hex:Et_3N, then gradient: Hex – 9:1 Hex:AcOEt – 8:2 Hex:AcOEt – 6:4 Hex:AcOEt) afforded the title product as a yellow solid: 45 mg, 59%.

^{1}H NMR (500 MHz, $CDCl_3$) δ: 8.59 (dd, *J* = 4.0 Hz, *J* = 2.0 Hz, 1H), 8.32 (dd, *J* = 7.0 Hz, *J* = 2.0 Hz, 1H), 7.83 – 7.81 (m, 2H), 7.53 – 7.49 (m, 2H), 7.46 – 7.43 (m, 1H), 6.78 (dd, *J* = 7.0 Hz, *J* = 4.0 Hz, 1H), 6.74 (dt, *J* = 9.0 Hz, *J* = 2.0 Hz, 1H), 6.62 – 6.57 (m, 2H), 5.86 (s, 1H), 3.85 (s, 3H).

^{13}C{^{1}H} NMR (125 MHz, $CDCl_3$) δ: 170.3, 163.5 (dd, *J* = 248.8 Hz, *J* = 12.5 Hz), 150.7, 148.9, 148.2, 137.9 (t, *J* = 8.8 Hz), 133.7, 133.1, 129.2, 129.0 (x2), 113.0, 110.7 (dd, *J* = 20.0 Hz, *J* = 6.3 Hz), 108.7, 103.9 (t, *J* = 25.0 Hz), 53.4, 46.8.

^{19}F{^{1}H} NMR (470 MHz, $CDCl_3$) δ: -107.6.

IR (ATR, cm^{-1}): 1736, 1624, 1598, 1499, 1317, 1220, 1157, 1119.

M.P.: 62 – 64 °C.

HRMS (ESI+, Orbitrap), calcd. for $[C_{21}H_{15}F_2N_3O_2 + H]^+$: 380.1205, found: 380.1197.

isopropyl 2-(3,4-dichlorophenyl)-2-(2-phenylimidazo[1,2-a]pyrimidin-3-yl)acetate (**9am**)

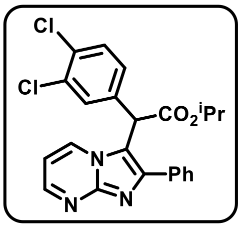

The **General Procedure B** was employed with 2-phenylimidazo[1,2-*a*]pyrimidine **7a** (39 mg, 0.2 mmol), isopropyl 2-diazo-2-(3,4-dichlorophenyl)acetate **8m** (164 mg, 0.6 mmol) and $CHCl_3$ (2 mL). Purification by flash column chromatography (SiO_2 pre-treated with 60:1 Hex:Et_3N, then gradient: Hex – 9:1 Hex:AcOEt – 8:2 Hex:AcOEt – 6:4 Hex:AcOEt) afforded the title product as a yellow solid: 65 mg, 74 %.

^{1}H NMR (600 MHz, $CDCl_3$) δ: 8.58 (dd, *J* = 4.0 Hz, *J* = 2.0 Hz, 1H), 8.32 (dd, *J* = 7.0 Hz, *J* = 2.0 Hz, 1H), 7.85 – 7.84 (m, 2H), 7.52 – 7.49 (m, 2H), 7.45 – 7.43 (m, 1H), 7.36 (d, *J* = 8.4 Hz, 1H), 7.17 (dd, *J* = 2.0 Hz, *J* = 1.2 Hz, 1H), 6.85 (ddd, *J* = 8.4 Hz, *J* = 2.0

Hz, *J* = 1.2 Hz, 1H), 6.75 (dd, *J* = 7.0 Hz, *J* = 4.0 Hz, 1H), 5.76 (t, *J* = 1.2 Hz, 1H), 5.15 (sept, *J* = 6.3 Hz, 1H), 1.34 (d, *J* = 6.3 Hz, 3H), 1.24 (d, *J* = 6.3 Hz, 3H).

$^{13}C\{^1H\}$ NMR (150 MHz, $CDCl_3$) δ: 169.5, 150.6, 148.8, 148.1, 134.4, 133.9, 133.6, 133.2, 132.4, 131.1, 129.4, 129.3, 128.9 (x2), 126.7, 113.5, 108.5, 70.7, 46.8, 22.0, 21.8.

IR (ATR, cm^{-1}): 2980, 1727, 1616, 1499, 1472, 1349, 1216, 1195, 1101, 1031.

M.P.: 183 – 185 °C.

HRMS (ESI+, Orbitrap), calcd. for [$C_{23}H_{19}Cl_2N_3O_2$ + H]$^+$: 440.0927, found: 440.0920.

isopropyl 2-(3,4-dichlorophenyl)-2-(2-phenylimidazo[1,2-a]pyridin-3-yl)acetate (**9lm**)

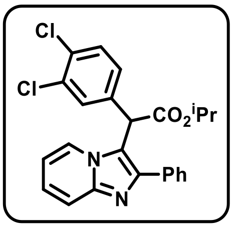

The **General Procedure B** was employed with 2-phenylimidazo[1,2-*a*]pyridine **7l** (39 mg, 0.2 mmol), isopropyl 2-diazo-2-(3,4-dichlorophenyl)acetate **8m** (218 mg, 0.8 mmol) and $CHCl_3$ (2 mL). Purification by flash column chromatography (SiO_2 pre-treated with 60:1 Hex:Et_3N, then gradient: Hex – 9:1 Hex:AcOEt – 85:15 Hex:AcOEt) afforded the title product as a brown solid: 69 mg, 79%.

^1H NMR (400 MHz, $CDCl_3$) δ: 7.96 (dt, *J* = 6.8 Hz, *J* = 1.2 Hz, 1H), 7.77 – 7.75 (m, 2H), 7.70 (dt, *J* = 8.8 Hz, *J* = 1.2 Hz, 1H), 7.50 – 7.46 (m, 2H), 7.43 – 7.39 (m, 1H), 7.35 (d, *J* = 8.4 Hz, 1H), 7.24 (ddd, *J* = 8.8 Hz, *J* = 6.8 Hz, *J* = 1.2 Hz, 1H), 7.21 (dd, *J* = 2.0 Hz, *J* = 1.0 Hz, 1H), 6.86 (dd, *J* = 8.4 Hz, *J* = 1.0 Hz, 1H), 6.70 (td, *J* = 6.8 Hz, *J* = 1.2 Hz, 1H), 5.72 (s, 1H), 5.12 (sept, *J* = 6.3 Hz, 1H), 1.30 (d, *J* = 6.3 Hz, 3H), 1.18 (d, *J* = 6.3 Hz, 3H).

$^{13}C\{^1H\}$ NMR (100 MHz, $CDCl_3$) δ: 169.5, 146.6, 145.9, 135.0, 133.9, 133.3, 132.0, 130.9, 129.8, 129.1, 128.9, 128.5, 127.1, 125.9, 125.4, 117.9, 114.7, 112.3, 70.4, 46.9, 22.0, 21.7.

M.P.: 48 - 50 ^oC.

IR (ATR, cm^{-1}): 2980, 1727, 1637, 1504, 1472, 1376, 1358, 1249, 1190, 1031, 1101.

HRMS (ESI+, Orbitrap), calcd. for [$C_{24}H_{20}Cl_2N_2O_2$ + H]$^+$: 439.0975, found: 439.0968.

methyl 2-phenyl-2-(2-phenylimidazo[1,2-a]pyridin-3-yl)acetate[15] (**9la**)

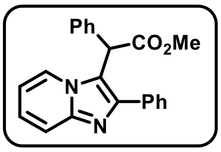

The **General Procedure B** was employed with 2-phenylimidazo[1,2-*a*]pyridine **7l** (39 mg, 0.2 mmol), methyl 2-diazo-2-phenylacetate **8a** (141 mg, 0.8 mmol) and $CHCl_3$ (2 mL). Purification by flash column chromatography (SiO_2 pre-treated with 60:1 Hex:Et_3N, then gradient: Hex – 9:1 Hex:AcOEt – 8:2 Hex:AcOEt – 7:3 Hex:AcOEt) afforded the title product as a yellow solid: 38 mg, 56%.

^{1}H NMR (600 MHz, $CDCl_3$) δ: 7.98 (dt, *J* = 7.0 Hz, *J* = 1.2 Hz, 1H), 7.78 – 7.76 (m, 2H), 7.69 (dt, *J* = 9.0 Hz, *J* = 1.2 Hz, 1H), 7.49 – 7.46 (m, 2H), 7.42 – 7.38 (m, 1H), 7.32 – 7.28 (m, 3H), 7.20 (ddd, *J* = 9.0 Hz, *J* = 6.8 Hz, *J* = 1.2 Hz, 1H), 7.07 – 7.05 (m, 2H), 6.65 (td, *J* = 6.8 Hz, *J* = 1.2 Hz, 1H), 5.90 (s, 1H), 3.79 (s, 3H).

^{13}C{^{1}H} NMR (150 MHz, $CDCl_3$) δ: 171.4, 146.3, 145.8, 134.5, 134.2, 129.1 (x2), 128.8, 128.3, 127.8, 127.6, 126.3, 125.0, 117.7, 115.5, 112.0, 52.8, 47.3.

IR (ATR, cm^{-1}): 3056, 3027, 2948, 1731, 1240, 1155.

M.P.: 92 – 94 °C.

HRMS (ESI+, Orbitrap), calcd. for [$C_{22}H_{18}N_2O_2$ + H]$^+$: 343.1441, found: 343.1433.

methyl 2-(2-(4-methoxyphenyl)imidazo[1,2-a]pyridin-3-yl)-2-phenylacetate[15] (**9ma**)

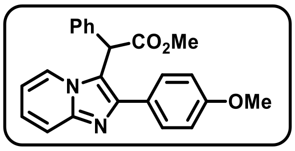

The **General Procedure B** was employed with 2-(4-methoxyphenyl)imidazo[1,2-*a*]pyridine **7m** (45 mg, 0.2 mmol), methyl 2-diazo-2-phenylacetate **8a** (141 mg, 0.8 mmol) and $CHCl_3$ (2 mL). Purification by flash column chromatography (SiO_2 pre-treated with 60:1 Hex:Et_3N, then gradient: Hex – 95:5 Hex:AcOEt – 85:15 Hex:AcOEt) afforded the title product as a brownish solid: 65 mg, 87%.

^{1}H NMR (600 MHz, $CDCl_3$) δ: 7.96 (dt, *J* = 7.2 Hz, *J* = 1.2 Hz, 1H), 7.71 (d, *J* = 9.0 Hz, 2H), 7.66 (dt, *J* = 9.0 Hz, *J* = 1.2 Hz, 1H), 7.31 – 7.27 (m, 3H), 7.18 (ddd, *J* = 9.0 Hz, *J* = 6.6 Hz, *J* = 1.2 Hz, 1H), 7.06 – 7.05 (m, 2H), 7.01 (d, *J* = 9.0 Hz, 2H), 6.63 (td, *J* = 7.2 Hz, *J* = 1.2 Hz, 1H), 5.87 (s, 1H), 3.85 (s, 3H), 3.79 (s, 3H).

^{13}C{^{1}H} NMR (150 MHz, $CDCl_3$) δ: 171.5, 159.8, 146.1, 145.7, 134.5, 130.3, 129.1, 127.8, 127.6, 126.7, 126.2, 124.9, 117.5, 115.0, 114.3, 111.9, 55.5, 52.8, 47.4.

---

[15] ^{1}H and ^{13}C{^{1}H} NMR spectra are in good agreement with the literature. See: H. Kim, M. Byeon, E. Jeong, Y. Baek, S. J. Jeong, K. Um, S. H. Han, G. U. Han, G. H. Ko, C. Maeng, J.-Y. Son, D. Kim, S. H. Kim, K. Lee, P. H. Lee, *Adv. Synth. Catal.*, **2019**, *361*, 2094-2106.

IR (ATR, cm^{-1}): 2957, 2839, 1728, 1614, 1497, 1432, 1388, 1238, 1174, 1158.
HRMS (ESI+, Orbitrap), calcd. for [$C_{23}H_{20}N_2O_3$ + H]$^+$: 373.1547, found: 373.1539.

methyl 2-(2-([1,1'-biphenyl]-4-yl)imidazo[1,2-a]pyridin-3-yl)-2-phenylacetate (**9na**)

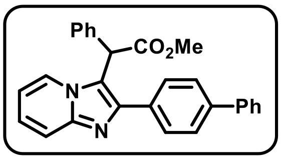

The **General Procedure B** was employed with 2-([1,1'-biphenyl]-4-yl)imidazo [1,2-*a*]pyridine **7n** (54 mg, 0.2 mmol), methyl 2-diazo-2-phenylacetate **8a** (141 mg, 0.8 mmol) and $CHCl_3$ (2 mL). Purification by flash column chromatography (SiO_2 pre-treated with 60:1 Hex:Et_3N, then gradient: Hex – 95:5 Hex:AcOEt – 85:15 Hex:AcOEt) afforded the title product as a dark brown solid: 59 mg, 71%.

^{1}H NMR (600 MHz, $CDCl_3$) δ: 7.99 (dt, *J* = 7.0 Hz, *J* = 1.0 Hz, 1H), 7.86 (d, *J* = 8.5 Hz, 2H), 7.73 (d, *J* = 8.5 Hz, 2H), 7.70 (dt, *J* = 9.0 Hz, *J* = 1.0 Hz, 1), 7.67 - 7.65 (m, 2H), 7.46 (t, *J* = 7.5 Hz, 2H), 7.38 – 7.35 (m, 1H), 7.33 – 7.29 (m, 3H), 7.21 (ddd, *J* = 9.0 Hz, *J* = 6.5 Hz, *J* = 1.4 Hz, 1H), 7.09 (d, *J* = 7.2 Hz, 2H), 6.65 (td, *J* = 7.0 Hz, *J* = 1.4 Hz, 1H), 5.96 (s, 1H), 3.81 (s, 3H).
^{13}C{^{1}H} NMR (150 MHz, $CDCl_3$) δ: 171.4, 145.9 (x2), 141.0, 140.8, 134.4, 133.2, 129.4, 129.1, 129.0, 127.8, 127.6 (x2), 127.5, 127.2, 126.3, 125.1, 117.7, 115.6, 112.0, 52.9, 47.4.
M.P.: 188 – 190 °C
IR (ATR, cm^{-1}): 2951, 1734, 1494, 1433, 1331, 1258, 1154, 1009.
HRMS (ESI+, Orbitrap), calcd. for [$C_{28}H_{22}N_2O_2$ + H]$^+$: 419.1754, found: 419.1745.

methyl 2-(4-methoxyphenyl)-2-(2-phenylimidazo[1,2-a]pyridin-3-yl)acetate[15] (**9lf**)

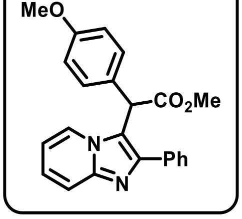

The **General Procedure B** was employed with 2-phenylimidazo[1,2-*a*]pyridine **7l** (39 mg, 0.2 mmol), methyl 2-diazo-2-(4-methoxyphenyl)acetate **8f** (165 mg, 0.8 mmol) and $CHCl_3$ (2 mL). Purification by flash column chromatography (SiO_2 pre-treated with 60:1 Hex:Et_3N, then gradient: Hex – 9:1 Hex:AcOEt – 8:2 Hex:AcOEt) afforded the title product as a white solid: 60 mg, 81%.

This reaction was also performed in a 2-mmol scale, according to **General Procedure B** employing 2-phenylimidazo[1,2-*a*]pyridine **7l** (390 mg, 2 mmol), methyl 2-diazo-2-(4-

methoxyphenyl)acetate **8f** (1.65 g, 8 mmol) and $CHCl_3$ (20 mL). Purification by flash column chromatography (SiO_2 pre-treated with 60:1 Hex:Et_3N, then gradient: Hex – 9:1 Hex:AcOEt – 8:2 Hex:AcOEt) afforded the title product as a white solid: 558 mg, 75%.

^{1}H NMR (500 MHz, $CDCl_3$) δ: 7.99 (dt, *J* = 7.0 Hz, *J* = 1.3 Hz, 1H), 7.77 – 7.75 (m, 2H), 7.67 (dt, *J* = 9.0 Hz, *J* = 1.3 Hz, 1H), 7.48 – 7.45 (m, 2H), 7.40 – 7.37 (m, 1H), 7.18 (ddd, *J* = 9.0 Hz, *J* = 7.0 Hz, *J* = 1.0 Hz, 1H), 6.99 – 6.96 (m, 2H), 6.82 (d, *J* = 9.0 Hz, 2H), 6.64 (td, *J* = 7.0 Hz, *J* = 1.0 Hz, 1H), 5.82 (s, 1H), 3.76 (s, 3H), 3.75 (s, 3H).
^{13}C{^{1}H} NMR (125 MHz, $CDCl_3$) δ: 171.6, 159.0, 146.0, 145.7, 134.2, 129.0, 128.7 (x2), 128.2, 126.3, 126.2, 124.9, 117.6, 115.8, 114.4, 111.9, 55.3, 52.7, 46.6.
IR (ATR, cm^{-1}): 2944, 1722, 1432, 1272, 1110.
HRMS (ESI+, Orbitrap), calcd. for [$C_{23}H_{20}N_2O_3$+ H]$^+$: 373.1547, found: 373.1538.

methyl 4-(2-methoxy-2-oxo-1-(2-phenylimidazo[1,2-a]pyridin-3-yl)ethyl)benzoate (**9lk**)

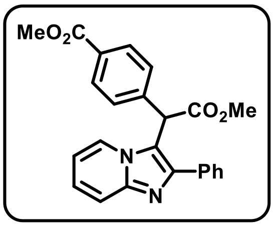

The **General Procedure B** was employed with 2-phenylimidazo[1,2-*a*]pyridine **7l** (39 mg, 0.2 mmol), methyl 4-(1-diazo-2-methoxy-2-oxoethyl)benzoate **8k** (187 mg, 0.8 mmol) and $CHCl_3$ (2 mL). Purification by flash column chromatography (SiO_2 pre-treated with 60:1 Hex:Et_3N, then gradient: Hex – 9:1 Hex:AcOEt – 8:2 Hex:AcOEt) afforded the title product as a brown solid: 58 mg, 73%.
^{1}H NMR (500 MHz, $CDCl_3$) δ: 7.97 (d, *J* = 8.5 Hz, 2H), 7.90 (dt, *J* = 7.0 Hz, *J* = 1.0 Hz, 1H), 7.77 – 7.75 (m, 2H), 7.69 (dt, *J* = 9.0 Hz, *J* = 1.3 Hz, 1H), 7.50 – 7.47 (m, 2H), 7.42 – 7.39 (m, 1H), 7.21 (ddd, *J* = 9.0 Hz, *J* = 7.0 Hz, *J* = 1.3 Hz, 1H), 7.14 (d, *J* = 8.5 Hz, 2H), 6.65 (td, *J* = 7.0 Hz, *J* = 1.3 Hz, 1H), 5.91 (s, 1H), 3.89 (s, 3H), 3.80 (s, 3H).
^{13}C{^{1}H} NMR (125 MHz, $CDCl_3$) δ: 170.9, 166.7, 146.6, 145.9, 139.6, 134.0, 130.3, 129.8, 129.1, 128.9, 128.4, 127.8, 126.0, 125.2, 117.9, 114.9, 112.3, 53.0, 52.4, 47.4.
M.P.: 116 – 118 °C.
IR (ATR, cm^{-1}): 2946, 1722, 1272, 1108.
HRMS (ESI+, Orbitrap), calcd. for [$C_{24}H_{20}N_2O_4$+H]$^+$: 401.1496, found: 401.1490.

1.2.5. Preliminary Investigations aiming at the Blue Light-Mediated Alkylation of Indolizine 7o with Aryldiazoacetate 8a

Table S1. Preliminary attempts to react **7o** with **8a** under blue light irradiation. [a]Observations made by ^{1}H NMR analysis of the crude reaction mixture, reactions performed with two blue LED lamps, except if otherwise noted. [b]Reaction performed in the absence of blue light irradiation. [c]Reaction performed with only one blue LED lamp.

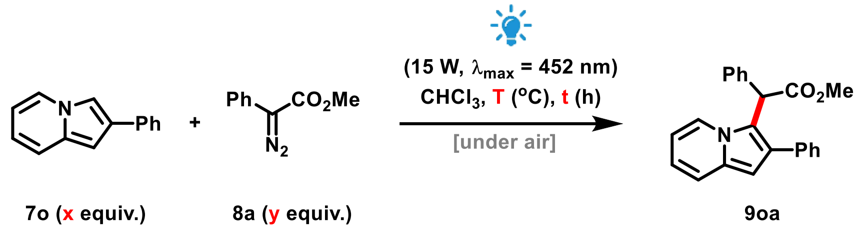

entry	x	y	T (°C)	t (h)	**9oa** (%)[a]
1	1	3	rt	24	degradation
2	3	1	rt	24	degradation
3	1	3	rt	6	degradation
4[b]	1	3	rt	24	no reaction, **7o** and **8a** recovered
5[b]	3	1	rt	24	no reaction, **7o** and **8a** recovered
6	1	3	-10	24	degradation
7	1	3	-10	3	degradation
8[c]	1	3	rt	5	degradation

1.2.6. Formal Synthesis of Zolpidem

Table S2. First round of preliminary studies for the conversion of **7p** to **13** or **14**. [a]Estimated yield based on the ^{1}H NMR analysis of crude reaction mixture using 1,3,5-trimethoxybenzene as internal standard.

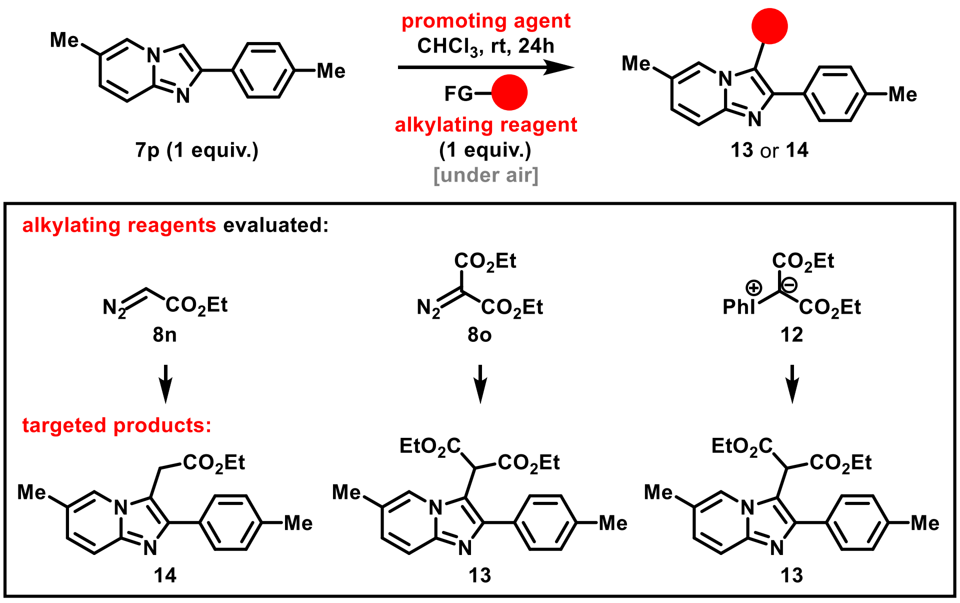

entry	alkylating reagent	promoting agent	yield of **13** or **14** (%)[a]	yield of recovered **7p** (%)[a]
1	**8n**	blue light (15W, λ_{max} = 452 nm)	**14**, < 10	< 10
2	**8n**	80 °C (in 1,2-DCE)	**14**, < 10	81
3	**8o**	blue light (15W, λ_{max} = 452 nm)	**13**, < 10	86
4	**8o**	80 °C (in 1,2-DCE)	**13**, < 10	96
**5**	**12**	**blue light (15W, λ_{max} = 452 nm)**	**13, 51**	**40**
6	**12**	rt (in the absence of blue light)	**13**, 44	27
7	**12**	80 °C (in 1,2-DCE)	**13**, 11	45
8	**12**	blue light (15W, λ_{max} = 452 nm), reaction in Schlenk tube, degassed (via freeze-pump-thaw) and performed under N_2	**13**, 47	30

Table S3. Second round of preliminary studies for the conversion of **7p** to **13**. [a]Estimated yield based on the ^{1}H NMR analysis of crude reaction mixture using 1,3,5-trimethoxybenzene as internal standard. [b]Yield is based on the integration of ^{1}H NMR signs of **7p**, used in excess, in relation to the internal standard.

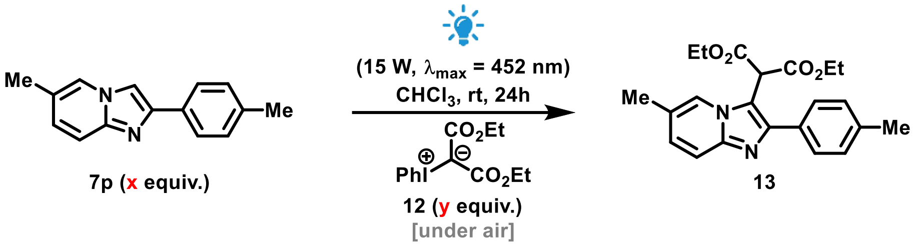

entry	x	y	yield **13** (%)[a]	yield of recovered **7p** (%)[a]
1	1	2	22	< 10
2	1	3	< 10	< 10
3	2	1	50	70[b]
4	3	1	30	86[b]

Purification of product **13** by flash column chromatography was found to be challenging. Because under the optimal conditions (Entry 5, Table S1), part of the heterocycle **7p** remained in the crude reaction mixture, and it was inseparable from **13** (*i.e.* very similar R_f), a second step involving a Krapcho decarboxylation was performed. This second step proceeded in high yield and allowed the isolation of the subsequent product **14**. This transformation was not further extensively optimized.

ethyl 2-(6-methyl-2-(p-tolyl)imidazo[1,2-a]pyridin-3-yl)acetate[16] (**14**)

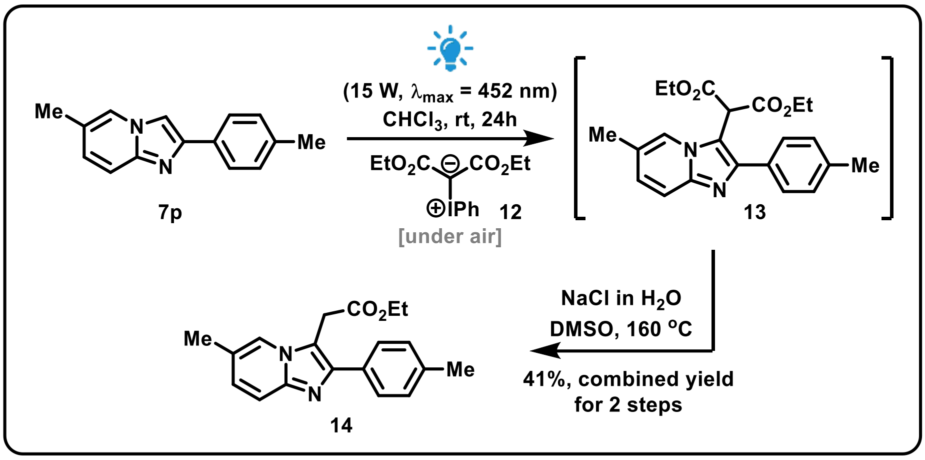

The **General Procedure B** was employed with 6-methyl-2-(*p*-tolyl)imidazo[1,2-a]pyridine **7p** (45 mg, 0.2 mmol, 1 equiv.), diethyl 2-(phenyl-λ^3-iodanylidene)malonate **12** (72 mg, 0.2 mmol, 1 equiv.), and $CHCl_3$ (2 mL), reaction time of 24 h. After the reaction was completed (TLC), the reaction mixture was concentrated under reduced pressure. Then, DMSO (3 mL) and a solution of NaCl (19 mg, 0.32 mmol, 1.6 equiv.) in H_2O (1 mL) were added. The resulting reaction mixture was heated to 160 °C and stirred at this temperature for 15 hours.[17] After reaction completion (TLC), the reaction mixture was diluted in H_2O, extracted with AcOEt (3×), dried (Na_2SO_4), and concentrated under reduced pressure. Purification by flash column chromatography (SiO_2, pre-treated with 60:1 Hex:Et_3N, then gradient: Hex – 95:5 Hex:AcOEt – 85:15 Hex:AcOEt) afforded the title compound as a yellowish solid: 25 mg, 41%.

This reaction was also performed in a 2-mmol scale, according to **General Procedure B** employing 6-methyl-2-(*p*-tolyl)imidazo[1,2-a]pyridine **7p** (450 mg, 2 mmol), diethyl 2-(phenyl-λ^3-iodanylidene)malonate **12** (724 mg, 2 mmol) and $CHCl_3$ (20 mL). Then, DMSO (30 mL) and a solution of NaCl (190 mg, 3.2 mmol) in H_2O (10 mL) were used for the Krapcho decarboxylation step.[17] Purification by flash column chromatography (SiO_2, pre-treated with 60:1 Hex:Et_3N, then gradient: Hex – 95:5 Hex:AcOEt – 85:15 Hex:AcOEt) afforded the title compound as a yellowish solid: 229 mg, 37%.

---

[16] ^1H and $^{13}C\{^1H\}$ NMR data are in good agreement with the literature. See: Q. Chang, Z. Liu, P. Liu, L. Yu, P. Sun, *J. Org. Chem.* **2017**, *82*, 5391-5397.
[17] The second step was adapted from: N. R. Chaubey, A. R. Kapdi, B. Maity, *Synthesis* **2021**, *53*, 1524-1530.

^{1}H NMR (500 MHz, CDCl$_3$) δ: 7.88 (d, *J* = 1.5 Hz, 1H), 7.72 (d, *J* = 8.0 Hz, 2H), 7.55 (d, *J* = 9.0 Hz, 1H), 7.28 (d, *J* = 8.0 Hz, 2H), 7.06 (dd, *J* = 9.0 Hz, *J* = 1.5 Hz, 1H), 4.22 (q, *J* = 7.0 Hz, 2H), 4.00 (s, 2H), 2.40 (s, 3H), 2.36 (s, 3H), 1.28 (t, *J* = 7.0 Hz, 3H).

^{13}C{^{1}H} NMR (125 MHz, CDCl$_3$) δ: 169.7, 144.6, 144.2, 137.7, 131.5, 129.4, 128.5, 127.6, 122.1, 121.5, 117.0, 112.5, 61.7, 31.0, 21.4, 18.6, 14.3.

M.P.: 93 – 95 ºC.

IR (ATR, cm^{-1}): 3442, 2923, 1730, 1541, 1504, 1391, 1347, 1275, 1258, 1185, 1141.

HRMS (ESI+): calcd. for [$C_{19}H_{20}N_2O_2$ + H^+]: 309.1598, found: 309.1594.

Structure defined by single crystal X-ray diffraction (SC-XRD) of **14** (CCDC 2427913)

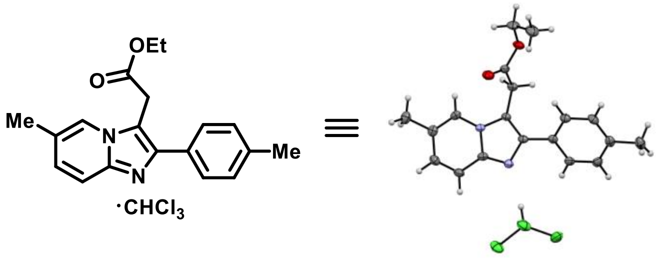

(ORTEP representation with ellipsoids showing 50% of probability)

Crystal data

$C_{19}H_{20}N_2O_2$·$CHCl_3$	*F*(000) = 888
M_r = 427.74	D_x = 1.382 Mg m^{-3}
Monoclinic, $P2_1/c$	Mo *K*α radiation, λ = 0.71073 Å
a = 8.7104 (13) Å	Cell parameters from 131 reflections
b = 18.838 (3) Å	θ = 2.2–20.9°
c = 12.5324 (17) Å	μ = 0.46 mm^{-1}
β = 90.740 (5)°	*T* = 120 K
V = 2056.2 (5) Å^3	Needle, orange
Z = 4	0.36 × 0.14 × 0.05 mm

Data collection

Bruker APEX CCD detector diffractometer	4358 independent reflections
Radiation source: fine-focus sealed tube	3288 reflections with *I* > 2σ(*I*)

Detector resolution: 8.3333 pixels mm^{-1}	R_{int} = 0.053
phi and ω scans	θ_{max} = 26.7°, θ_{min} = 2.0°
Absorption correction: numerical *SADABS* (Bruker, 2010)	h = -11 → 11
T_{min} = 0.882, T_{max} = 1.000	k = -23 → 23
26253 measured reflections	l = -15 → 15

Refinement

Refinement on F^2	Primary atom site location: structure-invariant direct methods
Least-squares matrix: full	Hydrogen site location: inferred from neighbouring sites
$R[F^2 > 2\sigma(F^2)]$ = 0.036	H-atom parameters constrained
$wR(F^2)$ = 0.086	$w = 1/[\sigma^2(F_o^2) + (0.0335P)^2 + 1.0726P]$ where $P = (F_o^2 + 2F_c^2)/3$
S = 1.02	$(\Delta/\sigma)_{max}$ = 0.001
4358 reflections	$\Delta\rangle_{max}$ = 0.22 e $Å^{-3}$
247 parameters	$\Delta\rangle_{min}$ = -0.26 e $Å^{-3}$
0 restraints	

2. Theoretical Calculations

2.1. Frontier Molecular Orbitals

All molecules were optimized and had their frequencies calculated at standard temperature and pressure using the M06-2X/aug-cc-pVTZ level in Gaussian 16.[18] The lack of imaginary frequencies was used to characterize true minima. Solvent effects were included using the IEFPCM[19] method with parameters of $CHCl_3$.

[18] Gaussian 16, Revision C.01, M. J. Frisch, G. W. Trucks, H. B. Schlegel, G. E. Scuseria, M. A. Robb, J. R. Cheeseman, G. Scalmani, V. Barone, G. A. Petersson, H. Nakatsuji, X. Li, M. Caricato, A. V. Marenich, J. Bloino, B. G. Janesko, R. Gomperts, B. Mennucci, H. P. Hratchian, J. V. Ortiz, A. F. Izmaylov, J. L. Sonnenberg, D. Williams-Young, F. Ding, F. Lipparini, F. Egidi, J. Goings, B. Peng, A. Petrone, T. Henderson, D. Ranasinghe, V. G. Zakrzewski, J. Gao, N. Rega, G. Zheng, W. Liang, M. Hada, M. Ehara, K. Toyota, R. Fukuda, J. Hasegawa, M. Ishida, T. Nakajima, Y. Honda, O. Kitao, H. Nakai, T. Vreven, K. Throssell, J. A. Montgomery, Jr., J. E. Peralta, F. Ogliaro, M. J. Bearpark, J. J. Heyd, E. N. Brothers, K. N. Kudin, V. N. Staroverov, T. A. Keith, R. Kobayashi, J. Normand, K. Raghavachari, A. P. Rendell, J. C. Burant, S. S. Iyengar, J. Tomasi, M. Cossi, J. M. Millam, M. Klene, C. Adamo, R. Cammi, J. W. Ochterski, R. L. Martin, K. Morokuma, O. Farkas, J. B. Foresman, and D. J. Fox, Gaussian, Inc., Wallingford CT, **2016**.

[19] B. Mennucci, R. Cammi, J. Tomasi, *J. Chem. Phys.* **1998**, *109*, 2798-2807.

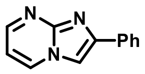

7a

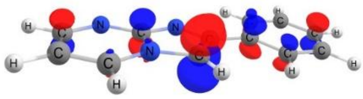

HOMO of 7a
Energy (eV) = -0.27159

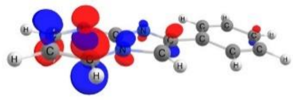

LUMO of 7a
Energy (eV) = -0.03769

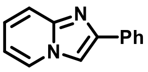

7l

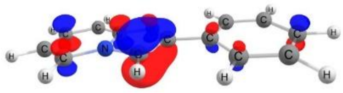

HOMO of 7l
Energy (eV) = -0.26460

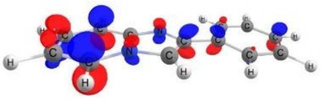

LUMO of 7l
Energy (eV) = -0.02205

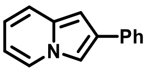

7o

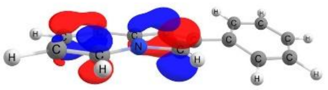

HOMO of 7o
Energy (eV) = -0.24571

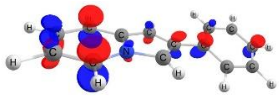

LUMO of 7o
Energy (eV) = -0.01383

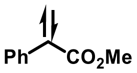

10a (S)

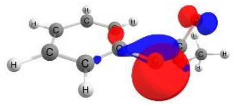

HOMO of singlet carbene 10a
Energy (eV) = -0.29241

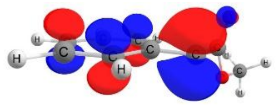

LUMO of singlet carbene 10a
Energy (eV) = -0.09567

Figure S5. HOMO and LUMO of heterocycles **7a**, **7l**, and **7o,** and of the singlet carbene **10a**, calculated at the M06-2X/aug-cc-pVTZ level. Calculated energies are given in electron-volts (eV).

Table S4. Cartesian coordinates, electronic and Gibbs free energies in hartrees and first harmonic frequency in cm^{-1} obtained for the *aza*-heterocycles **7a**, **7l** and **7o**, and singlet carbene **10a** at the M06-2X/aug-cc-pVTZ level in implicit $CHCl_3$ (IEFPCM implicit solvent model).

Imidazo[1,2-a]pyrimidine 7a
Electronic Energy = -626.9294701 hartrees
Gibbs Free Energy = -626.778041 hartrees
First Harmonic Frequency = 36.9682 cm^{-1}

C	1.760222207	-0.691199115	-0.025610041
C	3.052174265	1.332819052	0.049709964
C	4.169293357	0.565118015	0.022500962
C	4.019837371	-0.845326089	-0.030079041
N	2.865884299	-1.457430149	-0.053423043
N	0.504037124	-1.079286168	-0.040211042
C	0.562487089	1.182138000	0.040492964
H	3.044443245	2.411908132	0.089717967
H	5.147055419	1.018197068	0.040463964
H	4.903336450	-1.471914117	-0.052881043
H	0.352210054	2.235149067	0.085661967
N	1.846477188	0.706421988	0.024650963
C	-0.241744950	0.065679902	-0.001178039
C	-1.709220056	0.025624873	-0.002409039
C	-2.373182079	-1.198620225	0.054619965
C	-3.759325179	-1.246029257	0.057613965
C	-4.500234252	-0.073061183	0.003498961
C	-3.845105229	1.151213918	-0.055441043
C	-2.460578130	1.200047945	-0.059164044
H	-1.962143111	2.159080023	-0.112081047
H	-5.580947325	-0.111000205	0.005798961

H	-4.414807284	2.069327973	-0.101003047
H	-1.789106024	-2.106820282	0.097069968
H	-4.262578198	-2.202302331	0.102800968

Imidazo[1,2-a]pyridine 7l

Electronic Energy = -610.8863318 hartrees

Gibbs Free Energy = -610.723226 hartrees

First Harmonic Frequency = 35.3637 cm^{-1}

C	-1.747017123	-0.694903048	0.029073002
C	-3.034598219	1.351784098	-0.056960004
C	-4.183718300	0.635729046	-0.029029002
C	-4.140046296	-0.784850057	0.030948002
C	-2.947839212	-1.440812105	0.059562004
N	-0.485167035	-1.086122079	0.045789003
C	-0.550751038	1.169731084	-0.044297003
H	-2.987447214	2.428965177	-0.101959007
H	-5.128026369	1.157081081	-0.052773004
H	-5.065646362	-1.341732095	0.053805004
H	-2.880390206	-2.517467182	0.104866008
H	-0.344665025	2.223310160	-0.094216007
N	-1.835620133	0.692035051	-0.026936002
C	0.259699019	0.057718004	0.002150000
C	1.727796125	0.022506002	0.003110000
C	2.397450174	-1.198291088	-0.065200005
C	3.783795271	-1.241127092	-0.069026005
C	4.521276323	-0.066403005	-0.004474000
C	3.861182279	1.154497082	0.065786005
C	2.476259176	1.198302084	0.070444005
H	1.974283141	2.154972157	0.132702009
H	5.602118383	-0.100481007	-0.007343001
H	4.427273319	2.074483149	0.119769009
H	1.816942129	-2.108408152	-0.115994008
H	4.290141311	-2.195396155	-0.123355009

Indolizine 7o

Electronic Energy = -594.82564 hartrees

Gibbs Free Energy = -594.651269 hartrees

First Harmonic Frequency = 41.8772 cm^{-1}

C	1.846838135	0.745883053	0.112721008
C	2.982314216	-1.368942098	-0.215811016
C	4.181908302	-0.753378055	-0.121598009
C	4.250603304	0.654688049	0.100944007
C	3.109128226	1.382590098	0.215002015
C	0.531983037	1.170537085	0.175545013
C	0.530191036	-1.063753074	-0.157452011
H	2.855433207	-2.428155176	-0.381490027
H	5.081625367	-1.341679098	-0.216469015
H	5.215310373	1.135072081	0.176480012
H	3.133079227	2.450152174	0.381875028
H	0.216163015	2.184885155	0.350495025
H	0.302733022	-2.099761153	-0.339300024
N	1.826404130	-0.637984045	-0.096748007
C	-0.293850021	0.034853002	0.008722001
C	-1.762538125	0.012002001	0.005847000
C	-2.491409177	1.171487085	-0.263537019
C	-3.878214278	1.155515082	-0.267900019
C	-4.565491326	-0.022492002	-0.007286001
C	-3.851700275	-1.183615087	0.261259019
C	-2.465642177	-1.165100085	0.271435020
H	-1.920963141	-2.070721149	0.504628036
H	-5.646707387	-0.035716003	-0.011542001
H	-4.376693314	-2.105545150	0.472236034
H	-1.965906143	2.090756149	-0.485722035
H	-4.423613318	2.064950148	-0.481308035

Singlet carbene 10a

Electronic Energy = -498.0973605 hartrees

Gibbs Free Energy = -497.986308 hartrees

First Harmonic Frequency = 56.1511 cm^{-1}

C	0.529169030	-1.197006060	0.020036963
C	-0.708268039	-0.508528978	0.008219962
C	-1.879032146	-1.264470000	-0.221471054
C	-0.830943009	0.889268127	0.201051976

C	-3.116324216	-0.655064918	-0.264504058
H	-1.771267168	-2.330850079	-0.363873065
C	-2.066885079	1.495022203	0.163114973
H	0.058762072	1.478520144	0.384378989
C	-3.203758183	0.720373180	-0.070787044
H	-4.010201297	-1.234921938	-0.443092071
H	-2.161451059	2.561091285	0.311662984
H	-4.173201241	1.200128243	-0.101204046
C	1.722225137	-0.426940041	0.288578983
O	2.143757171	-0.264414041	1.416754062
O	2.346804196	-0.003620028	-0.815653100
C	3.613945303	0.631915980	-0.606278083
H	4.296074335	-0.048751086	-0.102300046
H	3.984203337	0.880099988	-1.594604153
H	3.491817322	1.531539052	-0.007552039

2.2. Cyclopropanation of *aza*-Arenes 7a or 7l with Aryldiazoacetate 8a

Transition state searches for the blue light-mediated cyclopropanations of *aza*-heteroarenes **7a**, or **7l**, with the carbene **10a** derived from aryldiazoacetate **8a** were run in ORCA 6[20,21,22] and using a development version of Autobench.[23] Several transition state conformers for this step were initially explored using Autobench in combination with the GOAT[24] method in ORCA for transition state conformational searches. Despite an exhaustive conformational search at the ωB97X-3c level, no transition state corresponding to cyclopropanation events could be located. To further investigate this reaction pathway, a relaxed scan of the carbene–*aza*-heteroarene approach was performed using the ScanTS keyword in ORCA 6. This scan revealed a minimum rather than a saddle point, confirming that the cyclopropanation occurring between the carbene **10a** derived from the aryldiazoacetate **8a** and either one of the *aza*-heteroarenes, **7a** or **7l**, proceeds very rapidly, essentially without an energy barrier.

Table S5. Cartesian coordinates, electronic and Gibbs free energies in hartrees and first harmonic frequency in cm^{-1} obtained for the transition state attempts for the cyclopropanation step calculated at the ωB97X-3c level for imidazo[1,2-*a*]pyrimidine **7a** and carbene **10a**.

Electronic Energy = -191.76928296 hartrees
Enthalpy = -191.40192191 hartrees
Gibbs Free Energy = -191.46833309 hartrees
First Harmonic Frequency = -58.83 cm^{-1}

C	0.250898	-2.532162	2.924825
N	-0.832370	-2.341370	3.729079
C	-1.741224	-1.520217	3.325592

[20] F. Neese, *WIREs Comput. Mol. Sci.* **2012**, *2*: 73–78.
[21] F. Neese, *WIREs Comput. Mol. Sci.* **2018**, *8*: e1327.
[22] F. Neese, F. Wennmohs, U. Becker, C. Riplinger, *J. Chem. Phys.* **2020**, *152*, 224108.
[23] R. A. Cormanich, G. D. da Silva, *J. Chem. Inf. Model.* **2024**, *64*, 3322–3331.
[24] B. de Souza *Angew. Chem. Int. Ed.* **2025**, e202500393.

C	-1.708911	-0.789151	2.102559
C	-0.632452	-0.974493	1.308339
C	2.144334	-3.162750	2.039872
N	1.247168	-3.296446	3.138365
H	-2.594103	-1.390035	3.984546
H	-2.503673	-0.116098	1.828211
H	-0.481634	-0.473870	0.362591
C	3.170578	-4.736237	0.353466
C	3.252658	-5.275016	2.680551
C	3.871164	-5.892051	0.058797
C	3.955086	-6.428761	2.387368
C	4.270494	-6.740295	1.075577
H	4.104588	-6.128968	-0.970003
H	4.252652	-7.091822	3.188165
H	4.817764	-7.644306	0.846566
C	2.809026	-1.806430	1.780115
C	4.061812	-1.810190	0.961374
C	4.103886	-1.262343	-0.309327
C	5.220242	-2.347937	1.501685
C	5.279468	-1.265518	-1.039050
H	3.213950	-0.816363	-0.732508
C	6.397248	-2.349713	0.778334
H	5.191394	-2.786402	2.489757
C	6.428928	-1.812623	-0.497400
H	5.298498	-0.835632	-2.031094
H	7.290884	-2.780247	1.208194
H	7.347800	-1.819591	-1.067208
C	2.722826	-0.669970	2.757965
O	2.235226	-0.694642	3.850859
O	3.240956	0.439627	2.222483
C	3.219821	1.593225	3.052367
C	1.537783	-2.187137	1.060495
H	1.515059	-2.279100	-0.015709
N	0.323877	-1.828832	1.706195
C	2.864276	-4.412931	1.665000
H	2.875232	-4.081691	-0.453963
H	2.984054	-5.035834	3.699147
H	3.779659	1.411682	3.966068
H	2.197427	1.857615	3.310624
H	3.684691	2.382683	2.472591

Electronic Energy = -191.76929048 hartrees
Enthalpy = -191.40193318 hartrees
Gibbs Free Energy = -191.46835787 hartrees
First Harmonic Frequency = -58.49 cm^{-1}

C	0.259680	-2.541313	2.943959
N	-0.817808	-2.353726	3.756329
C	-1.731535	-1.533946	3.360895
C	-1.709929	-0.801469	2.138712
C	-0.639625	-0.984626	1.335476
C	2.148456	-3.165690	2.044653
N	1.260103	-3.302210	3.149757
H	-2.579491	-1.406796	4.026743
H	-2.507273	-0.128843	1.870874
H	-0.497943	-0.482371	0.389164
C	3.289416	-5.261208	2.681373
C	3.156179	-4.743625	0.351804
C	3.997973	-6.410132	2.384499
C	3.863327	-5.894611	0.053443
C	4.291280	-6.729655	1.069428

H	4.317865	-7.063131	3.184951
H	4.079187	-6.138187	-0.977635
H	4.843655	-7.629788	0.837452
C	2.806273	-1.807622	1.778588
C	4.052906	-1.808377	0.950437
C	5.218628	-2.335165	1.485833
C	4.082072	-1.269013	-0.324274
C	6.390219	-2.334701	0.753702
H	5.199931	-2.766533	2.477254
C	5.252225	-1.269943	-1.062628
H	3.186194	-0.831337	-0.743533
C	6.409033	-1.806272	-0.525860
H	7.289689	-2.756700	1.179842
H	5.261453	-0.846808	-2.057716
H	7.323588	-1.811517	-1.102587
C	2.724522	-0.670593	2.756295
O	2.246962	-0.695685	3.853506
O	3.233899	0.440428	2.214713
C	3.215406	1.594930	3.043522
C	1.530781	-2.192672	1.069208
H	1.500584	-2.285346	-0.006731
N	0.320930	-1.838141	1.724183
C	2.872440	-4.412126	1.666188
H	3.037835	-5.015869	3.702905
H	2.837602	-4.099574	-0.455277
H	3.665912	2.387156	2.456236
H	3.789592	1.418936	3.949408
H	2.194846	1.851532	3.316366

Electronic Energy = -191.77484163 hartrees
Enthalpy = -191.40722495 hartrees
Gibbs Free Energy = -191.47411736 hartrees
First Harmonic Frequency = -126.92 cm^{-1}

C	0.556779	-2.272957	3.027988
N	-0.418974	-1.937778	3.917532
C	-1.393963	-1.205797	3.486915
C	-1.523975	-0.703603	2.164550
C	-0.534523	-1.015073	1.295098
C	2.352603	-3.061789	2.042993
N	1.594045	-2.986117	3.253380
H	-2.161520	-0.960455	4.214610
H	-2.359320	-0.089479	1.873407
H	-0.494948	-0.668344	0.272638
C	3.259441	-4.807005	0.474440
C	3.409070	-5.150664	2.836441
C	3.906487	-6.009643	0.256302
C	4.058267	-6.351504	2.618212
C	4.311496	-6.784963	1.328009
H	4.094880	-6.340218	-0.755746
H	4.364856	-6.954274	3.462049
H	4.817253	-7.725515	1.158506
C	2.972109	-1.745327	1.589935
C	4.133407	-1.772996	0.649171
C	4.023248	-1.235163	-0.623102
C	5.348592	-2.303434	1.057804
C	5.105227	-1.241422	-1.486815
H	3.085129	-0.801335	-0.943481
C	6.429679	-2.308581	0.198517
H	5.438853	-2.714988	2.052064
C	6.310217	-1.782452	-1.077300

H	5.006552	-0.819895	-2.477625
H	7.370420	-2.729673	0.524669
H	7.156925	-1.791729	-1.749761
C	3.119615	-0.686434	2.644569
O	4.064509	-0.637173	3.376239
O	2.109689	0.185641	2.654321
C	2.091288	1.178981	3.676661
C	1.650614	-2.186514	1.029301
H	1.555703	-2.403284	-0.024324
N	0.467259	-1.796286	1.711577
C	3.010297	-4.364913	1.764436
H	2.959787	-4.210268	-0.375427
H	3.193920	-4.812735	3.839361
H	1.165921	1.057528	4.232768
H	2.114474	2.152958	3.196503
H	2.945651	1.062333	4.334076

Electronic Energy = -191.76863513 hartrees
Enthalpy = -191.40099003 hartrees
Gibbs Free Energy = -191.46724775 hartrees
First Harmonic Frequency = -39.81 cm^{-1}

C	0.698988	-2.222988	3.095175
N	-0.220705	-1.834267	4.021656
C	-1.150405	-1.023019	3.635444
C	-1.285140	-0.489070	2.325750
C	-0.353721	-0.861501	1.417015
C	2.408066	-3.124710	2.035186
N	1.696881	-3.002446	3.279819
H	-1.873082	-0.733541	4.392201
H	-2.079882	0.192317	2.072955
H	-0.321392	-0.502789	0.398389
C	3.974051	-4.983589	2.491962
C	2.277509	-5.314984	0.834758
C	4.437512	-6.266031	2.273590
C	2.742653	-6.600556	0.612711
C	3.826221	-7.075914	1.328816
H	5.280458	-6.636973	2.840074
H	2.255296	-7.231768	-0.117533
H	4.191774	-8.078871	1.156640
C	3.111461	-1.851104	1.609658
C	4.176039	-1.750351	0.556542
C	4.712948	-2.825893	-0.135010
C	4.606232	-0.469941	0.215036
C	5.653289	-2.624036	-1.133200
H	4.417575	-3.834411	0.097066
C	5.546862	-0.269493	-0.774424
H	4.199172	0.388742	0.731819
C	6.076948	-1.350889	-1.457626
H	6.060548	-3.480216	-1.653110
H	5.866569	0.735540	-1.012655
H	6.815273	-1.199918	-2.232750
C	3.339408	-0.885086	2.739040
O	4.272983	-0.970934	3.481434
O	2.419550	0.079183	2.818778
C	2.527012	0.938428	3.950325
C	1.733810	-2.182914	1.072760
H	1.628371	-2.333294	0.008281
N	0.597035	-1.725832	1.787884
C	2.899251	-4.499208	1.762153
H	4.446745	-4.341795	3.222926

H	1.427479	-4.943898	0.278003
H	1.709946	1.645724	3.862718
H	3.484168	1.453248	3.947233
H	2.433283	0.356709	4.863971

Electronic Energy = -191.76961194 hartrees
Enthalpy = -191.40193041 hartrees
Gibbs Free Energy = -191.46887984 hartrees
First Harmonic Frequency = -131.44 cm^{-1}

C	0.640535	-2.295365	3.138411
N	-0.300538	-1.975812	4.070584
C	-1.257672	-1.186705	3.707017
C	-1.401282	-0.607003	2.417276
C	-0.449237	-0.907938	1.503905
C	2.384585	-3.092963	2.062675
N	1.657359	-3.055956	3.296326
H	-1.997574	-0.955126	4.467159
H	-2.219028	0.053641	2.183322
H	-0.421776	-0.508648	0.500457
C	4.288520	-4.691968	2.098576
C	2.237931	-5.369039	1.065963
C	4.840469	-5.925794	1.809342
C	2.789100	-6.603485	0.771946
C	4.093727	-6.881890	1.141525
H	5.857875	-6.142130	2.104200
H	2.199163	-7.350198	0.258498
H	4.527205	-7.845842	0.913126
C	3.032735	-1.782107	1.647234
C	4.140256	-1.766047	0.637053
C	4.105712	-2.579651	-0.492592
C	5.228567	-0.914403	0.788551
C	5.117405	-2.540663	-1.432536
H	3.301135	-3.282853	-0.642727
C	6.239685	-0.873495	-0.154761
H	5.307452	-0.280492	1.657650
C	6.191953	-1.685190	-1.271665
H	5.064923	-3.192801	-2.293278
H	7.072966	-0.200488	-0.006993
H	6.984524	-1.656247	-2.006196
C	3.184404	-0.779496	2.755094
O	4.029092	-0.863568	3.598203
O	2.296864	0.214060	2.673850
C	2.282495	1.189083	3.713420
C	1.671837	-2.156723	1.112087
H	1.540395	-2.290311	0.048767
N	0.532919	-1.747668	1.851388
C	2.987564	-4.407152	1.720961
H	4.866931	-3.938010	2.614786
H	1.214575	-5.151345	0.788864
H	1.302904	1.155936	4.182445
H	2.444239	2.161861	3.258309
H	3.056146	0.977651	4.443236

Electronic Energy = -191.76046065 hartrees
Enthalpy = -191.39250726 hartrees
Gibbs Free Energy = -191.45817646 hartrees
First Harmonic Frequency = -44.91 cm^{-1}

C	0.459896	-2.059954	2.994212
N	-0.529320	-1.699247	3.852123
C	-1.574494	-1.125514	3.347463

C	-1.766927	-0.835173	1.972740
C	-0.769358	-1.184999	1.128687
C	2.331775	-2.812805	2.092156
N	1.574466	-2.620208	3.297194
H	-2.350506	-0.847598	4.053320
H	-2.650777	-0.330913	1.620826
H	-0.771763	-0.984397	0.067790
C	4.205005	-4.358041	1.299537
C	2.460212	-5.194937	2.698033
C	4.773323	-5.620774	1.263483
C	3.027117	-6.453706	2.660406
C	4.187731	-6.674296	1.938930
H	5.685684	-5.774431	0.703743
H	2.561765	-7.265252	3.202782
H	4.635107	-7.658231	1.909814
C	2.760198	-1.513606	1.414446
C	3.867358	-1.374294	0.406579
C	3.679772	-1.650401	-0.940732
C	5.114998	-0.905862	0.806293
C	4.712900	-1.511964	-1.849897
H	2.721087	-1.981250	-1.306640
C	6.148060	-0.762538	-0.100098
H	5.291661	-0.638430	1.835815
C	5.956333	-1.075597	-1.433644
H	4.538390	-1.741678	-2.891856
H	7.108017	-0.400364	0.240659
H	6.764213	-0.966845	-2.143469
C	2.565497	-0.220023	2.182298
O	1.843686	0.635593	1.754070
O	3.265240	-0.000389	3.288791
C	3.950329	-1.010193	4.021489
C	1.503194	-2.207493	0.980556
H	1.404685	-2.636037	-0.002387
N	0.303520	-1.812316	1.628165
C	3.036169	-4.130239	2.012512
H	4.694015	-3.559883	0.767952
H	1.568055	-5.018037	3.279988
H	4.764405	-0.503463	4.530620
H	4.348696	-1.790025	3.379578
H	3.267624	-1.456532	4.737134

Electronic Energy = -191.76929682 hartrees
Enthalpy = -191.40193677 hartrees
Gibbs Free Energy = -191.46835444 hartrees
First Harmonic Frequency = -58.29 cm^{-1}

C	0.284161	-2.550358	2.983226
N	-0.783258	-2.365267	3.809359
C	-1.712351	-1.561155	3.417579
C	-1.717436	-0.843615	2.186349
C	-0.655985	-1.022429	1.370536
C	2.165455	-3.166061	2.062493
N	1.294734	-3.299748	3.181801
H	-2.551856	-1.435482	4.094319
H	-2.527430	-0.184881	1.921963
H	-0.533583	-0.529875	0.416472
C	3.171654	-4.745164	0.369323
C	3.318506	-5.254952	2.699811
C	3.881109	-5.894661	0.070939
C	4.029691	-6.402277	2.402849
C	4.317215	-6.725012	1.087322

H	4.092484	-6.140758	-0.960469
H	4.356150	-7.051622	3.203625
H	4.871447	-7.624017	0.855370
C	2.806889	-1.804230	1.774461
C	4.041094	-1.800243	0.928020
C	4.047388	-1.266789	-0.349465
C	5.218699	-2.315360	1.448642
C	5.206517	-1.262408	-1.105045
H	3.142156	-0.838081	-0.757802
C	6.379380	-2.309390	0.699385
H	5.217729	-2.742466	2.442098
C	6.375261	-1.787106	-0.582852
H	5.197602	-0.844141	-2.102184
H	7.288261	-2.722359	1.114254
H	7.281293	-1.788115	-1.172900
C	2.728103	-0.659080	2.742686
O	2.267350	-0.678934	3.847224
O	3.217864	0.451929	2.183541
C	3.201896	1.613376	3.002602
C	1.524910	-2.207951	1.087324
H	1.480025	-2.311172	0.012829
N	0.321259	-1.858244	1.756277
C	2.893305	-4.410470	1.684170
H	2.846931	-4.104597	-0.438047
H	3.071355	-5.007289	3.721821
H	2.181985	1.872297	3.275862
H	3.650633	2.400602	2.407284
H	3.778448	1.445214	3.908408

Table S6. Cartesian coordinates, electronic and Gibbs free energies in hartrees and first harmonic frequency in cm^{-1} obtained for the transition state attempts for the cyclopropanation step calculated at the ωB97X-3c level for imidazo[1,2-*a*]pyridine **7l** and carbene **10a**.

Electronic Energy = -187.99407180 hartrees
Enthalpy = -187.61724410 hartrees
Gibbs Free Energy = -187.68591066 hartrees
First Harmonic Frequency = -238.85 cm^{-1}

C	0.995323	-1.875601	0.377134
C	-0.075769	-1.135425	-0.237137
C	0.172589	-0.377087	-1.317217
C	1.494123	-0.269375	-1.875636
C	2.500291	-0.958178	-1.316601
C	2.351561	-3.068928	1.672088
N	0.978199	-2.602259	1.419880
H	-1.054276	-1.210067	0.211402
H	-0.632156	0.180252	-1.776625
H	1.681240	0.356561	-2.733317
H	3.521640	-0.921546	-1.664829
C	3.368852	-5.170054	2.584445
C	1.606051	-5.366504	0.982635
C	3.487886	-6.548361	2.624689
C	1.726306	-6.743825	1.016581
C	2.670090	-7.339405	1.836862
H	4.216076	-7.006697	3.280101
H	1.074950	-7.356054	0.407738
H	2.760124	-8.416636	1.868791
C	3.079738	-2.216115	2.653334
C	3.951620	-1.320291	1.766570

C	3.478601	0.017884	1.683144
C	5.383328	-1.475223	1.716114
C	4.334765	1.103621	1.623259
H	2.409750	0.178265	1.706194
C	6.213475	-0.416325	1.547025
H	5.782171	-2.478442	1.798272
C	5.688956	0.890046	1.529081
H	3.937873	2.108392	1.627538
H	7.282279	-0.564727	1.483483
H	6.363655	1.731386	1.446739
C	2.570171	-1.699671	3.884493
O	2.892305	-0.657652	4.427355
O	1.731221	-2.580650	4.469626
C	1.228343	-2.205669	5.735599
H	0.555605	-3.002301	6.036487
H	2.034475	-2.100340	6.458842
H	0.690533	-1.261767	5.673418
C	3.181356	-2.379204	0.588237
H	4.002131	-2.887742	0.101383
N	2.254503	-1.771608	-0.246565
C	2.432341	-4.569477	1.761020
H	3.989250	-4.540038	3.207743
H	0.850907	-4.903329	0.362897[1]

Electronic Energy = -187.99406878 hartrees
Enthalpy = -187.61723849 hartrees
Gibbs Free Energy = -187.68589947 hartrees
First Harmonic Frequency = -236.97 cm^{-1}

C	0.984892	-1.879145	0.383455
C	-0.089669	-1.140800	-0.226877
C	0.152892	-0.384383	-1.309654
C	1.471543	-0.276942	-1.874843
C	2.480819	-0.964318	-1.319636
C	2.348315	-3.068482	1.674214
N	0.973268	-2.603863	1.427665
H	-1.065881	-1.215393	0.226641
H	-0.654366	0.171544	-1.766371
H	1.653800	0.347692	-2.734527
H	3.500495	-0.928198	-1.672795
C	1.603122	-5.369765	0.998220
C	3.370489	-5.164189	2.593822
C	1.724812	-6.746754	1.038096
C	3.491069	-6.542225	2.639956
C	2.671773	-7.337645	1.858184
H	1.072163	-7.362437	0.434151
H	4.221689	-6.996792	3.295297
H	2.763053	-8.414630	1.894662
C	3.081553	-2.210020	2.646194
C	3.953341	-1.322516	1.750970
C	3.485099	0.017049	1.666286
C	5.384528	-1.483035	1.696618
C	4.345435	1.099842	1.605101
H	2.416948	0.181866	1.689822
C	6.218043	-0.427405	1.525401
H	5.780028	-2.487619	1.778292
C	5.698388	0.881295	1.508561
H	3.951779	2.105892	1.609767
H	7.286124	-0.579911	1.459368
H	6.376260	1.719950	1.424884
C	2.581674	-1.688639	3.879061

O	2.910885	-0.646381	4.417179
O	1.743637	-2.565395	4.471752
C	1.250316	-2.185563	5.740037
H	0.576234	-2.978792	6.046814
H	2.061334	-2.081899	6.458047
H	0.716102	-1.239532	5.679161
C	3.172422	-2.383052	0.583251
H	3.988396	-2.894585	0.091363
N	2.240668	-1.775720	-0.246796
C	2.431014	-4.568365	1.770421
H	0.845617	-4.910058	0.378809
H	3.992347	-4.530854	3.212249

Electronic Energy = -187.98833273 hartrees
Enthalpy = -187.61132149 hartrees
Gibbs Free Energy = -187.67934014 hartrees
First Harmonic Frequency = -581.16 cm^{-1}

C	0.662058	-1.995727	0.002301
C	-0.666036	-1.740391	-0.374148
C	-1.006881	-0.539842	-0.920531
C	-0.030915	0.450374	-1.116966
C	1.240329	0.202924	-0.713111
C	2.087269	-3.335227	1.238098
N	1.049788	-3.204902	0.440434
H	-1.386190	-2.529488	-0.223638
H	-2.031639	-0.349086	-1.206139
H	-0.275592	1.401572	-1.562519
H	2.047453	0.914913	-0.785743
C	1.871079	-5.809056	1.263099
C	3.657783	-4.751709	2.479385
C	2.322756	-7.043844	1.676618
C	4.107032	-5.991916	2.892962
C	3.441581	-7.136940	2.492853
H	1.804560	-7.940412	1.365936
H	4.979191	-6.064152	3.527426
H	3.793574	-8.107130	2.816157
C	2.717098	-2.072146	1.626564
C	4.222149	-1.164117	1.473197
C	4.473917	-0.023884	2.255785
C	5.333894	-1.923403	1.058584
C	5.767905	0.323017	2.584891
H	3.655725	0.599187	2.578566
C	6.616298	-1.551294	1.396230
H	5.175673	-2.821424	0.475418
C	6.857898	-0.423997	2.166072
H	5.924150	1.216504	3.175701
H	7.441100	-2.166457	1.060440
H	7.864769	-0.135900	2.430299
C	2.028925	-1.433739	2.757043
O	1.565908	-0.318306	2.767063
O	1.938189	-2.261288	3.804158
C	1.224035	-1.771317	4.928127
H	1.217173	-2.580727	5.649841
H	1.719475	-0.897260	5.342994
H	0.208093	-1.503972	4.647528
C	2.866957	-1.312590	0.388829
H	3.399545	-1.840809	-0.392046
N	1.567569	-0.974489	-0.143773
C	2.536382	-4.653515	1.663999
H	0.999910	-5.717070	0.631249

H	4.172512	-3.851137	2.783041

Electronic Energy = -187.99406886 hartrees
Enthalpy = -187.61723861 hartrees
Gibbs Free Energy = -187.68591661 hartrees
First Harmonic Frequency = -237.75 cm^{-1}

C	0.962279	-1.899803	0.410515
C	-0.125423	-1.172014	-0.188994
C	0.096191	-0.423990	-1.282068
C	1.404977	-0.315699	-1.869471
C	2.426252	-0.993593	-1.324576
C	2.351764	-3.070549	1.689735
N	0.971252	-2.614823	1.461434
H	-1.093971	-1.247050	0.280595
H	-0.720996	0.124307	-1.730254
H	1.570704	0.301737	-2.737642
H	3.439735	-0.956070	-1.694991
C	1.608738	-5.382441	1.047457
C	3.397467	-5.151881	2.615577
C	1.737916	-6.758378	1.099694
C	3.525412	-6.528725	2.674352
C	2.699098	-7.336166	1.912500
H	1.080054	-7.383311	0.511090
H	4.267344	-6.973026	3.323992
H	2.796132	-8.412259	1.958980
C	3.098173	-2.199729	2.641122
C	3.953011	-1.318058	1.724315
C	3.481439	0.019739	1.628757
C	5.382967	-1.477371	1.646925
C	4.338467	1.101988	1.531195
H	2.413668	0.183263	1.671848
C	6.211705	-0.423736	1.441451
H	5.781225	-2.479988	1.738737
C	5.689879	0.883391	1.411339
H	3.943876	2.107667	1.526350
H	7.278635	-0.575941	1.358376
H	6.364449	1.721375	1.299197
C	2.613384	-1.662250	3.873313
O	2.949864	-0.612960	4.393089
O	1.783439	-2.530780	4.488831
C	1.307769	-2.134593	5.758819
H	2.127190	-2.039051	6.468503
H	0.787978	-1.180751	5.696609
H	0.624571	-2.915226	6.077546
C	3.155162	-2.392129	0.579173
H	3.965002	-2.903985	0.077661
N	2.207303	-1.796235	-0.240748
C	2.443411	-4.569092	1.799572
H	0.840005	-4.933116	0.434220
H	4.024953	-4.508867	3.218150

Electronic Energy = -187.98846628 hartrees
Enthalpy = -187.61178713 hartrees
Gibbs Free Energy = -187.68019869 hartrees
First Harmonic Frequency = -226.63 cm^{-1}

C	0.918967	-1.739946	0.033712
C	-0.022982	-1.121099	-0.867015
C	0.420501	-0.464570	-1.950594
C	1.823176	-0.360830	-2.257132
C	2.711148	-0.943823	-1.437946

C	2.017902	-2.772361	1.647864
N	0.709130	-2.338611	1.131389
H	-1.070336	-1.193302	-0.618190
H	-0.290674	0.007769	-2.614546
H	2.160941	0.172393	-3.130911
H	3.780548	-0.904751	-1.583841
C	3.281999	-4.888375	2.142139
C	0.960426	-5.062395	1.623665
C	3.364454	-6.259812	2.273496
C	1.037671	-6.438842	1.764543
C	2.237483	-7.044223	2.084959
H	4.307900	-6.718533	2.536418
H	0.149415	-7.039581	1.623214
H	2.296389	-8.118334	2.194665
C	2.660319	-1.907455	2.677853
C	3.557129	-0.970408	1.858447
C	3.104420	0.351977	1.516236
C	4.952123	-1.099672	2.090316
C	3.966765	1.390514	1.376284
H	2.044154	0.497348	1.361635
C	5.819020	-0.024667	2.023557
H	5.333715	-2.079093	2.341025
C	5.333026	1.207028	1.650951
H	3.602399	2.370027	1.100959
H	6.867902	-0.156236	2.245785
H	6.007611	2.048911	1.570440
C	3.063854	-2.237215	3.994665
O	4.001365	-1.754653	4.615604
O	2.232914	-3.143123	4.558689
C	2.533437	-3.513810	5.887992
H	3.550810	-3.892200	5.965743
H	2.425080	-2.666000	6.561631
H	1.822280	-4.291733	6.147273
C	3.034851	-2.188629	0.688617
H	3.912815	-2.751539	0.404975
N	2.276412	-1.641866	-0.350012
C	2.081961	-4.273279	1.808548
H	4.161560	-4.286398	2.331059
H	0.022471	-4.588772	1.378110

Electronic Energy = -187.98847340 hartrees
Enthalpy = -187.61180003 hartrees
Gibbs Free Energy = -187.68027467 hartrees
First Harmonic Frequency = -239.36 cm^{-1}

C	0.883280	-1.717093	0.085414
C	-0.077771	-1.085029	-0.785622
C	0.341673	-0.418137	-1.872304
C	1.737173	-0.314560	-2.210983
C	2.643144	-0.906100	-1.418277
C	2.017695	-2.771149	1.660265
N	0.697448	-2.333325	1.177320
H	-1.119165	-1.157532	-0.512973
H	-0.383677	0.063156	-2.514109
H	2.055313	0.225898	-3.087679
H	3.708914	-0.867019	-1.588536
C	3.287405	-4.888570	2.137001
C	0.976381	-5.067904	1.575173
C	3.378180	-6.262054	2.237981
C	1.061828	-6.446698	1.685665
C	2.260755	-7.050347	2.012301

H	4.320524	-6.719730	2.506618
H	0.180672	-7.050460	1.515703
H	2.326155	-8.126224	2.098520
C	2.679604	-1.916911	2.686453
C	3.570588	-0.978688	1.865648
C	3.126303	0.351088	1.543881
C	4.968649	-1.125477	2.066847
C	3.997574	1.380724	1.390637
H	2.064510	0.509462	1.414354
C	5.846577	-0.060687	1.987208
H	5.343815	-2.110794	2.304050
C	5.366984	1.179282	1.633069
H	3.638696	2.366297	1.130133
H	6.898460	-0.205776	2.185599
H	6.049643	2.013659	1.542860
C	3.092288	-2.254228	3.997939
O	4.036410	-1.777032	4.613168
O	2.264487	-3.161703	4.564627
C	2.575316	-3.537640	5.890039
H	3.580857	-3.949699	5.951652
H	2.507023	-2.684930	6.562500
H	1.843874	-4.291282	6.164128
C	3.013370	-2.178143	0.683894
H	3.883193	-2.738015	0.370517
N	2.232734	-1.611756	-0.325531
C	2.088362	-4.274701	1.797219
H	4.158696	-4.284274	2.354080
H	0.038506	-4.596071	1.325824

Electronic Energy = -187.99070290 hartrees
Enthalpy = -187.61401749 hartrees
Gibbs Free Energy = -187.68279855 hartrees
First Harmonic Frequency = -302.03 cm^{-1}

C	0.690164	-2.142923	0.696232
C	-0.516305	-1.473414	0.283413
C	-0.512881	-0.723061	-0.829539
C	0.678097	-0.553699	-1.620543
C	1.805944	-1.179746	-1.254930
C	2.323459	-3.252202	1.725766
N	0.907382	-2.839899	1.736625
H	-1.390372	-1.595508	0.904306
H	-1.418174	-0.219898	-1.140088
H	0.669976	0.068679	-2.500992
H	2.741591	-1.094200	-1.786777
C	3.530540	-5.338355	2.411034
C	1.584303	-5.551615	1.040298
C	3.705848	-6.710579	2.376656
C	1.761612	-6.922361	0.997205
C	2.825278	-7.506206	1.664901
H	4.527917	-7.161156	2.916005
H	1.061630	-7.538822	0.449760
H	2.959921	-8.578946	1.639332
C	3.169206	-2.398381	2.600126
C	3.720631	-1.363741	1.619196
C	3.044364	-0.113062	1.702909
C	5.119403	-1.291086	1.284952
C	3.703624	1.094394	1.549146
H	1.990035	-0.125387	1.942296
C	5.733298	-0.109899	1.022642
H	5.670741	-2.221352	1.232479

C	5.030059	1.098135	1.187159
H	3.172716	2.025366	1.687881
H	6.777988	-0.087421	0.745870
H	5.544321	2.036789	1.031058
C	2.931244	-2.142029	3.982893
O	2.360981	-2.899344	4.739174
O	3.528339	-1.001568	4.430396
C	3.483424	-0.806260	5.829534
H	2.455023	-0.755123	6.181146
H	3.986915	-1.617345	6.352170
H	3.992599	0.133779	6.018697
C	2.898267	-2.499729	0.523824
H	3.694095	-2.920031	-0.074812
N	1.806406	-1.993688	-0.155450
C	2.472851	-4.749672	1.740944
H	4.203506	-4.708528	2.976459
H	0.736484	-5.101076	0.542916

Electronic Energy = -187.99072477 hartrees
Enthalpy = -187.61405163 hartrees
Gibbs Free Energy = -187.68287891 hartrees
First Harmonic Frequency = -291.22 cm^{-1}

C	0.953935	-2.103858	0.238644
C	-0.119197	-1.413484	-0.428750
C	0.151428	-0.615743	-1.474014
C	1.498382	-0.415815	-1.940620
C	2.506939	-1.058972	-1.334373
C	2.292133	-3.257847	1.591191
N	0.913757	-2.849616	1.266987
H	-1.117883	-1.559095	-0.046910
H	-0.654039	-0.096665	-1.975017
H	1.701908	0.242923	-2.769615
H	3.544002	-0.952820	-1.615230
C	1.759734	-5.539511	0.689985
C	3.325376	-5.357962	2.486704
C	1.956626	-6.907797	0.653464
C	3.518559	-6.727890	2.457904
C	2.839112	-7.506274	1.537259
H	1.411563	-7.511216	-0.059630
H	4.194705	-7.190590	3.163913
H	2.987298	-8.577302	1.515417
C	2.890812	-2.428796	2.669727
C	3.695430	-1.386567	1.889581
C	3.048976	-0.119113	1.840324
C	5.135004	-1.347885	1.922653
C	3.752555	1.071867	1.905118
H	1.968669	-0.104881	1.796194
C	5.821327	-0.179286	1.868834
H	5.660852	-2.292714	1.977275
C	5.126713	1.045304	1.893636
H	3.224481	2.014205	1.936033
H	6.902307	-0.180885	1.867263
H	5.684924	1.971638	1.906992
C	2.302789	-2.191291	3.948258
O	1.546424	-2.953226	4.511633
O	2.775052	-1.070320	4.561343
C	2.369525	-0.901563	5.904969
H	2.708299	-1.732274	6.521010
H	2.824338	0.024727	6.242290
H	1.285777	-0.837708	5.977515

C	3.142215	-2.465973	0.594460
H	4.052344	-2.875513	0.178888
N	2.243671	-1.917112	-0.302756
C	2.449662	-4.754452	1.601619
H	1.048395	-5.077461	0.019480
H	3.837572	-4.741360	3.212623

Electronic Energy = -187.98386559 hartrees
Enthalpy = -187.60701551 hartrees
Gibbs Free Energy = -187.67508232 hartrees
First Harmonic Frequency = -580.36 cm^{-1}

C	0.674479	-2.126487	0.115068
C	-0.669495	-1.903564	-0.229358
C	-1.077375	-0.673317	-0.647776
C	-0.155688	0.382921	-0.745535
C	1.129650	0.162910	-0.371726
C	2.169361	-3.515220	1.210904
N	1.124941	-3.351102	0.426346
H	-1.345640	-2.741465	-0.159727
H	-2.112731	-0.509463	-0.911232
H	-0.452217	1.358067	-1.098514
H	1.899847	0.918560	-0.379964
C	3.781044	-4.973957	2.341207
C	2.052772	-5.985980	1.005631
C	4.275372	-6.228254	2.646169
C	2.550746	-7.234202	1.309735
C	3.663189	-7.356660	2.131185
H	5.139348	-6.323987	3.288467
H	2.073142	-8.118501	0.911303
H	4.050531	-8.337728	2.370363
C	2.746380	-2.272446	1.721308
C	4.205068	-1.281285	1.645140
C	4.416338	-0.232558	2.558161
C	5.348448	-1.928891	1.135867
C	5.695958	0.140477	2.914419
H	3.578383	0.312926	2.960155
C	6.615052	-1.535928	1.504863
H	5.225926	-2.759749	0.453131
C	6.813952	-0.495436	2.400091
H	5.816722	0.962975	3.607634
H	7.463783	-2.066692	1.093329
H	7.809352	-0.192324	2.689301
C	2.115617	-1.886019	2.997201
O	2.120137	-2.570396	3.986275
O	1.475994	-0.703725	2.944187
C	0.887677	-0.270796	4.163890
H	1.641416	-0.191872	4.943823
H	0.450760	0.699944	3.955448
H	0.121965	-0.972574	4.484844
C	2.839659	-1.374527	0.576555
H	3.380808	-1.803079	-0.258643
N	1.520921	-1.046571	0.077731
C	2.666505	-4.846699	1.520446
H	4.250065	-4.086281	2.740716
H	1.184286	-5.871215	0.373853

Electronic Energy = -187.99073241 hartrees
Enthalpy = -187.61405482 hartrees
Gibbs Free Energy = -187.68285585 hartrees
First Harmonic Frequency = -293.79 cm^{-1}

C	0.951612	-2.158991	0.245708
C	-0.132029	-1.496250	-0.432728
C	0.124564	-0.726525	-1.502194
C	1.466498	-0.529768	-1.984554
C	2.484411	-1.148720	-1.369009
C	2.308944	-3.263657	1.620236
N	0.925391	-2.872772	1.297041
H	-1.126613	-1.638177	-0.038971
H	-0.688552	-0.227829	-2.011464
H	1.658737	0.107462	-2.832835
H	3.518443	-1.042978	-1.660953
C	3.395936	-5.327836	2.532744
C	1.756545	-5.576732	0.811577
C	3.600440	-6.696243	2.537674
C	1.963915	-6.944097	0.809149
C	2.888925	-7.508121	1.671670
H	4.310479	-7.131526	3.227667
H	1.393834	-7.573566	0.139449
H	3.045483	-8.578166	1.676505
C	2.911438	-2.404141	2.673431
C	3.685968	-1.365184	1.858167
C	3.011900	-0.113514	1.783555
C	5.124328	-1.294902	1.874588
C	3.690393	1.093315	1.808384
H	1.931102	-0.123058	1.753436
C	5.785054	-0.113807	1.782487
H	5.671030	-2.226497	1.948481
C	5.064679	1.095822	1.782304
H	3.142443	2.024674	1.820716
H	6.865744	-0.092698	1.769162
H	5.602828	2.033889	1.765170
C	2.326099	-2.139687	3.948863
O	1.579371	-2.893887	4.534682
O	2.791325	-0.999804	4.531492
C	2.389522	-0.800342	5.872023
H	2.834822	0.139787	6.182667
H	1.305429	-0.747485	5.947931
H	2.740942	-1.610441	6.508162
C	3.143864	-2.491568	0.596772
H	4.058689	-2.900924	0.191589
N	2.235462	-1.980763	-0.312529
C	2.477579	-4.758516	1.668082
H	3.932435	-4.684872	3.217173
H	1.013038	-5.141080	0.158770

Electronic Energy = -187.98420780 hartrees
Enthalpy = -187.60771726 hartrees
Gibbs Free Energy = -187.67635302 hartrees
First Harmonic Frequency = -251.27 cm^{-1}

C	0.577108	-1.795532	0.357408
C	-0.482454	-1.113768	-0.346774
C	-0.198746	-0.377160	-1.432235
C	1.144299	-0.246737	-1.935043
C	2.139452	-0.884016	-1.300106
C	1.892705	-2.950254	1.706086
N	0.524518	-2.482723	1.420159
H	-1.482392	-1.209235	0.047103
H	-0.996593	0.140289	-1.947444
H	1.352755	0.347667	-2.809750
H	3.175366	-0.832344	-1.601374

C	0.848278	-5.238302	1.504269
C	3.187420	-5.091620	1.944155
C	0.943142	-6.620199	1.484819
C	3.287318	-6.467865	1.915210
C	2.161296	-7.241031	1.683785
H	0.054481	-7.213166	1.316250
H	4.243669	-6.940776	2.091888
H	2.233907	-8.319820	1.671334
C	2.668412	-2.163107	2.699511
C	3.472329	-1.184460	1.826721
C	3.033035	0.175848	1.659177
C	4.881015	-1.375667	1.838678
C	3.912316	1.199063	1.512022
H	1.968162	0.363865	1.652870
C	5.775424	-0.323606	1.777690
H	5.248403	-2.389177	1.924963
C	5.293951	0.953139	1.598101
H	3.556266	2.210607	1.377879
H	6.838685	-0.504421	1.841625
H	5.985215	1.781412	1.517603
C	3.092197	-2.589148	3.980284
O	2.571787	-3.477116	4.626747
O	4.120338	-1.847234	4.494031
C	4.434038	-2.112631	5.845981
H	3.582712	-1.902718	6.490806
H	4.721266	-3.153200	5.982473
H	5.262901	-1.457525	6.097161
C	2.764029	-2.290740	0.651886
H	3.576881	-2.835212	0.191167
N	1.866329	-1.655823	-0.209740
C	1.970169	-4.459581	1.729288
H	-0.104932	-4.754458	1.357477
H	4.065992	-4.501692	2.168130

Electronic Energy = -187.98421278 hartrees
Enthalpy = -187.60771523 hartrees
Gibbs Free Energy = -187.67649000 hartrees
First Harmonic Frequency = -262.74 cm^{-1}

C	0.579774	-1.758384	0.388838
C	-0.472280	-1.052089	-0.302773
C	-0.184978	-0.322548	-1.391811
C	1.154861	-0.223374	-1.911127
C	2.142925	-0.883196	-1.288752
C	1.885620	-2.940394	1.724331
N	0.524143	-2.443844	1.452139
H	-1.469151	-1.123995	0.103730
H	-0.976778	0.213240	-1.897504
H	1.366030	0.365866	-2.788695
H	3.175786	-0.855717	-1.603350
C	0.816804	-5.204395	1.410749
C	3.120716	-5.111269	2.020158
C	0.888669	-6.586953	1.364147
C	3.198018	-6.488435	1.964800
C	2.078773	-7.234843	1.634511
H	0.004316	-7.158929	1.118173
H	4.130585	-6.983329	2.199064
H	2.133551	-8.314272	1.601460
C	2.684806	-2.169575	2.710349
C	3.502810	-1.211316	1.833621
C	3.093348	0.157629	1.665988

C	4.906966	-1.435816	1.826531
C	3.994011	1.159227	1.496354
H	2.033149	0.370429	1.678056
C	5.824780	-0.406559	1.741332
H	5.250083	-2.457664	1.916084
C	5.369963	0.880289	1.560048
H	3.660124	2.178316	1.362455
H	6.884487	-0.611474	1.789508
H	6.079385	1.691171	1.461717
C	3.091316	-2.576041	4.000562
O	2.558866	-3.445741	4.662782
O	4.128954	-1.837503	4.503846
C	4.438183	-2.085052	5.859913
H	5.270153	-1.430996	6.103690
H	3.586786	-1.861640	6.500072
H	4.719776	-3.125029	6.012515
C	2.758075	-2.300725	0.657278
H	3.556344	-2.861406	0.190640
N	1.865869	-1.648634	-0.194332
C	1.932945	-4.451715	1.734400
H	-0.116211	-4.700646	1.210318
H	3.990097	-4.542840	2.320454

Electronic Energy = -187.98607279 hartrees
Enthalpy = -187.60908224 hartrees
Gibbs Free Energy = -187.67623636 hartrees
First Harmonic Frequency = -252.26 cm^{-1}

C	0.813222	-1.754180	0.613900
C	-0.293988	-1.045794	0.033884
C	-0.094004	-0.264739	-1.041391
C	1.208419	-0.103739	-1.626483
C	2.253321	-0.747613	-1.084017
C	2.235557	-2.906120	1.856256
N	0.839665	-2.537400	1.616352
H	-1.260818	-1.163341	0.497816
H	-0.928001	0.268222	-1.476788
H	1.352945	0.528097	-2.487954
H	3.266444	-0.670667	-1.449023
C	1.567039	-5.270942	1.335113
C	3.508261	-4.912915	2.680248
C	1.782889	-6.636724	1.387469
C	3.726535	-6.278134	2.734752
C	2.864859	-7.145240	2.085337
H	1.098764	-7.307861	0.886245
H	4.566886	-6.666903	3.293742
H	3.030921	-8.212804	2.129810
C	2.944108	-1.954547	2.784591
C	3.954875	-1.233593	1.866128
C	3.722802	0.144178	1.621731
C	5.335970	-1.640468	1.851000
C	4.755093	1.040841	1.401924
H	2.702313	0.493514	1.598835
C	6.338046	-0.776220	1.551243
H	5.558415	-2.679216	2.056703
C	6.050010	0.585198	1.345659
H	4.536216	2.088673	1.256002
H	7.362550	-1.119130	1.516133
H	6.857866	1.275806	1.144898
C	2.342714	-1.130734	3.803570
O	2.643179	0.030769	4.014146

O	1.447258	-1.687814	4.643473
C	1.269475	-3.085466	4.728214
H	0.659769	-3.455078	3.907402
H	2.227995	-3.599631	4.741312
H	0.754020	-3.263799	5.667736
C	3.024773	-2.225036	0.746990
H	3.775556	-2.751885	0.172667
N	2.060110	-1.555649	-0.000132
C	2.429725	-4.397231	1.979856
H	0.710511	-4.874873	0.808113
H	4.160185	-4.229981	3.208810

Electronic Energy = -187.99073483 hartrees
Enthalpy = -187.61405271 hartrees
Gibbs Free Energy = -187.68283744 hartrees
First Harmonic Frequency = -294.13 cm^{-1}

C	1.030235	-2.040195	0.174078
C	0.006308	-1.310877	-0.528467
C	0.343135	-0.512345	-1.553586
C	1.713431	-0.349847	-1.963669
C	2.676556	-1.030179	-1.325249
C	2.277049	-3.249380	1.565516
N	0.926344	-2.791707	1.193842
H	-1.010941	-1.429003	-0.188421
H	-0.425204	0.035871	-2.081162
H	1.970172	0.310495	-2.776475
H	3.726819	-0.953609	-1.563347
C	1.676864	-5.503598	0.633651
C	3.242838	-5.390534	2.435558
C	1.833776	-6.876673	0.580951
C	3.396502	-6.764827	2.389965
C	2.696589	-7.511654	1.458395
H	1.272599	-7.455034	-0.140357
H	4.057356	-7.255736	3.091398
H	2.813530	-8.586196	1.423467
C	2.858790	-2.458156	2.681405
C	3.704362	-1.411965	1.948474
C	3.075069	-0.136461	1.893280
C	5.141312	-1.391006	2.035218
C	3.789922	1.044842	1.996392
H	1.997114	-0.107824	1.813818
C	5.843544	-0.230268	2.021354
H	5.653793	-2.342676	2.097597
C	5.163403	1.001992	2.034709
H	3.271991	1.993021	2.019841
H	6.923723	-0.245595	2.060009
H	5.731446	1.921338	2.079625
C	2.224941	-2.221010	3.938948
O	1.429767	-2.971576	4.462615
O	2.698348	-1.119127	4.584534
C	2.244080	-0.957196	5.913343
H	2.703631	-0.044099	6.279019
H	1.159818	-0.872350	5.944579
H	2.542175	-1.801470	6.531984
C	3.188392	-2.468727	0.616503
H	4.109100	-2.894527	0.243098
N	2.345753	-1.890921	-0.315133
C	2.387174	-4.750384	1.556179
H	0.980822	-5.012836	-0.032225

H	3.770022	-4.798160	3.171015

Electronic Energy = -187.98172409 hartrees
Enthalpy = -187.60493598 hartrees
Gibbs Free Energy = -187.67311389 hartrees
First Harmonic Frequency = -324.75 cm^{-1}

C	0.726861	-1.560498	1.051589
C	-0.434535	-0.801246	0.655748
C	-0.414597	-0.096127	-0.484891
C	0.742774	-0.070593	-1.344359
C	1.830340	-0.771760	-0.998931
C	2.296900	-2.786113	2.019041
N	0.924153	-2.248095	2.096500
H	-1.287934	-0.817823	1.315794
H	-1.284965	0.477241	-0.773067
H	0.738275	0.503204	-2.256997
H	2.742054	-0.796568	-1.576793
C	1.121066	-5.004356	1.739374
C	3.474826	-5.010909	2.149486
C	1.132284	-6.385844	1.655793
C	3.486907	-6.390731	2.061988
C	2.315241	-7.084808	1.814373
H	0.208842	-6.917735	1.471190
H	4.415541	-6.926088	2.204260
H	2.323382	-8.164482	1.755356
C	3.338908	-2.048838	2.795014
C	3.968043	-1.129643	1.766867
C	3.550155	0.235850	1.655168
C	5.305567	-1.399360	1.370020
C	4.348707	1.192589	1.105989
H	2.557281	0.484773	2.004936
C	6.135559	-0.422622	0.859542
H	5.662202	-2.414511	1.468511
C	5.652461	0.861383	0.715718
H	4.000308	2.210945	1.009344
H	7.151522	-0.659447	0.579150
H	6.292593	1.630129	0.303162
C	4.164466	-2.429292	3.875208
O	5.330901	-2.079580	4.011459
O	3.639410	-3.220820	4.847331
C	2.243809	-3.260920	5.064170
H	1.779111	-4.066365	4.498570
H	2.101601	-3.447954	6.125865
H	1.777975	-2.317372	4.787671
C	2.868837	-2.186020	0.748954
H	3.537816	-2.742518	0.110067
N	1.821490	-1.517172	0.148741
C	2.292004	-4.301926	1.986558
H	0.192880	-4.463050	1.633242
H	4.391428	-4.485093	2.375561

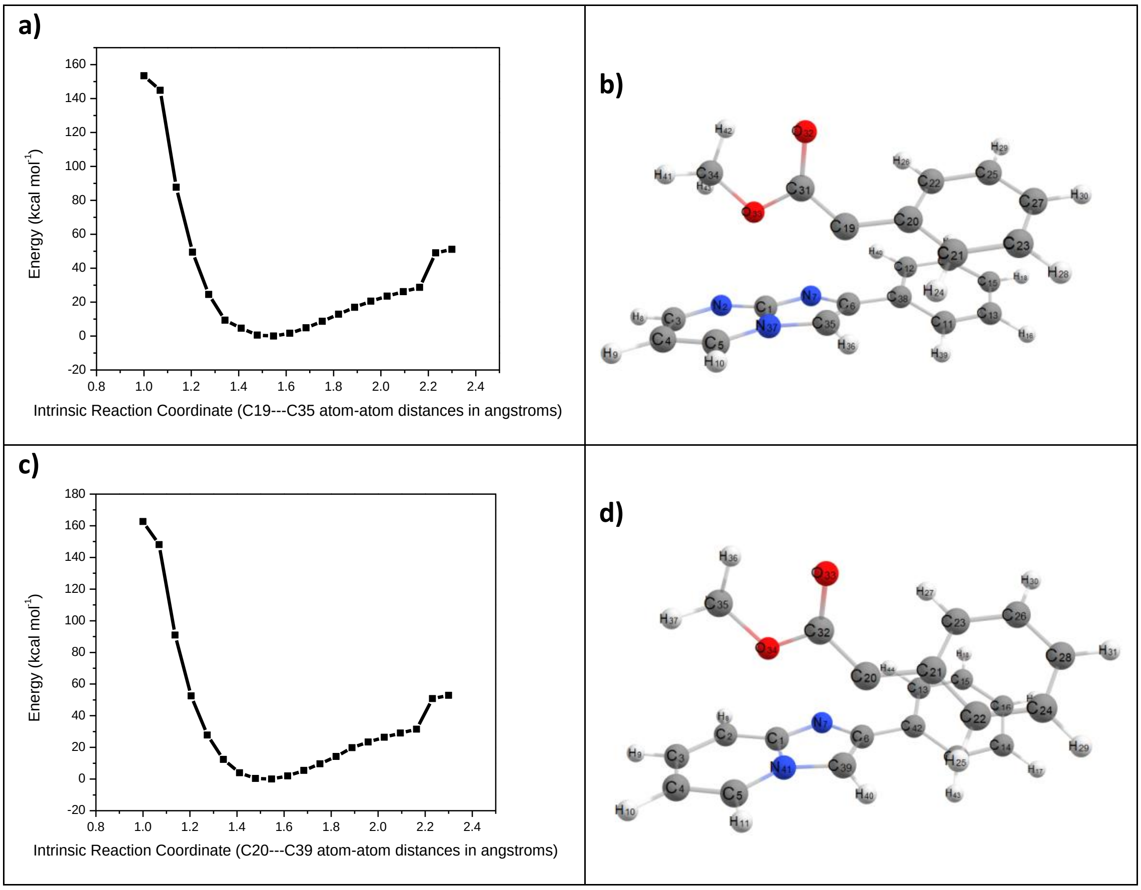

Figure S6: Relaxed scan for each carbene **10a** – aza-arene (**7a** or **7l**) approach performed using the ScanTS keyword in ORCA 6 and using the GFN2-xTB level are given in Figures **a)** and **c)**. Molecular representation and atom numbering for each carbene **10a** – aza-arene (**7a** or **7l**) approach are given in Figures **b)** and **d)**.

3. UV/ Vis Absorbance Spectra of Isolated Compounds and Mixtures

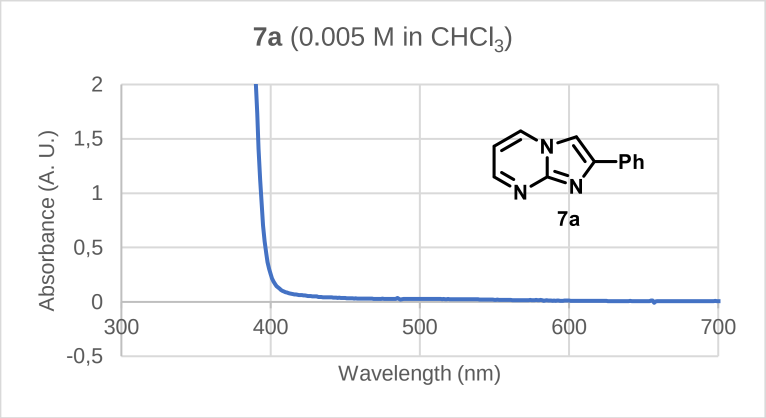

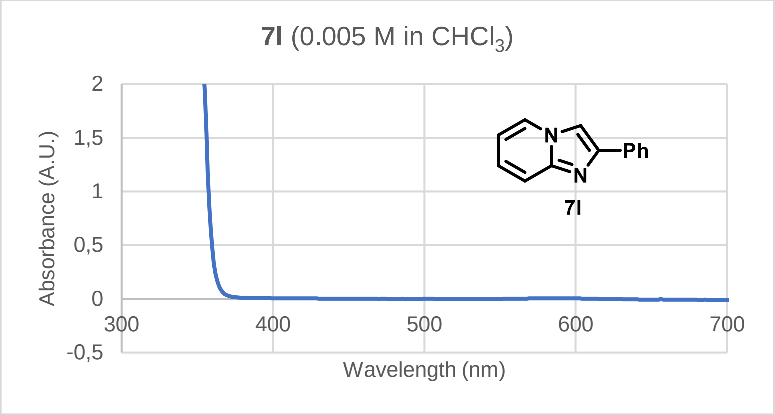
7l (0.005 M in CHCl3)
Absorbance (A.U.)
Wavelength (nm)
2
1,5
1
0,5
0
-0,5
300
400
500
600
700
N
N
Ph
7l

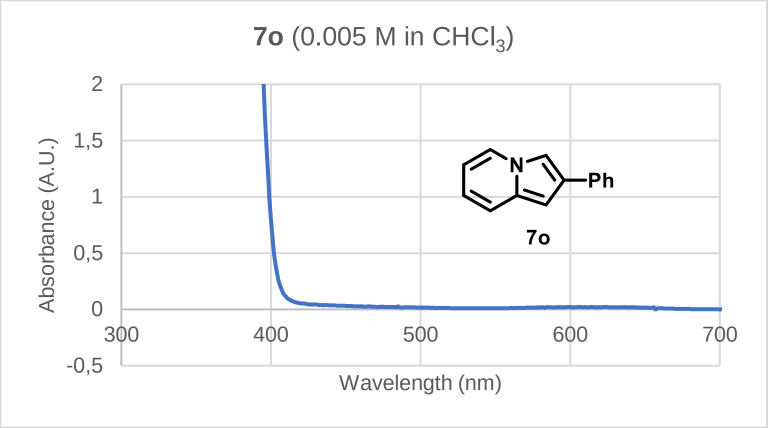
7o (0.005 M in CHCl3)
Absorbance (A.U.)
Wavelength (nm)
2
1,5
1
0,5
0
-0,5
300
400
500
600
700
N
Ph
7o

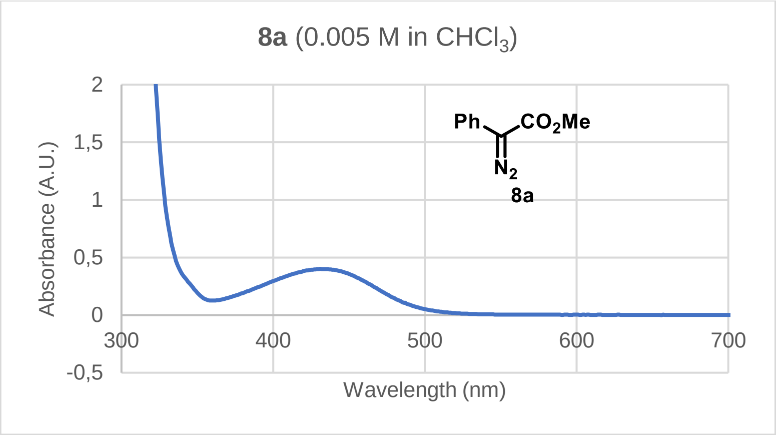
8a (0.005 M in CHCl3)
Absorbance (A.U.)
Wavelength (nm)
2
1,5
1
0,5
0
-0,5
300
400
500
600
700
Ph
CO2Me
N2
8a

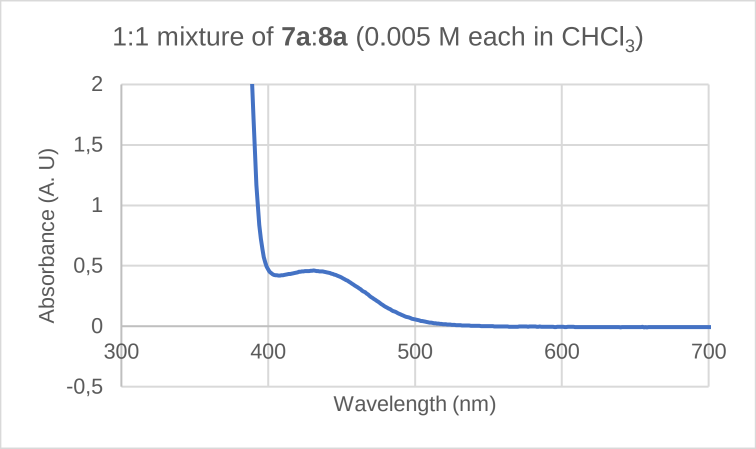
1:1 mixture of **7a**:**8a** (0.005 M each in $CHCl_3$)
Absorbance (A. U)
2
1,5
1
0,5
0
-0,5
300
400
500
600
700
Wavelength (nm)

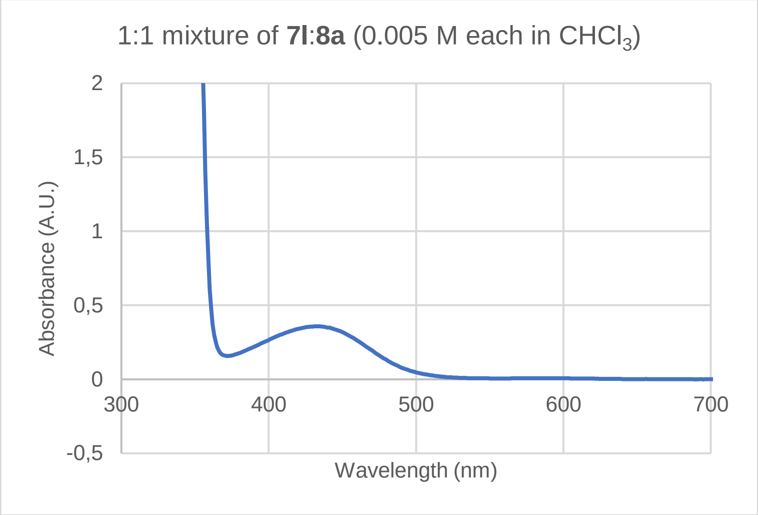
1:1 mixture of **7l**:**8a** (0.005 M each in $CHCl_3$)
Absorbance (A.U.)
2
1,5
1
0,5
0
-0,5
300
400
500
600
700
Wavelength (nm)

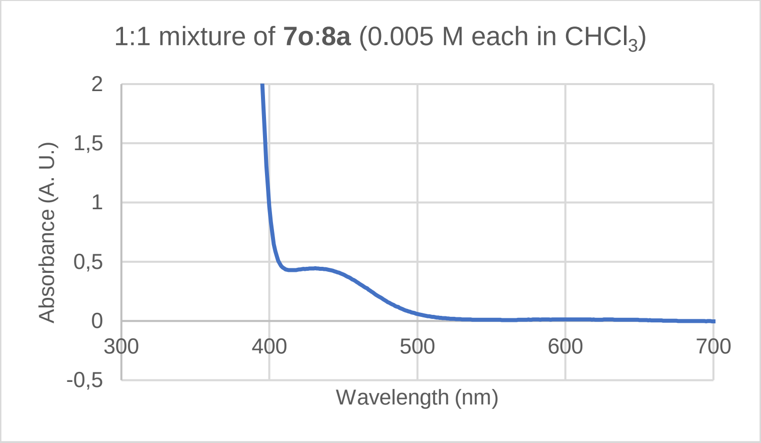
1:1 mixture of **7o**:**8a** (0.005 M each in $CHCl_3$)
Absorbance (A. U.)
2
1,5
1
0,5
0
-0,5
300
400
500
600
700
Wavelength (nm)

UV/Vis spectra superimposed:

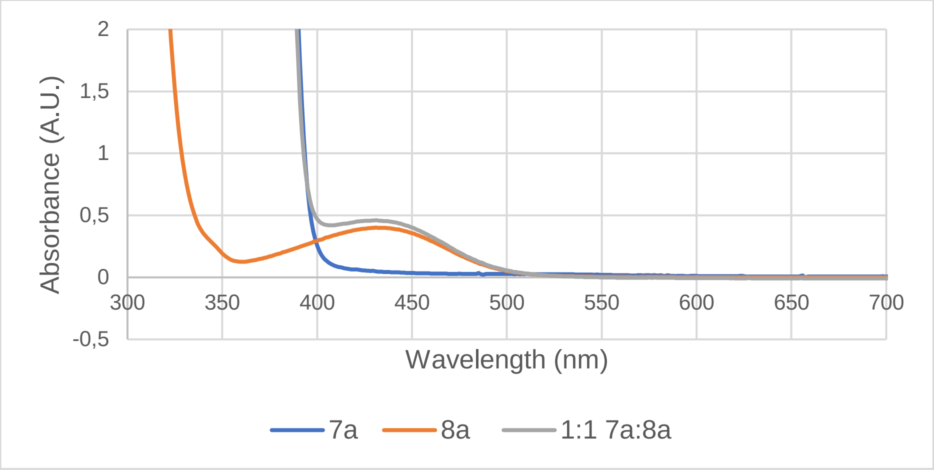

Conclusion: An EDA complex formed between **7a** and **8a** is not observed.

UV/Vis spectra superimposed:

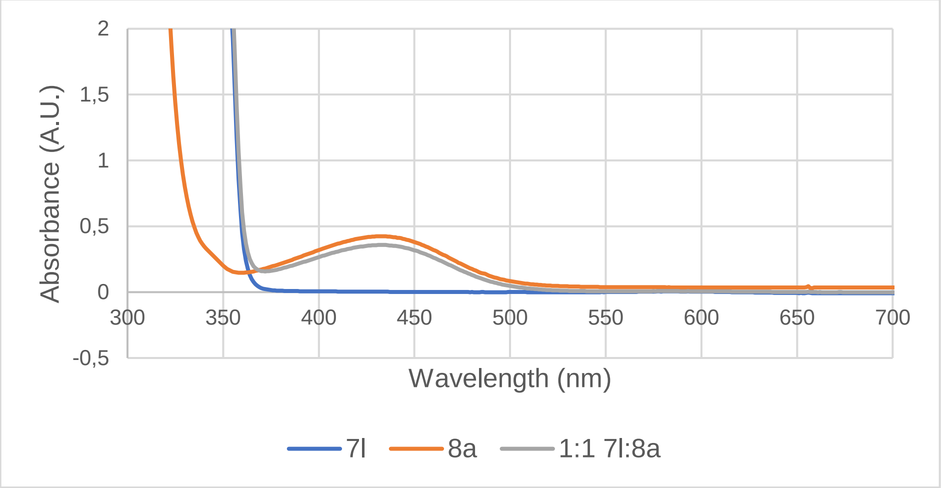

Conclusion: An EDA complex formed between **7l** and **8a** is not observed.

UV/Vis spectra superimposed:

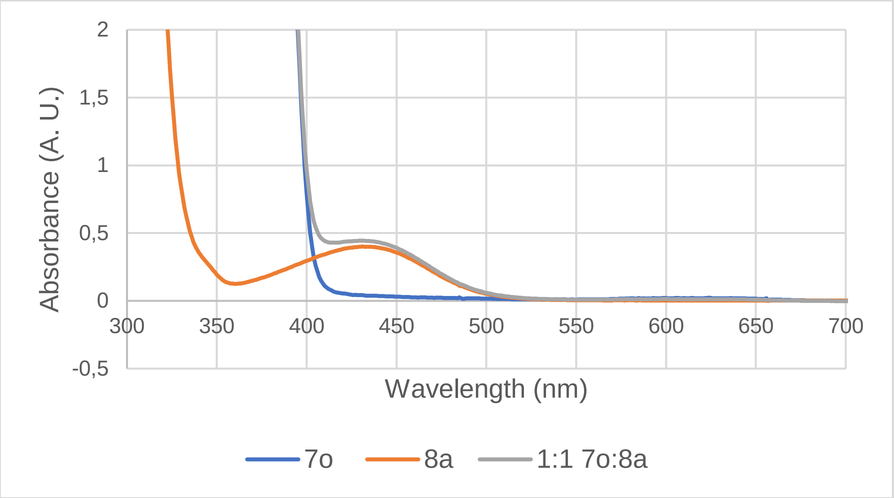

Conclusion: An EDA complex formed between **7o** and **8a** is not observed.

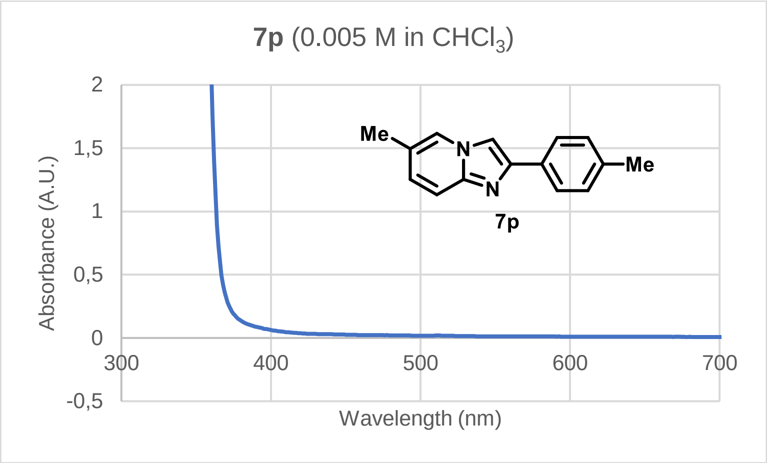

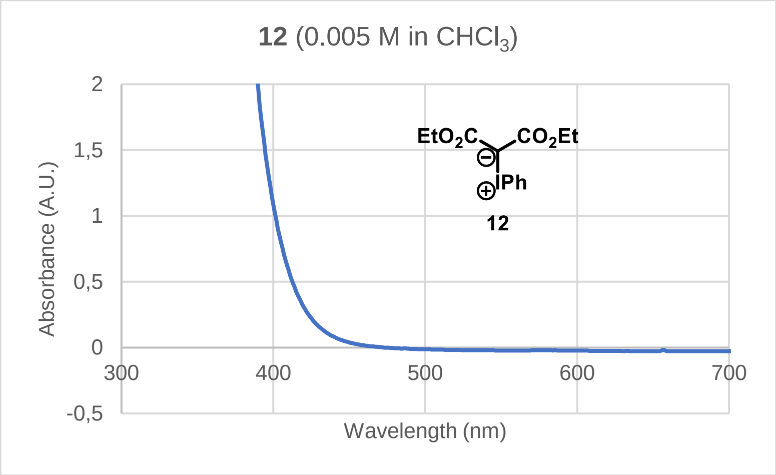

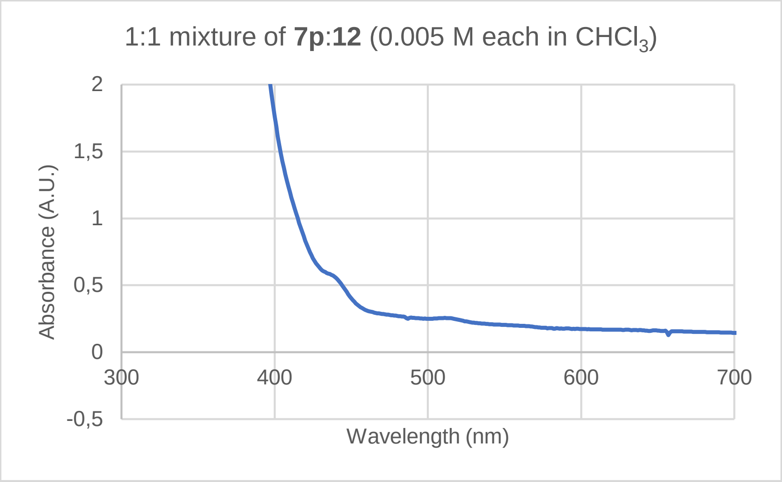

Each component at a concentration of 0.005 M in $CHCl_3$, previous UV/Vis spectra superimposed:

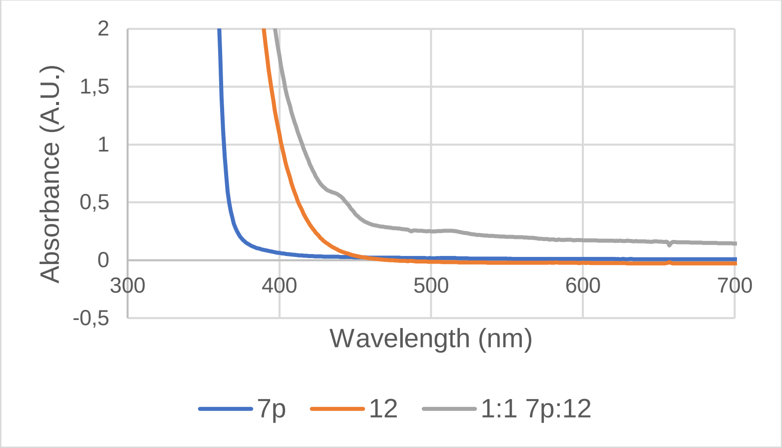

Repeating the same analyses as before, but this time with each component at a concentration of 0.05 M in $CHCl_3$:

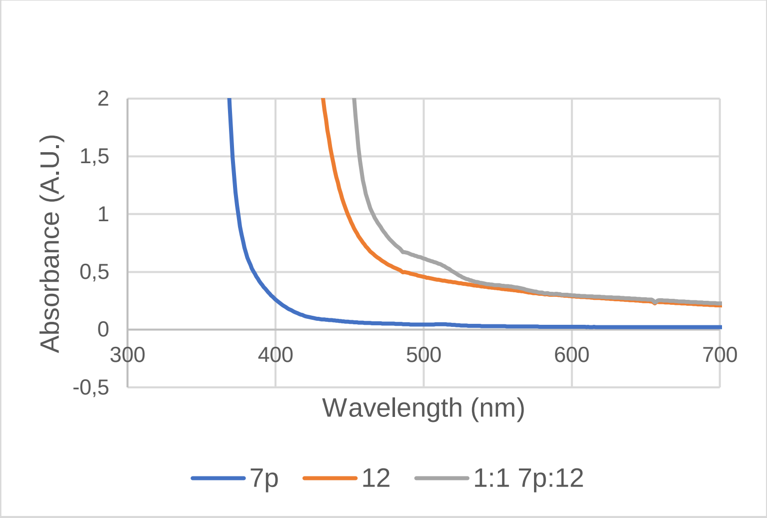

Conclusion: An EDA complex between **7p** and **12** seems to be formed and could be possibly involved in the reaction mechanism.

4. Evaluation of the Photostability of *aza*-Arenes 7a, 7l and 7o Under Blue Light Irradiation in $CHCl_3$

Based on the extensive degradation previously observed for indolizine **7o** and the UV/Vis absorption spectra of compounds **7a**, **7l** and **7o** obtained, in which these compounds seemed to absorb near the blue region of the visible spectrum (*ca.* 450 nm), we decided to evaluate control experiments consisting of allowing each of these representative members (containing either 3N, 2N or 1N, respectively, 0.2 mmol each) to stir at room temperature for 24 h in $CHCl_3$ for 24 h (Figure S7), then to estimate the recovered amount of each of them by performing a ^{1}H NMR analysis in the presence of 1,3,5-trimethoxybenzene as an internal standard. Apparently, *these compounds are not perfectly photostable under blue light irradiation in $CHCl_3$* (Table S7).

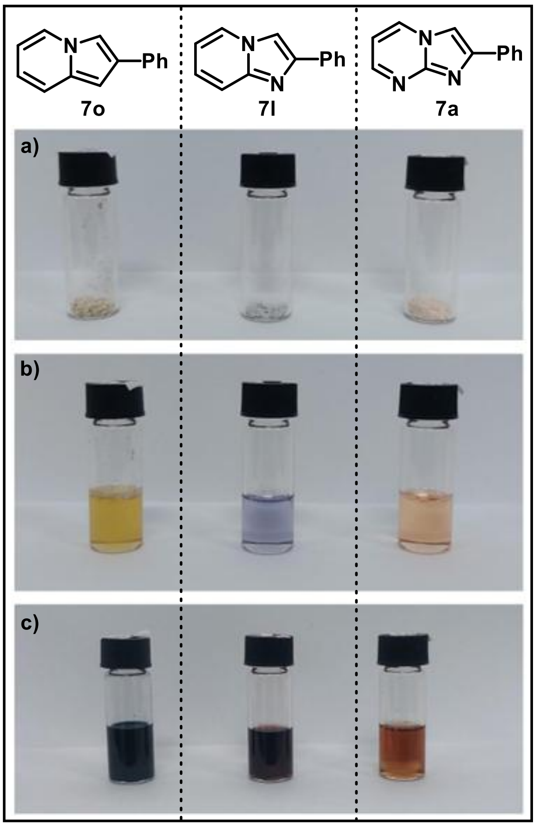

Figure S7. a) Solids of **7a**, **7l** and **7o** at t = 0. **b)** Solutions of **7a**, **7l** and **7o** in $CHCl_3$ at t = 0 (before irradiation under blue light). **c)** Solutions of **7a**, **7l** and **7o** in $CHCl_3$ after irradiating them under blue light at t = 24 h.

Table S7. Control experiments evaluating the photostability of compounds **7a**, **7l** and **7o** under blue light irradiation. [a]Estimated yield based on the analysis of the ^{1}H NMR of the crude reaction mixture employing 1,3,5-trimethoxybenzene as an internal standard.

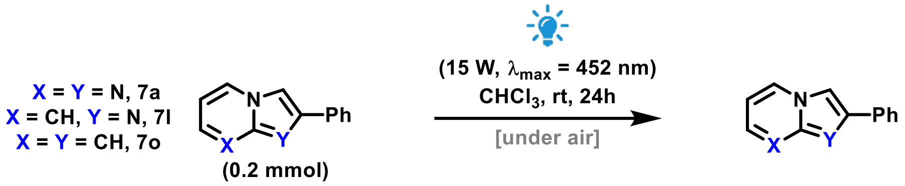

entry	*aza*-Arene	Recovered *aza*-arene (%)[a]	Observation
1	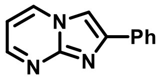 **7a**	91 ± 7	the initial light-colored solid is recovered with ~little change in color after 24 h
2	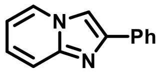 **7l**	94 ± 5	the initial light-colored solid becomes dark after 24 h
3	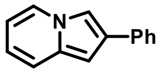 **7o**	85 ± 2	the initial light-colored solid becomes dark after 24 h

5. Copies of ^{1}H, ^{13}C{^{1}H} and ^{19}F{^{1}H} NMR Spectra of Compounds

Molecule 7a: ^{1}H NMR (500 MHz, $CDCl_3$)

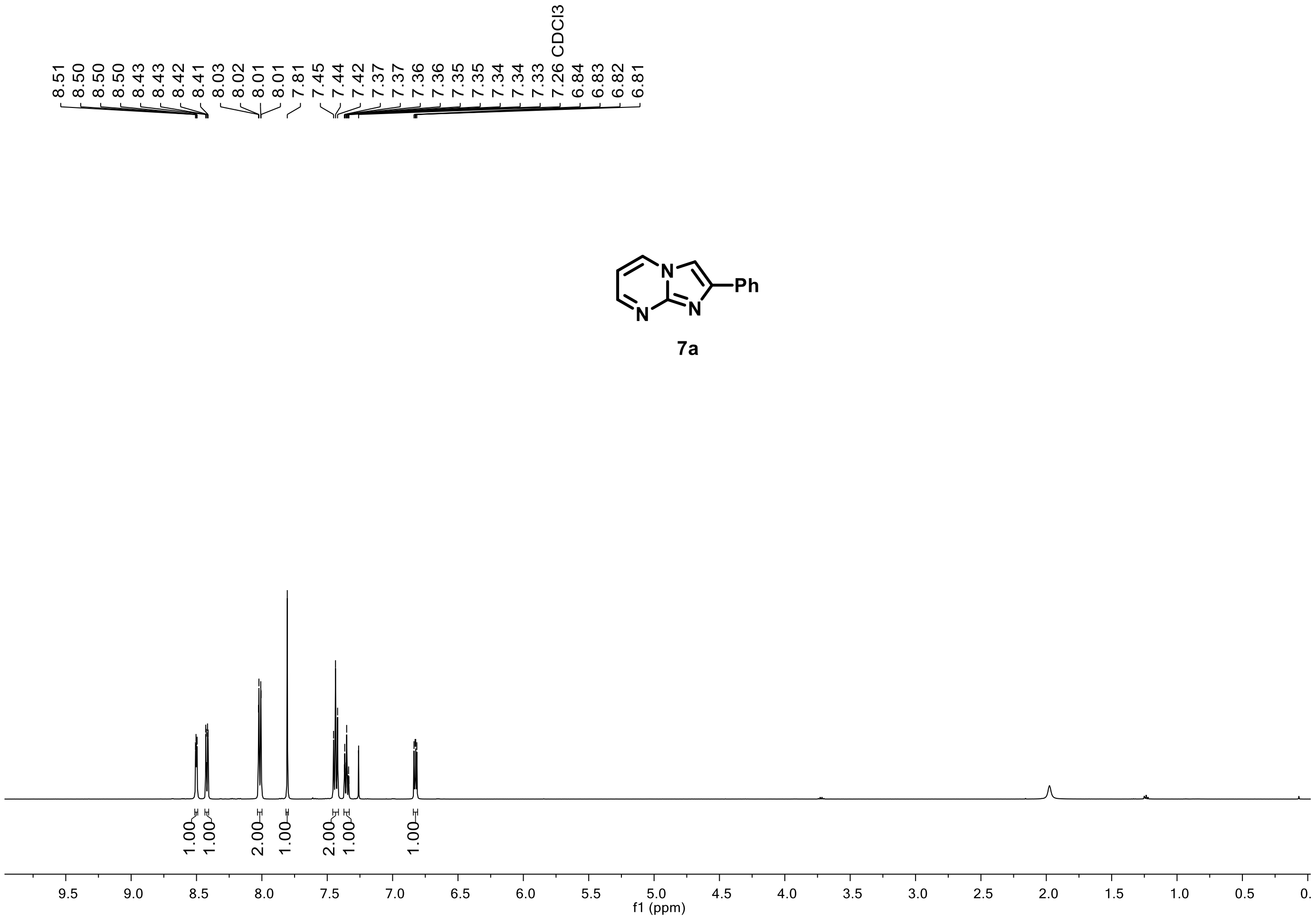

Molecule 7a: $^{13}C\{^{1}H\}$ NMR (125 MHz, $CDCl_3$)

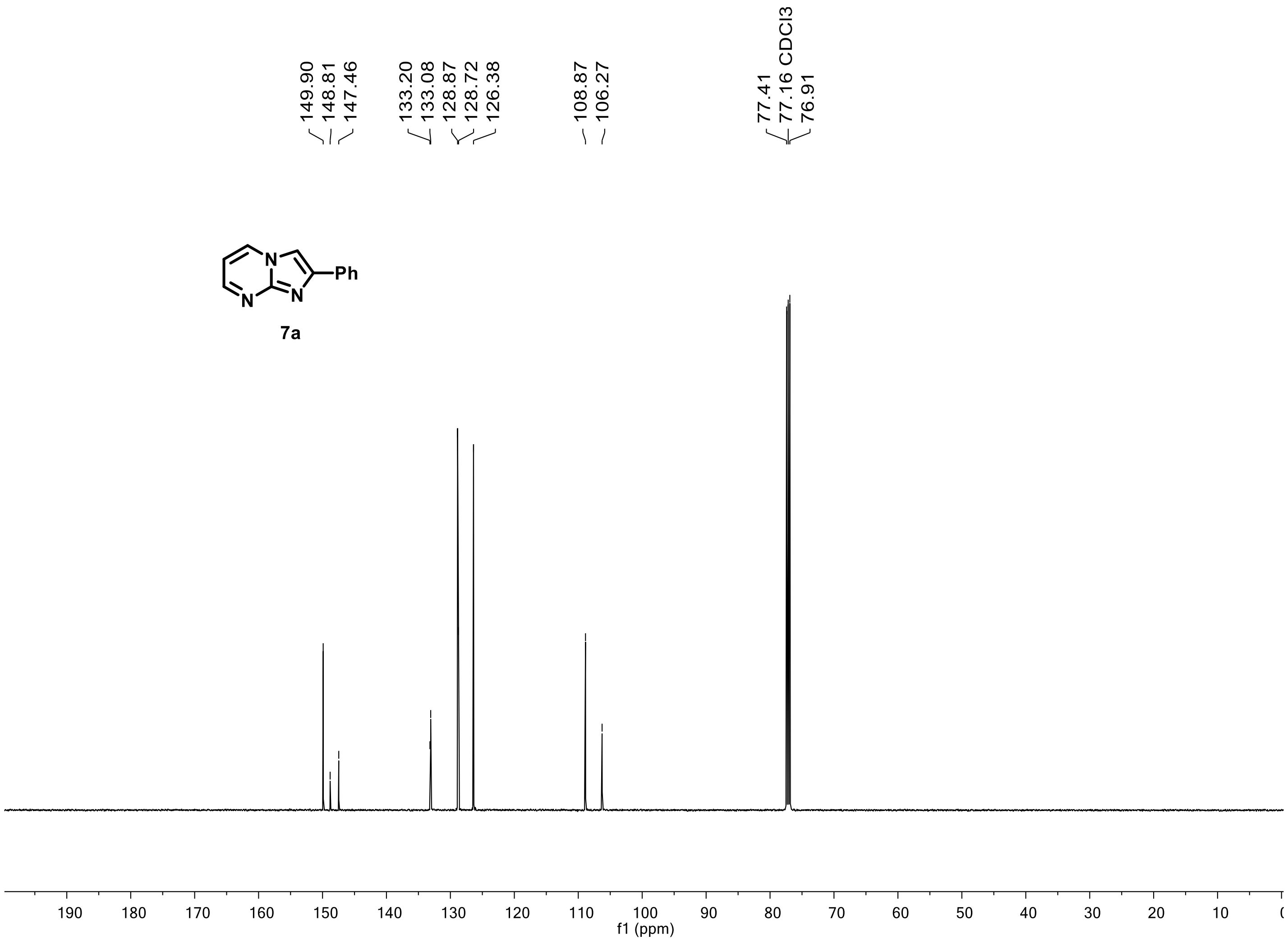

Molecule 7b: ^{1}H NMR (500 MHz, $CDCl_3$)

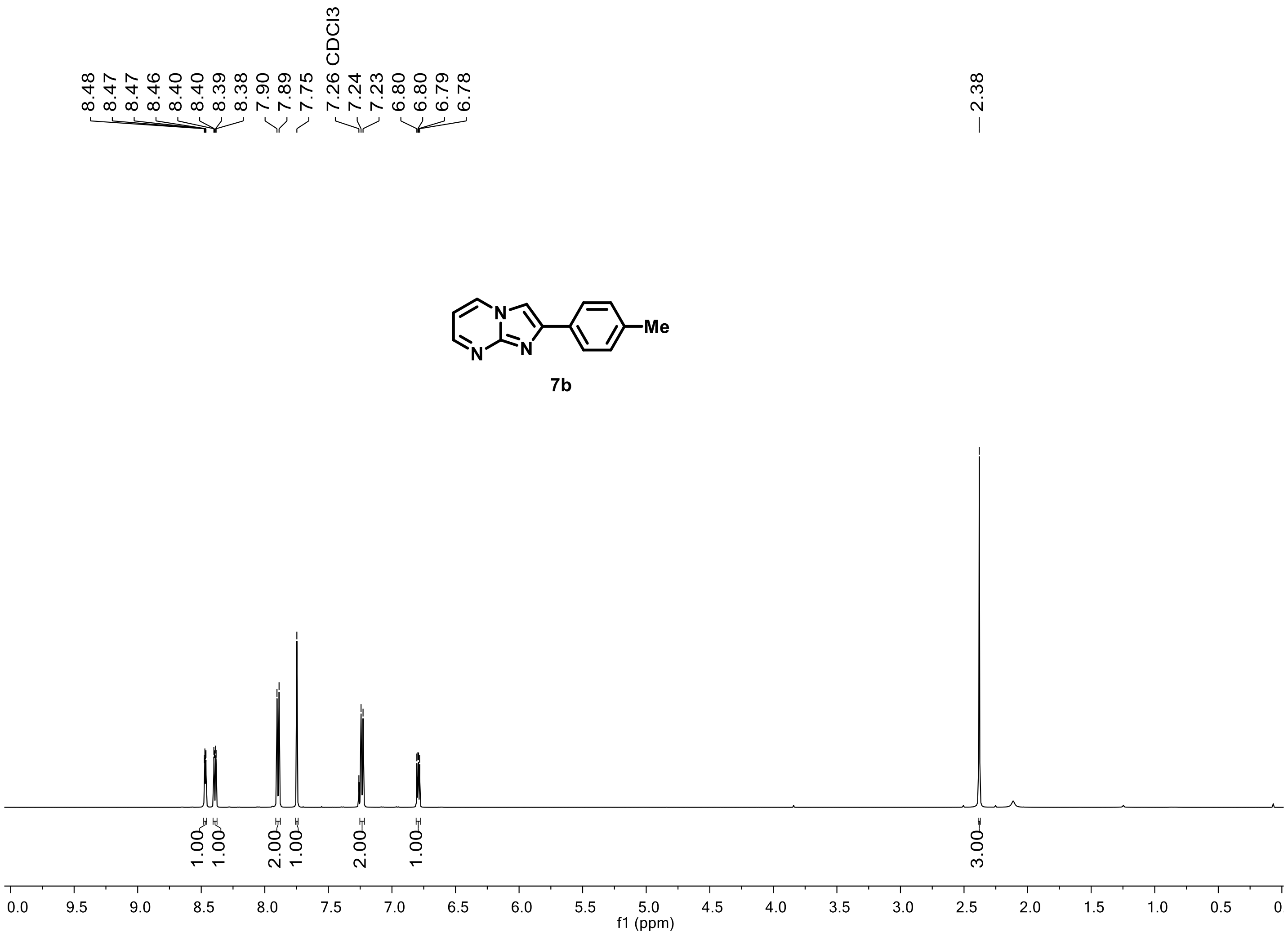

Molecule 7b: $^{13}C\{^1H\}$ NMR (125 MHz, $CDCl_3$)

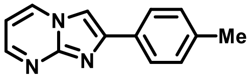

7b

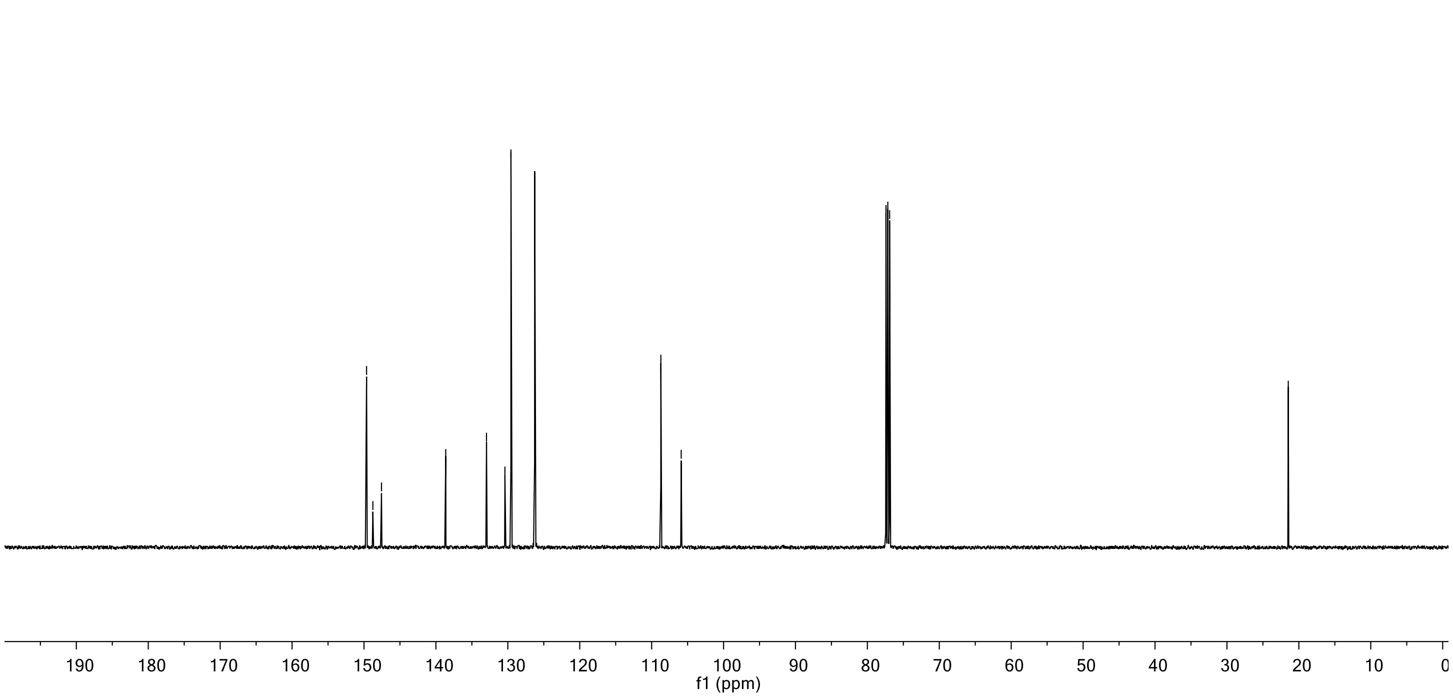

Molecule 7c: ^{1}H NMR (400 MHz, $CDCl_3$)

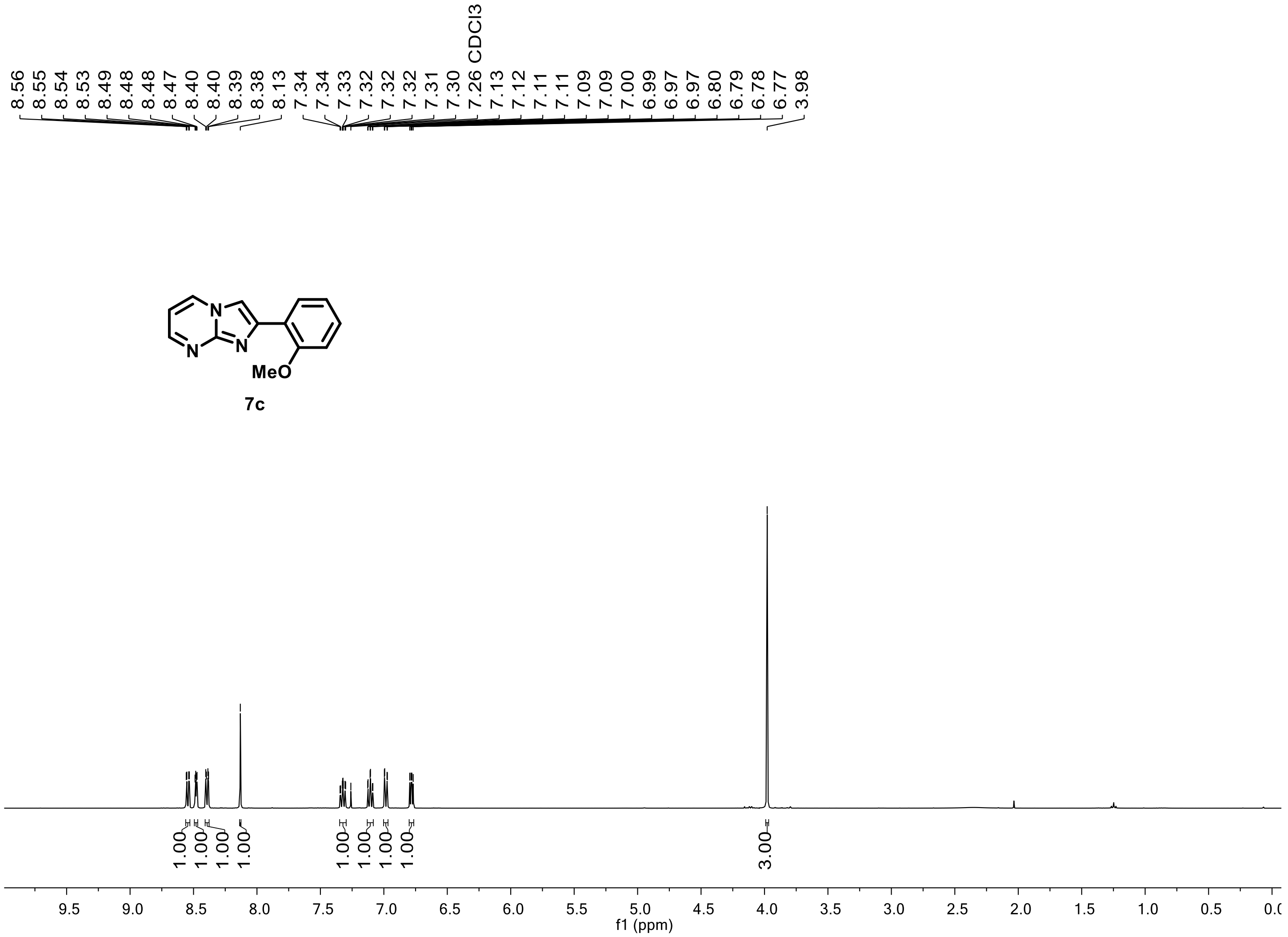

7c

Molecule 7c: $^{13}C\{^{1}H\}$ NMR (100 MHz, $CDCl_3$)

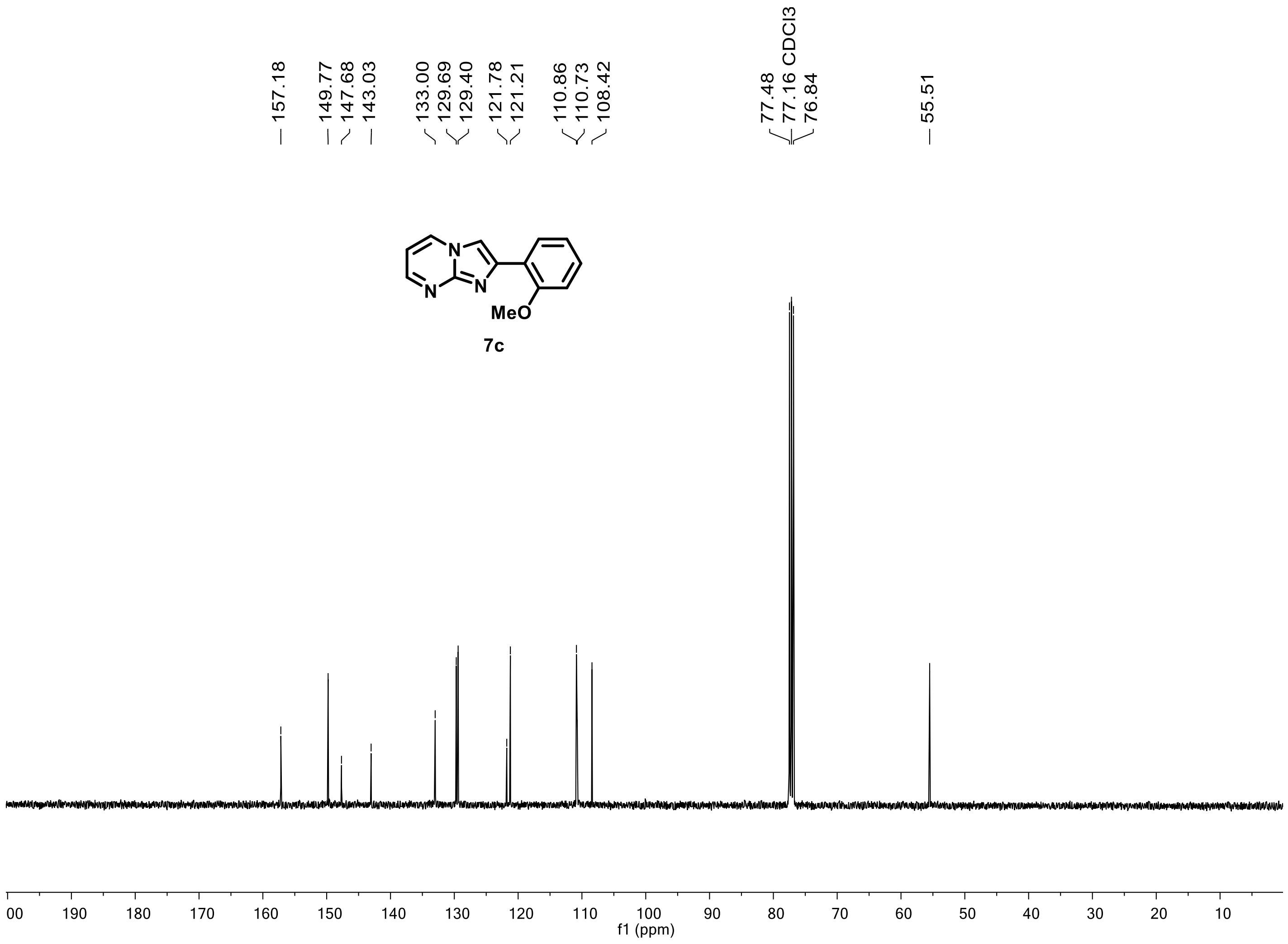

Molecule 7d: ^{1}H NMR (400 MHz, $CDCl_3$)

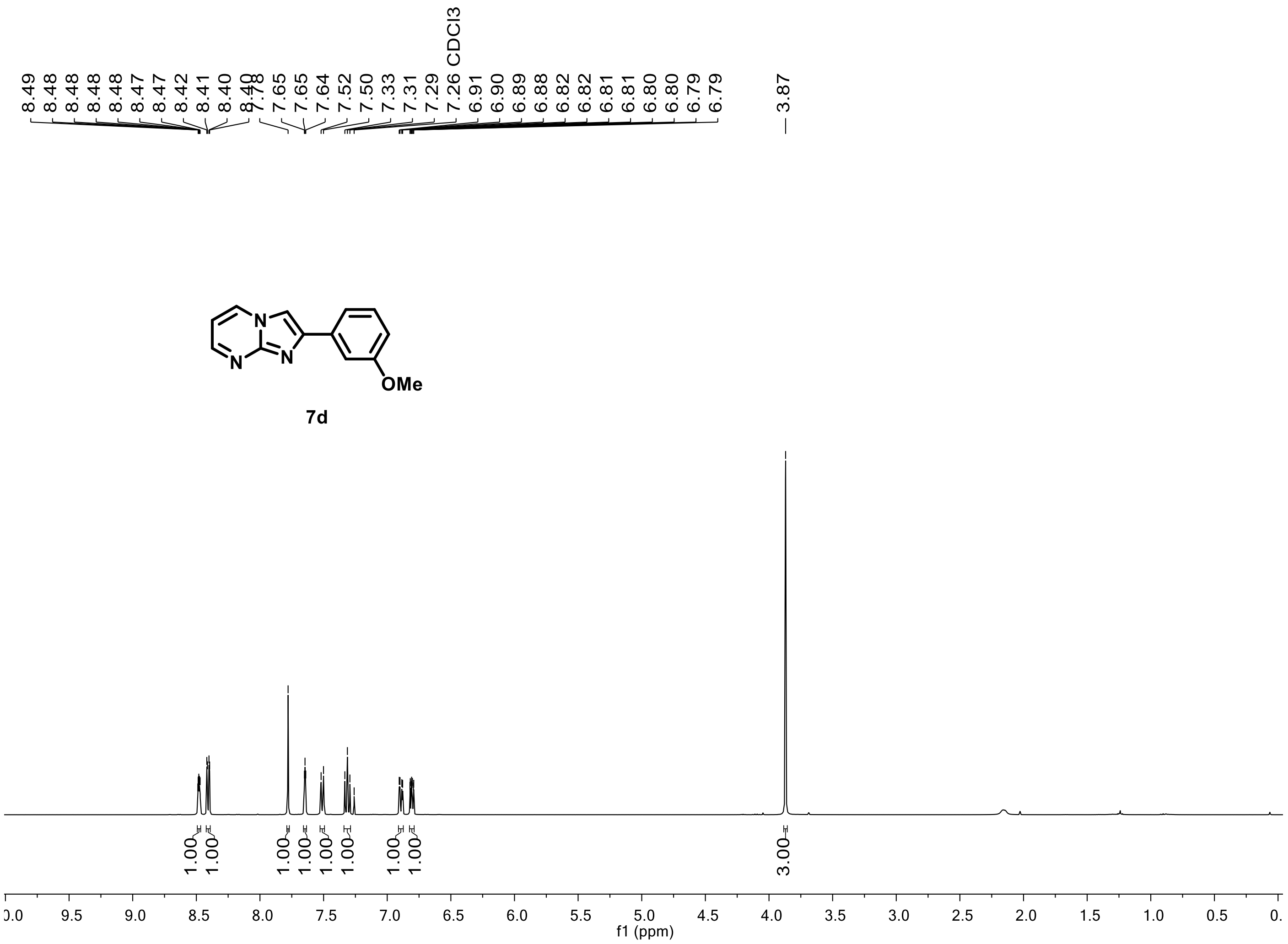

Molecule 7d: $^{13}C\{^{1}H\}$ NMR (100 MHz, CDCl$_3$)

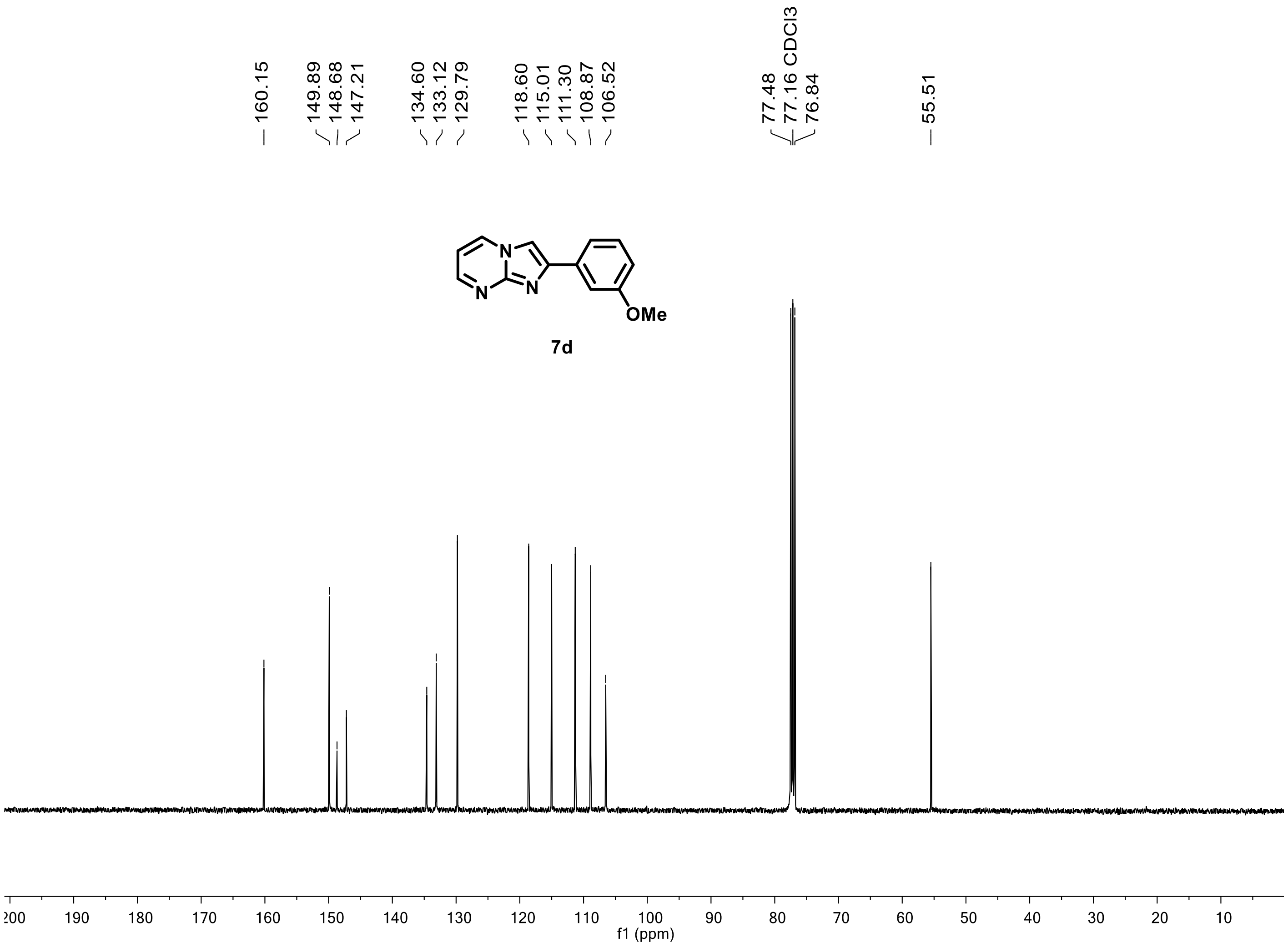

Molecule 7e: ^{1}H NMR (500 MHz, $CDCl_3$)

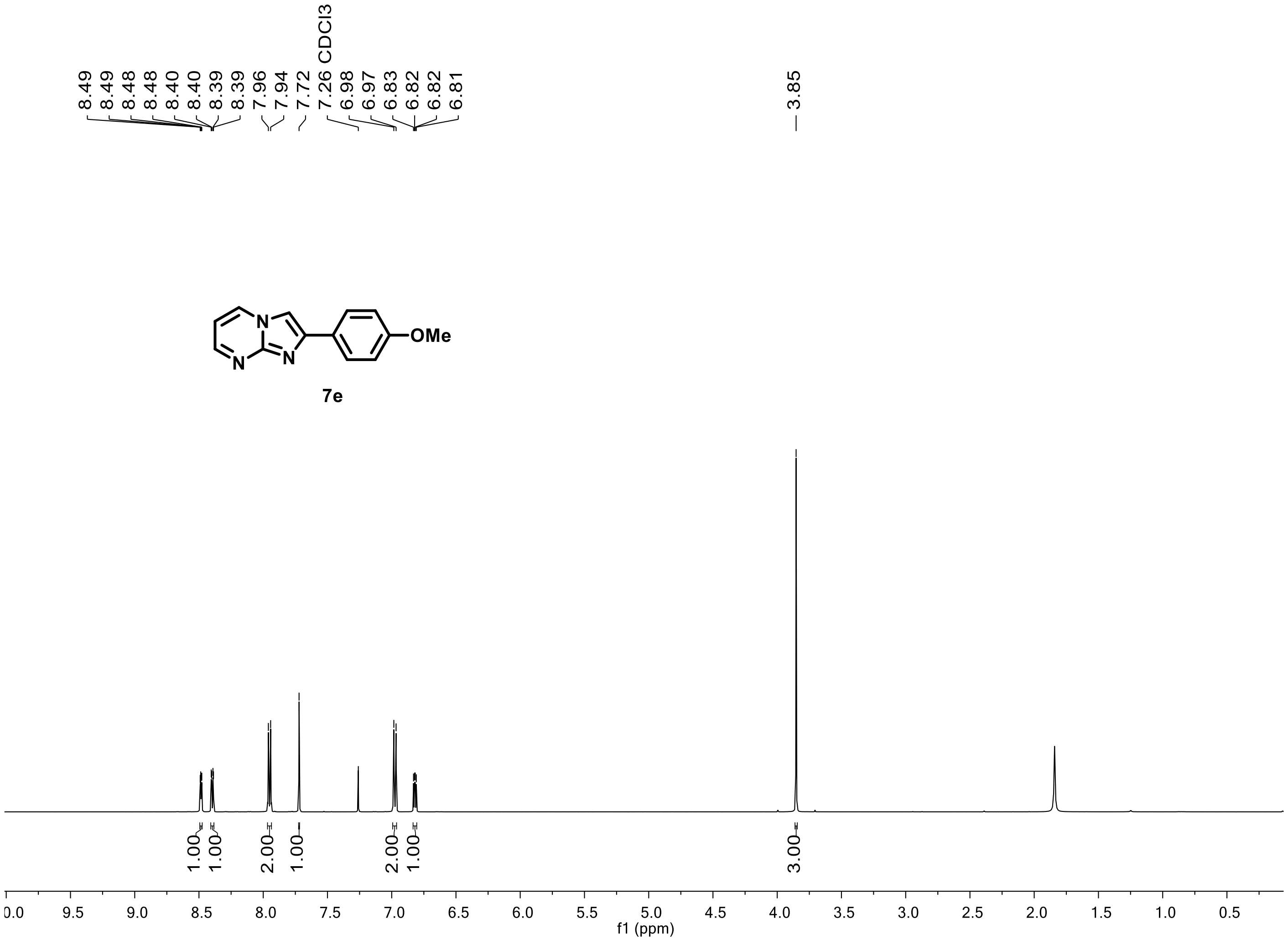

Molecule 7e: $^{13}C\{^1H\}$ NMR (125 MHz, $CDCl_3$)

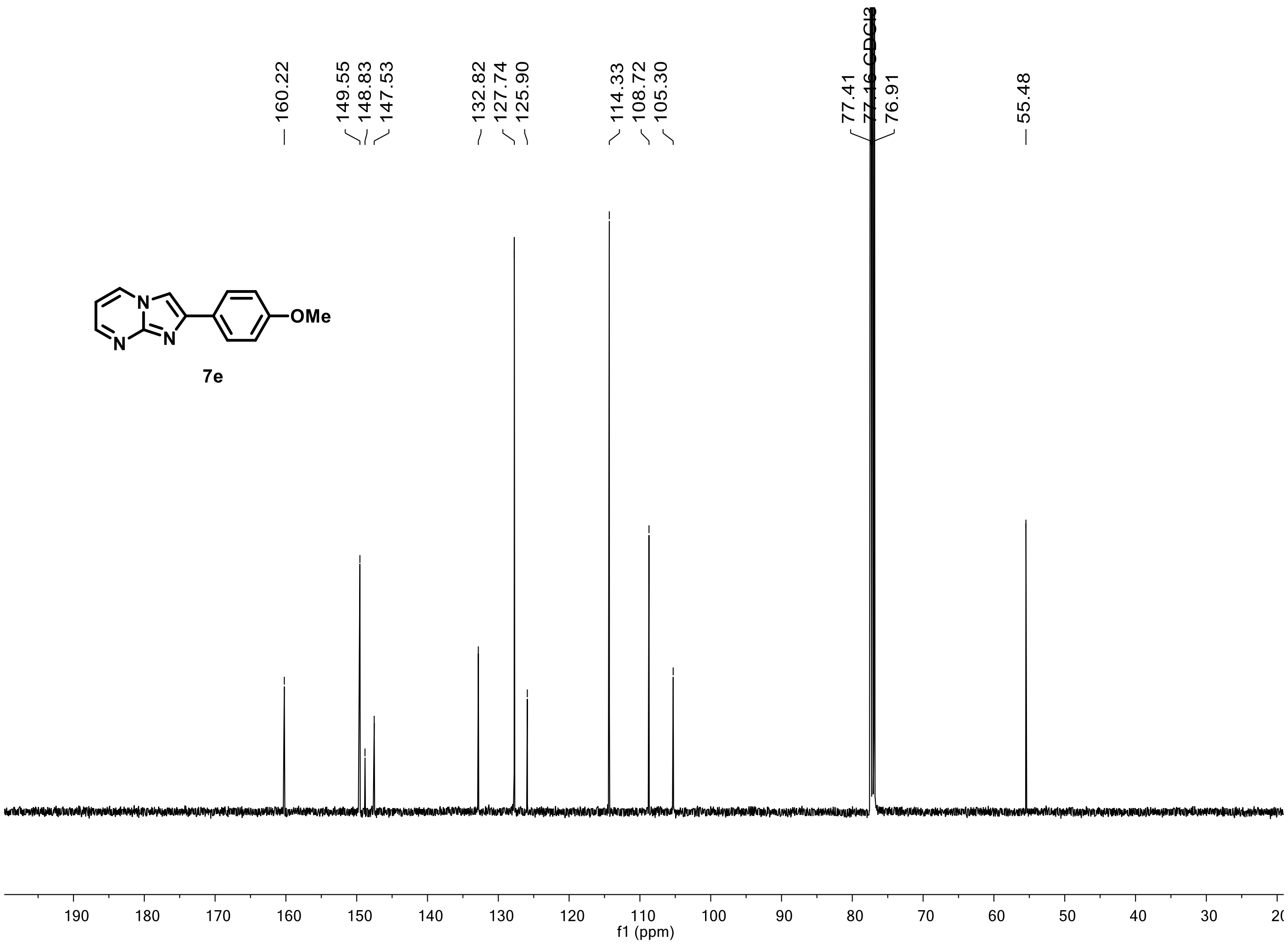

Molecule 7f: ^{1}H NMR (500 MHz, *d₆*-DMSO)

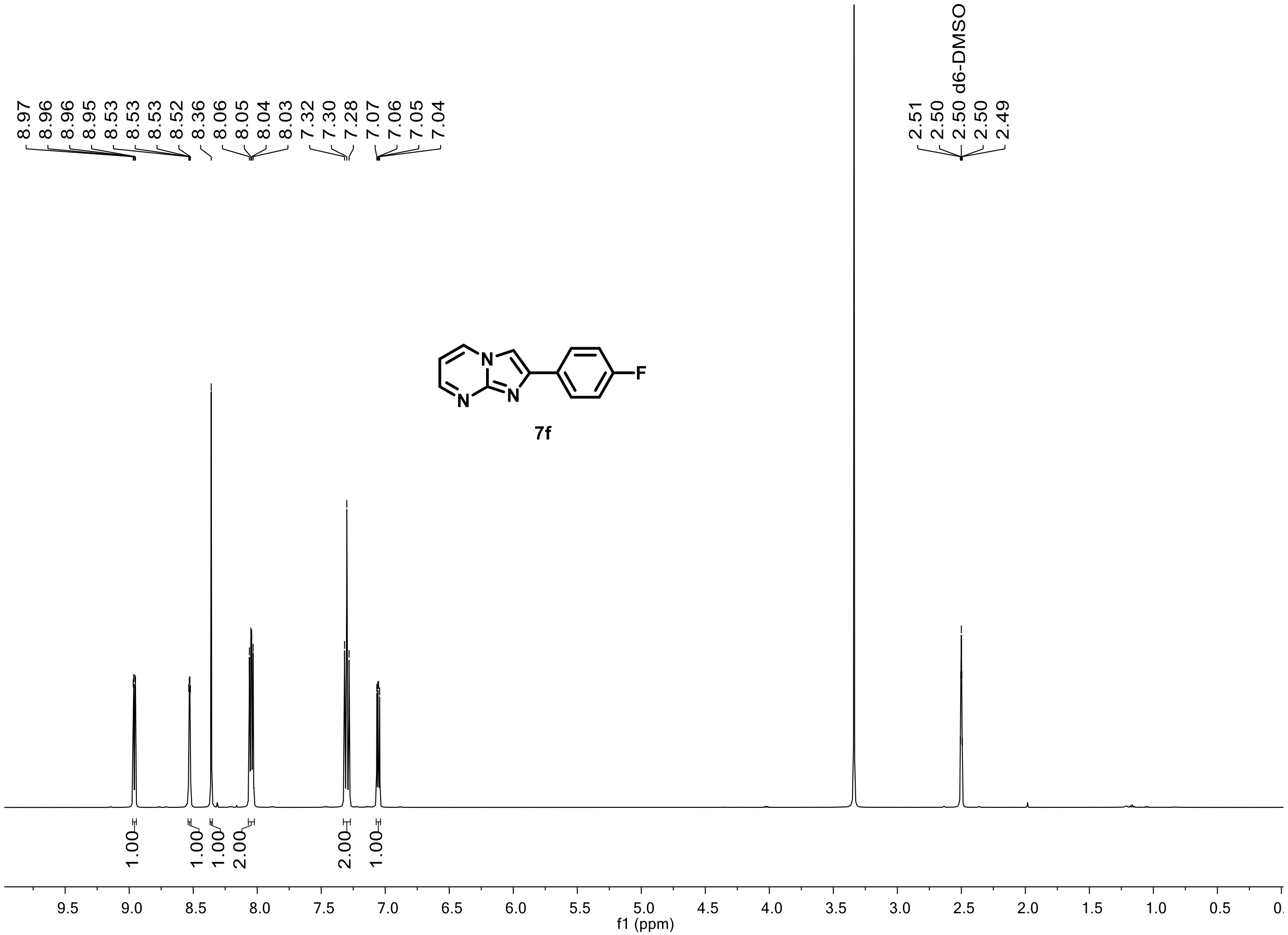

Molecule 7f: $^{13}C\{^{1}H\}$ NMR (125 MHz, d_6-DMSO)

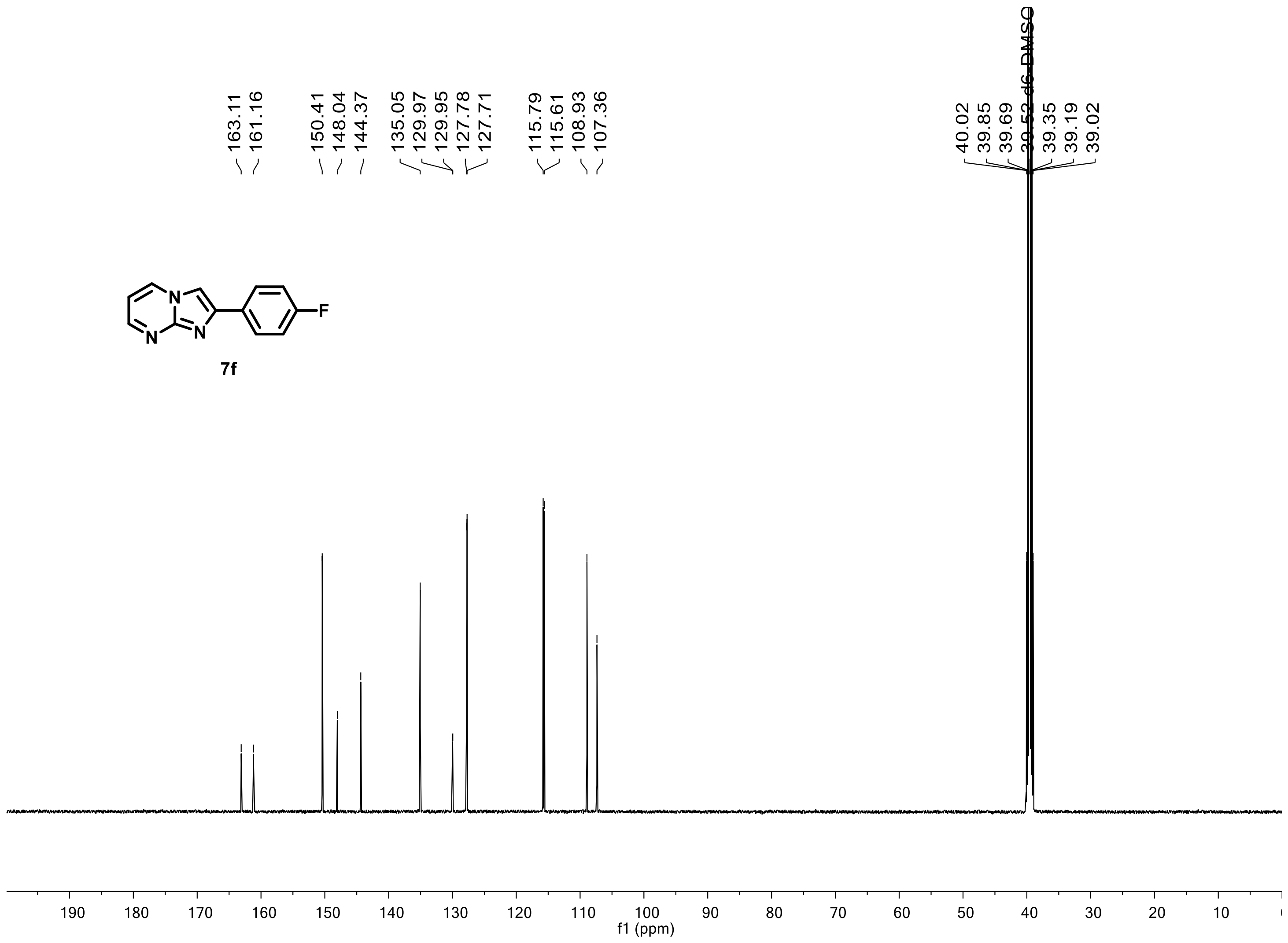

Molecule 7f: $^{19}F\{^1H\}$ NMR (470 MHz, d_6-DMSO)

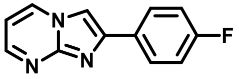

7f

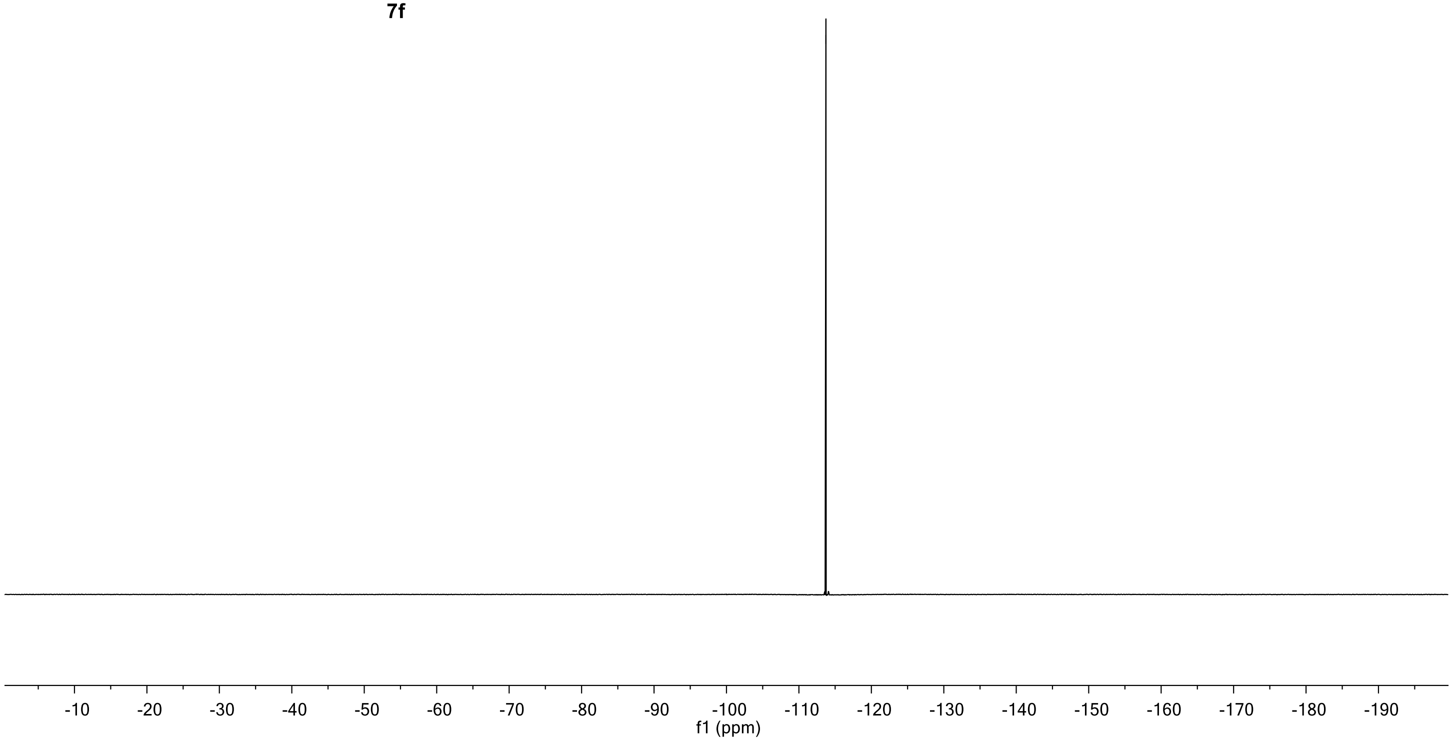

Molecule 7g: ^{1}H NMR (300 MHz, d_6-DMSO)

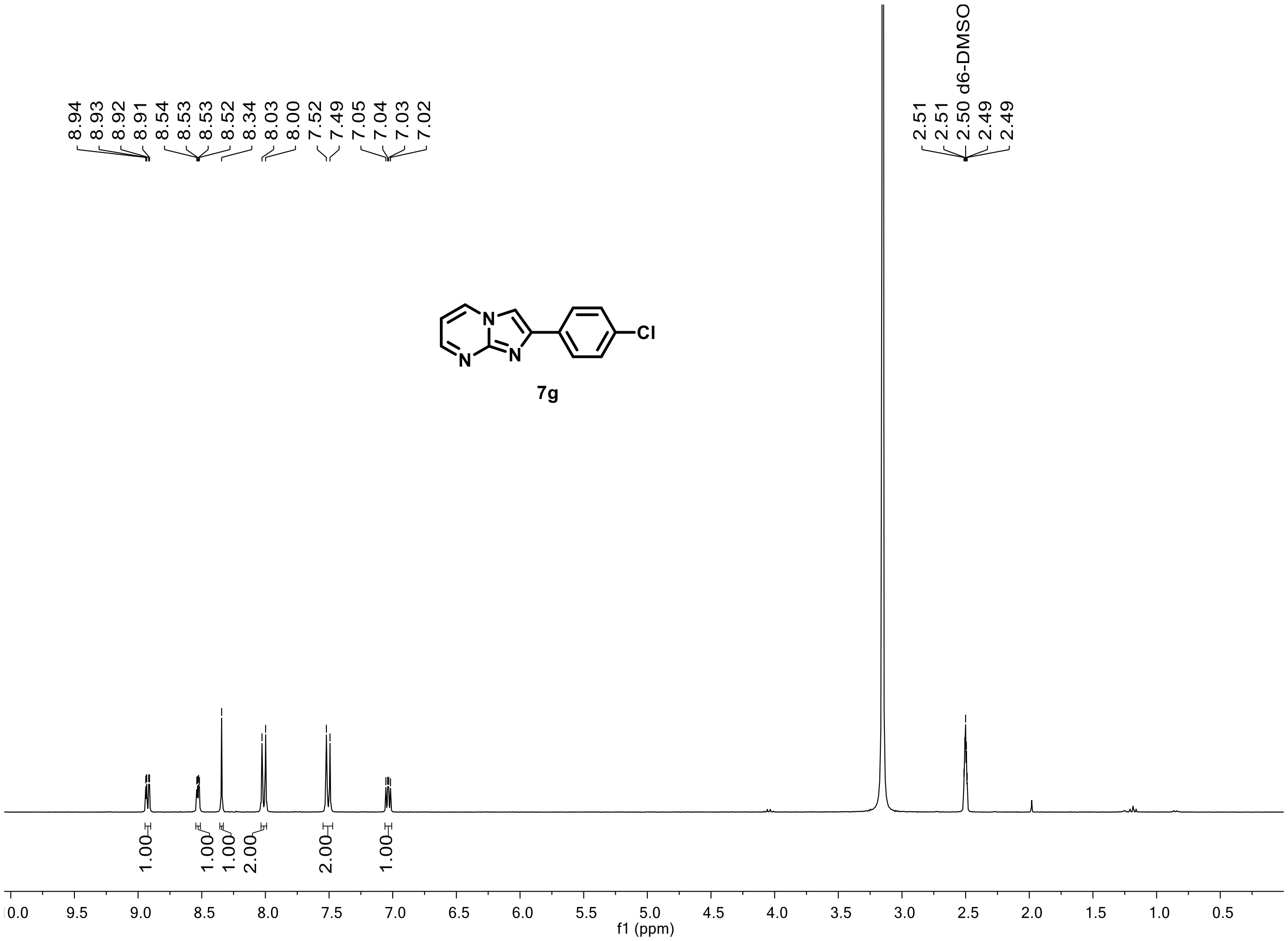

Molecule 7g: $^{13}C\{^{1}H\}$ NMR (75 MHz, d_6-DMSO)

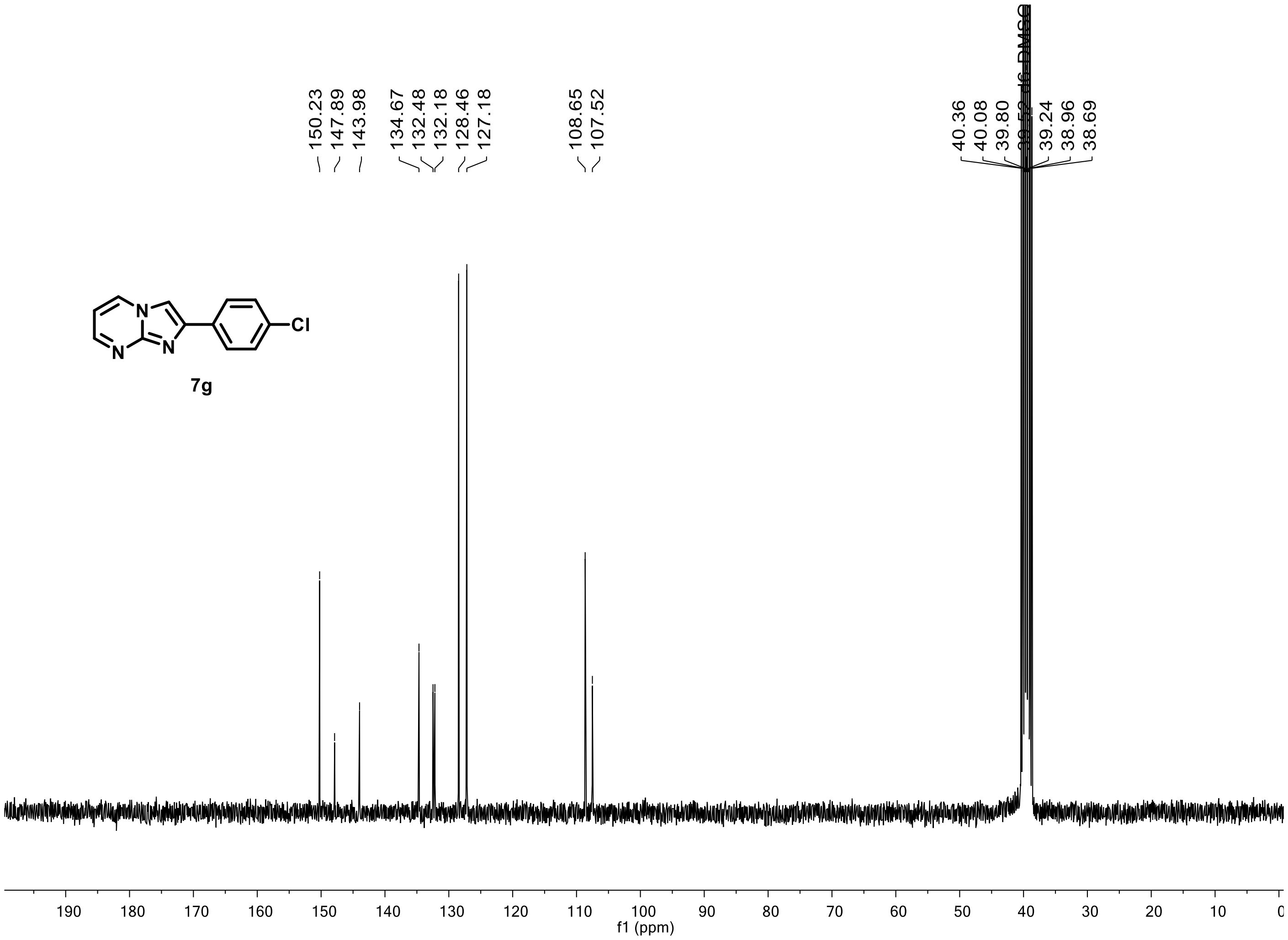

Molecule 7h: ^{1}H NMR (300 MHz, *d₆*-DMSO)

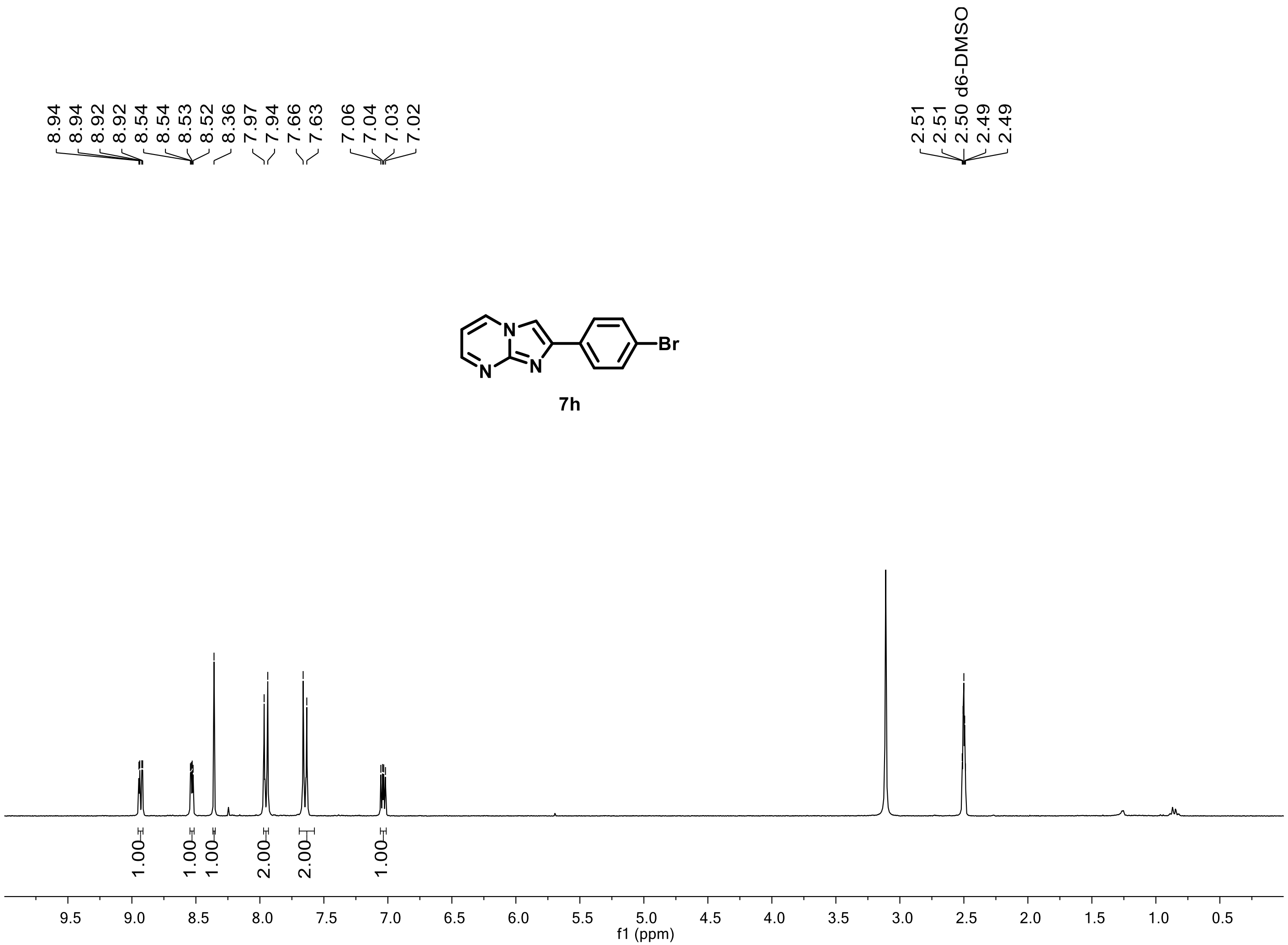

Molecule 7h: $^{13}C\{^1H\}$ NMR (75 MHz, d_6-DMSO)

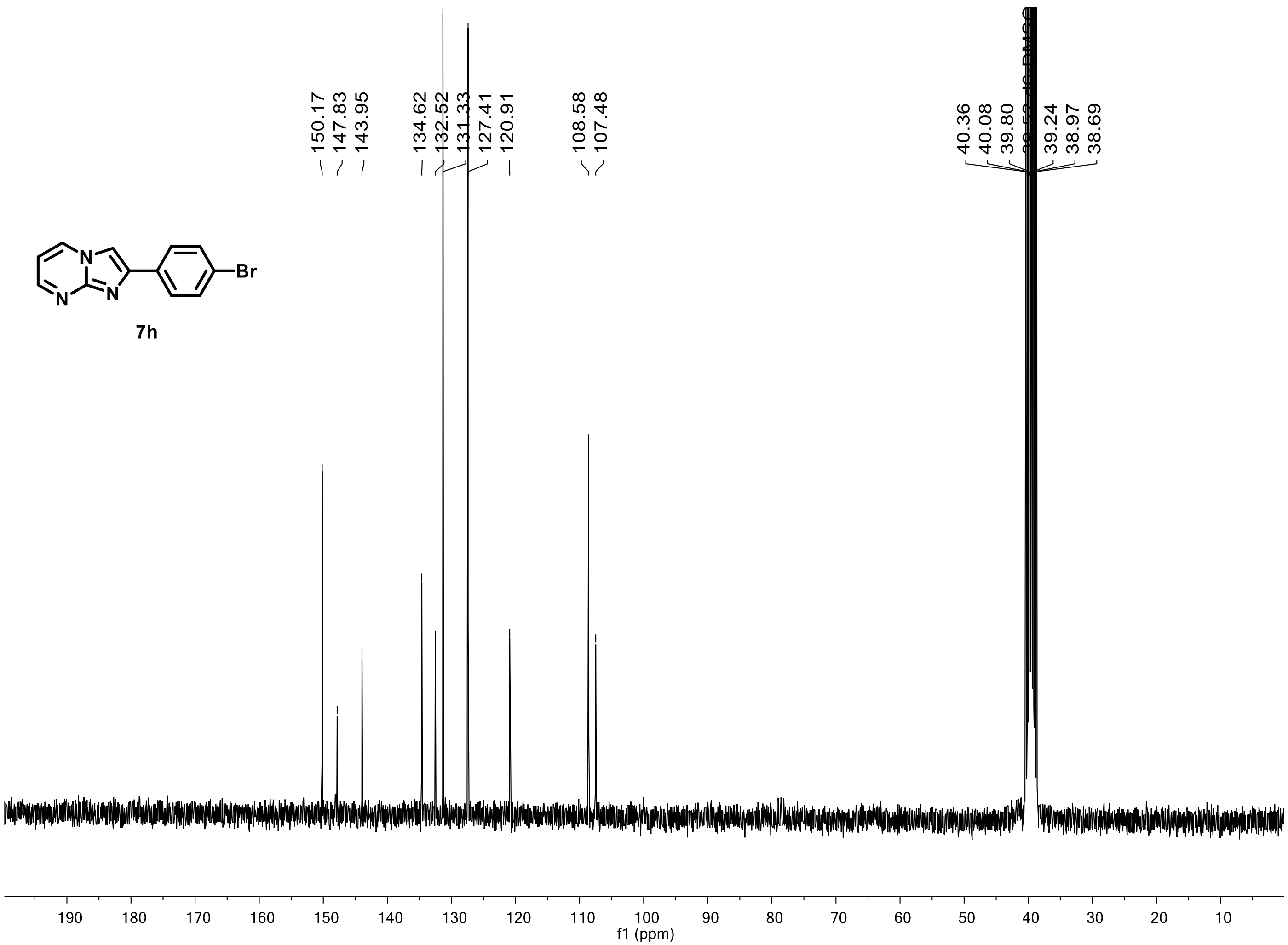

Molecule 7i: ^{1}H NMR (600 MHz, d_6-DMSO)

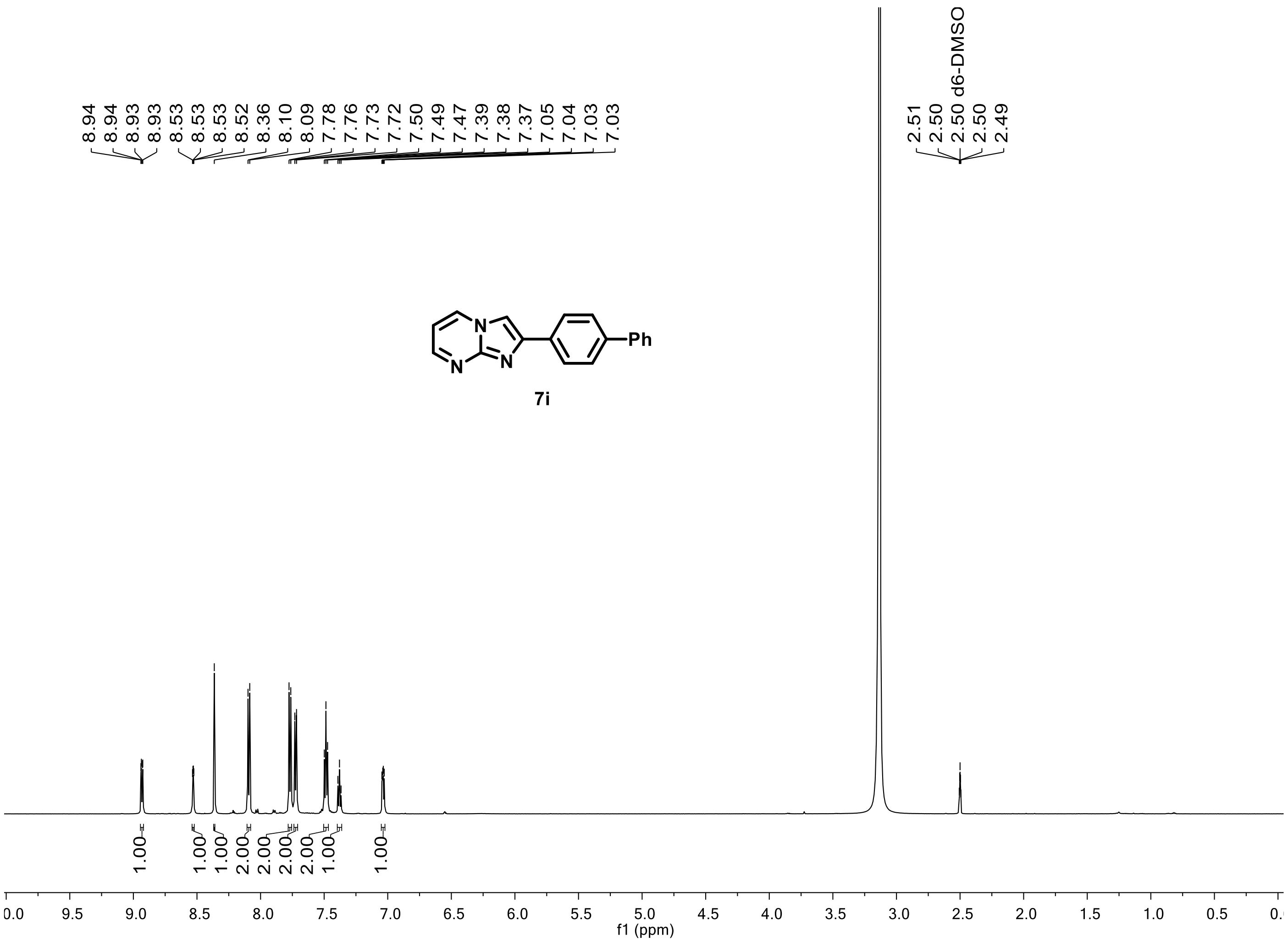

Molecule 7i: $^{13}C\{^{1}H\}$ NMR (150 MHz, d_6-DMSO)

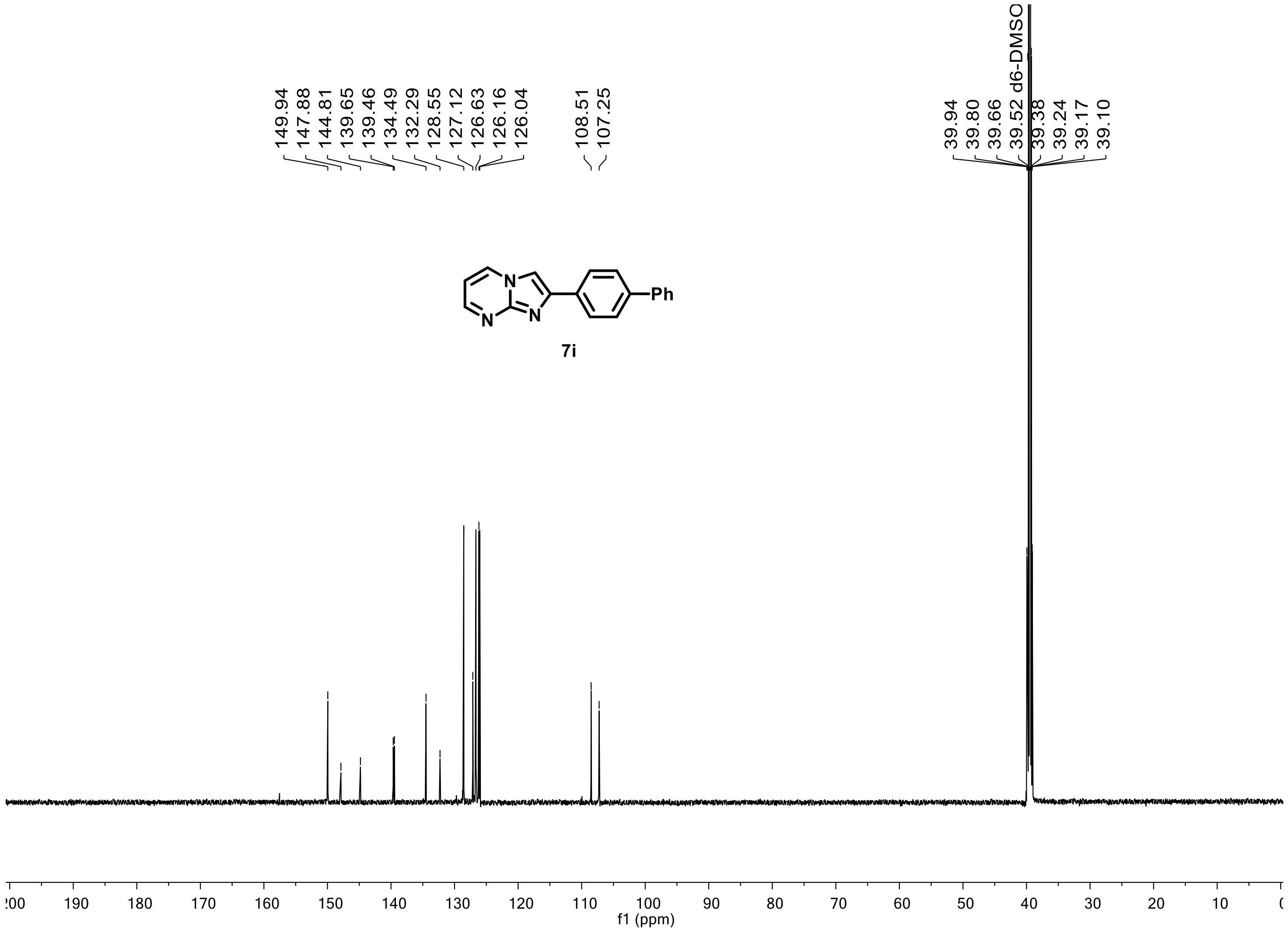

Molecule 7j: ^{1}H NMR (500 MHz, CDCl$_3$)

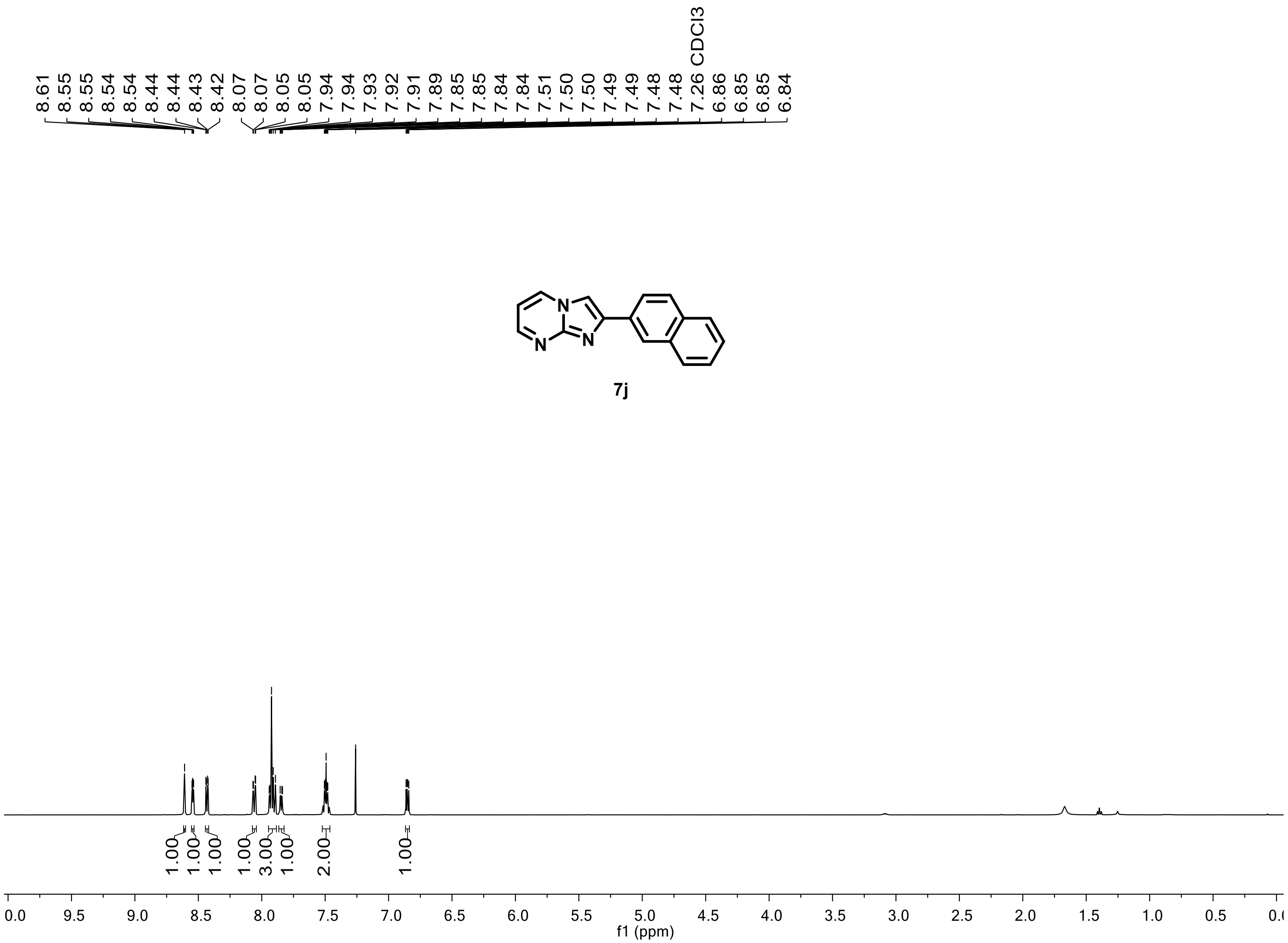

Molecule 7j: $^{13}C\{^{1}H\}$ NMR (125 MHz, $CDCl_3$)

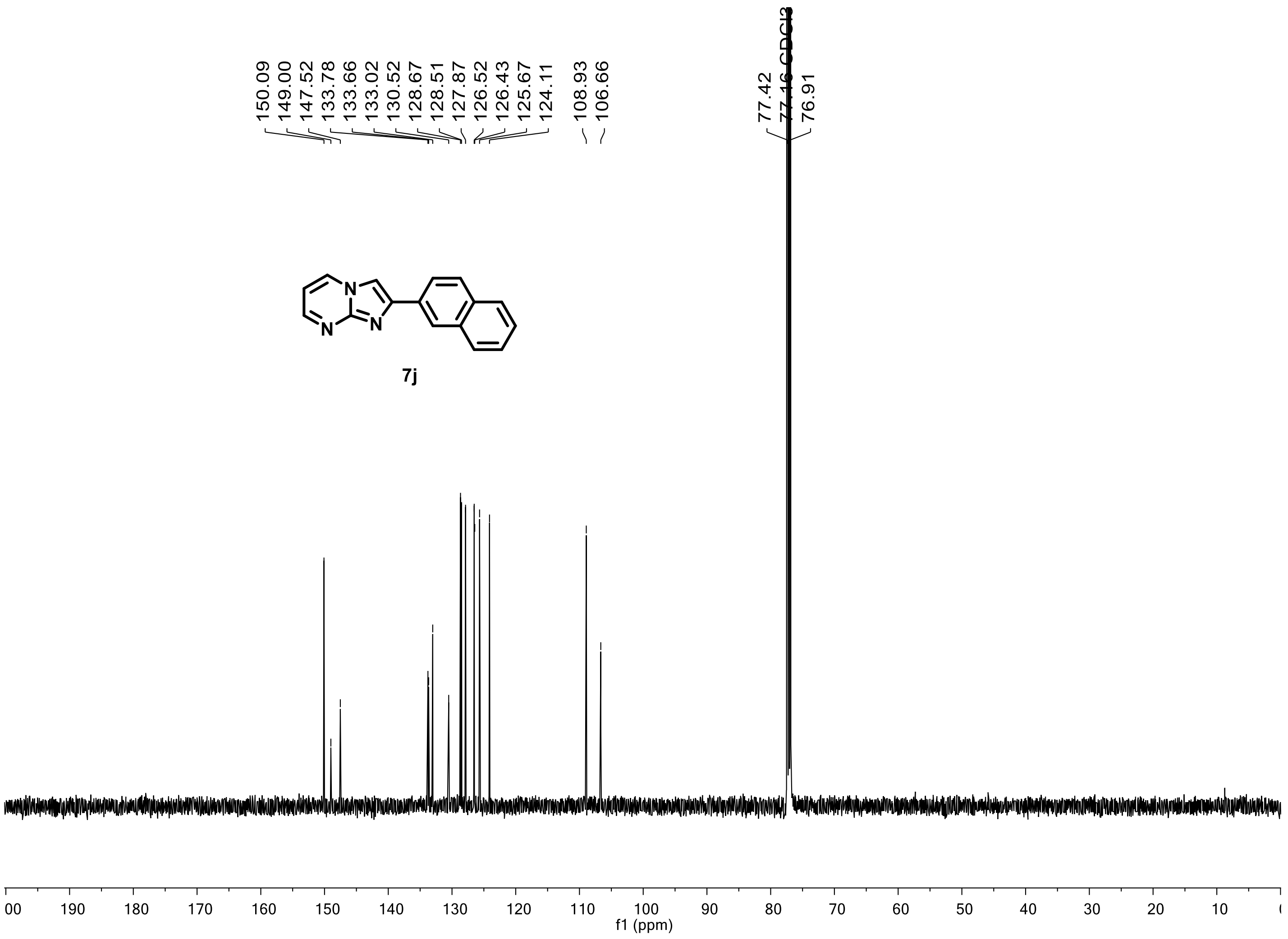

Molecule 7k: ^{1}H NMR (300 MHz, $CDCl_3$)

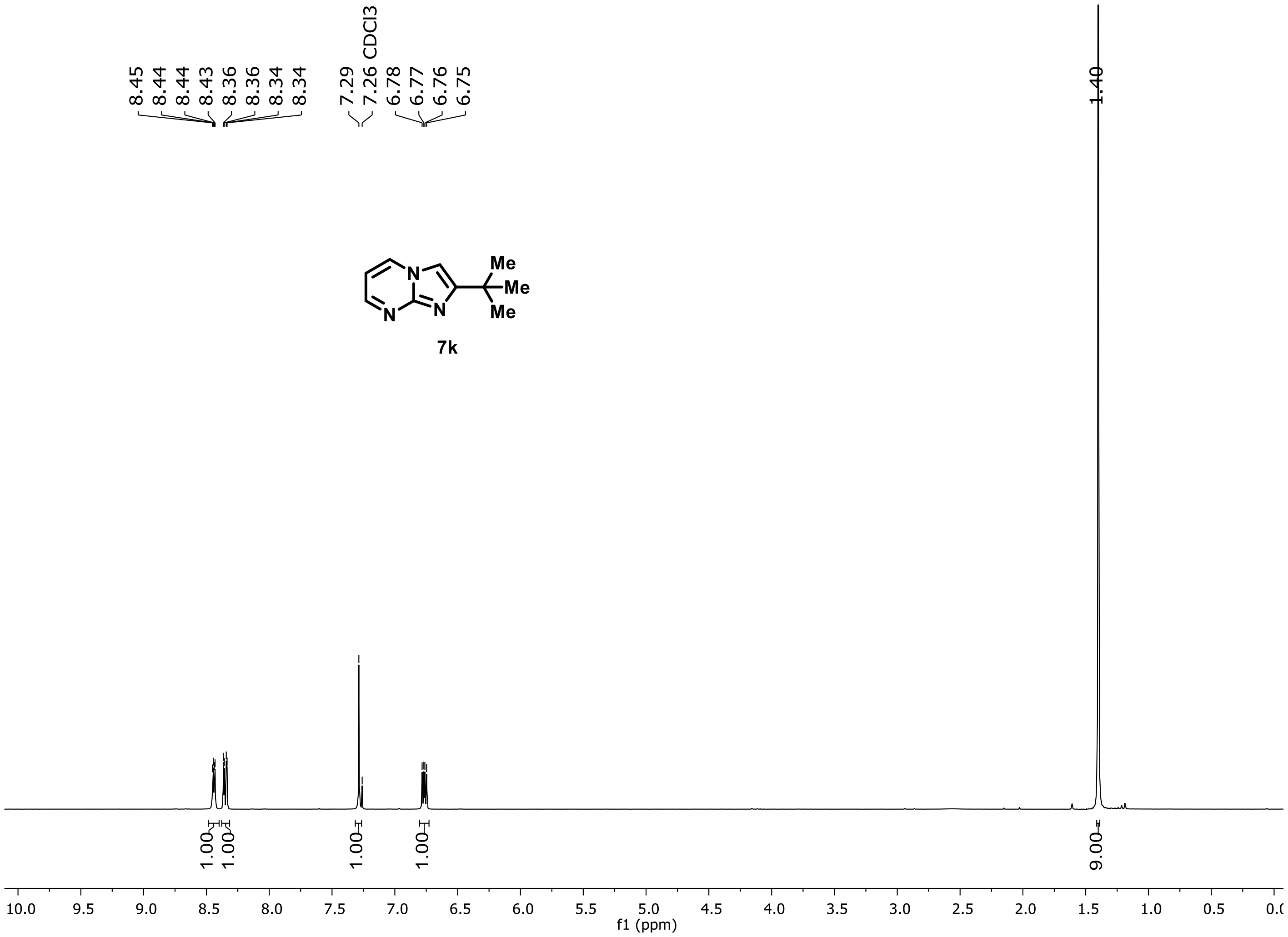

Molecule 7k: $^{13}C\{^{1}H\}$ NMR (75 MHz, $CDCl_3$)

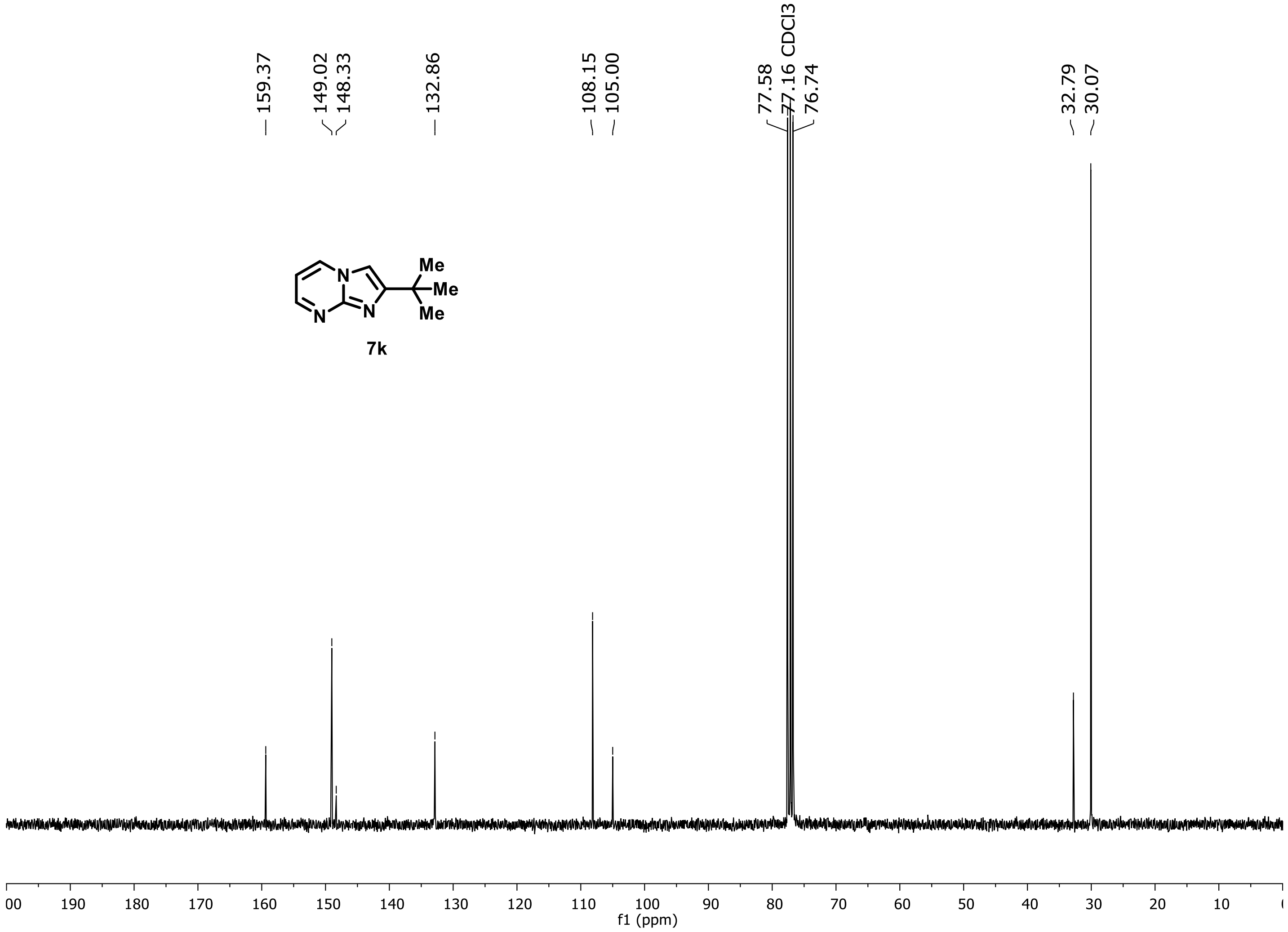

Molecule 7l: ^{1}H NMR (600 MHz, $CDCl_3$)

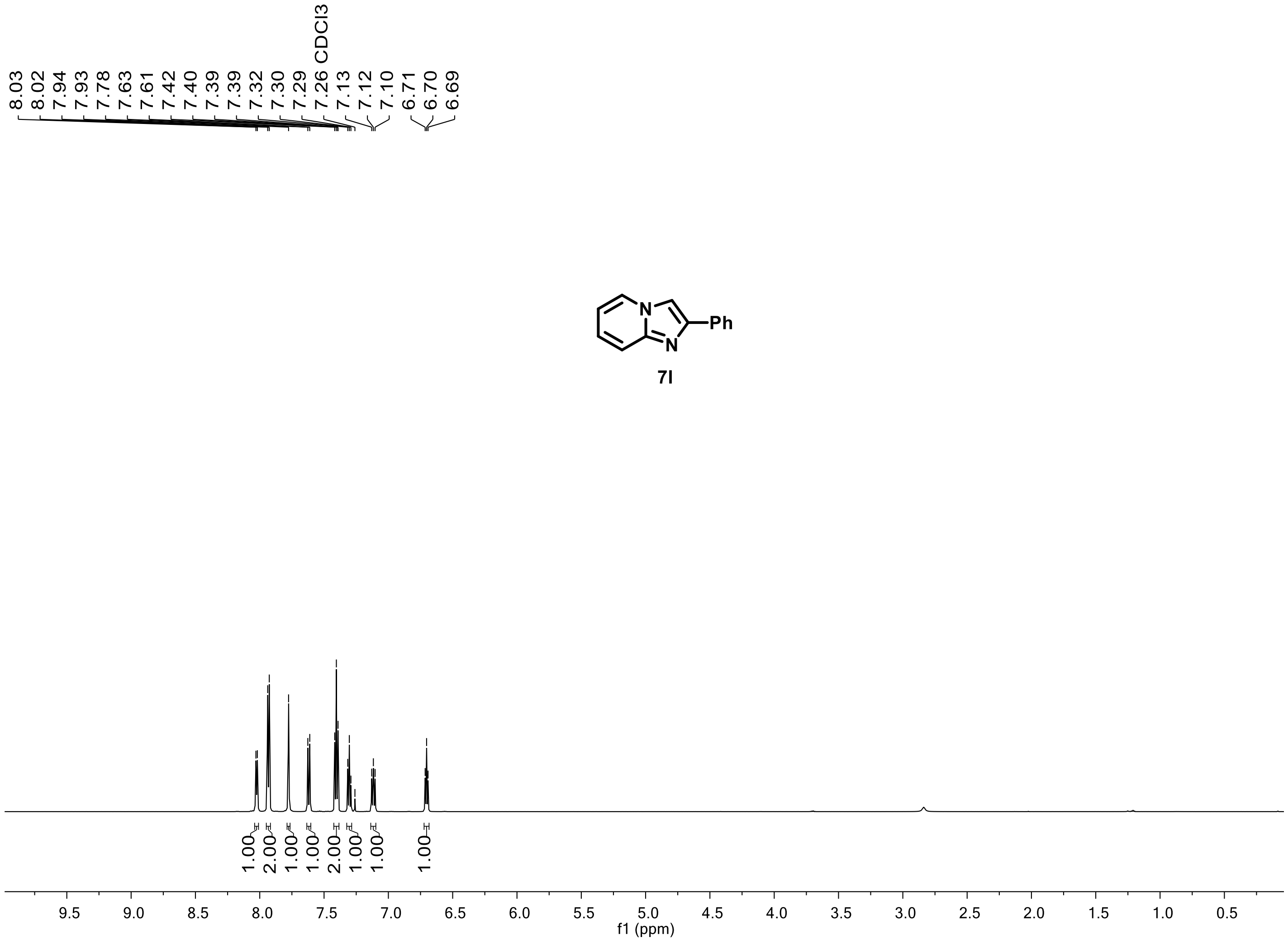

Molecule 7l: $^{13}C\{^{1}H\}$ NMR (150 MHz, $CDCl_3$)

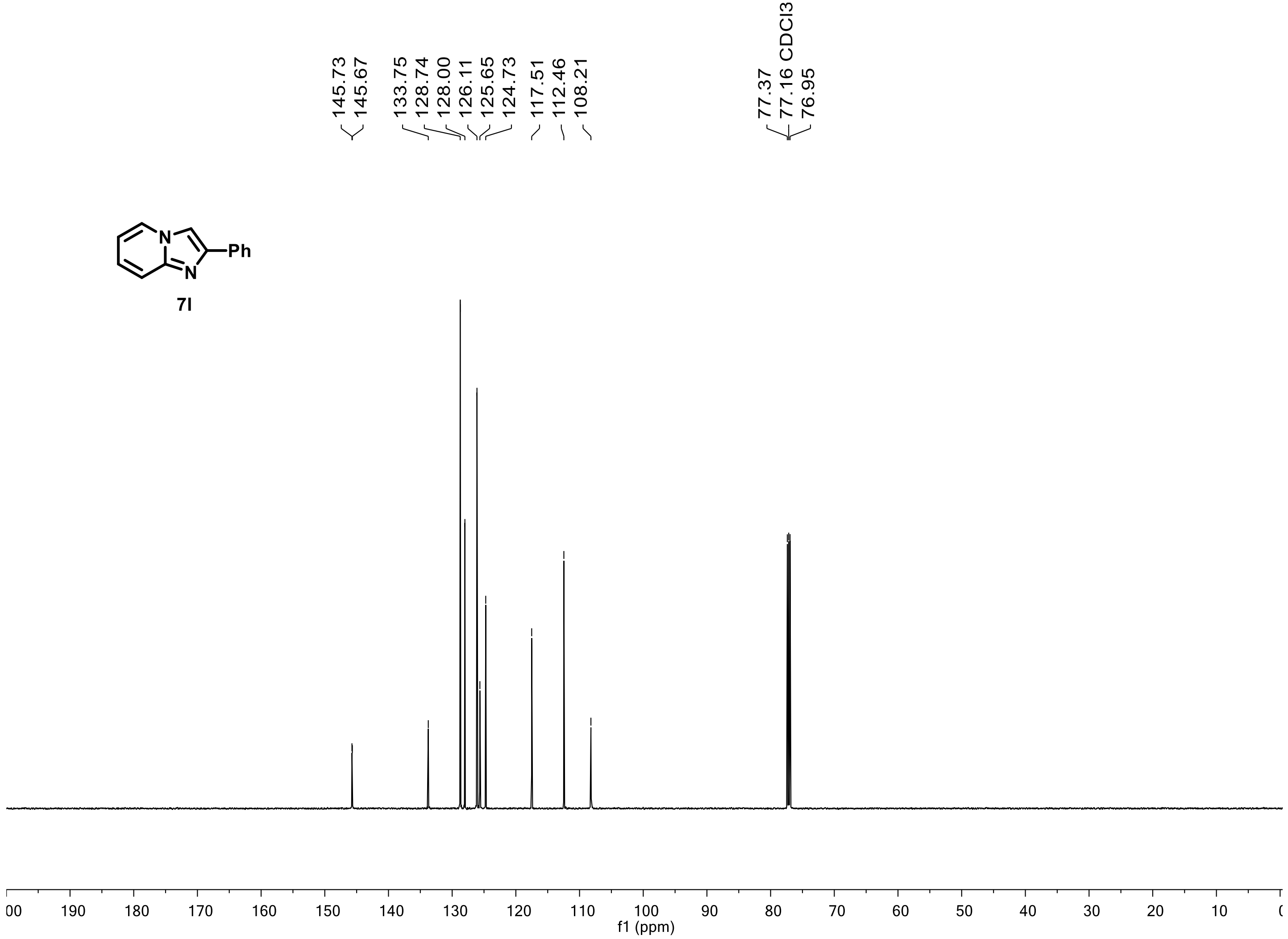

Molecule 7m: ^{1}H NMR (400 MHz, CDCl$_3$)

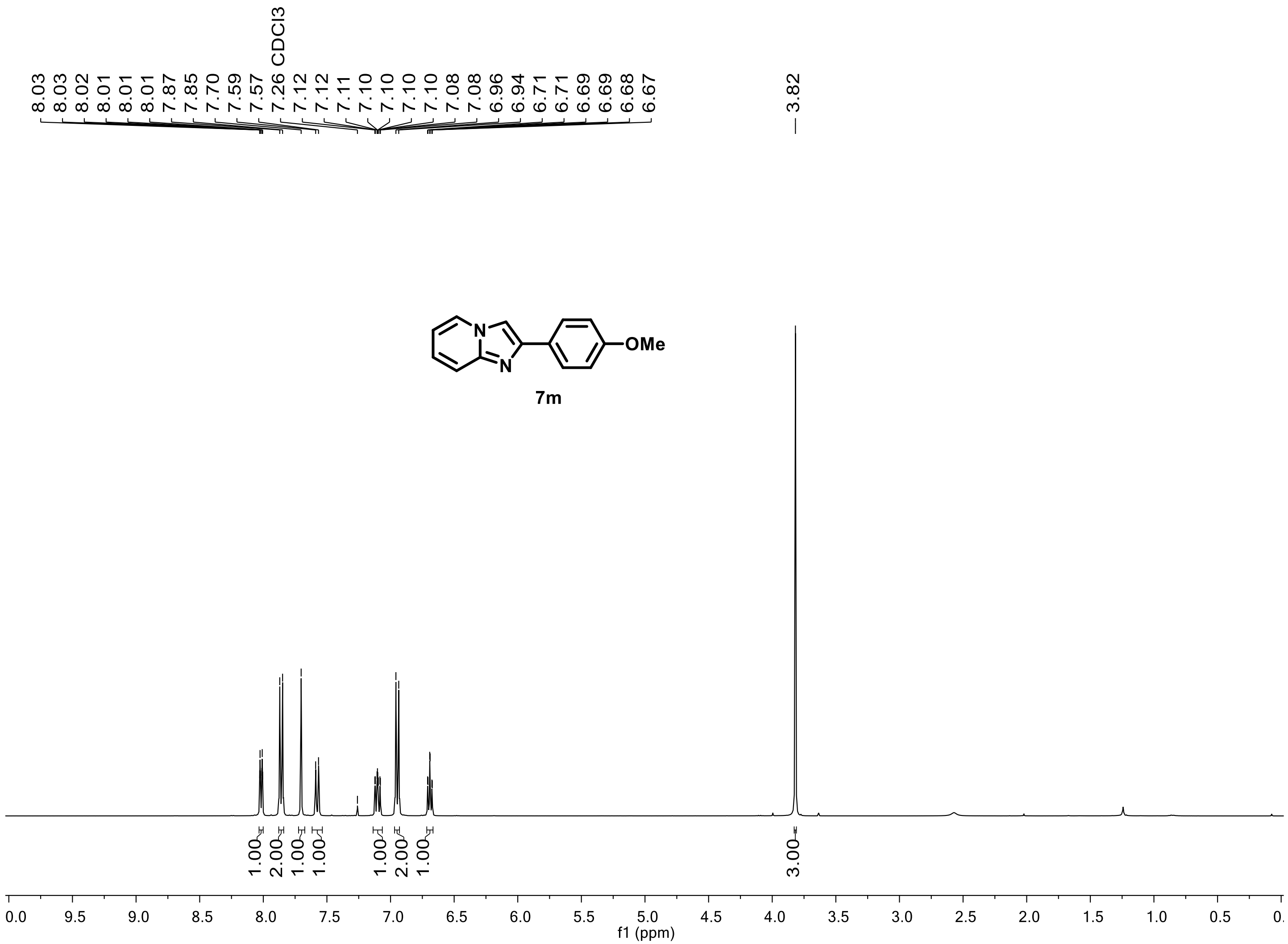

Molecule 7m: $^{13}C\{^{1}H\}$ NMR (100 MHz, $CDCl_3$)

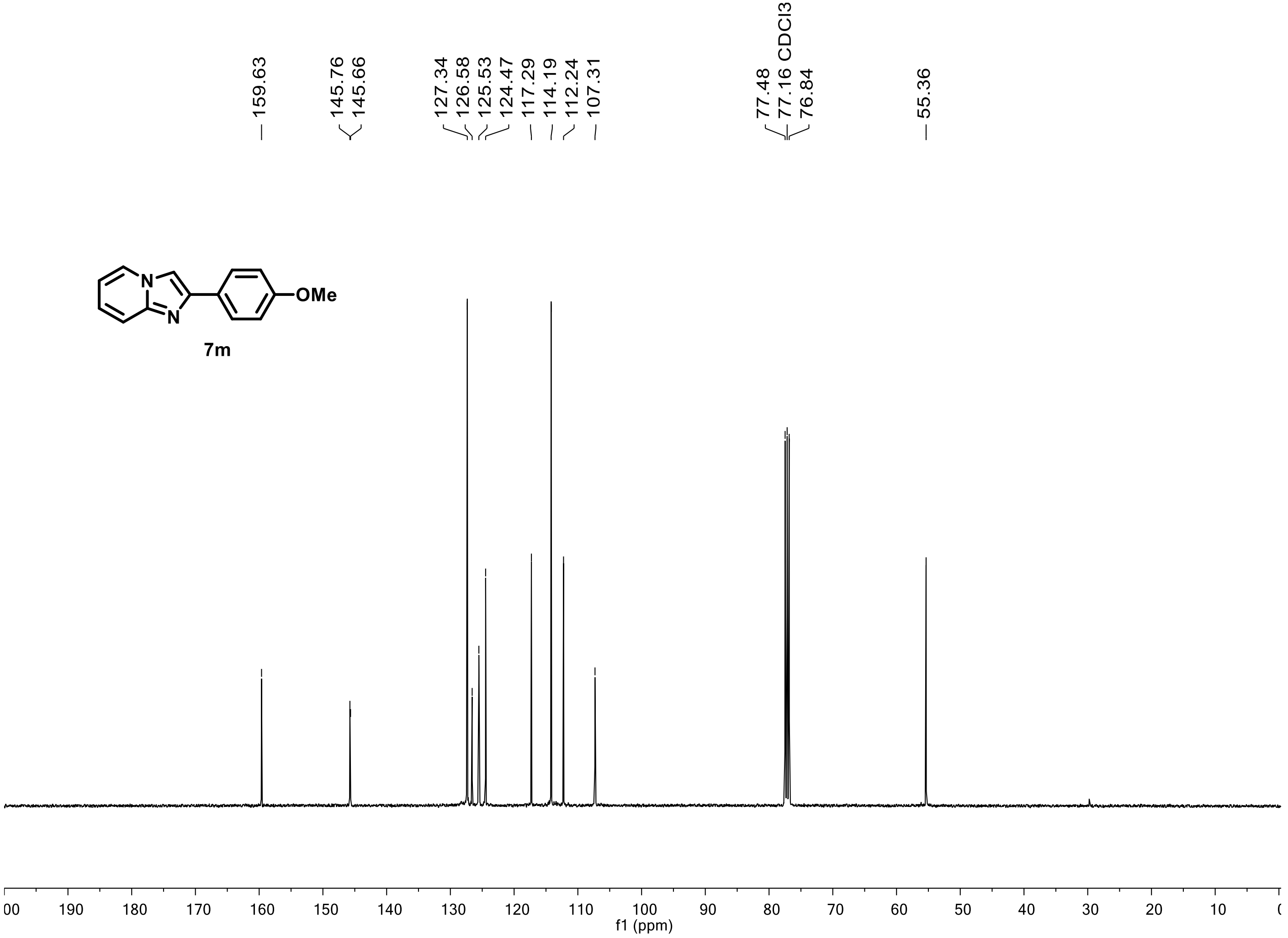

Molecule 7n: ^{1}H NMR (400 MHz, $CDCl_3$)

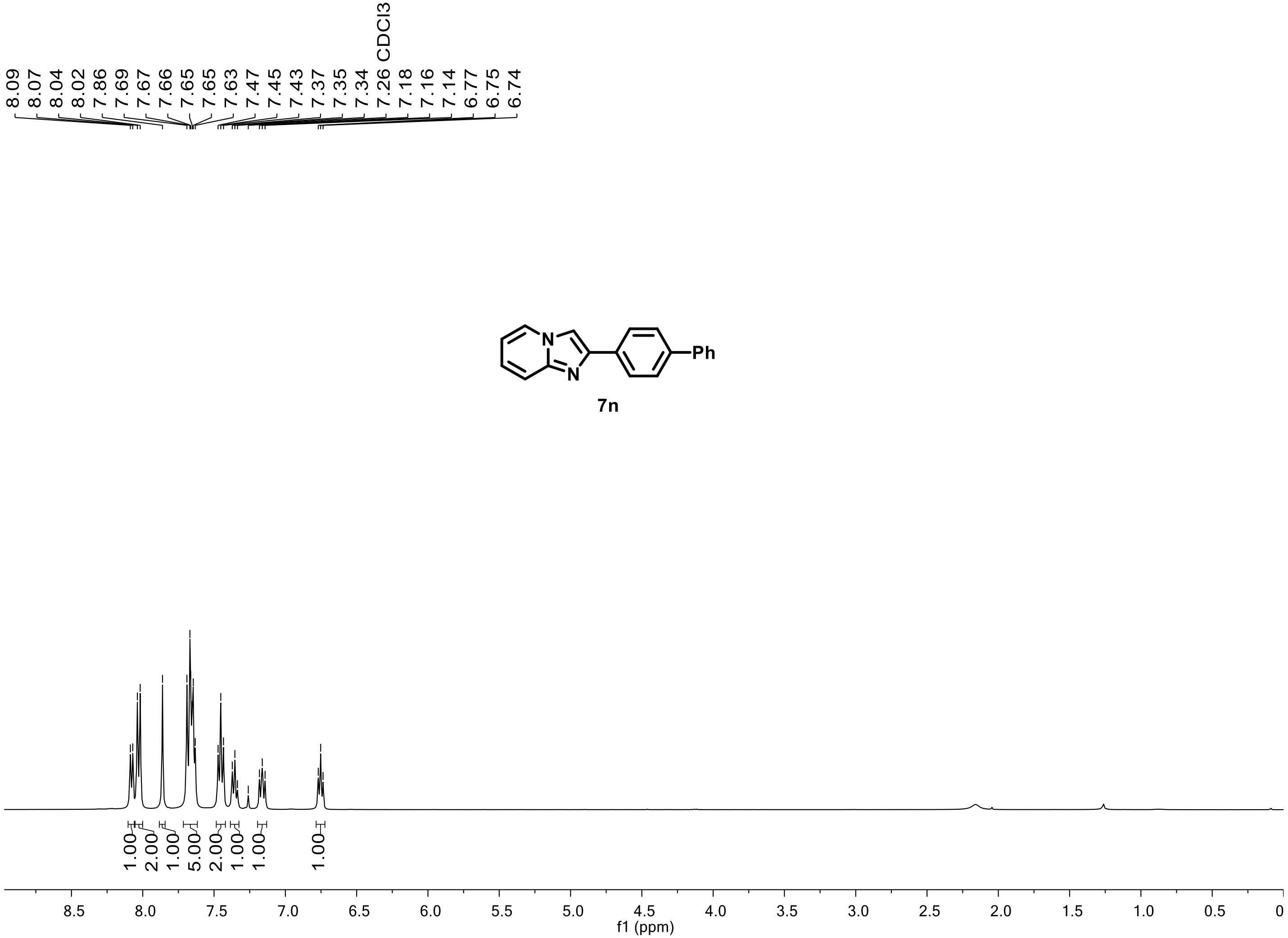

7n

Molecule 7n: $^{13}C\{^{1}H\}$ NMR (100 MHz, CDCl$_3$)

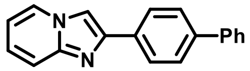

7n

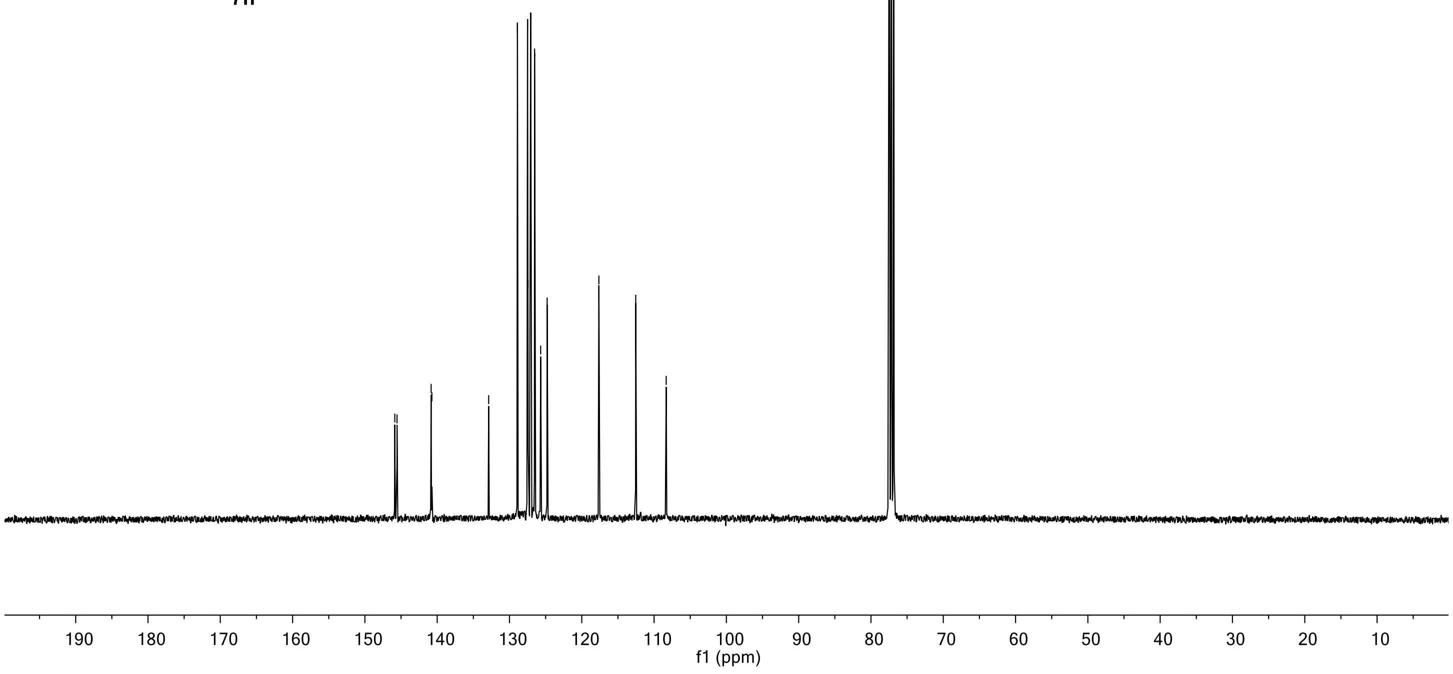

Molecule 7o: ^{1}H NMR (600 MHz, d_6-DMSO)

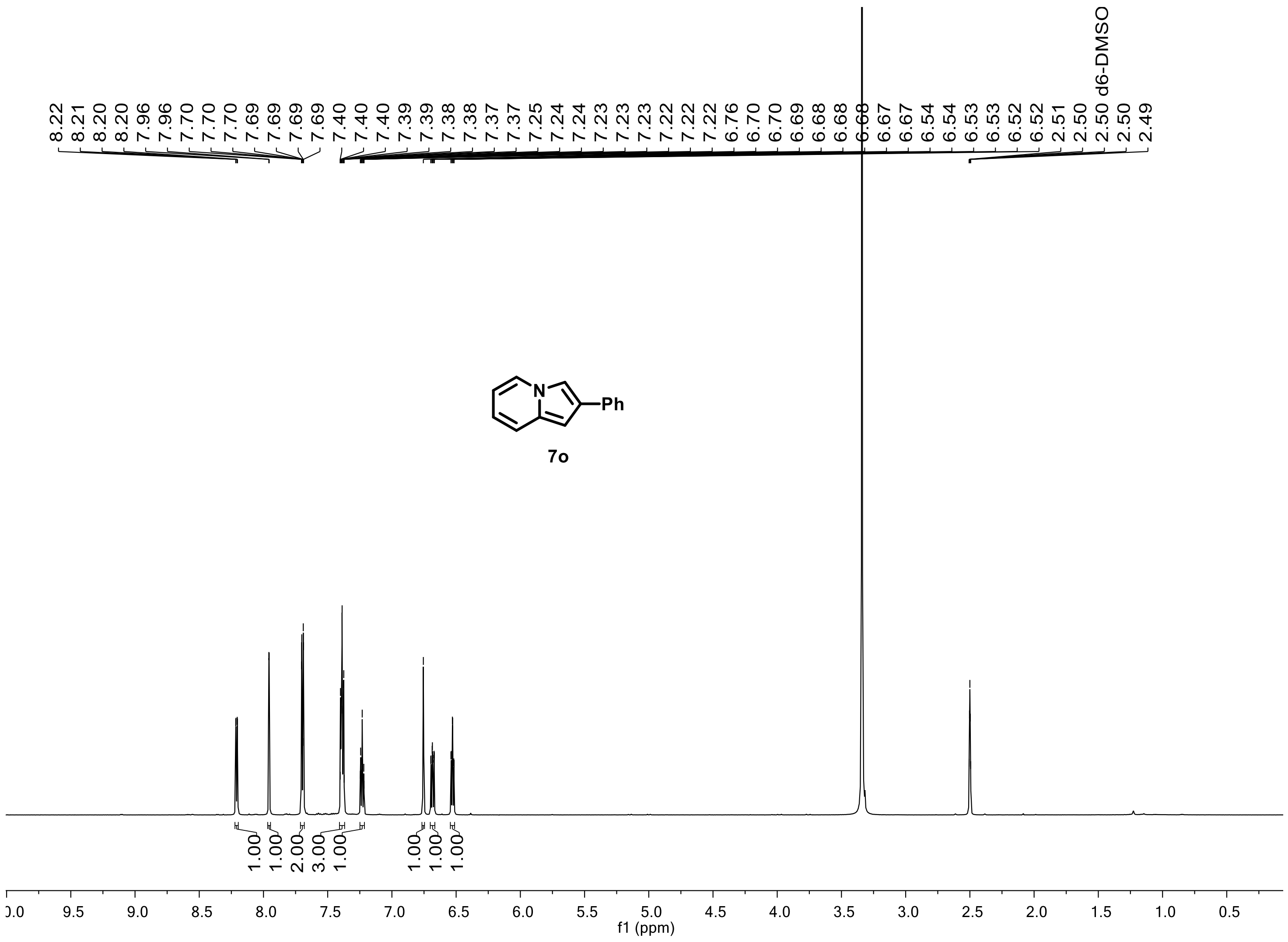

Molecule 7o: $^{13}C\{^{1}H\}$ NMR (150 MHz, d_6-DMSO)

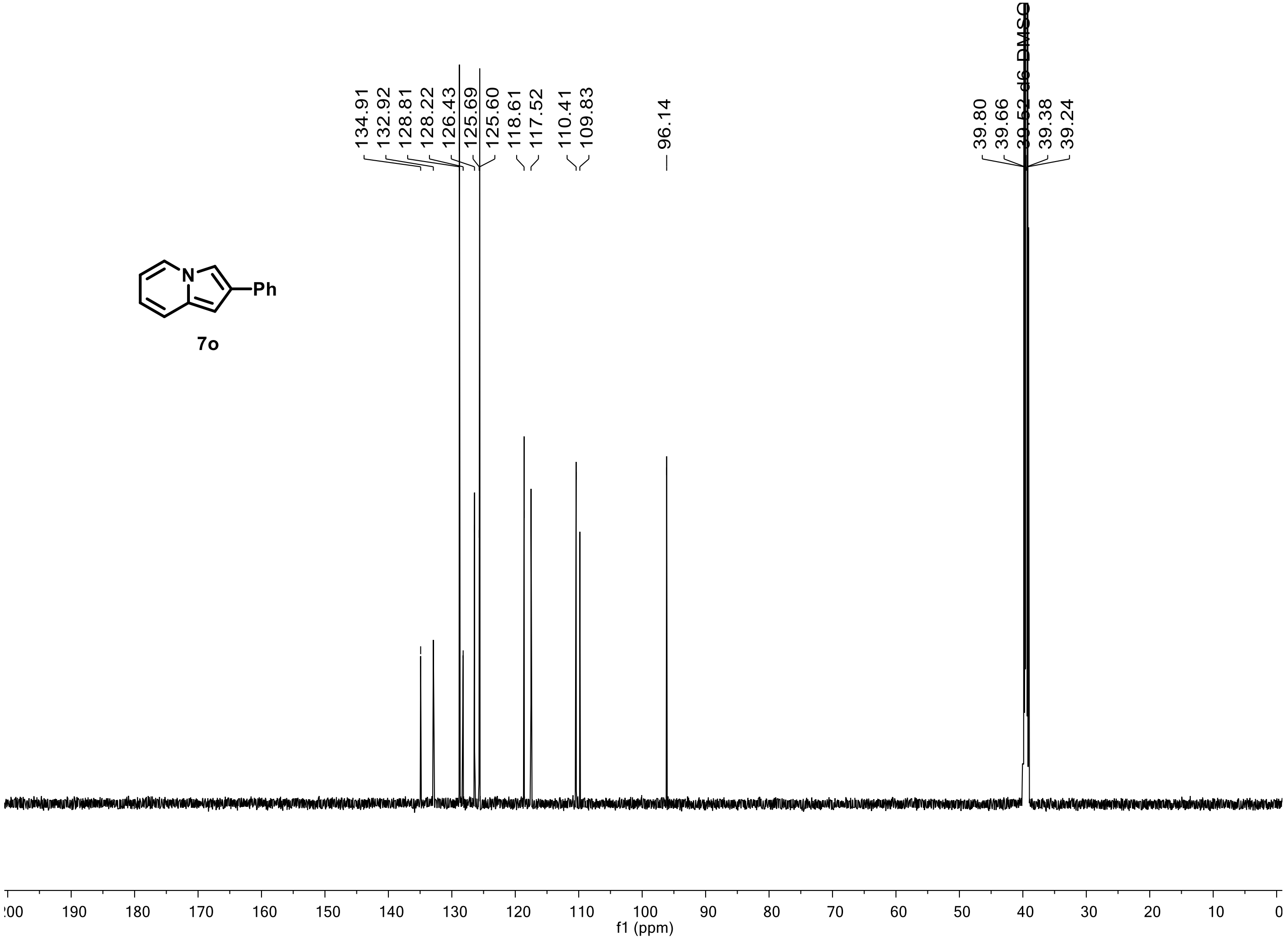

Molecule 7p: ^{1}H NMR (600 MHz, $CDCl_3$)

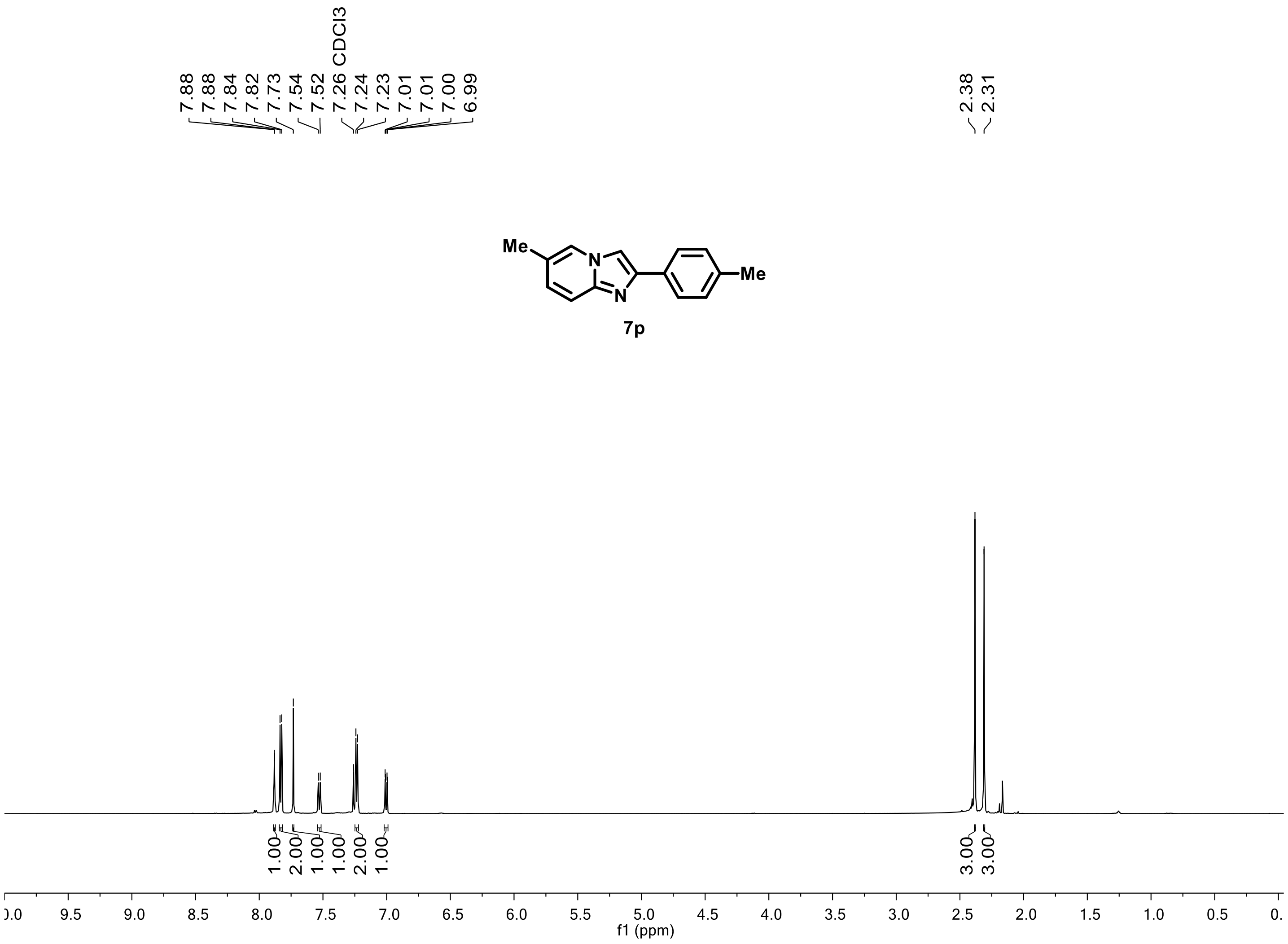

Molecule 7p: $^{13}C\{^{1}H\}$ NMR (150 MHz, $CDCl_3$)

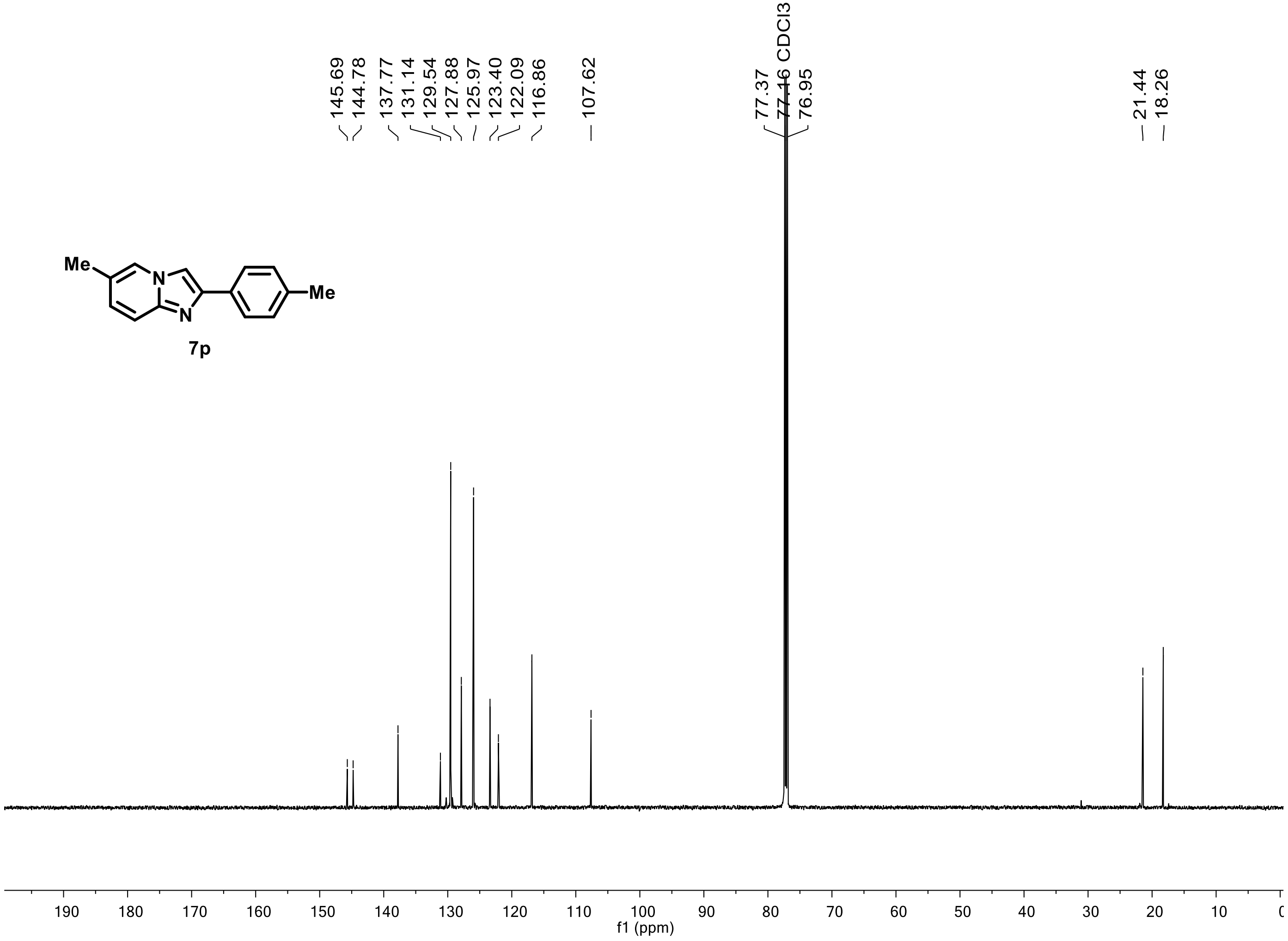

Molecule pre-8m: ^{1}H NMR (600 MHz, $CDCl_3$)

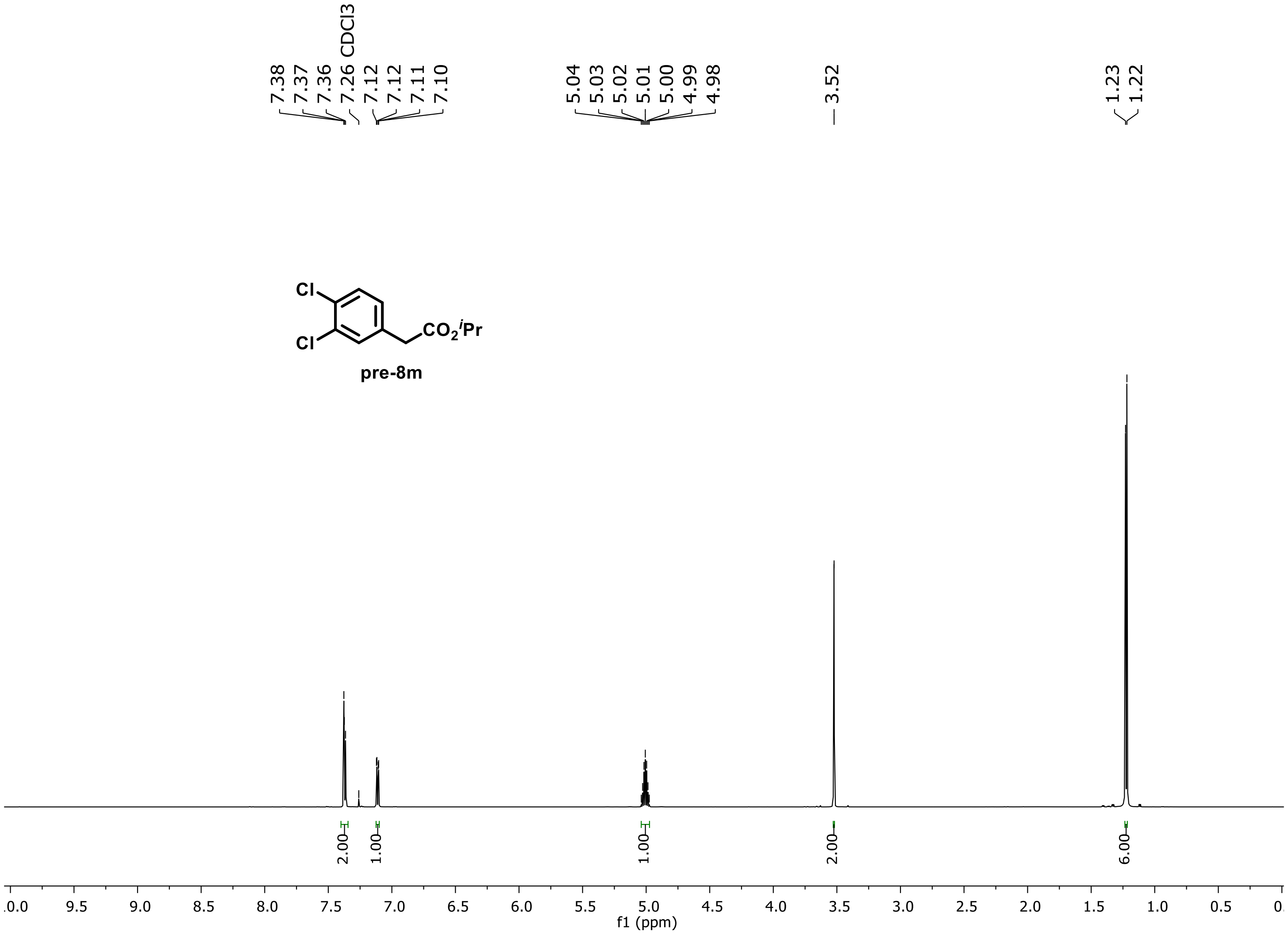

Molecule pre-8m: $^{13}C\{^1H\}$ NMR (150 MHz, $CDCl_3$)

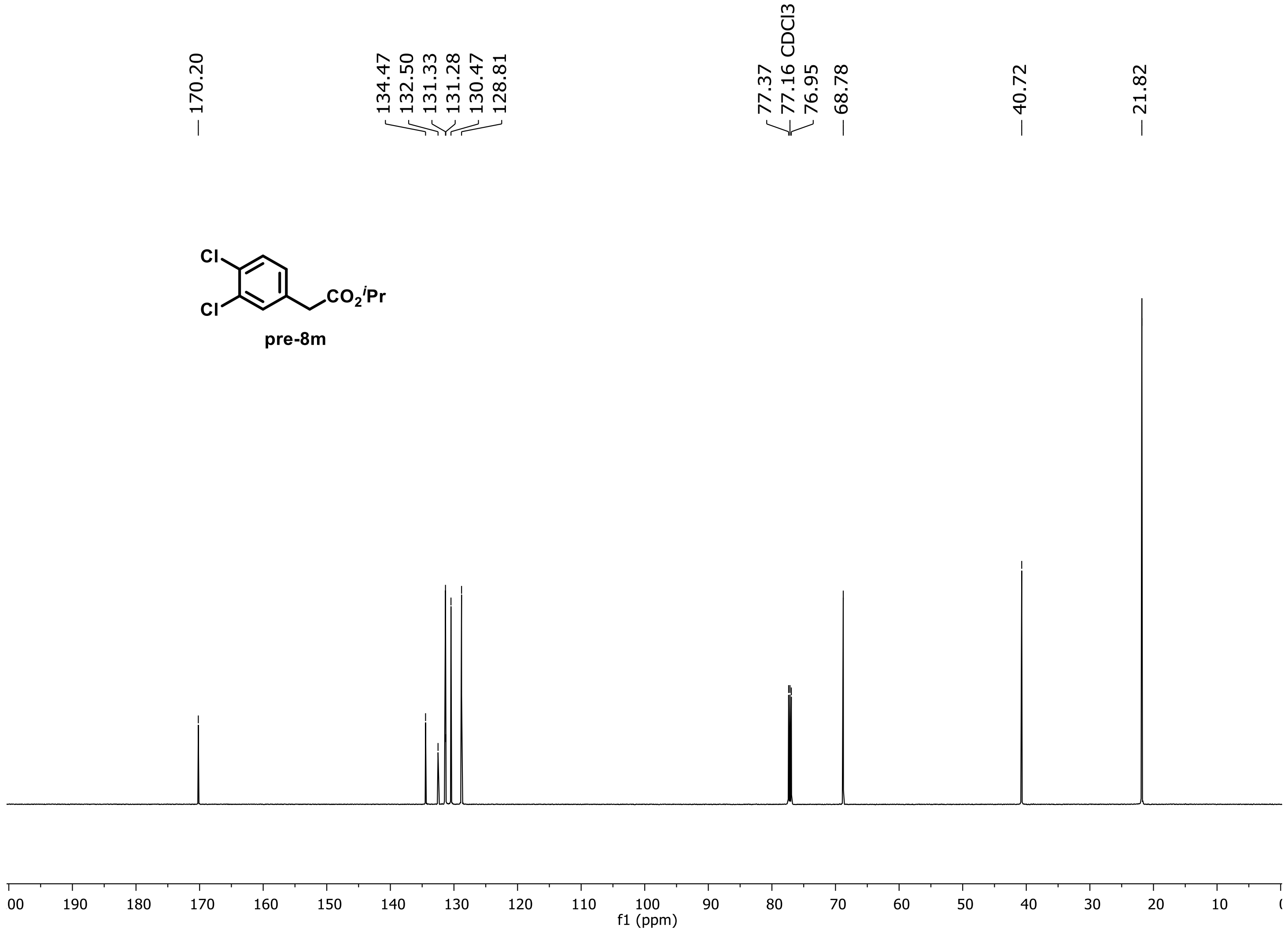

Molecule 8m: ^{1}H NMR (600 MHz, $CDCl_3$)

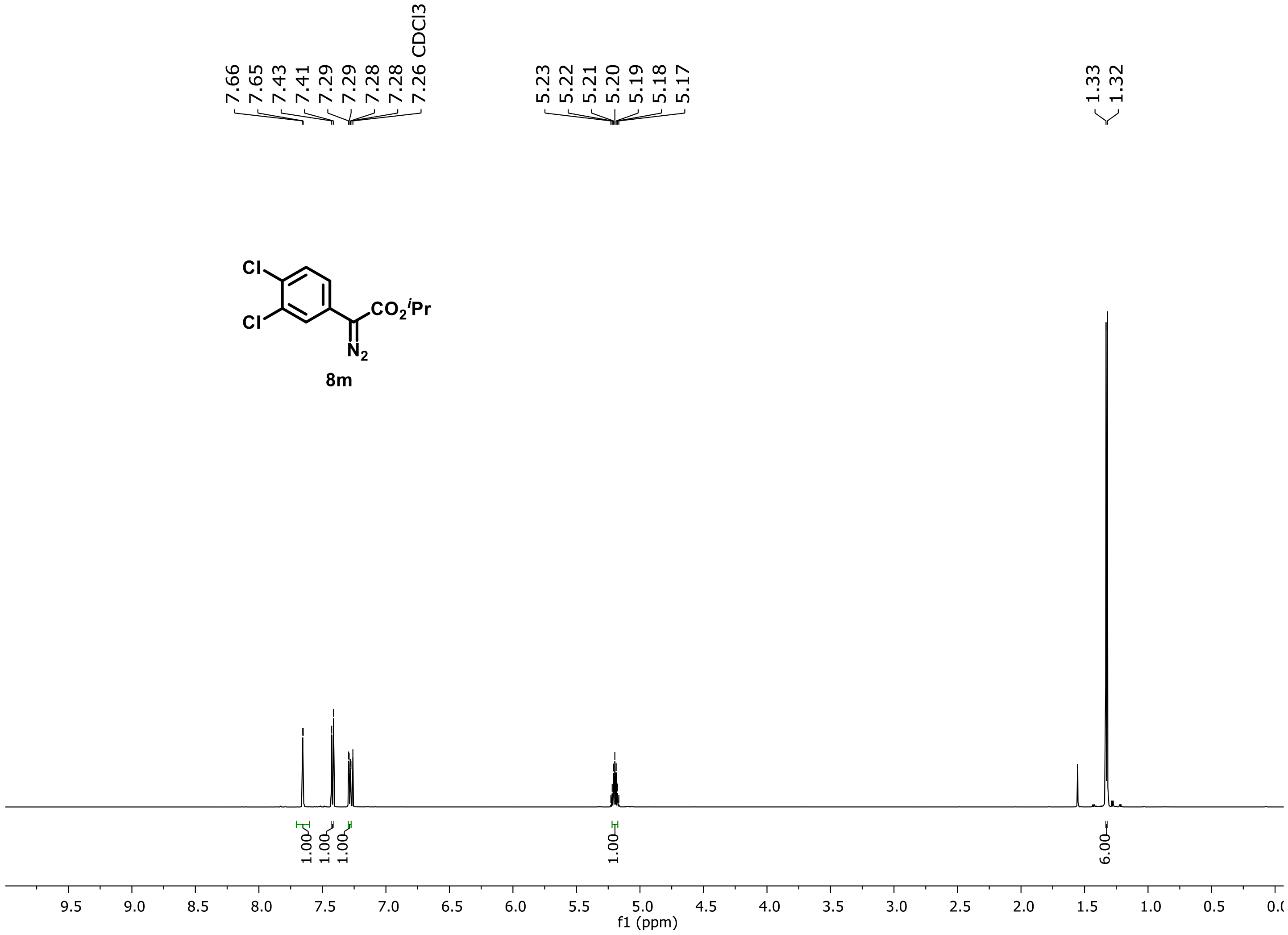

Molecule 8m: $^{13}C\{^1H\}$ NMR (150 MHz, $CDCl_3$)

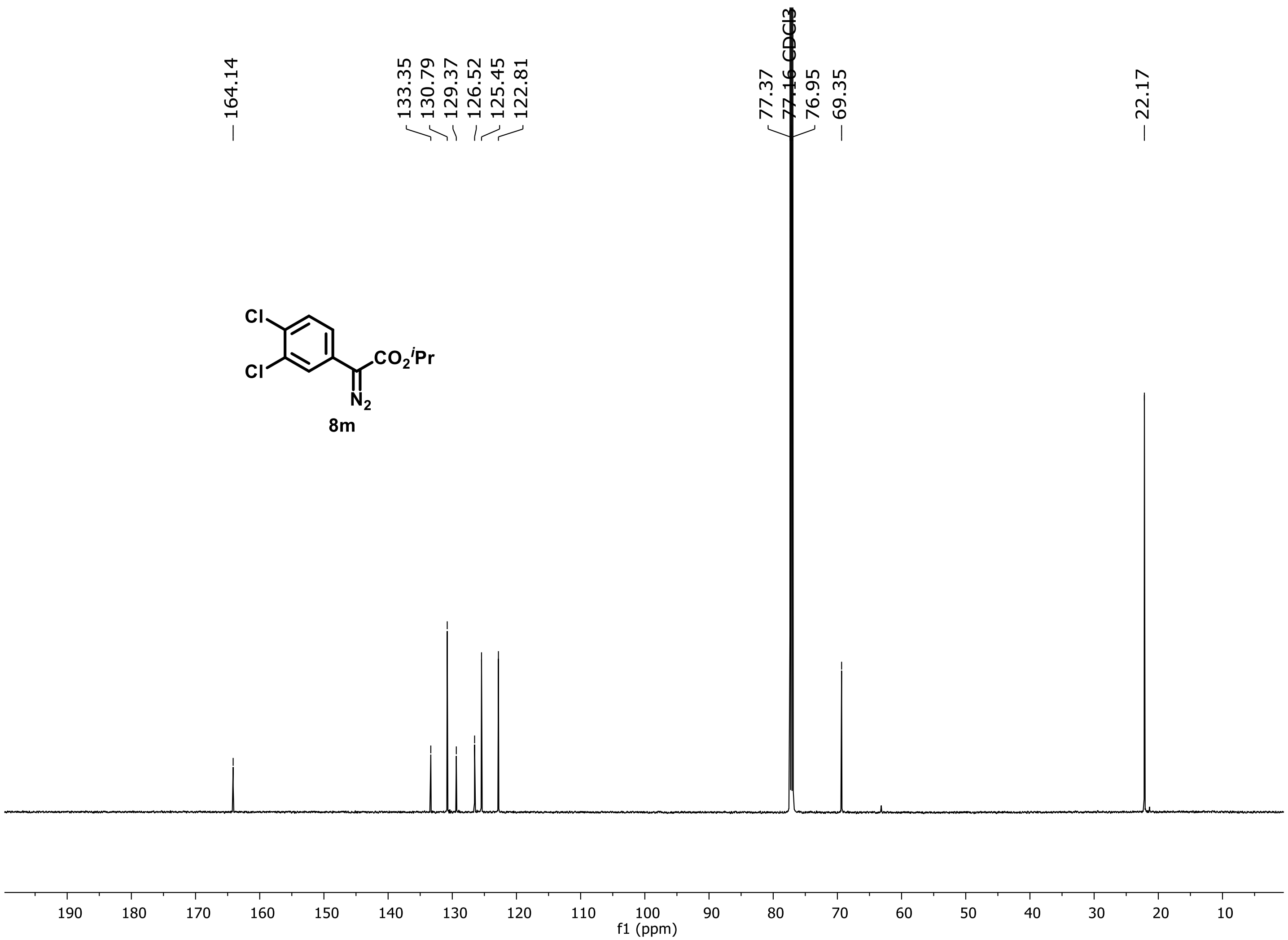

Molecule 9aa: ^{1}H NMR (500 MHz, $CDCl_3$)

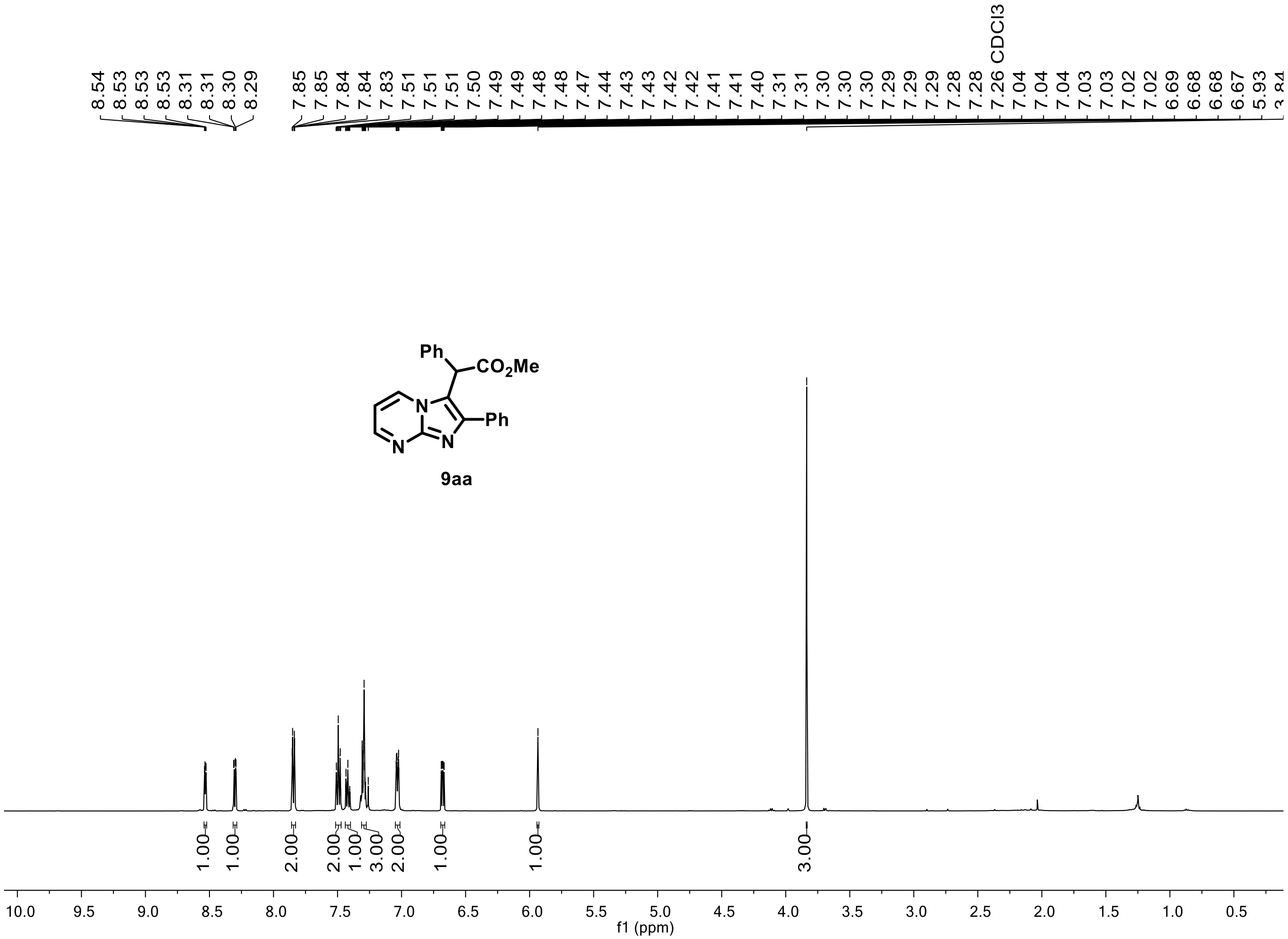

Molecule 9aa: $^{13}C\{^1H\}$ NMR (125 MHz, $CDCl_3$)

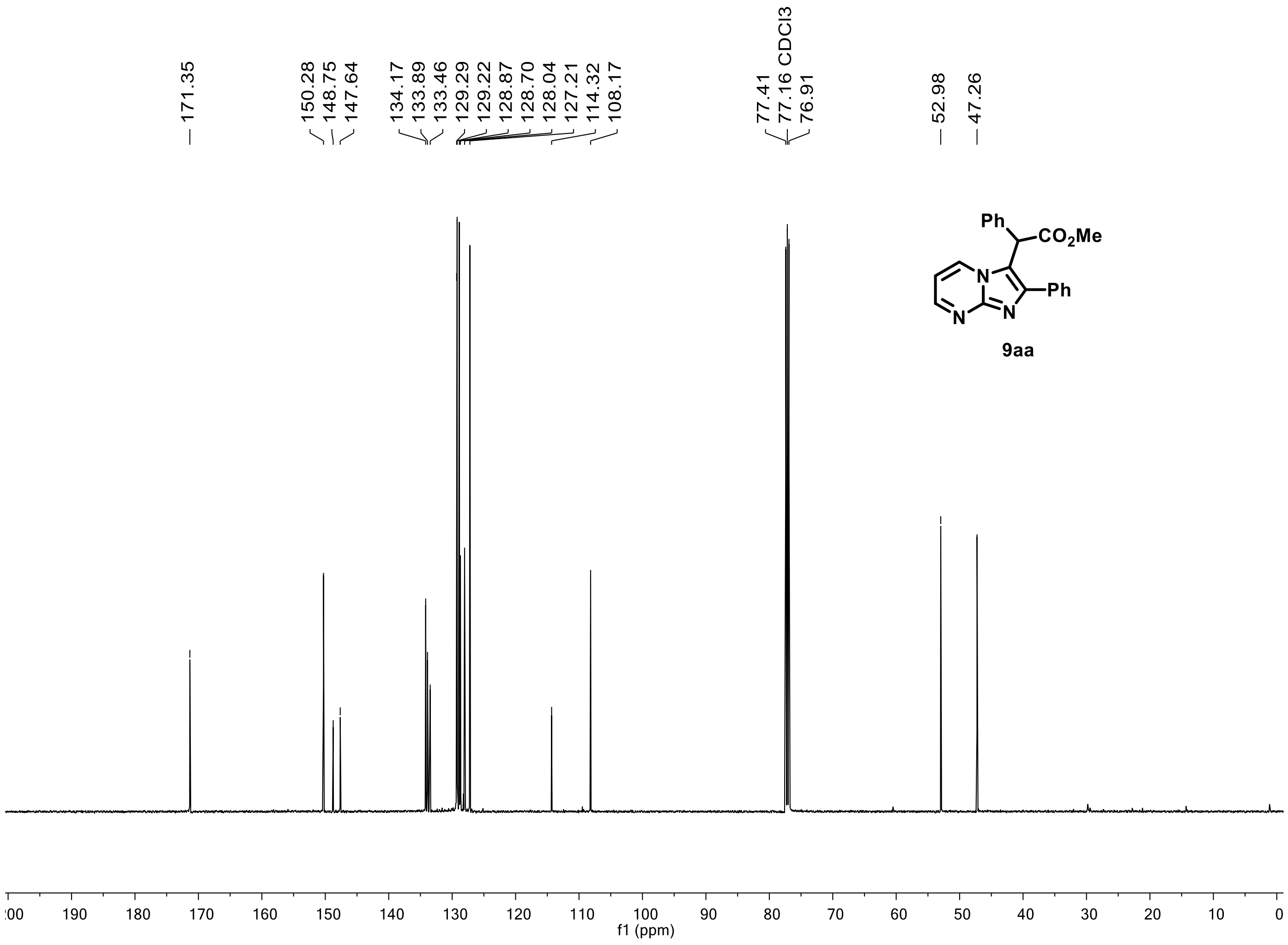

Molecule 9ba: ^{1}H NMR (250 MHz, $CDCl_3$)

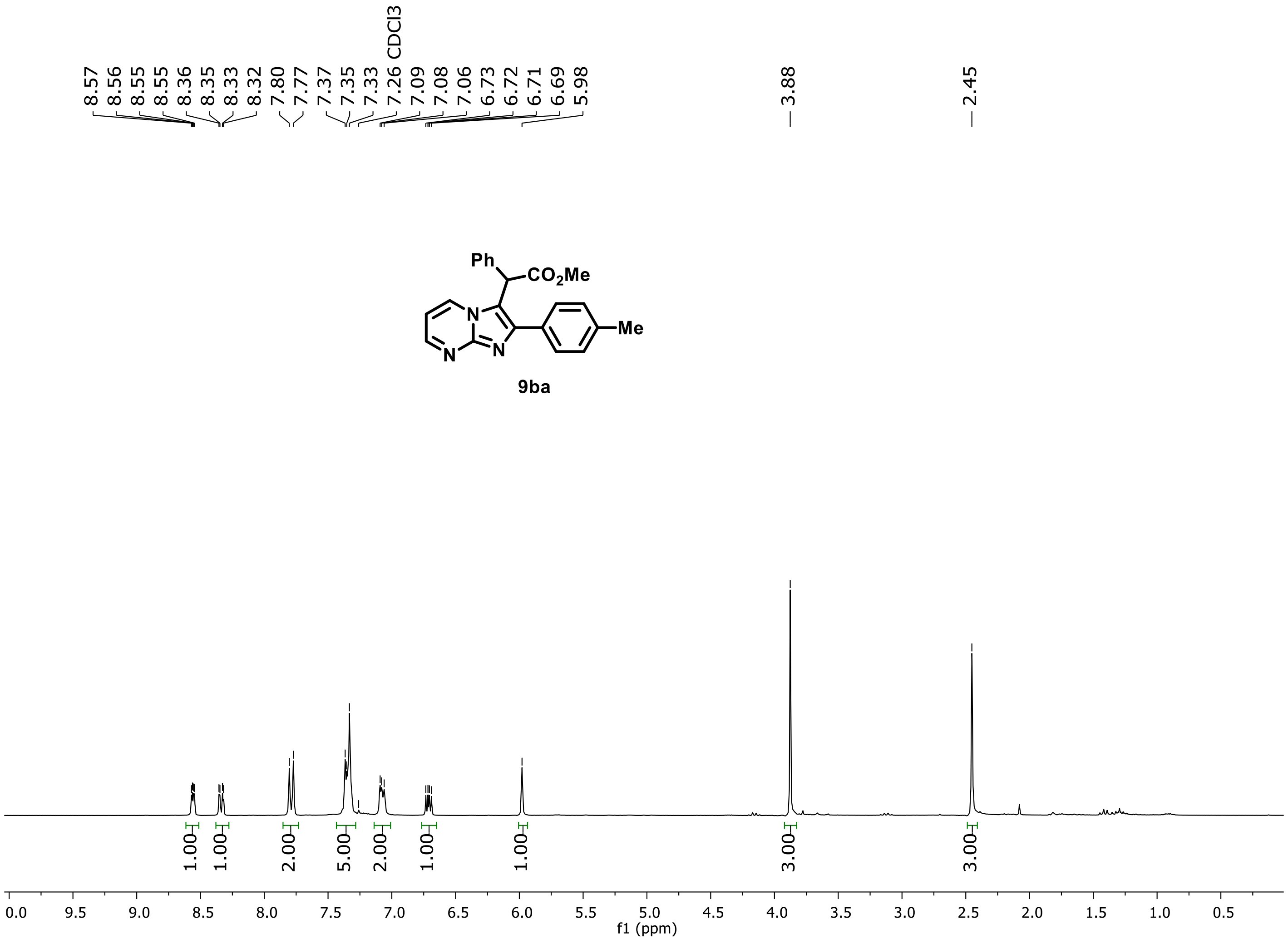

Molecule 9ba: $^{13}C\{^{1}H\}$ NMR (62.5 MHz, $CDCl_3$)

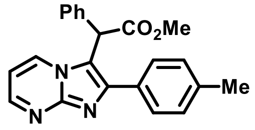

9ba

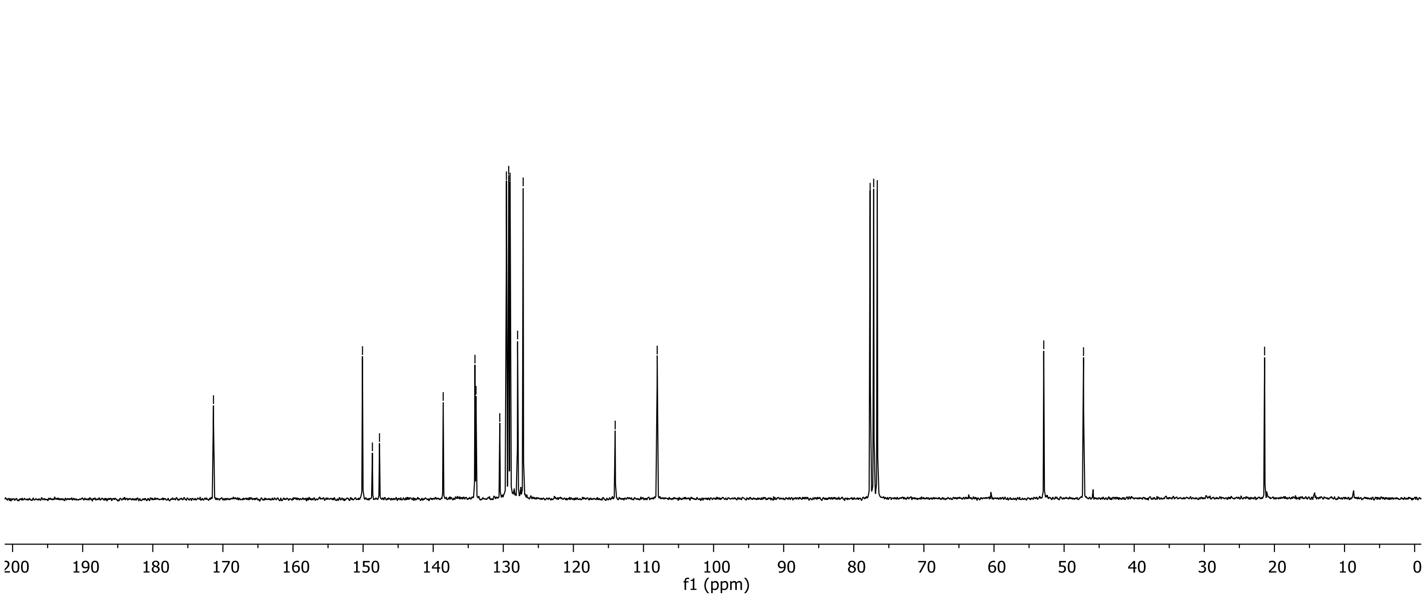

Molecule 9ca: ^{1}H NMR (500 MHz, $CDCl_3$)

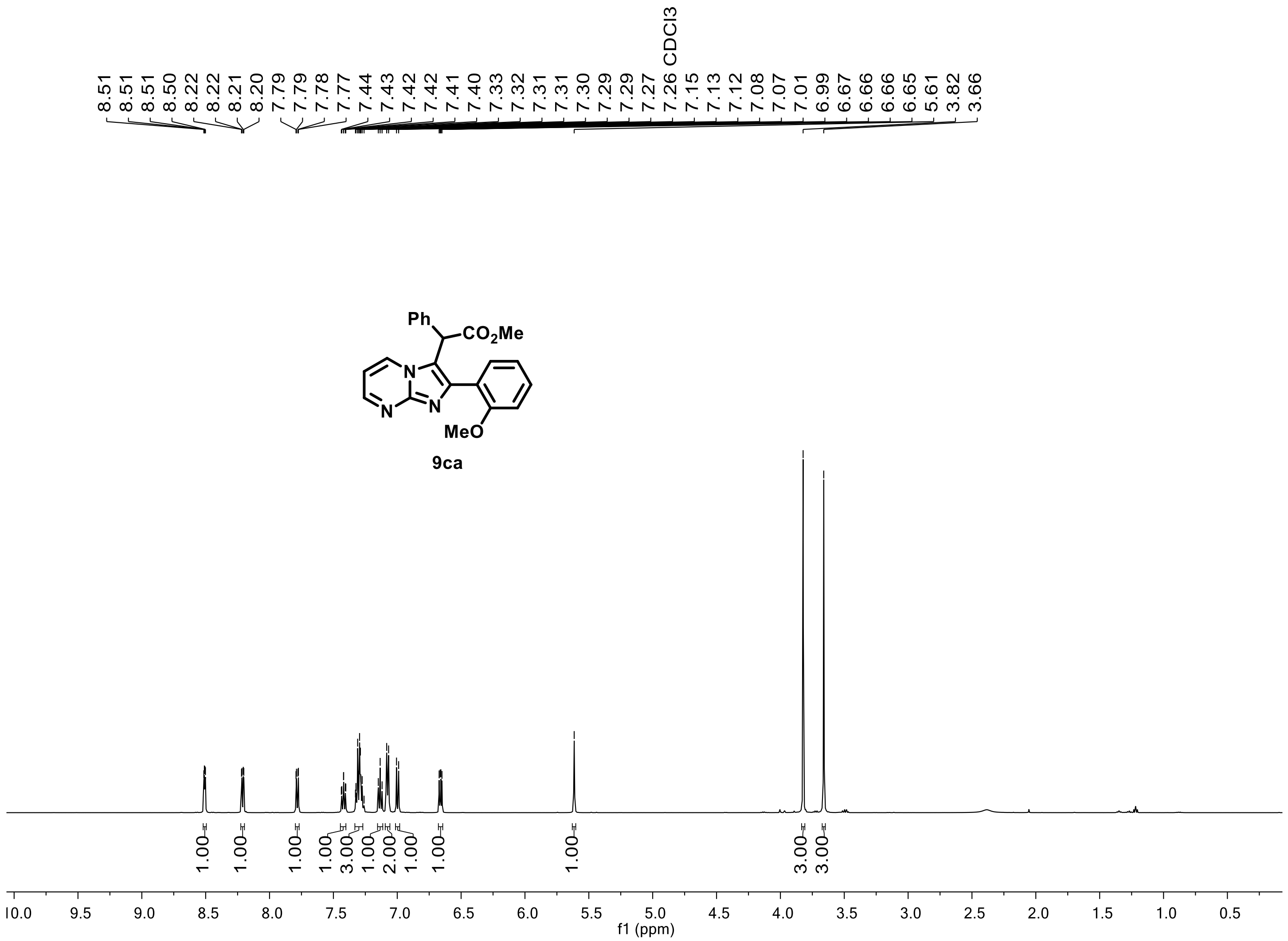

Molecule 9ca: $^{13}C\{^{1}H\}$ NMR (125 MHz, $CDCl_3$)

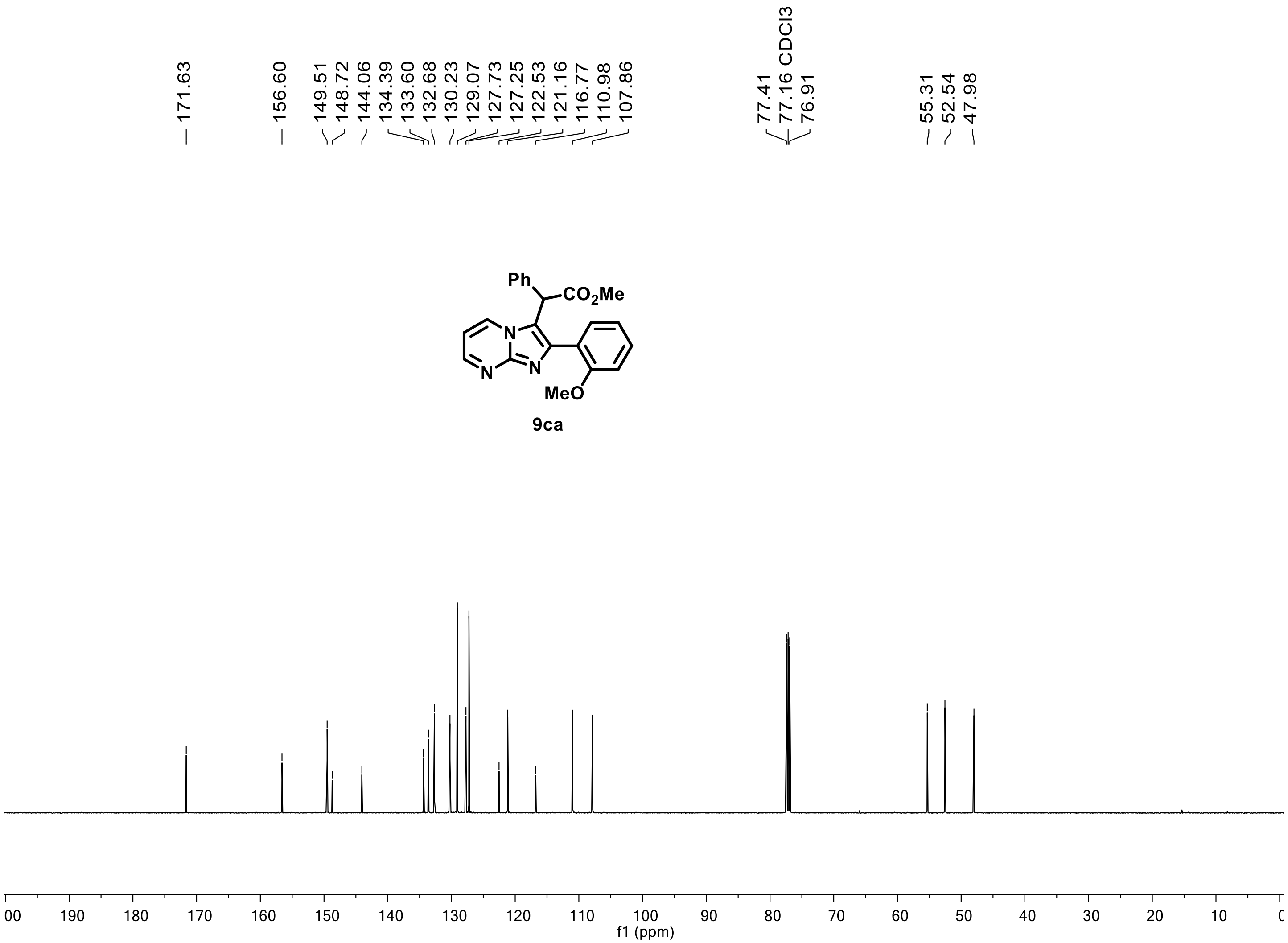

Molecule 9da: ^{1}H NMR (600 MHz, $CDCl_3$)

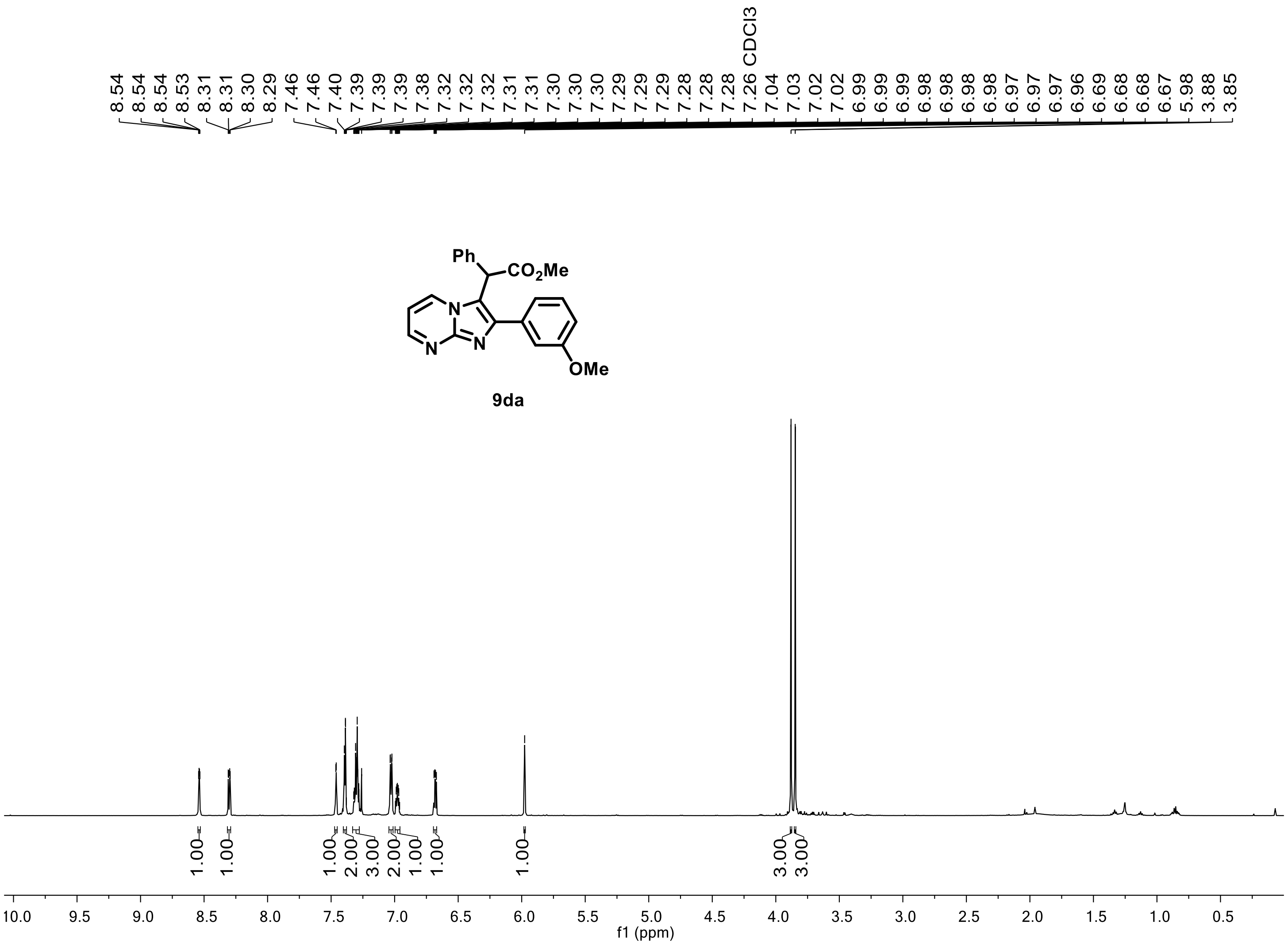

Molecule 9da: $^{13}C\{^1H\}$ NMR (150 MHz, $CDCl_3$)

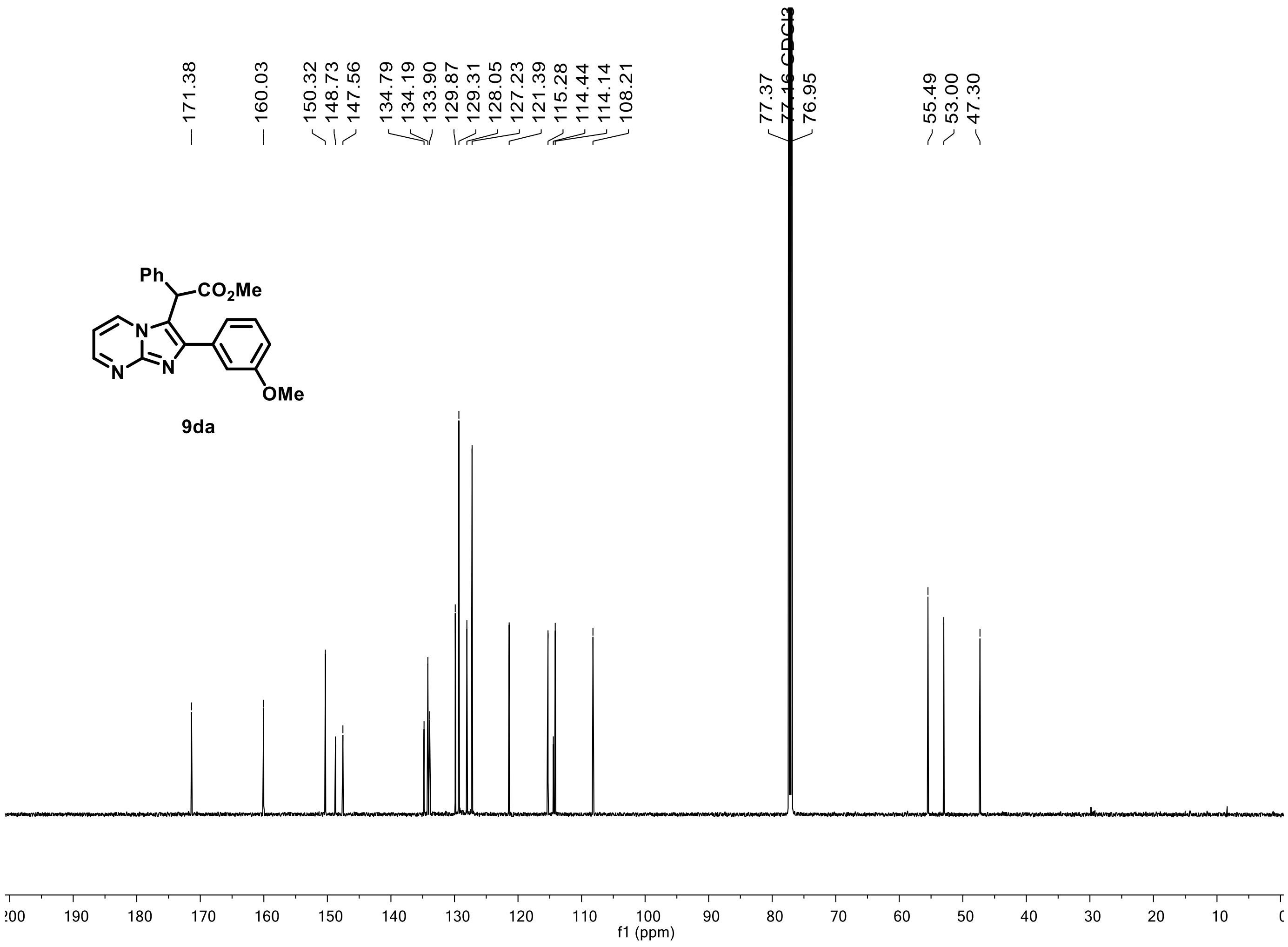

Molecule 9ea: ^{1}H NMR (250 MHz, CDCl$_3$)

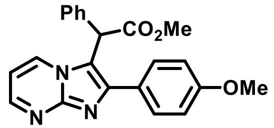

9ea

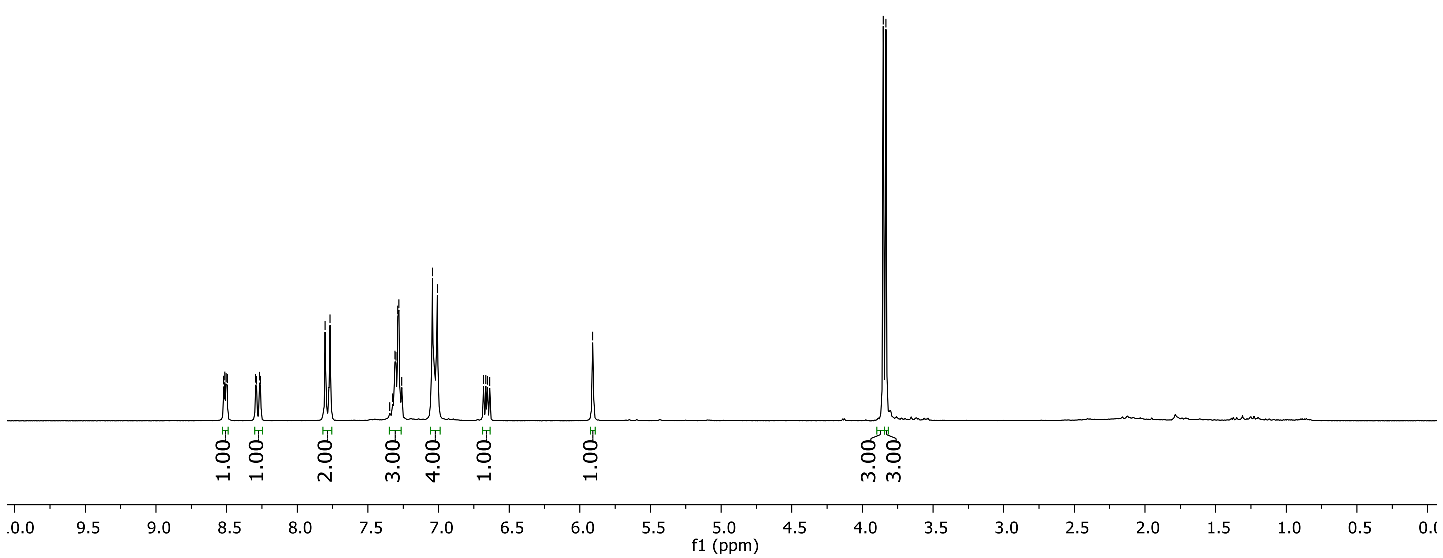

Molecule 9ea: $^{13}C\{^{1}H\}$ NMR (62.5 MHz, $CDCl_3$)

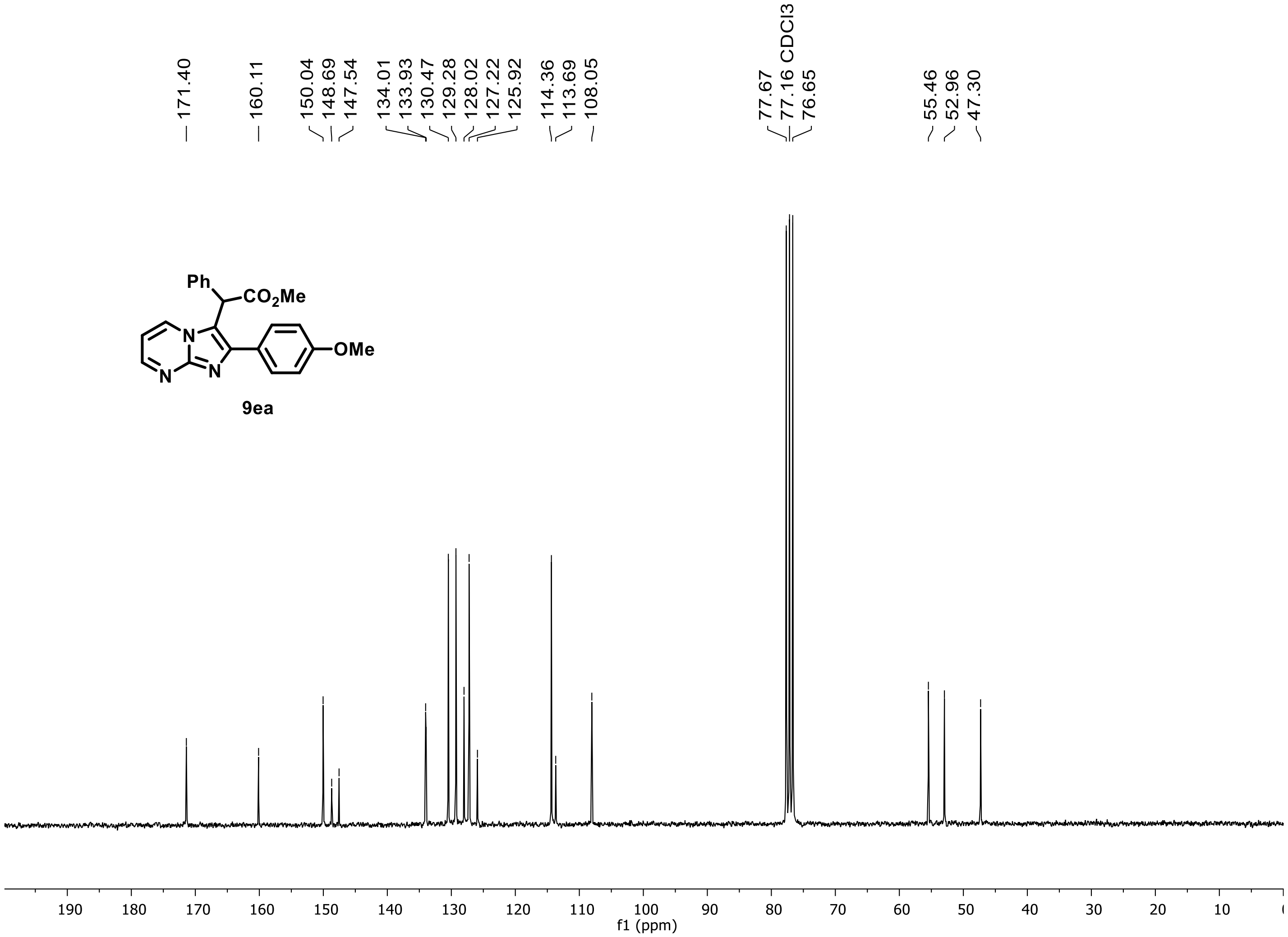

Molecule 9fa: ^{1}H NMR (500 MHz, $CDCl_3$)

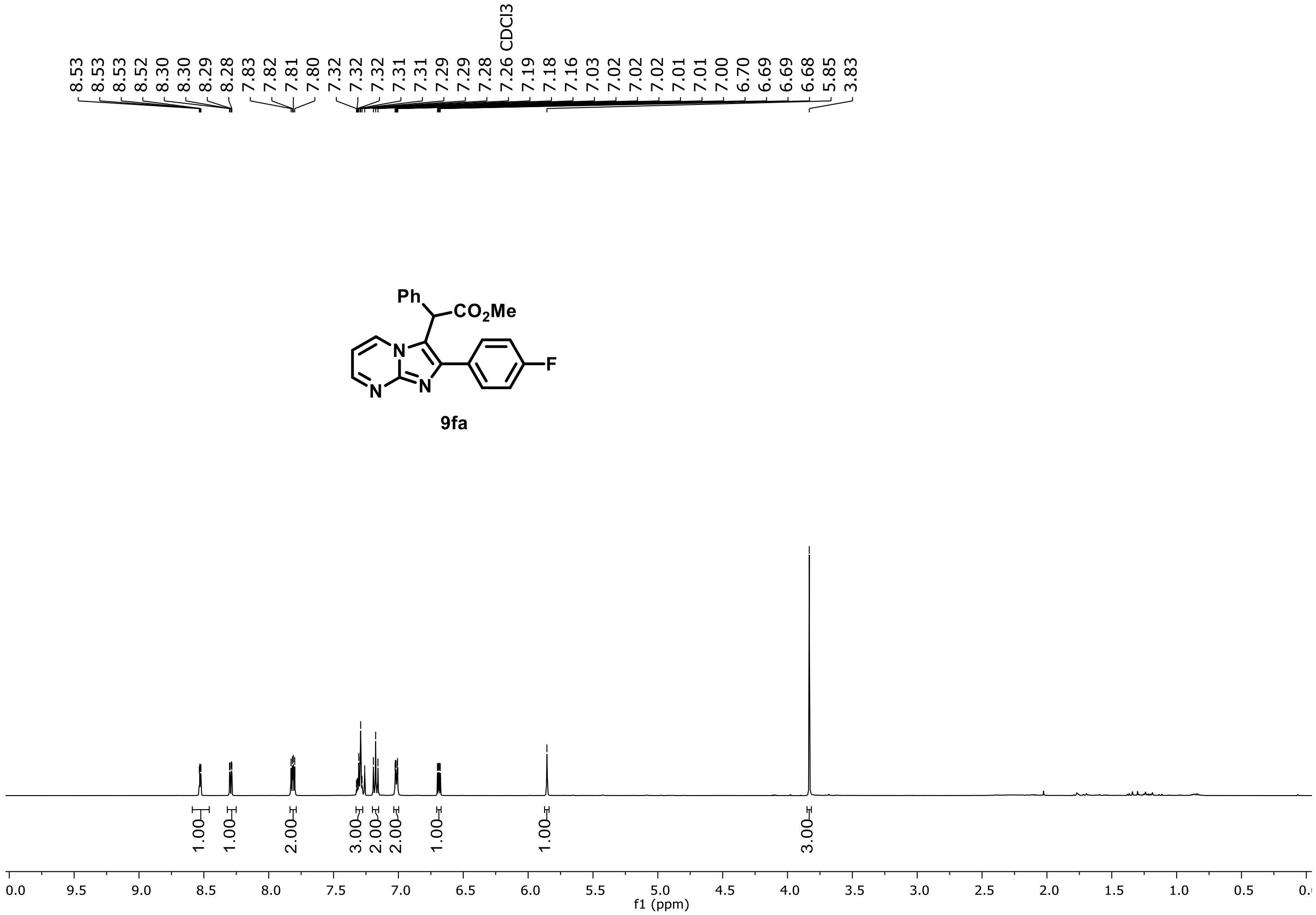

Molecule 9fa: $^{13}C\{^1H\}$ NMR (125 MHz, $CDCl_3$)

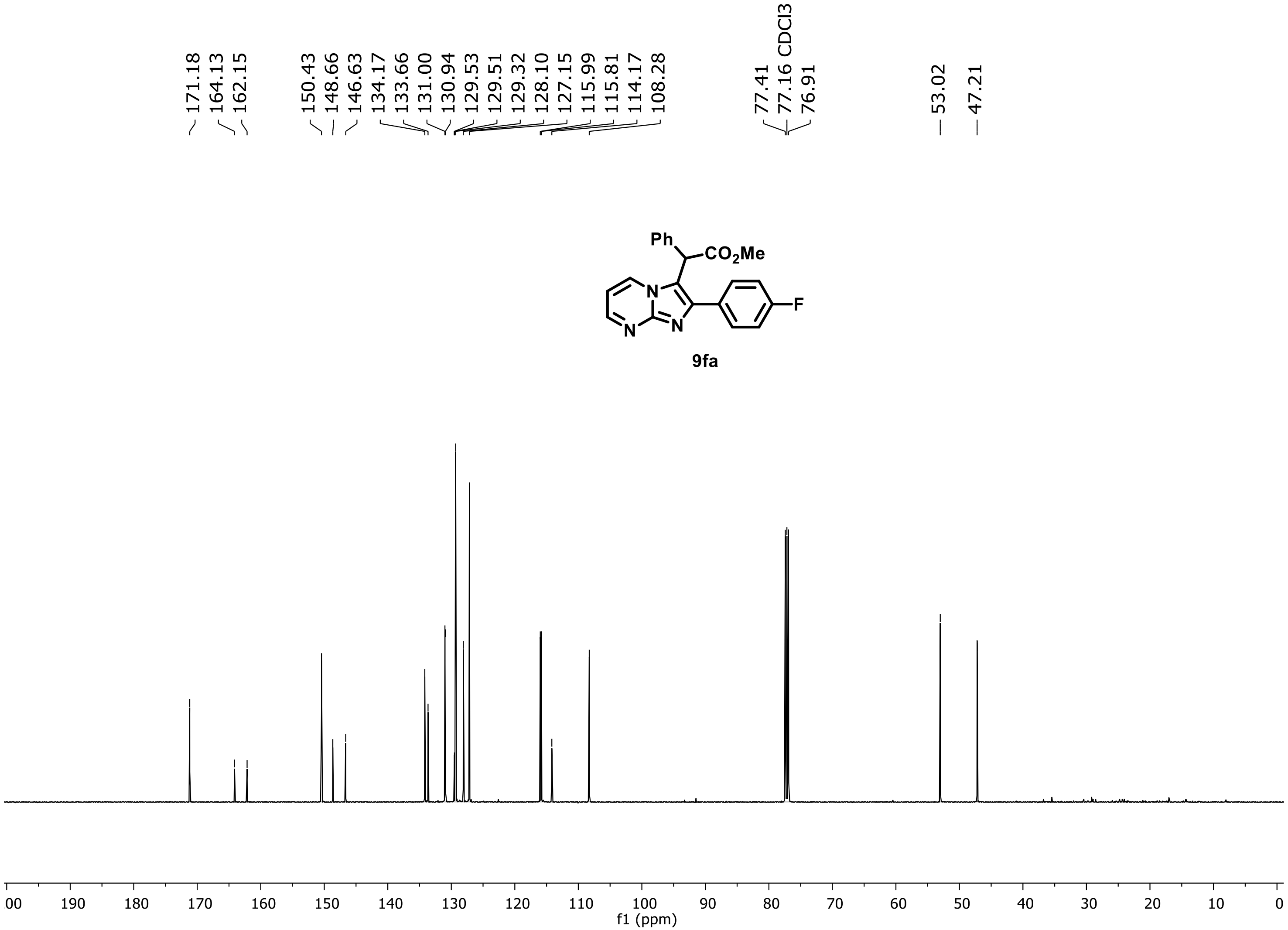

Molecule 9fa: $^{19}F\{^1H\}$ NMR (470 MHz, $CDCl_3$)

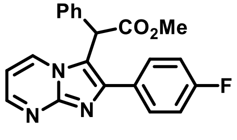

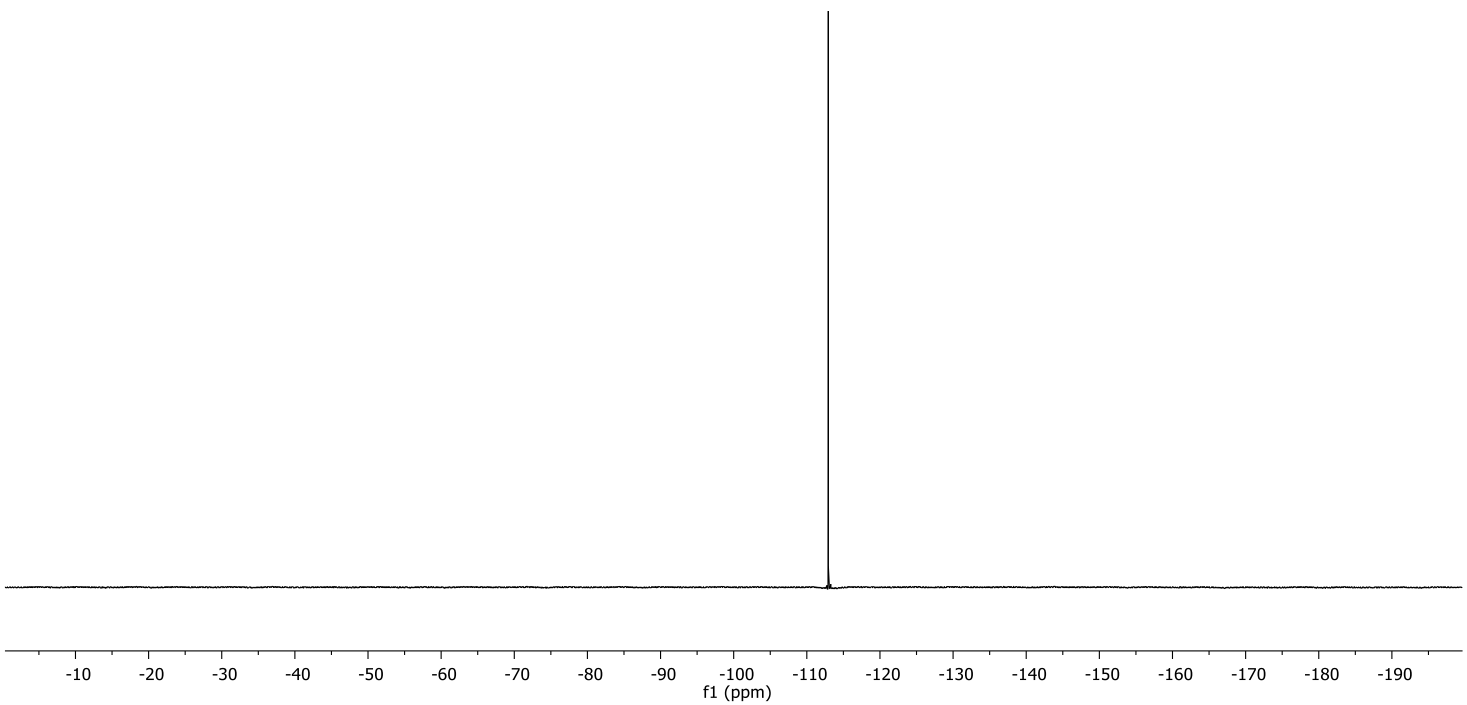

Molecule 9ga: ^{1}H NMR (250 MHz, $CDCl_3$)

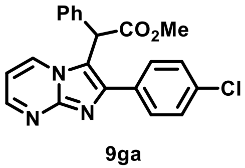

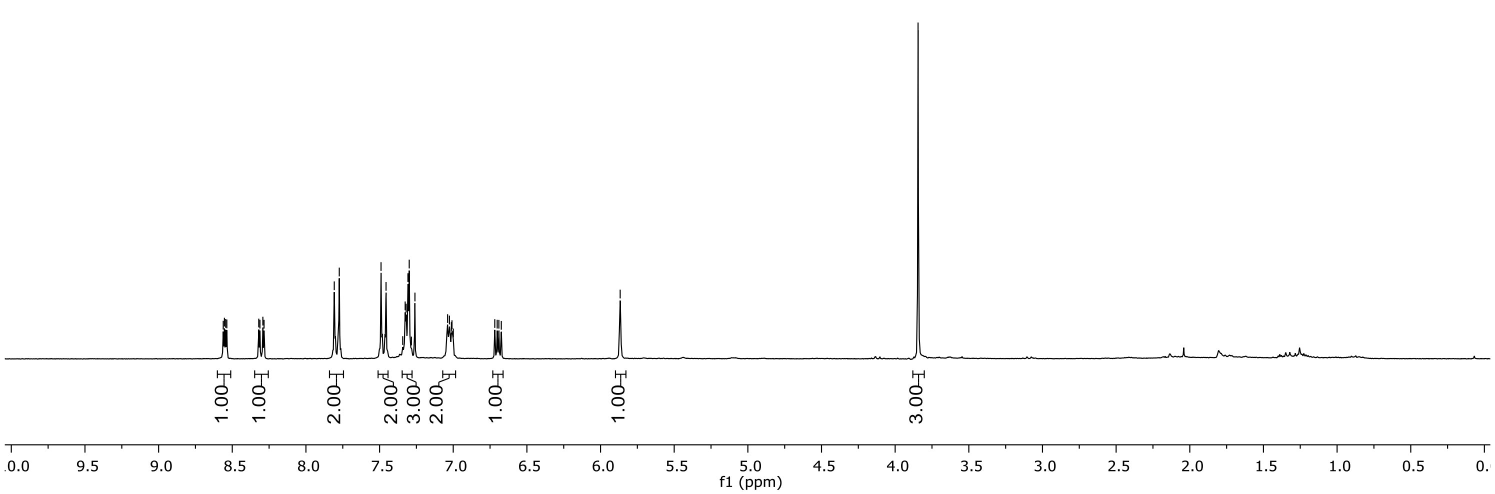

Molecule 9ga: $^{13}C\{^{1}H\}$ NMR (62.5 MHz, $CDCl_3$)

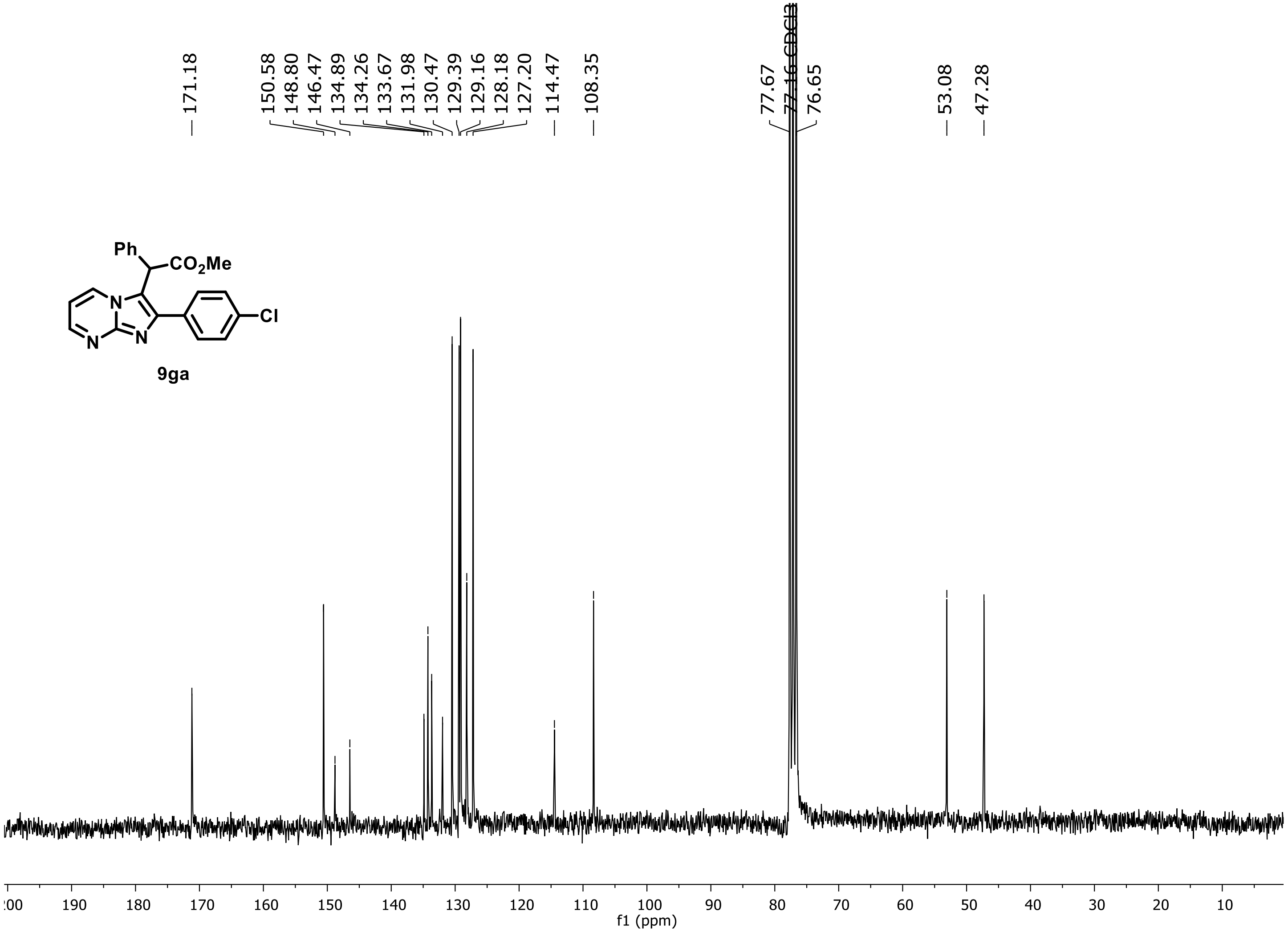

Molecule 9ha: ^{1}H NMR (500 MHz, $CDCl_3$)

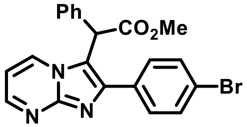

9ha

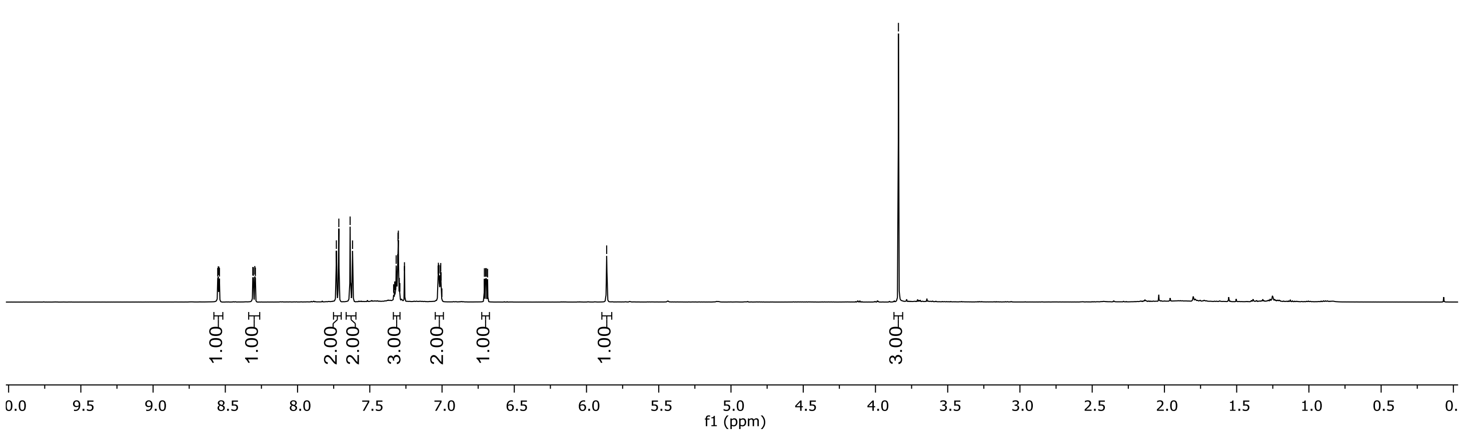

Molecule 9ha: $^{13}C\{^1H\}$ NMR (125 MHz, $CDCl_3$)

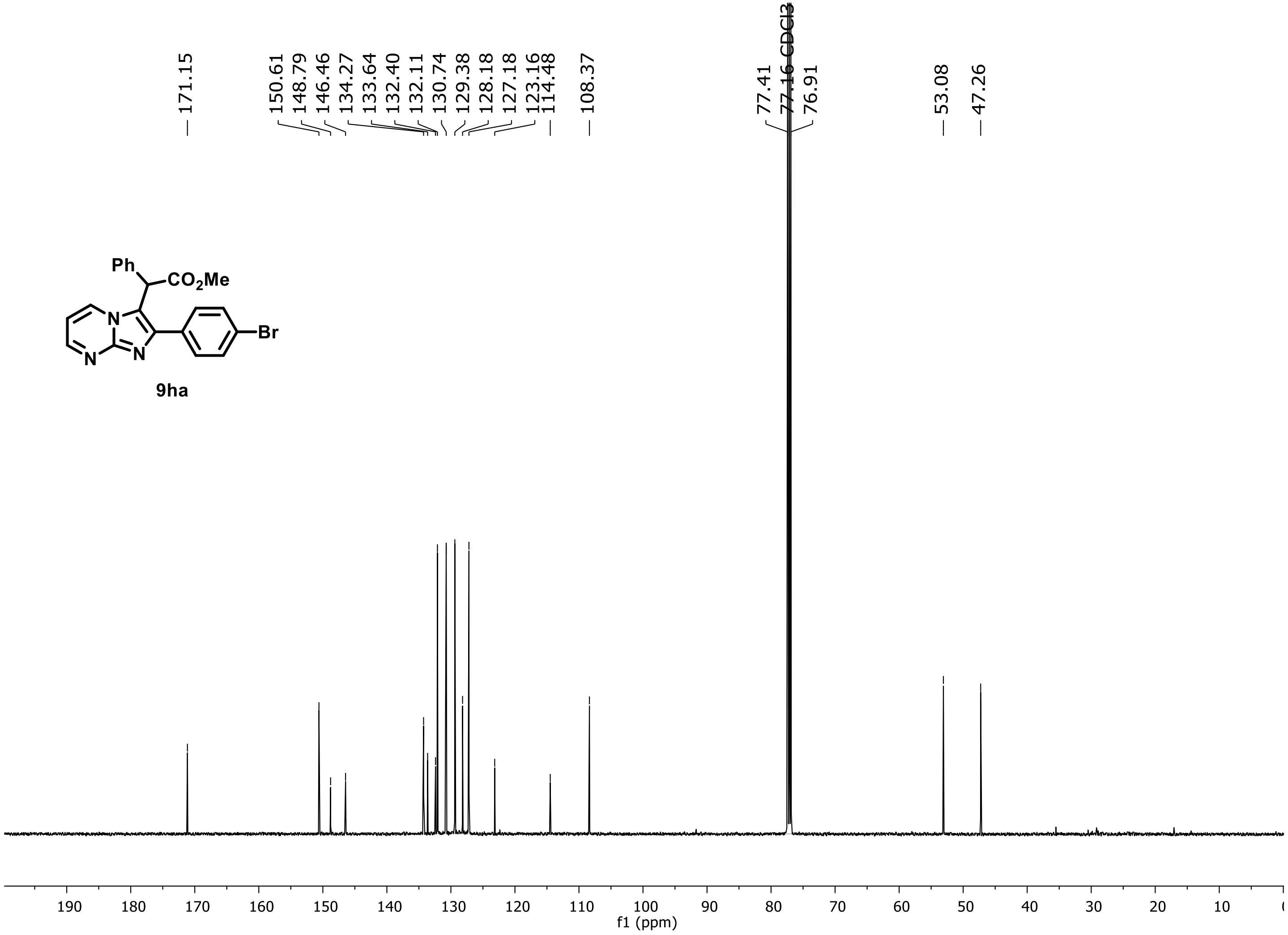

Molecule 9ia: ^{1}H NMR (600 MHz, $CDCl_3$)

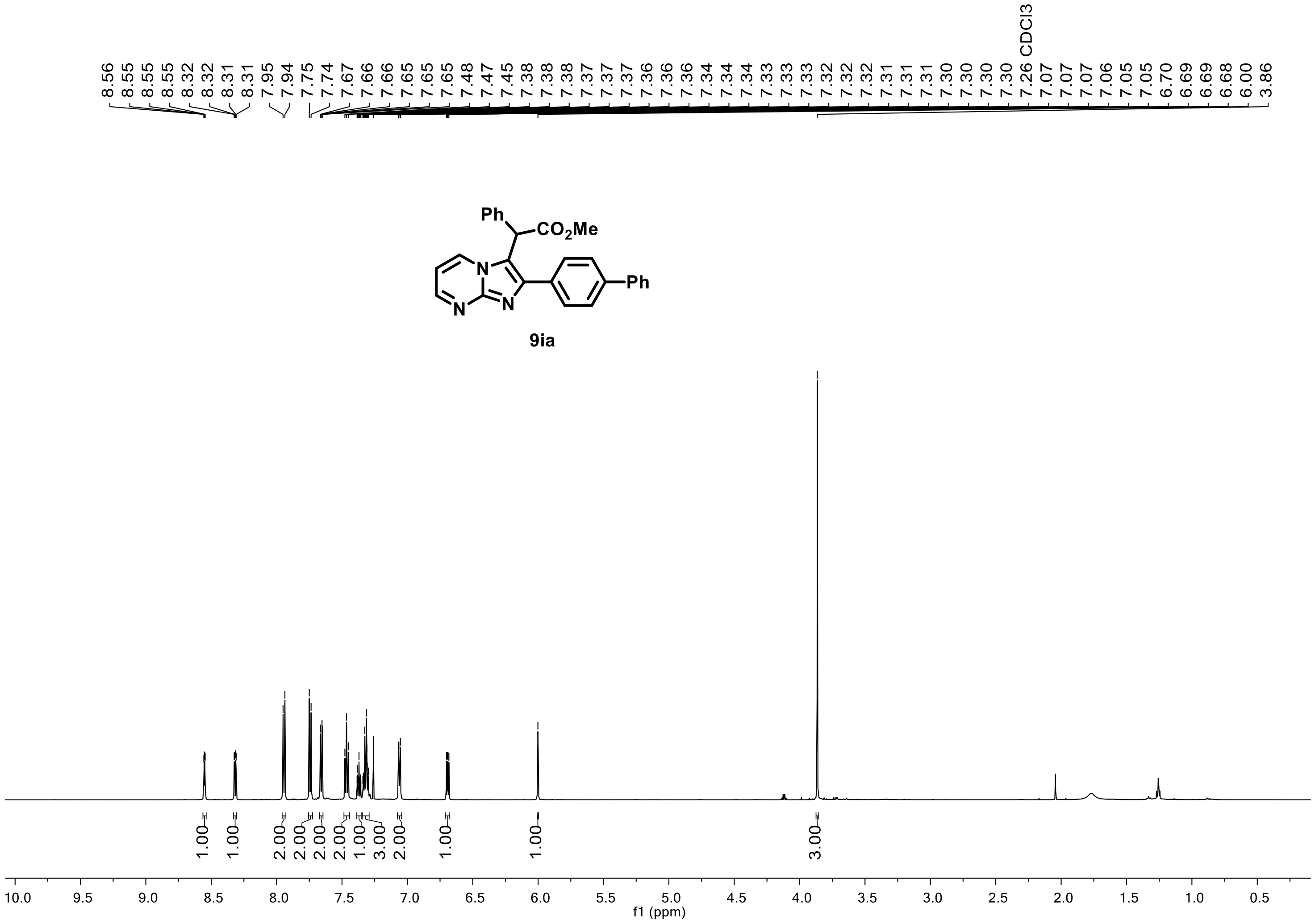

Molecule 9ia: $^{13}C\{^1H\}$ NMR (150 MHz, $CDCl_3$)

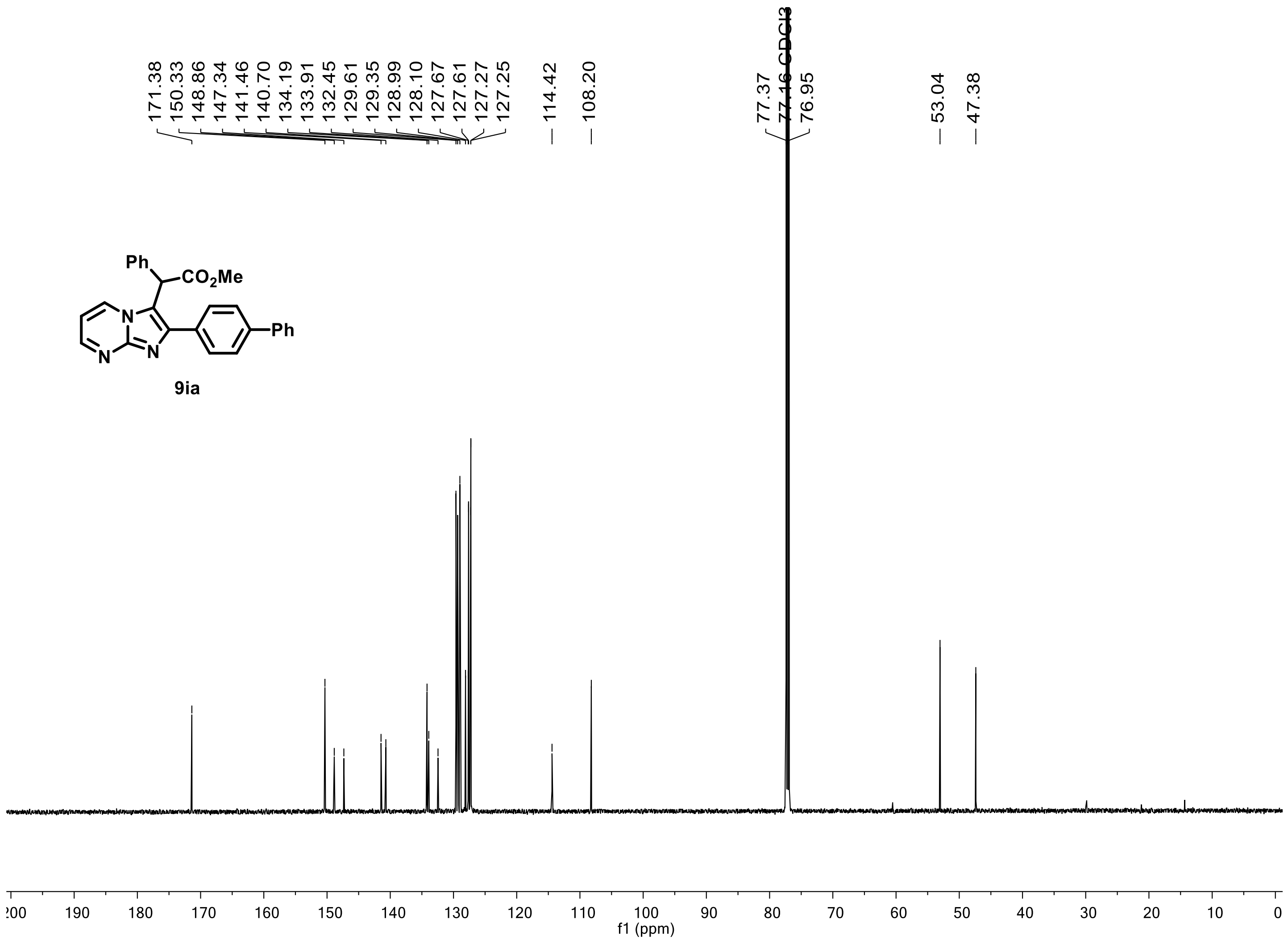

Molecule 9ja: ^{1}H NMR (500 MHz, $CDCl_3$)

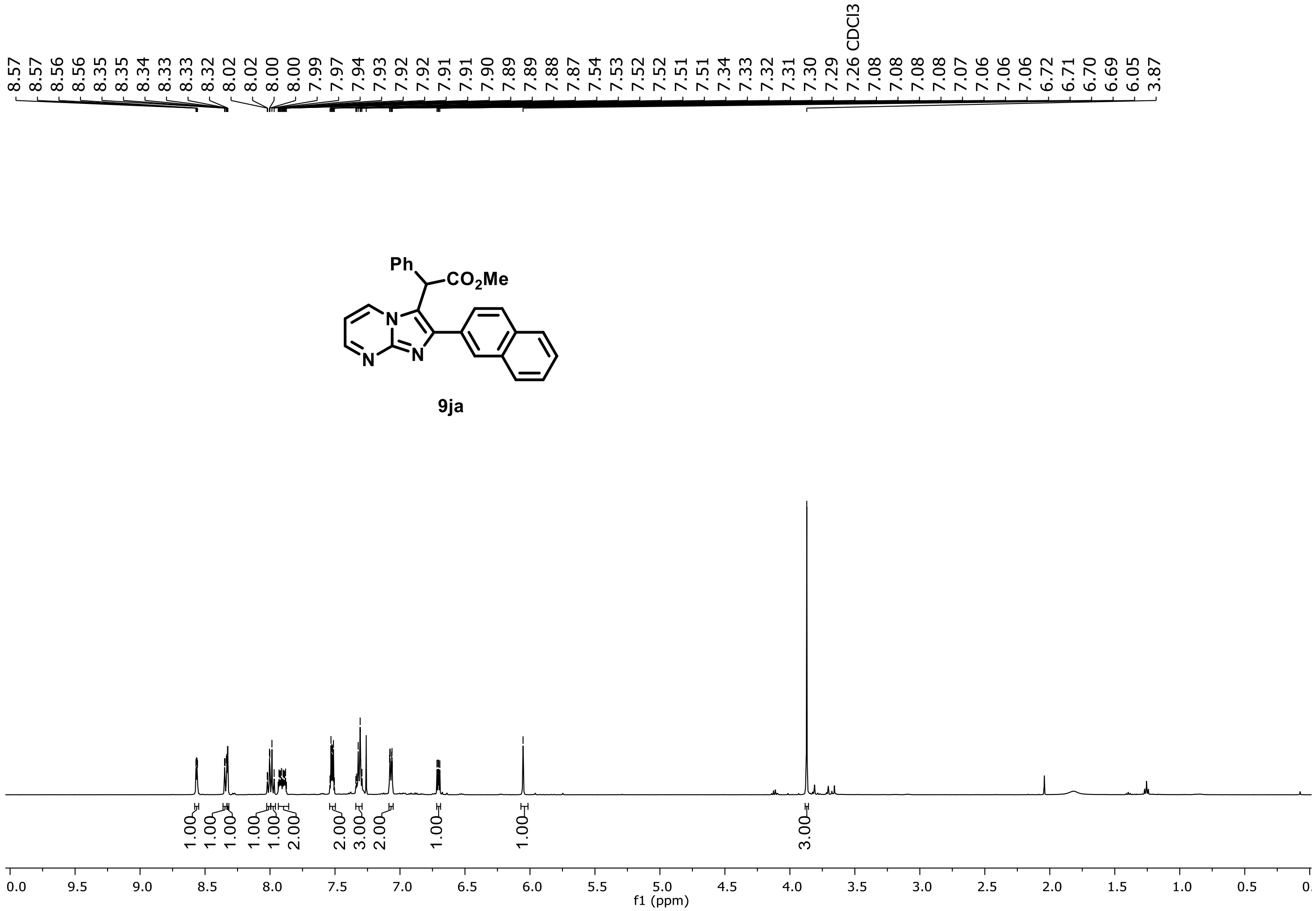

Molecule 9ja: $^{13}C\{^1H\}$ NMR (125 MHz, CDCl$_3$)

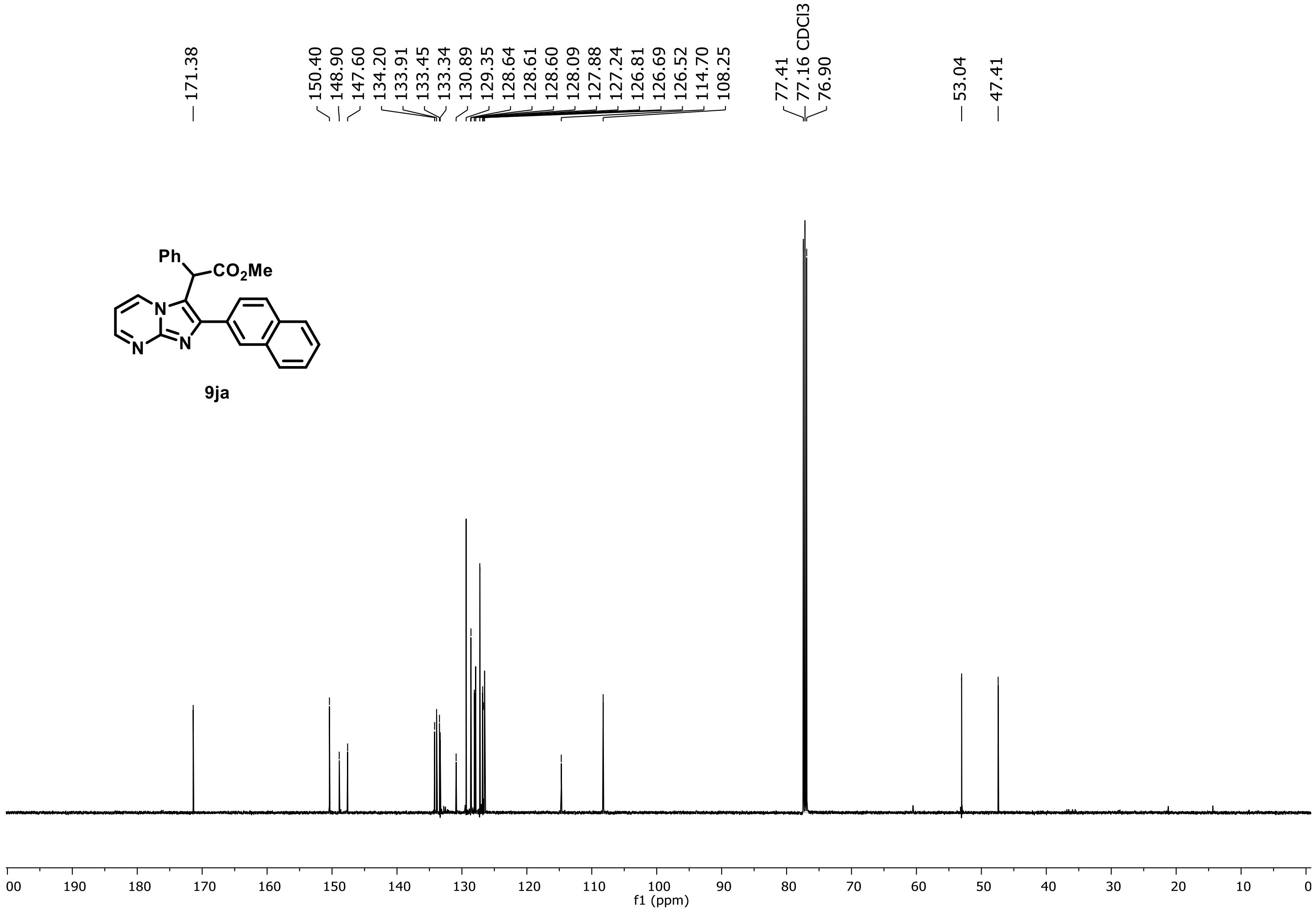

Molecule 9ka: ^{1}H NMR (500 MHz, $CDCl_3$)

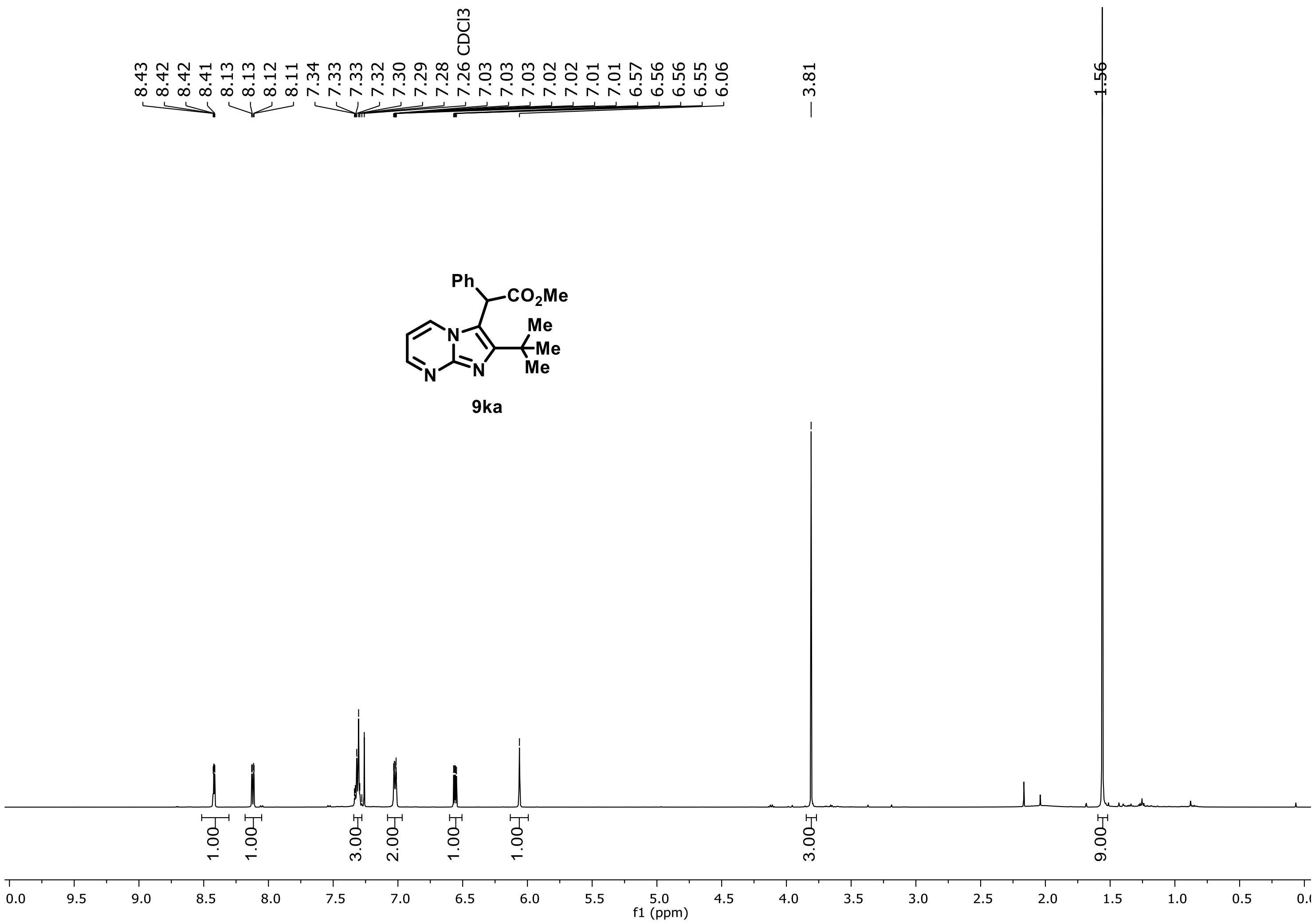

Molecule 9ka: $^{13}C\{^{1}H\}$ NMR (125 MHz, $CDCl_3$)

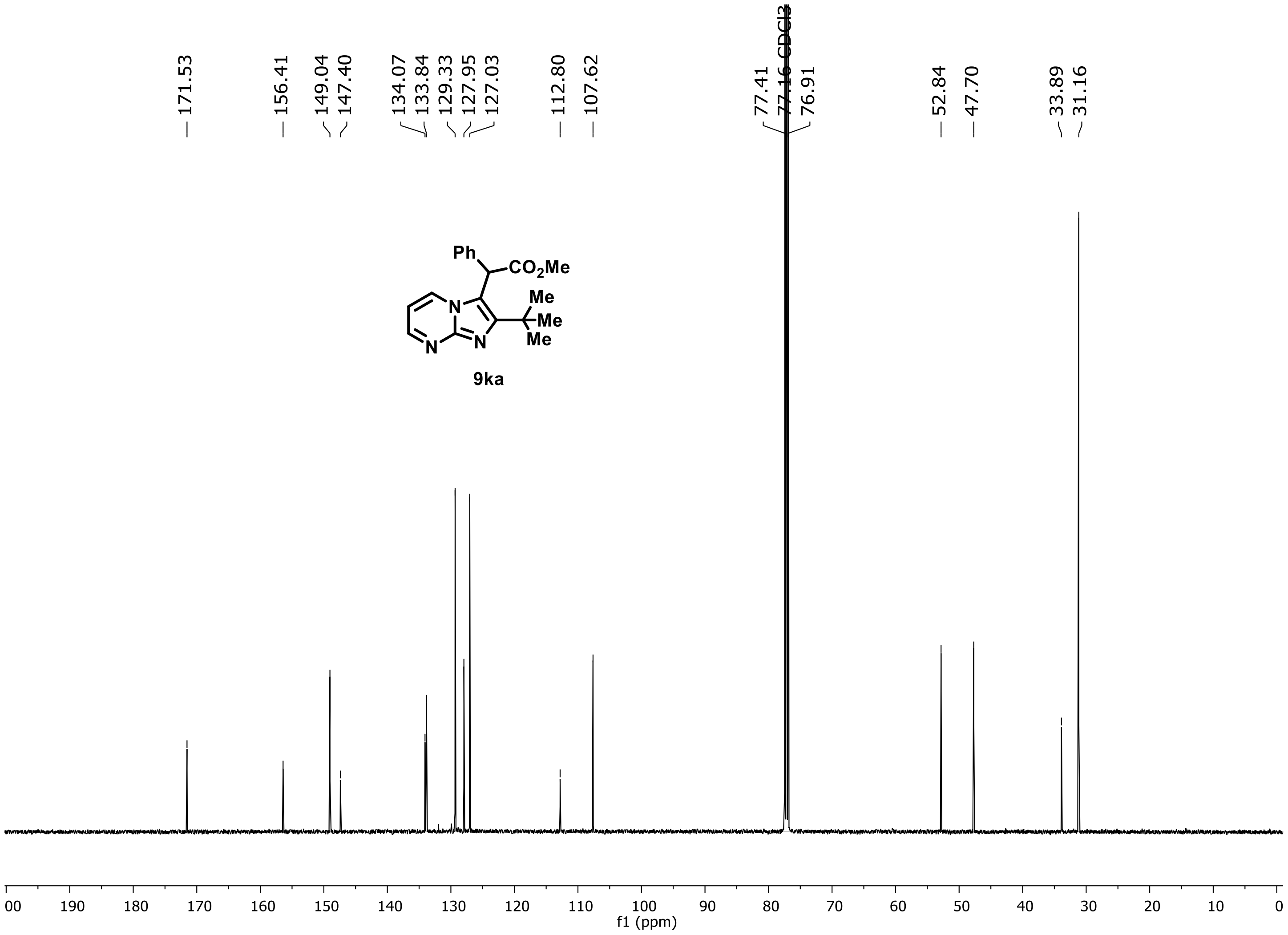

Molecule 9ab: ^{1}H NMR (600 MHz, $CDCl_3$)

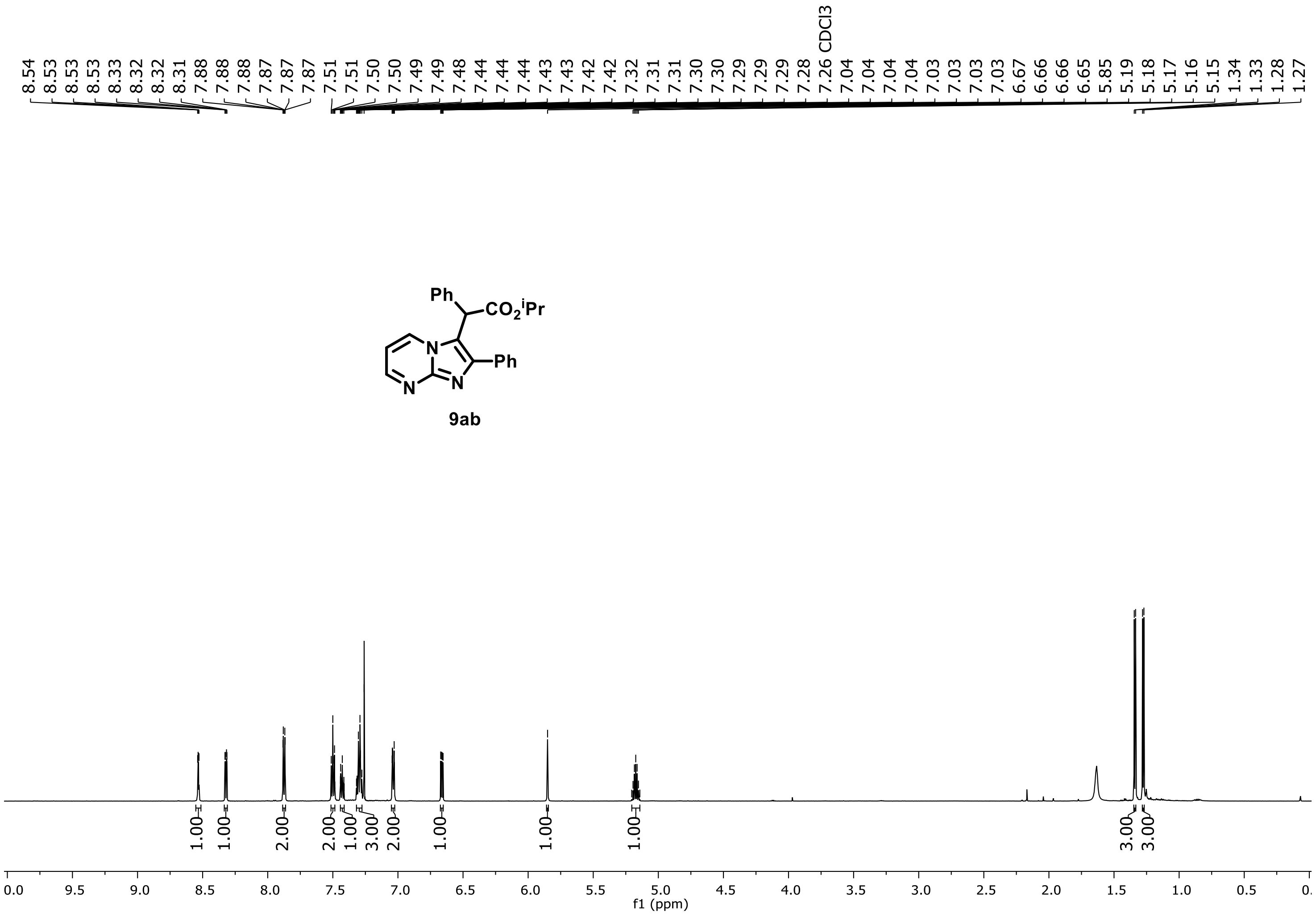

Molecule 9ab: $^{13}C\{^{1}H\}$ NMR (151 MHz, $CDCl_3$)

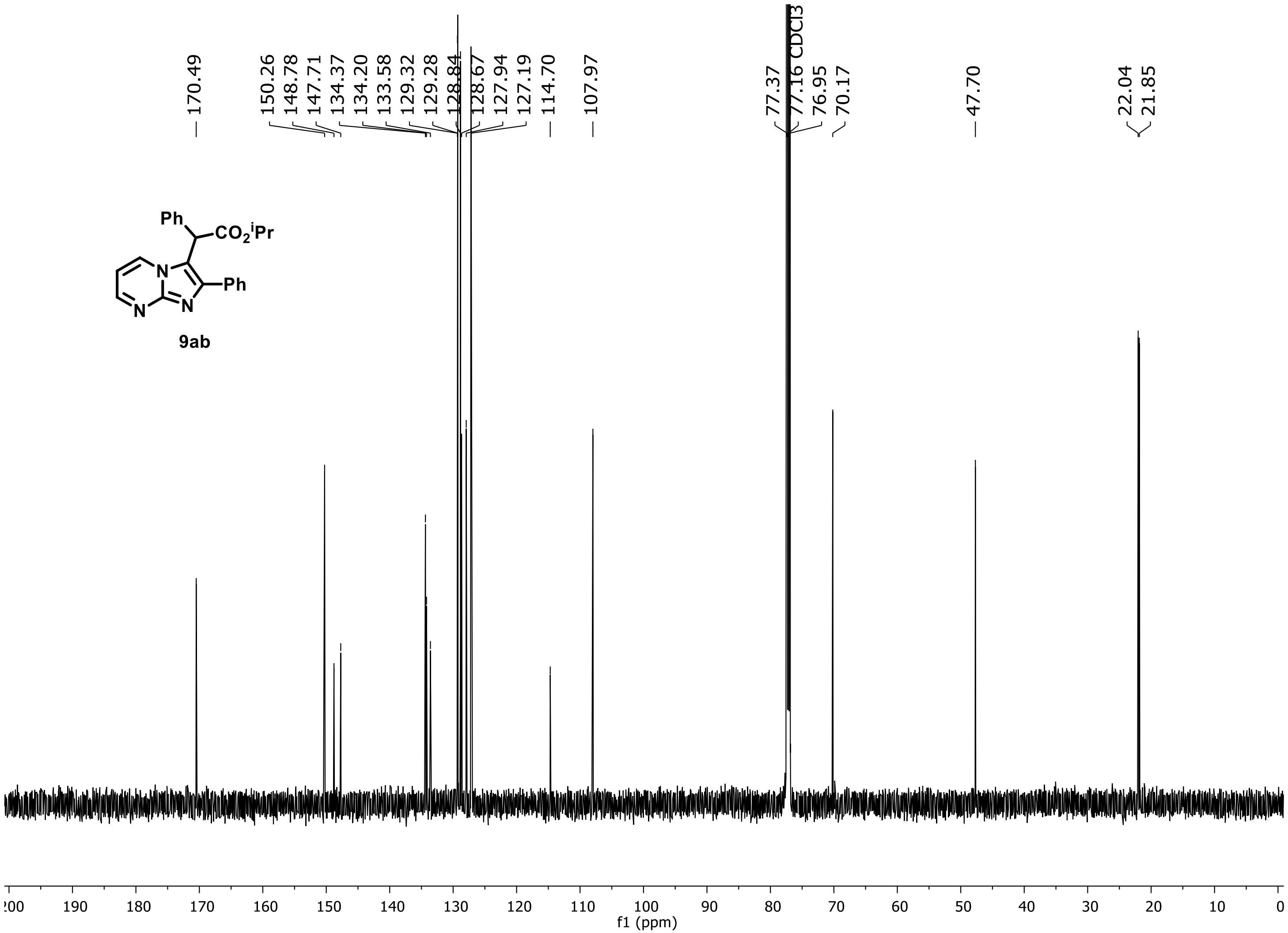

Molecule 9ac: ^{1}H NMR (500 MHz, $CDCl_3$)

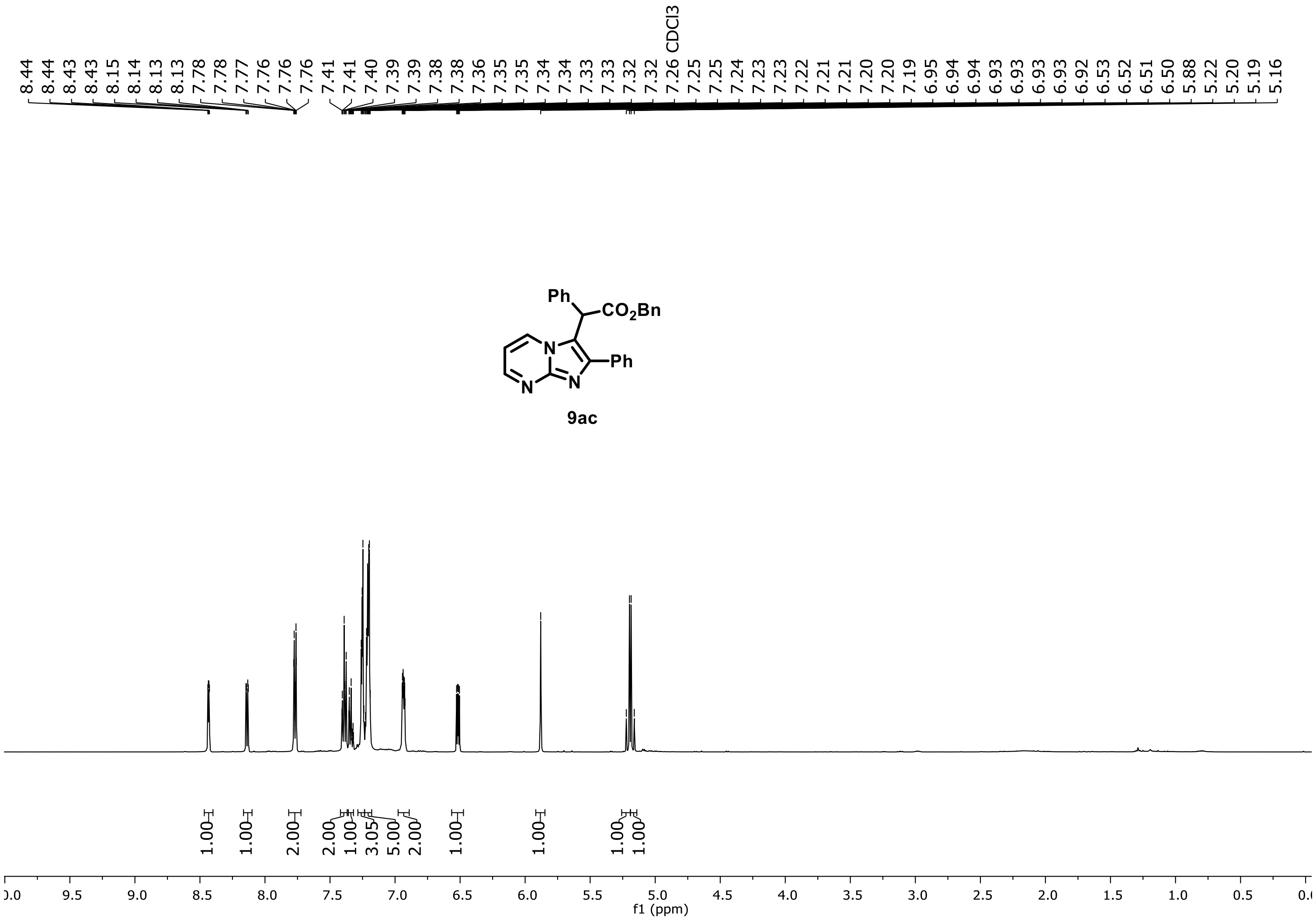

Molecule 9ac: $^{13}C\{^{1}H\}$ NMR (125 MHz, $CDCl_3$)

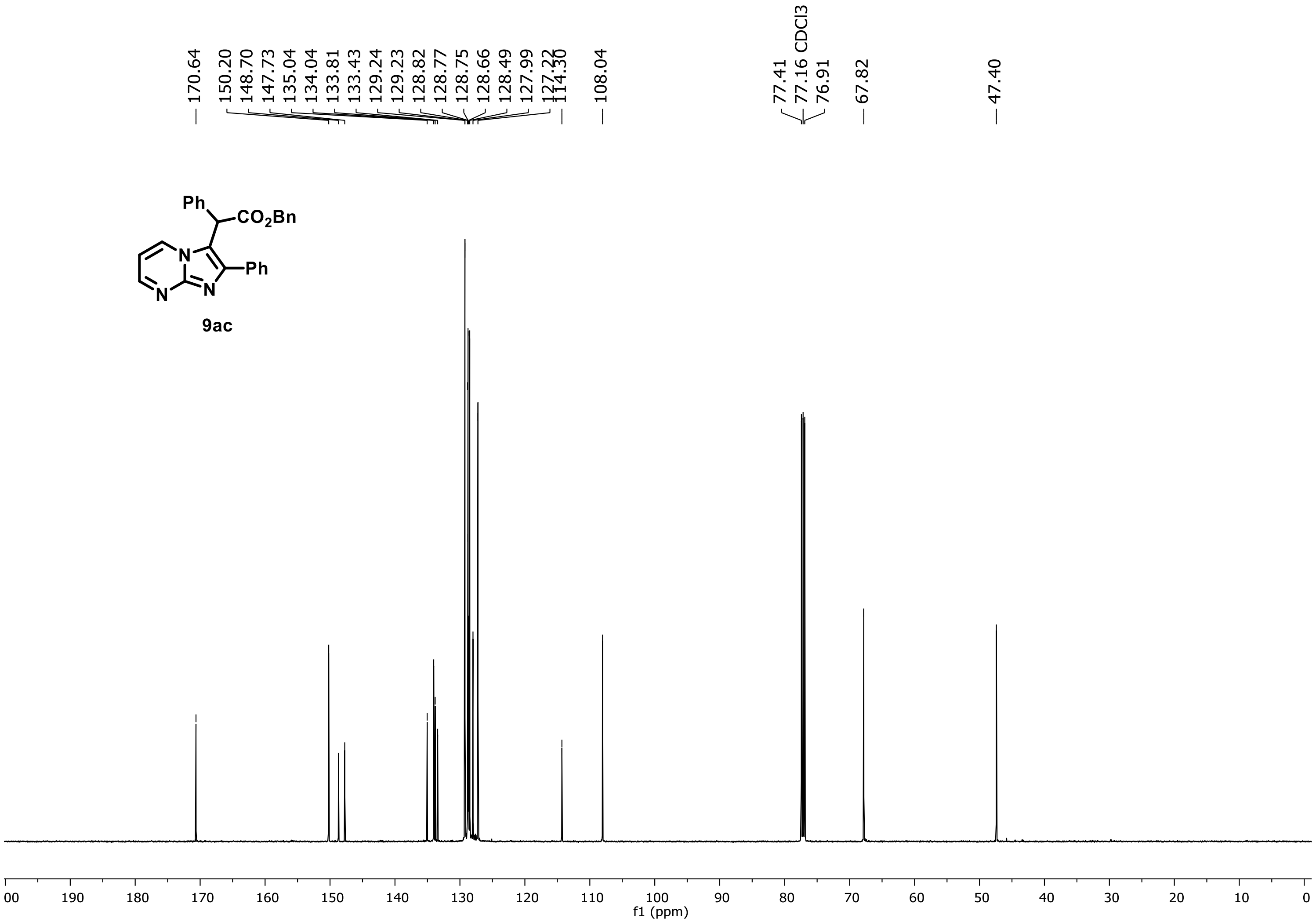

Molecule 9ad: ^{1}H NMR (250 MHz, $CDCl_3$)

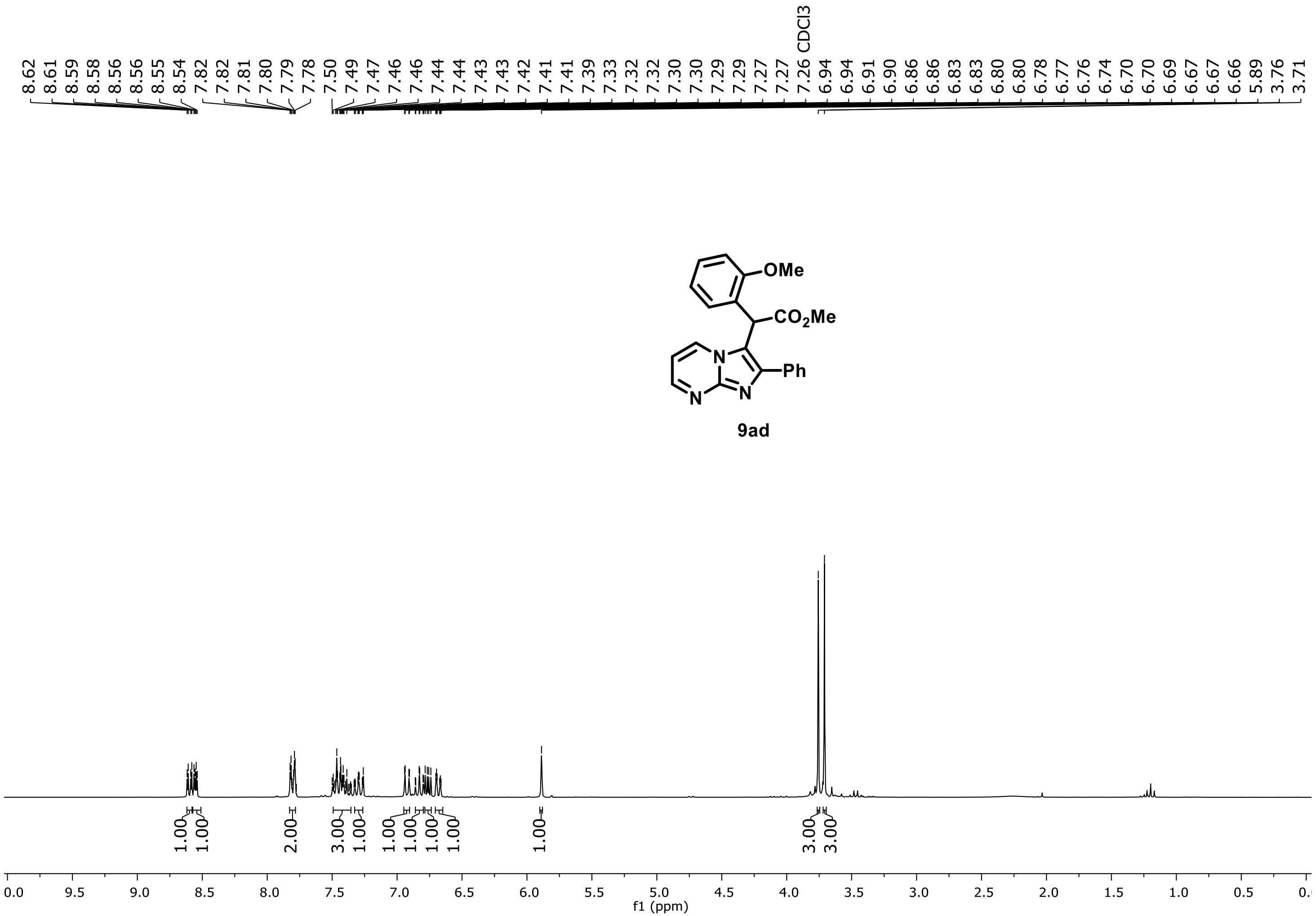

Molecule 9ad: $^{13}C\{^{1}H\}$ NMR (62.5 MHz, $CDCl_3$)

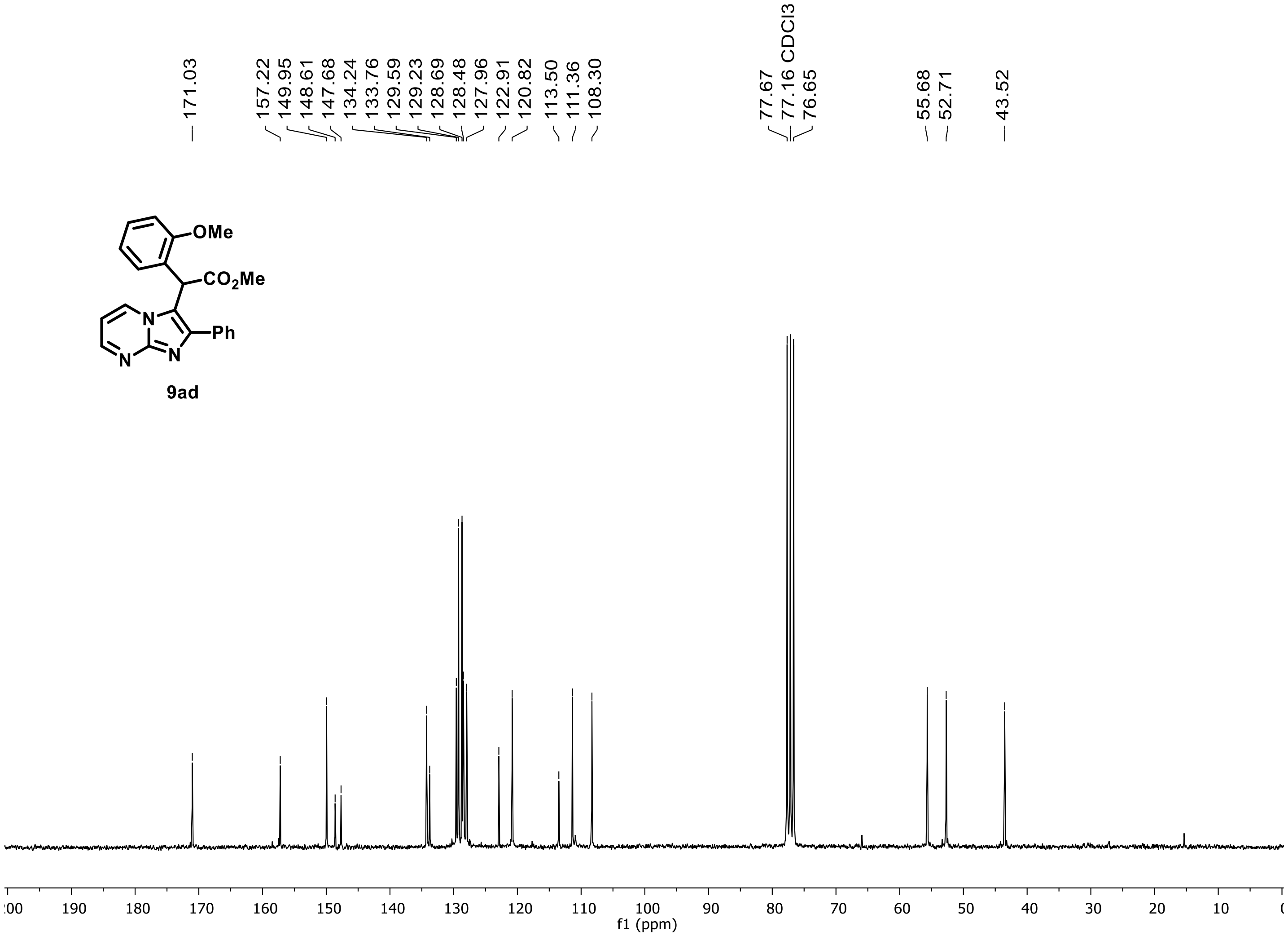

Molecule 9ae: ¹H NMR (300 MHz, $CDCl_3$)

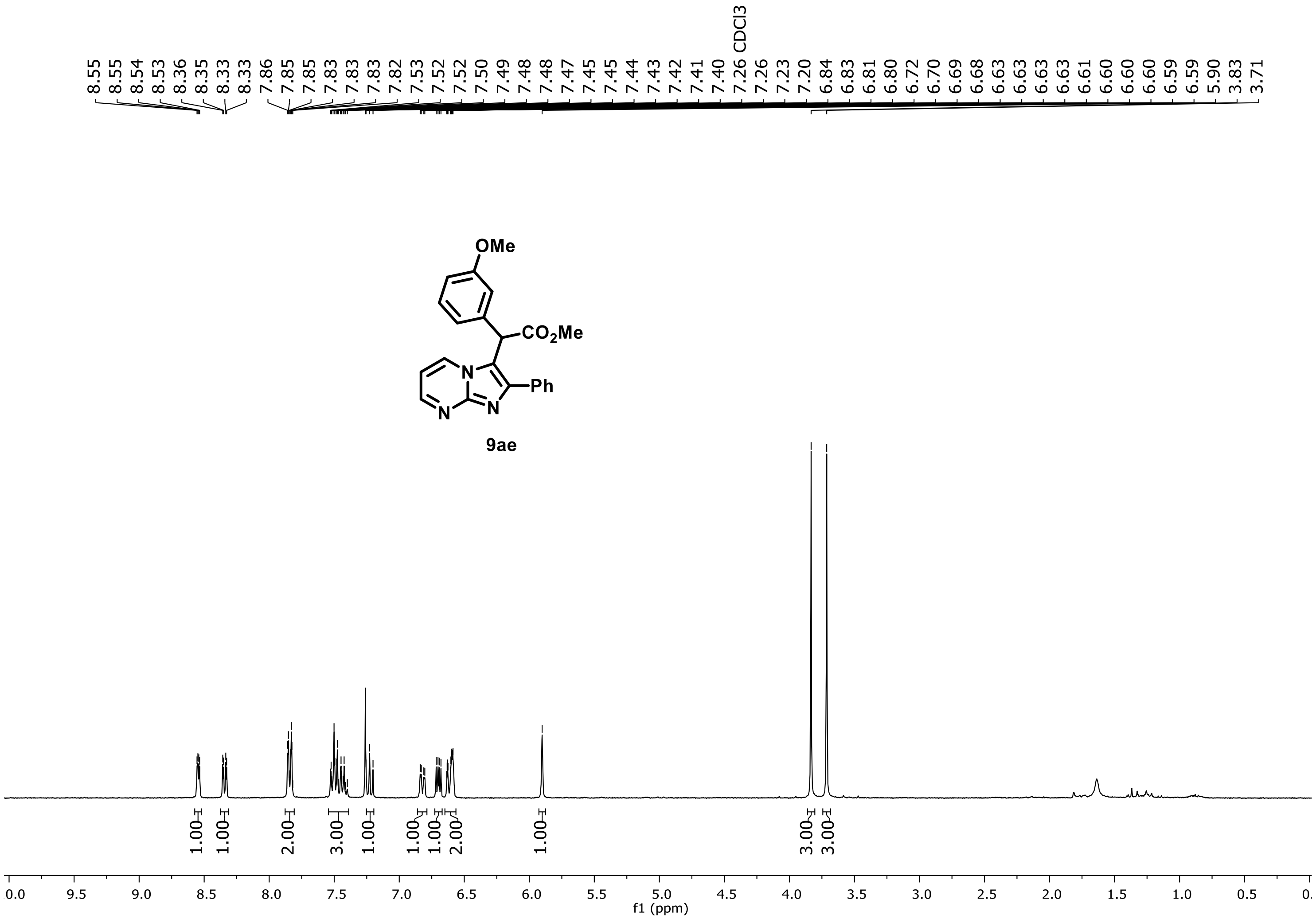

Molecule 9ae: $^{13}C\{^{1}H\}$ NMR (75 MHz, $CDCl_3$)

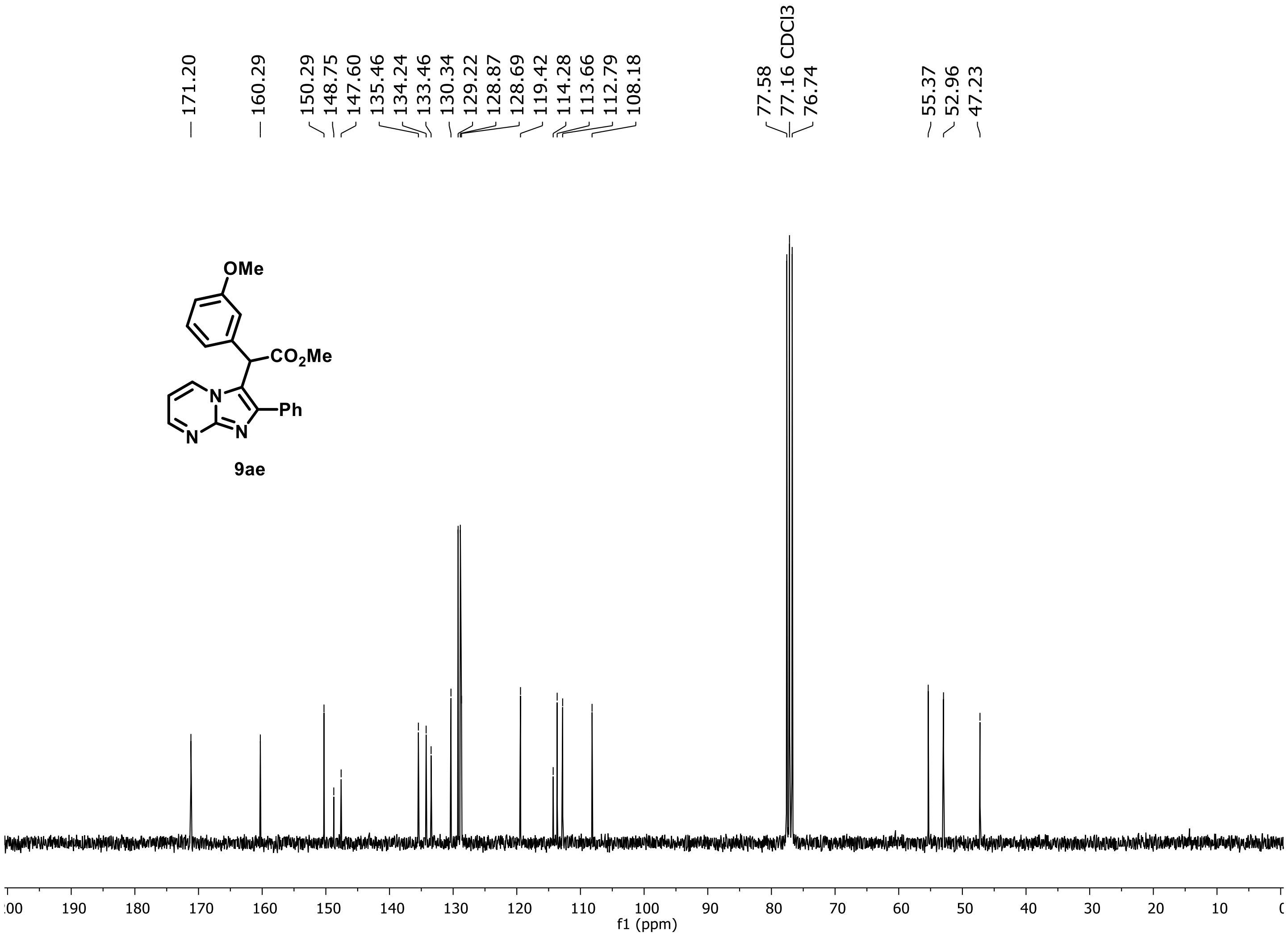

Molecule 9af: ^{1}H NMR (300 MHz, $CDCl_3$)

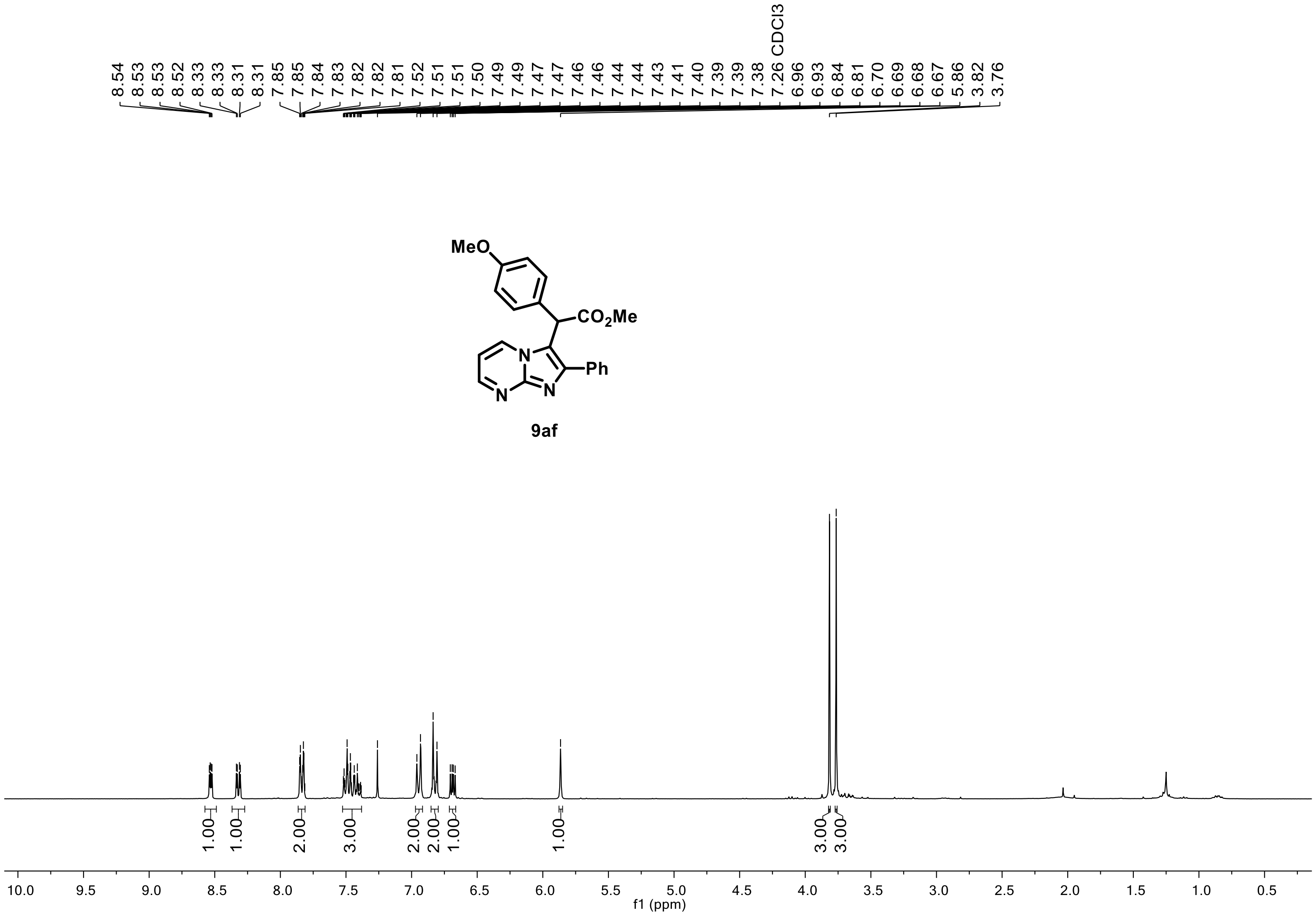

Molecule 9af: $^{13}C\{^1H\}$ NMR (75 MHz, $CDCl_3$)

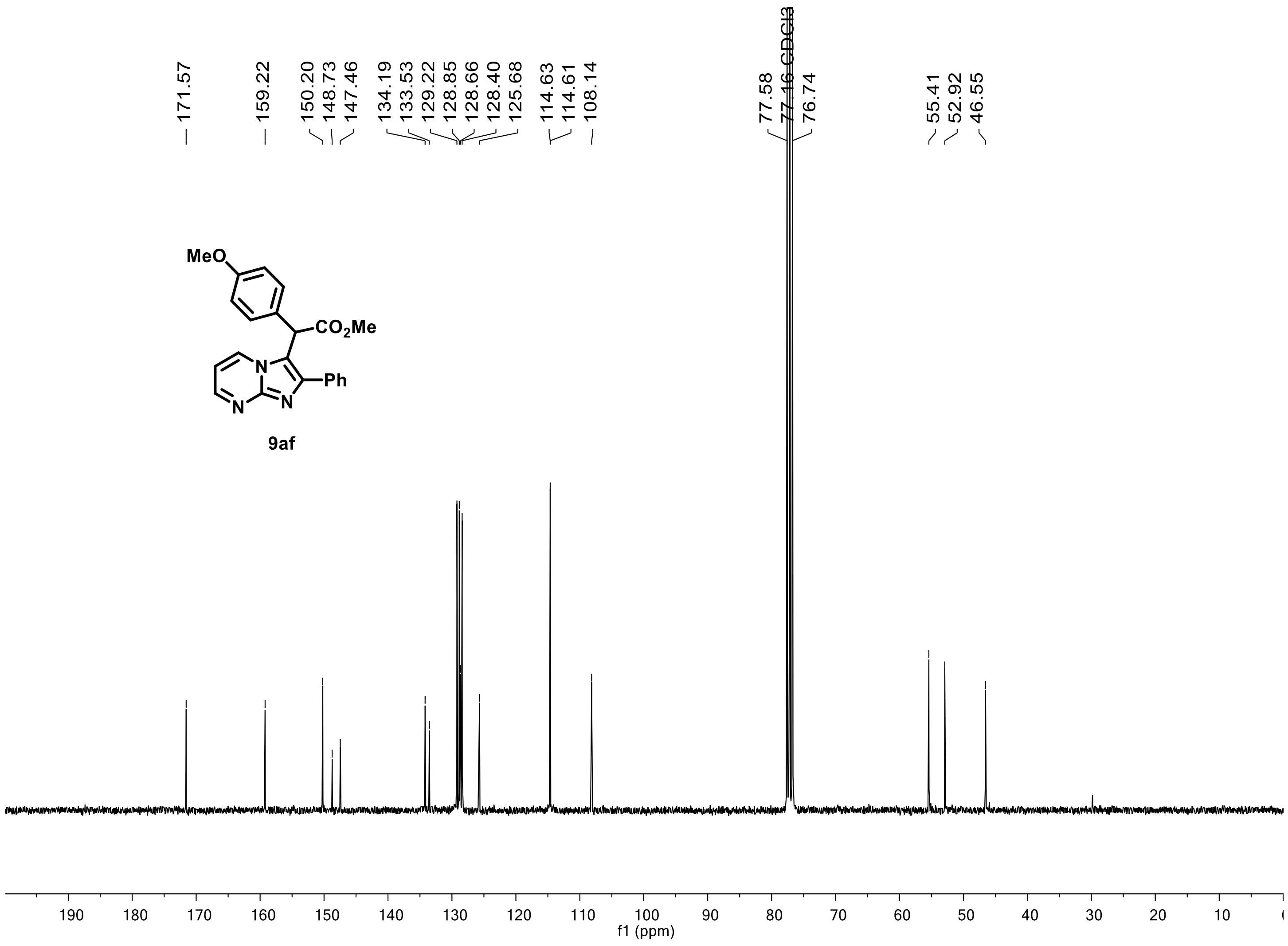

Molecule 9ag: ^{1}H NMR (250 MHz, $CDCl_3$)

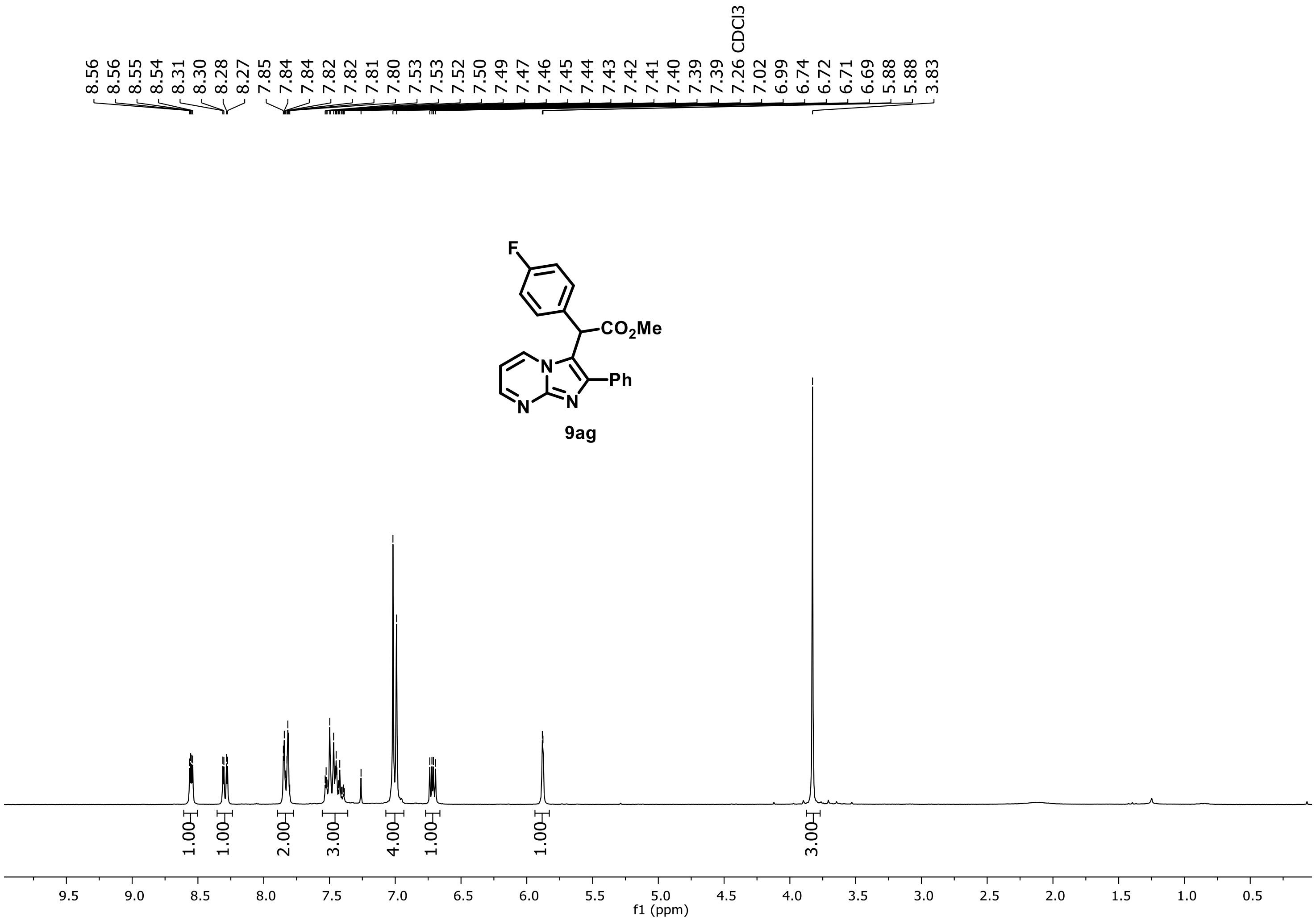

Molecule 9ag: $^{13}C\{^1H\}$ NMR (62.5 MHz, $CDCl_3$)

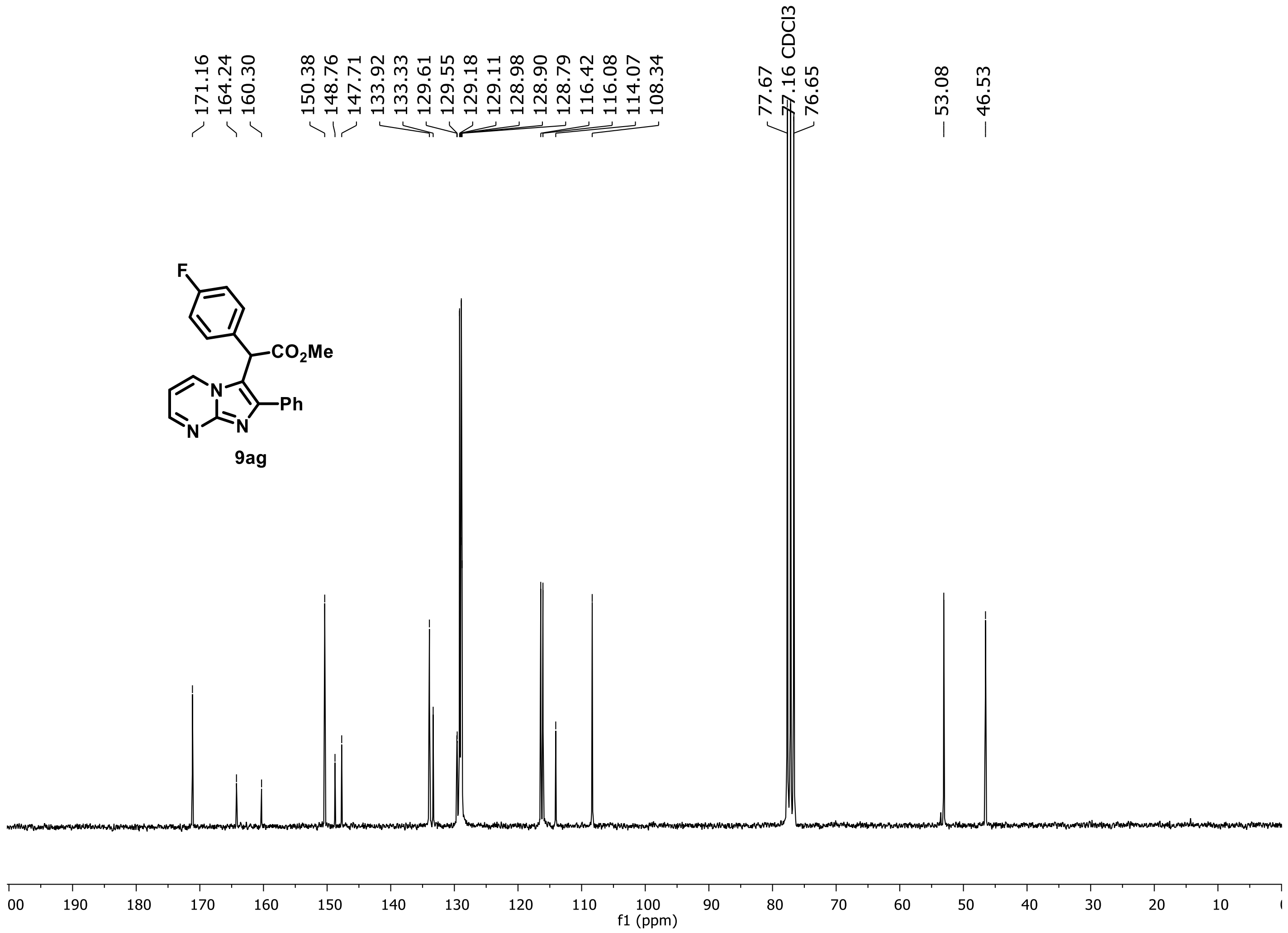

Molecule 9ag: $^{19}F\{^1H\}$ NMR (235 MHz, $CDCl_3$)

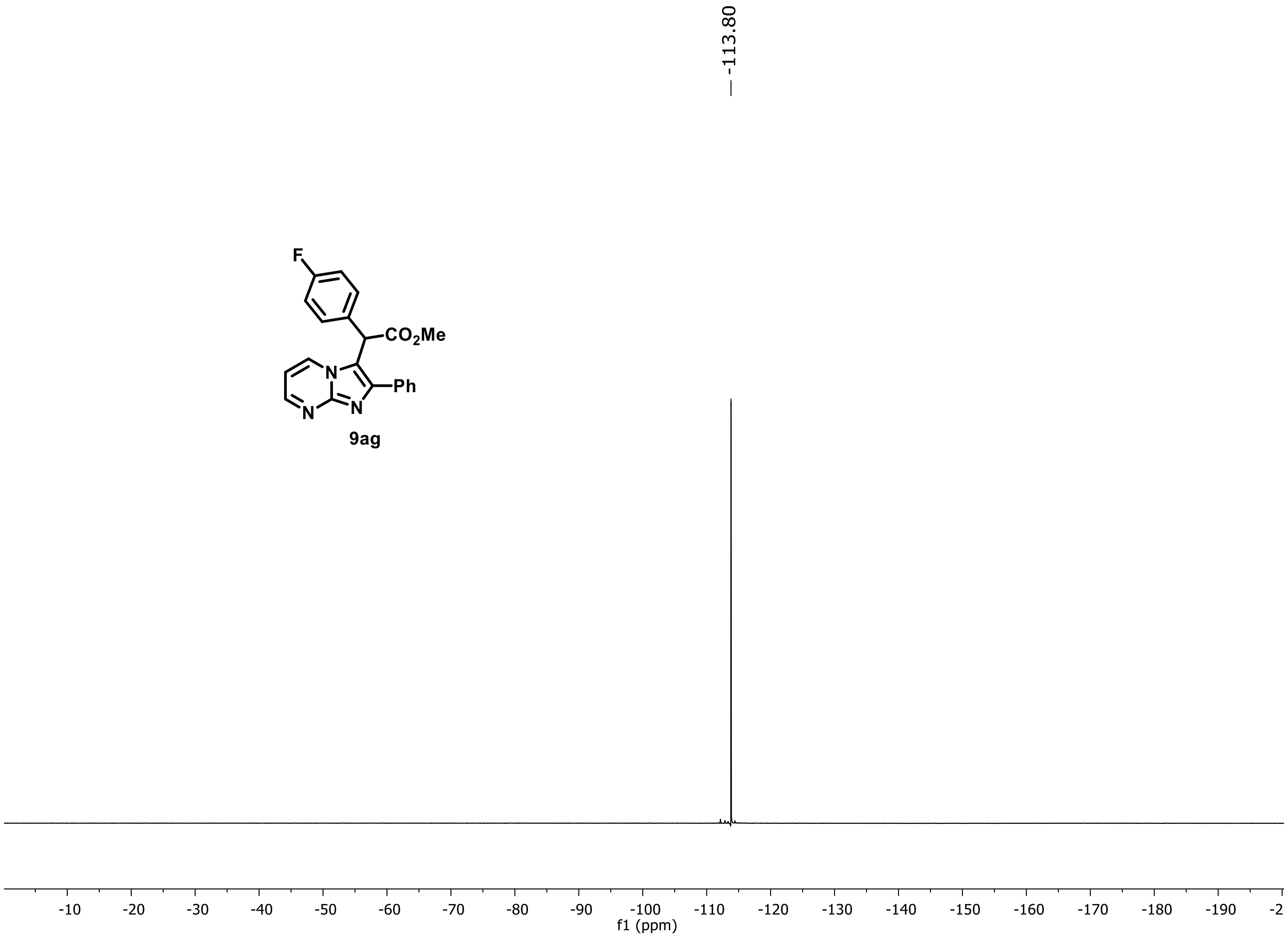

Molecule 9ah: ^{1}H NMR (250 MHz, $CDCl_3$)

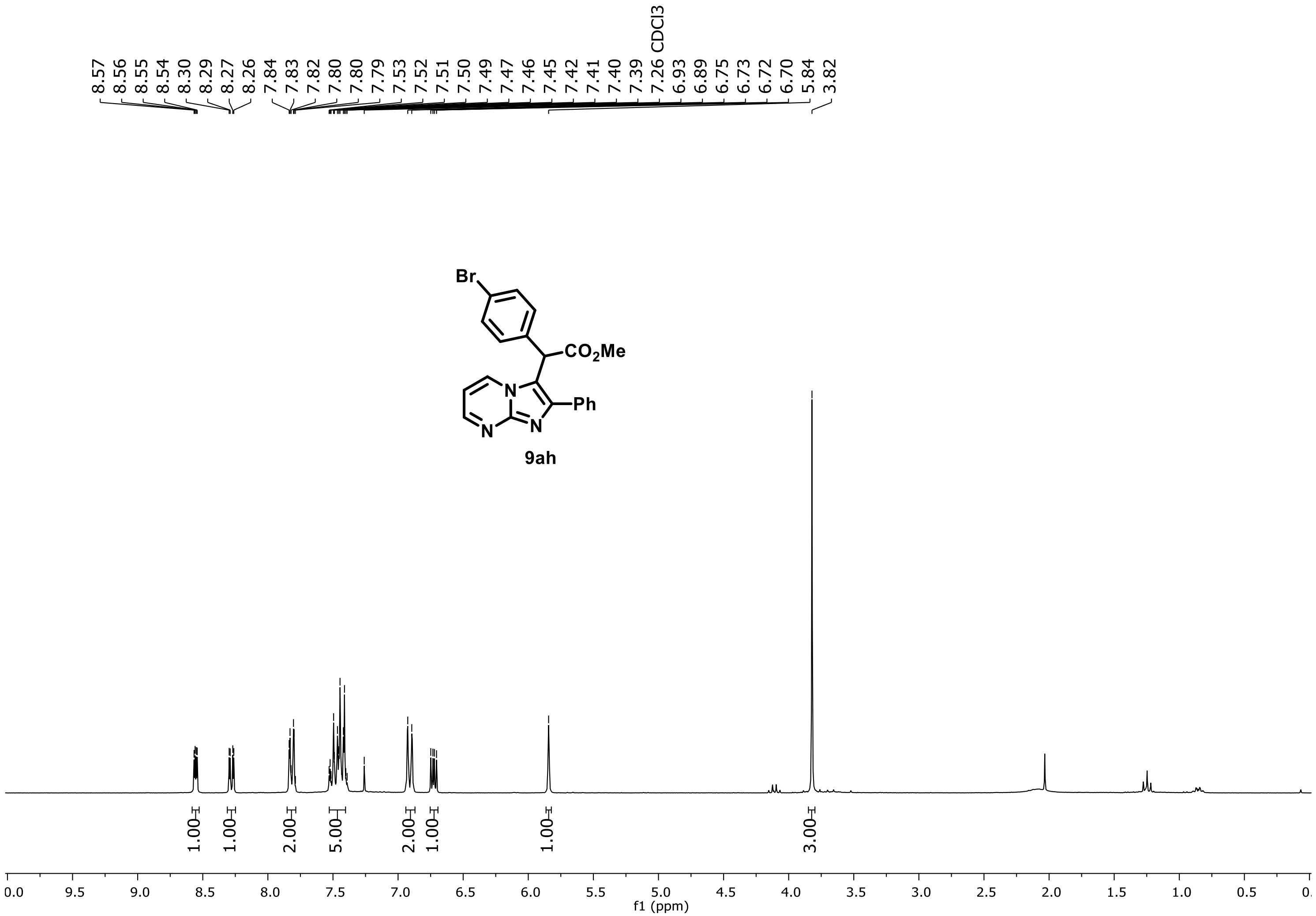

9ah

Molecule 9ah: $^{13}C\{^{1}H\}$ NMR (63 MHz, $CDCl_3$)

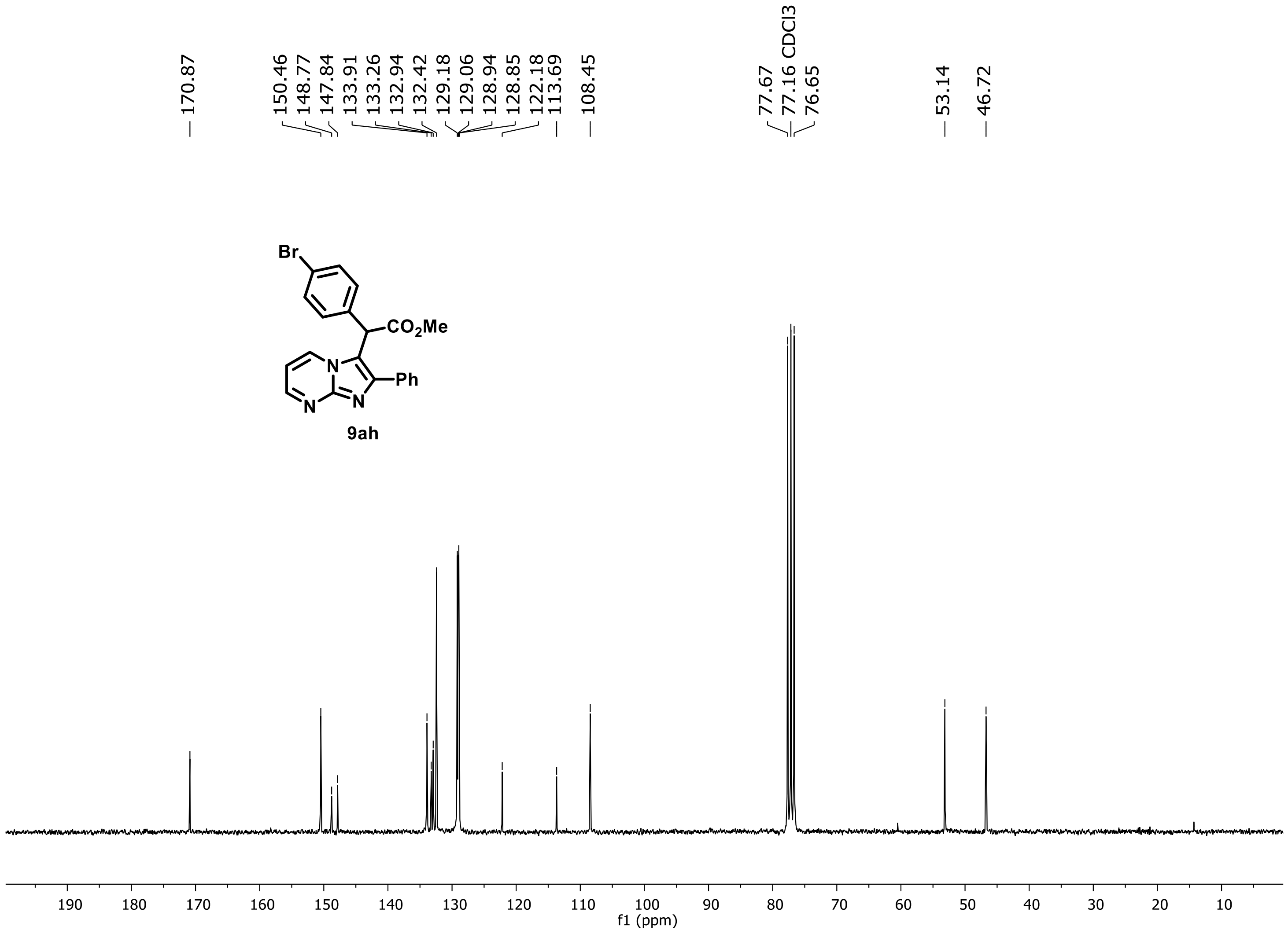

Molecule 9ai: ^{1}H NMR (500 MHz, $CDCl_3$)

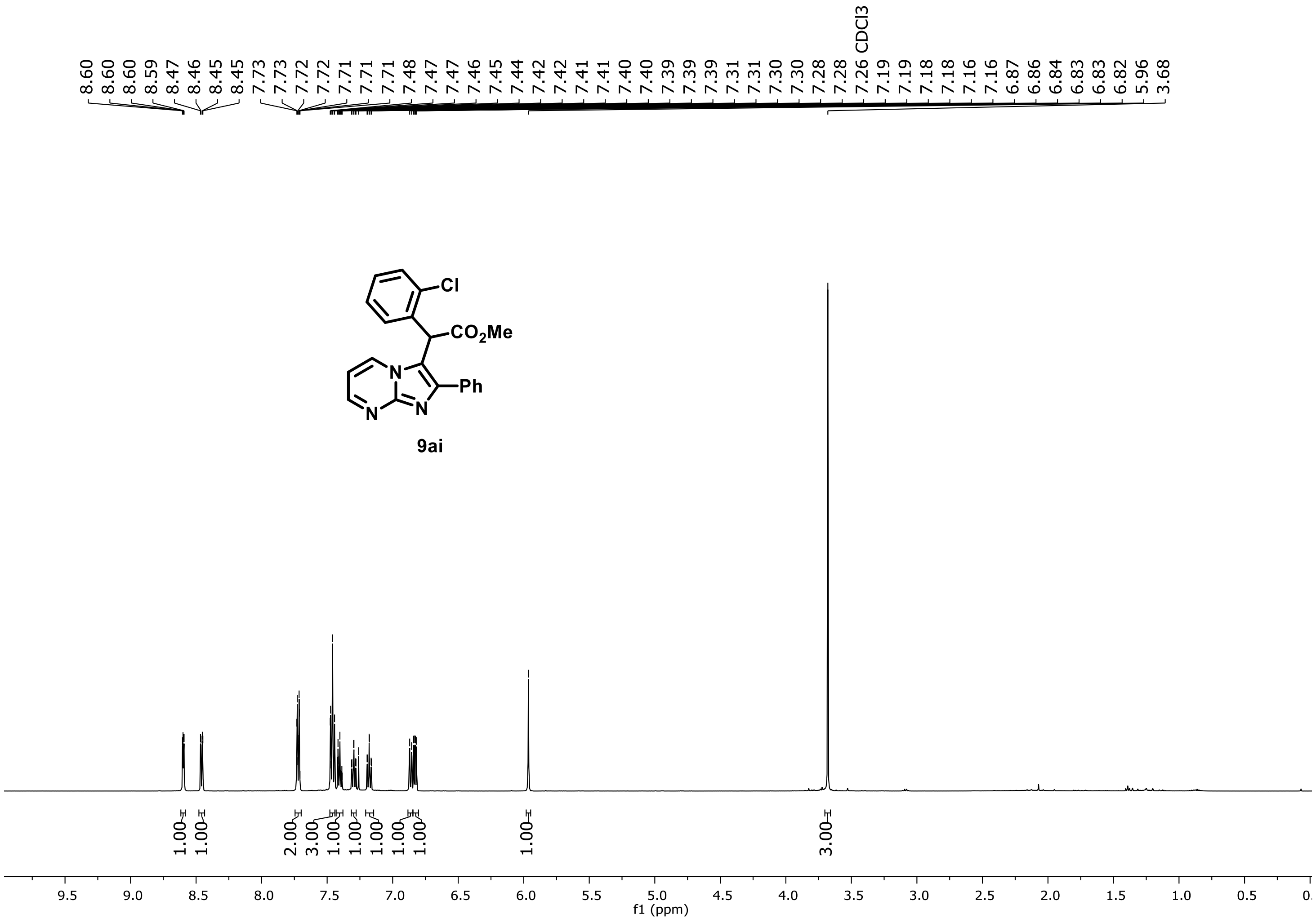

Molecule 9ai: $^{13}C\{^{1}H\}$ NMR (125 MHz, $CDCl_3$)

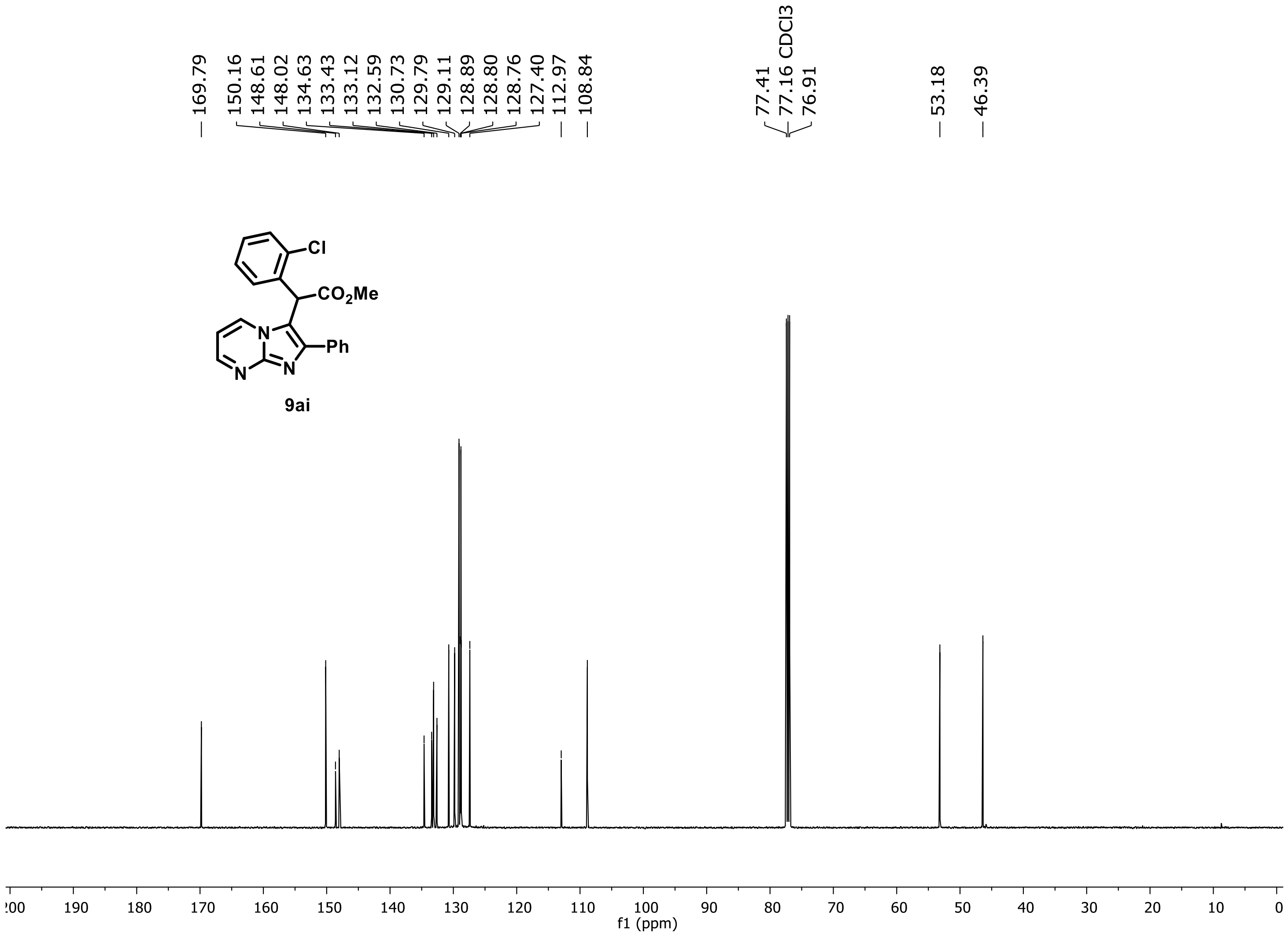

Molecule 9aj: ^{1}H NMR (300 MHz, $CDCl_3$)

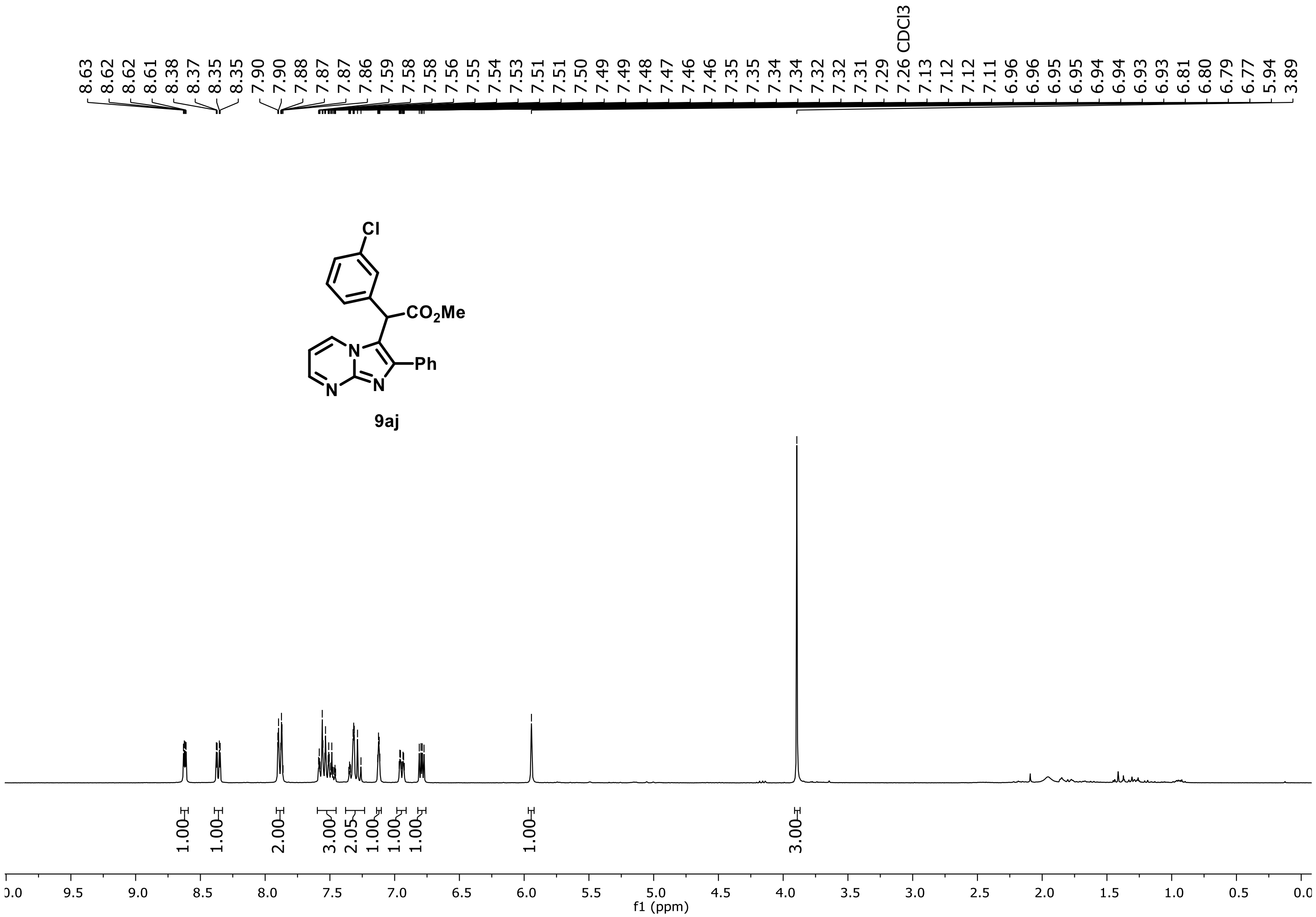

Molecule 9aj: $^{13}C\{^1H\}$ NMR (75 MHz, $CDCl_3$)

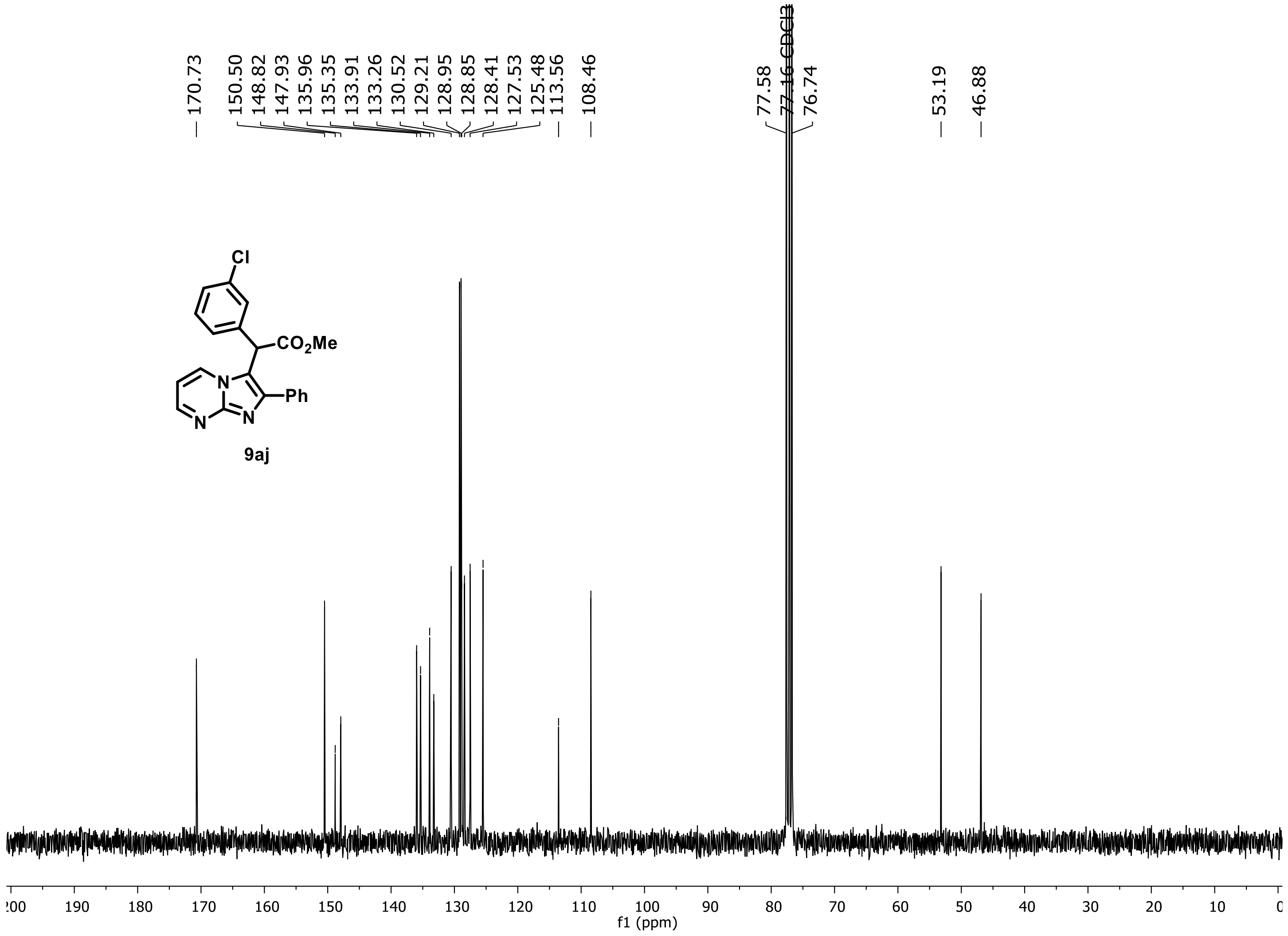

Molecule 9ak: ¹H NMR (300 MHz, $CDCl_3$)

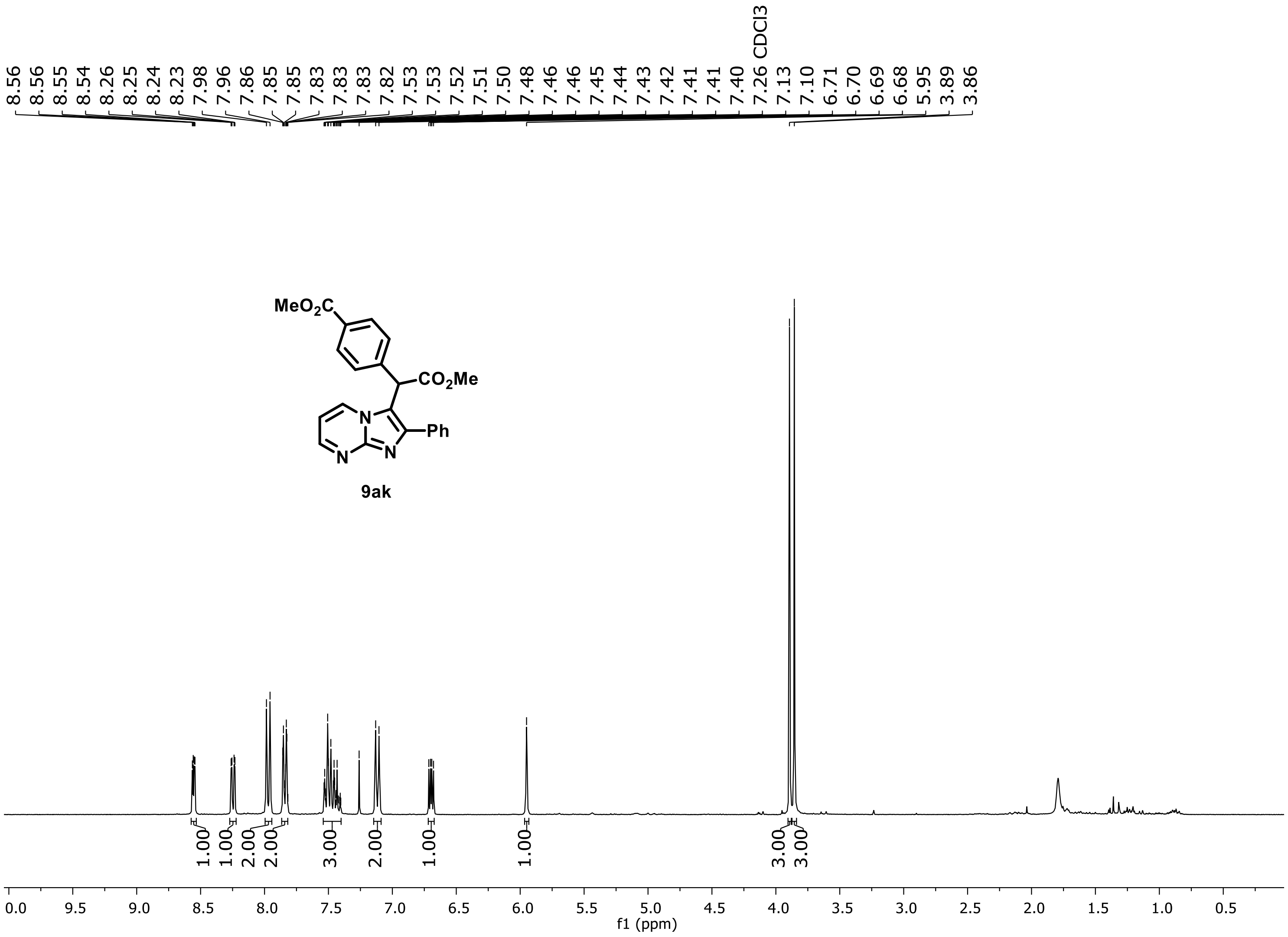

Molecule 9ak: $^{13}C\{^{1}H\}$ NMR (75 MHz, $CDCl_3$)

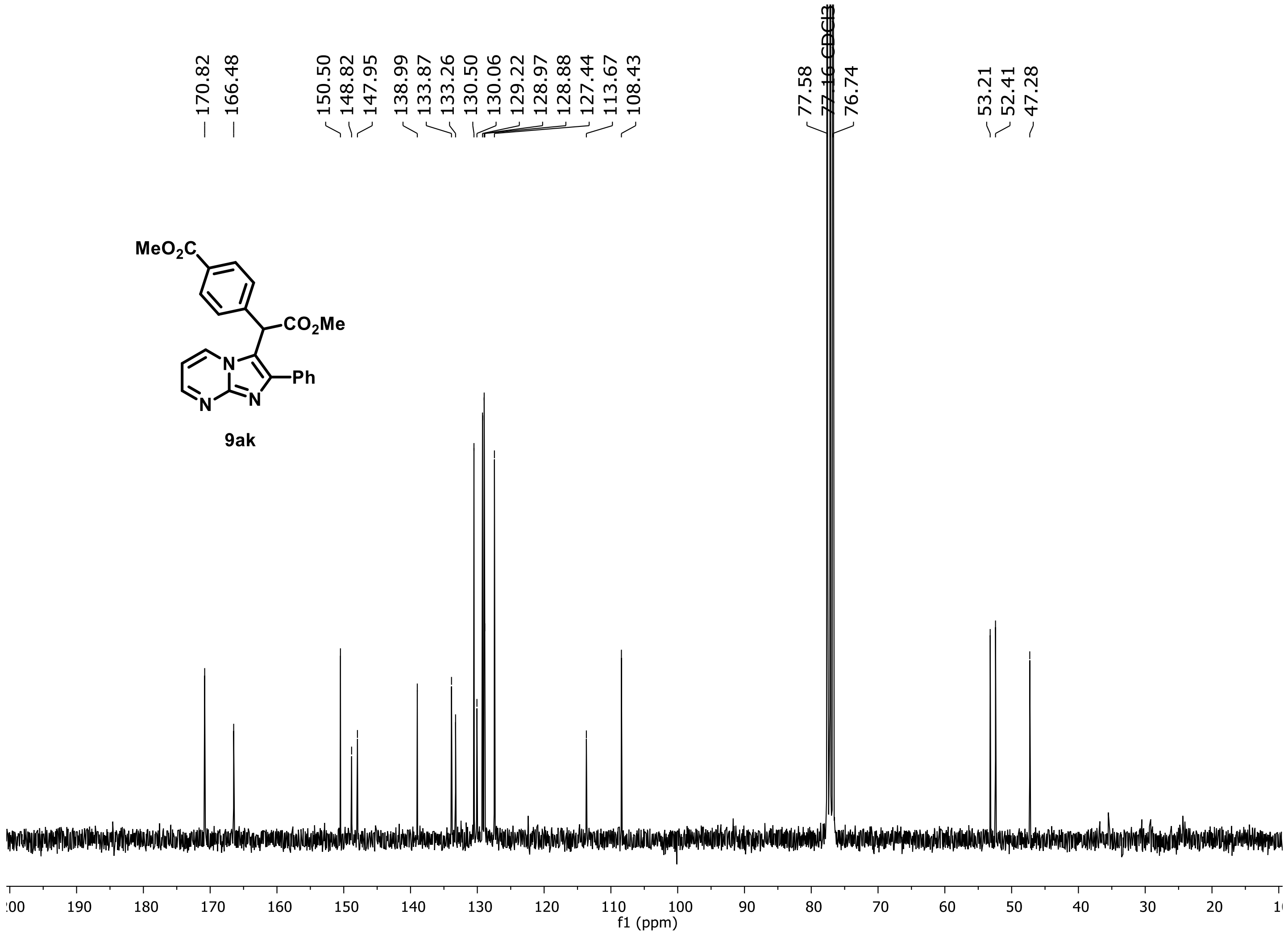

Molecule 9al: ^{1}H NMR (500 MHz, $CDCl_3$)

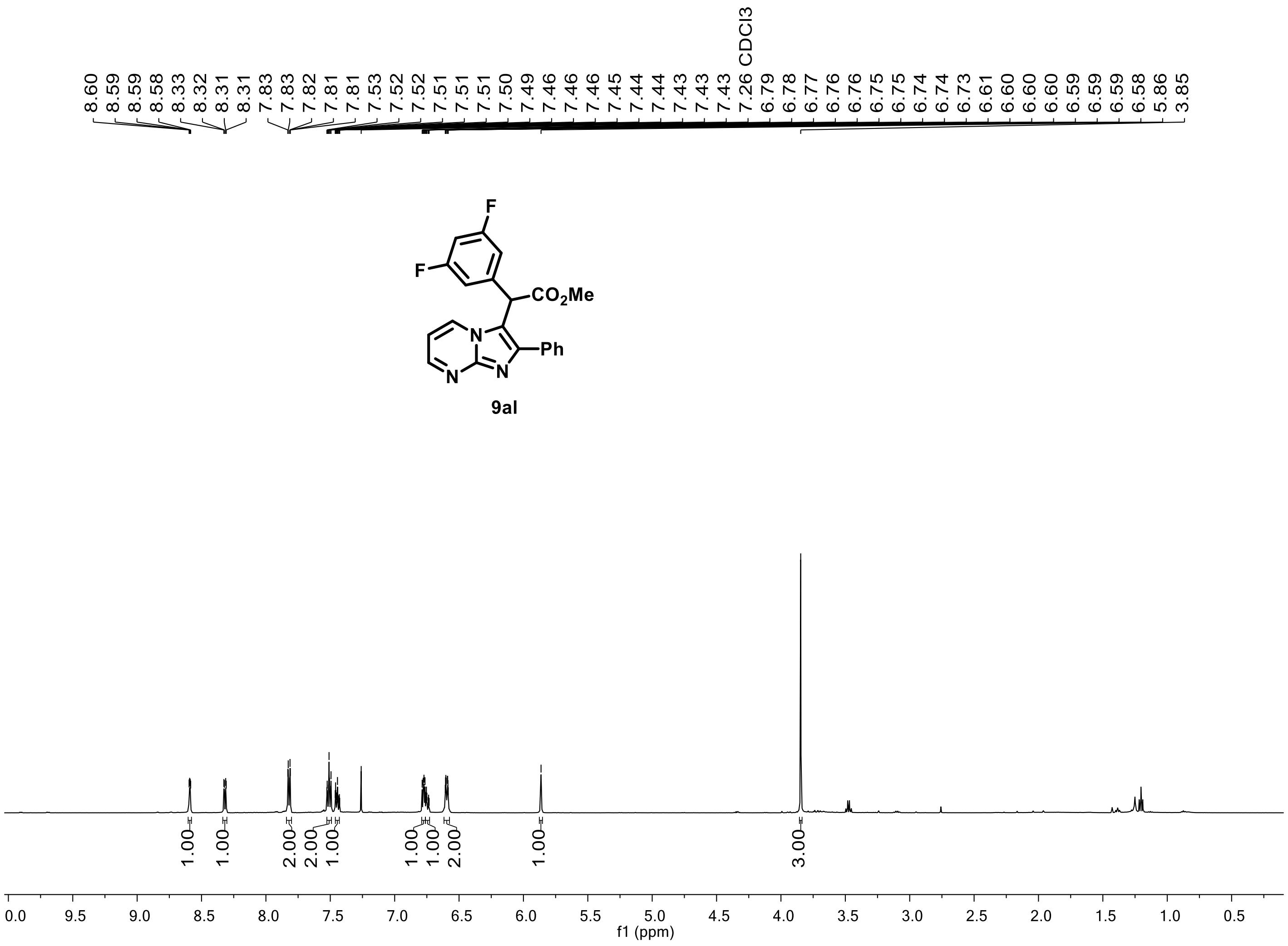

Molecule 9al: $^{13}C\{^1H\}$ NMR (125 MHz, $CDCl_3$)

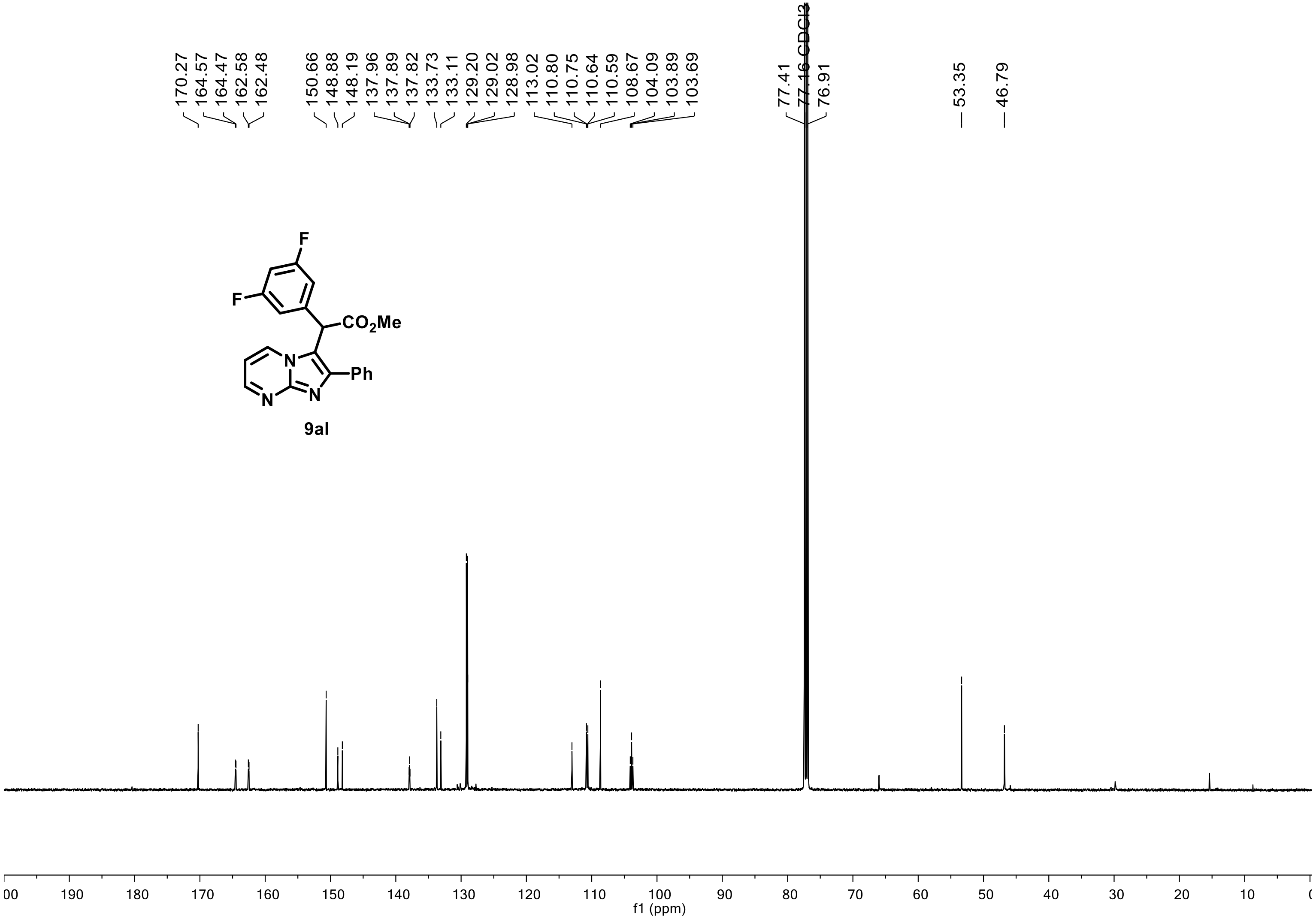

Molecule 9al: $^{19}F\{^{1}H\}$ NMR (470 MHz, CDCl₃)

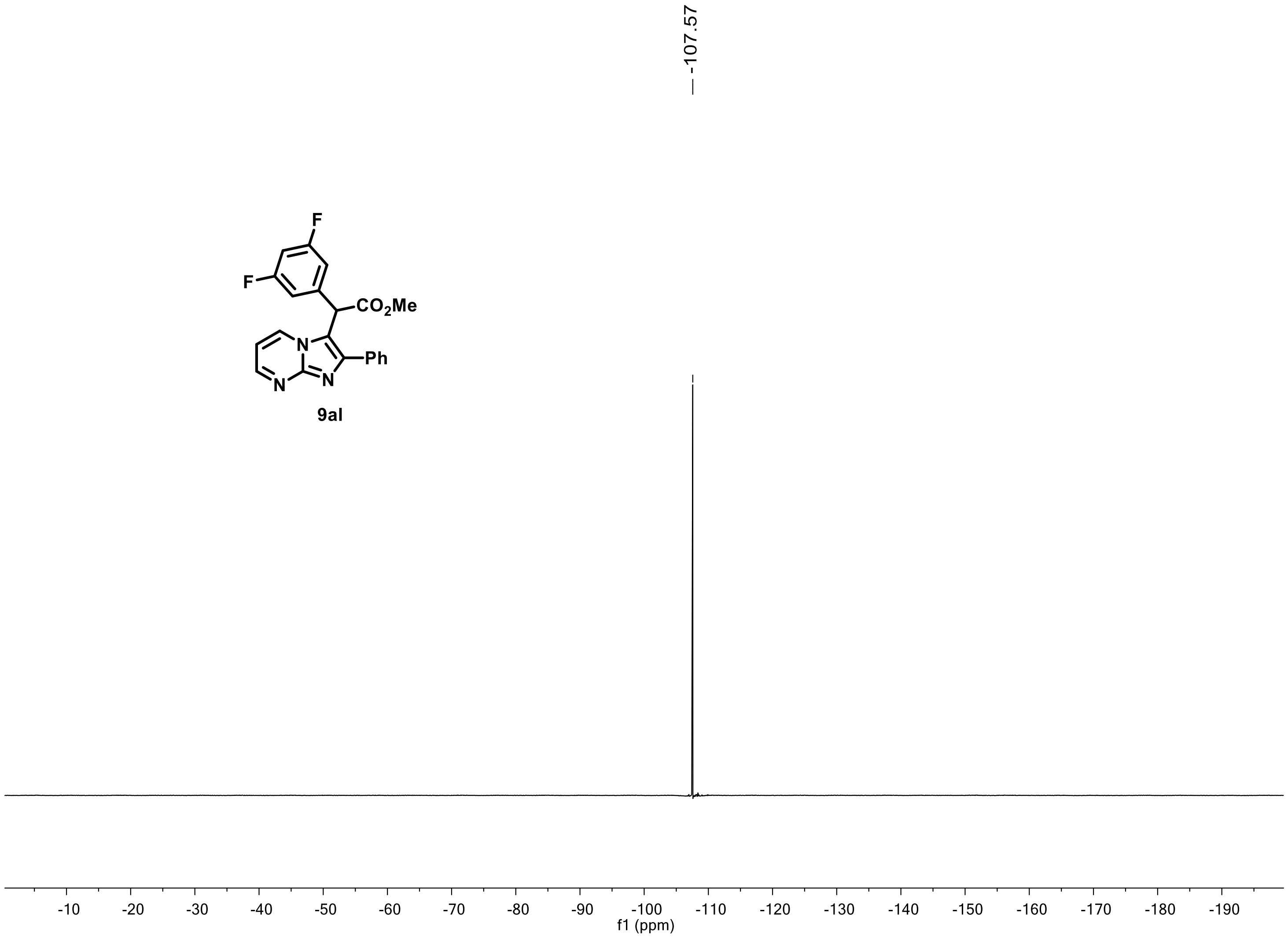

Molecule 9am: ^{1}H NMR (600 MHz, $CDCl_3$)

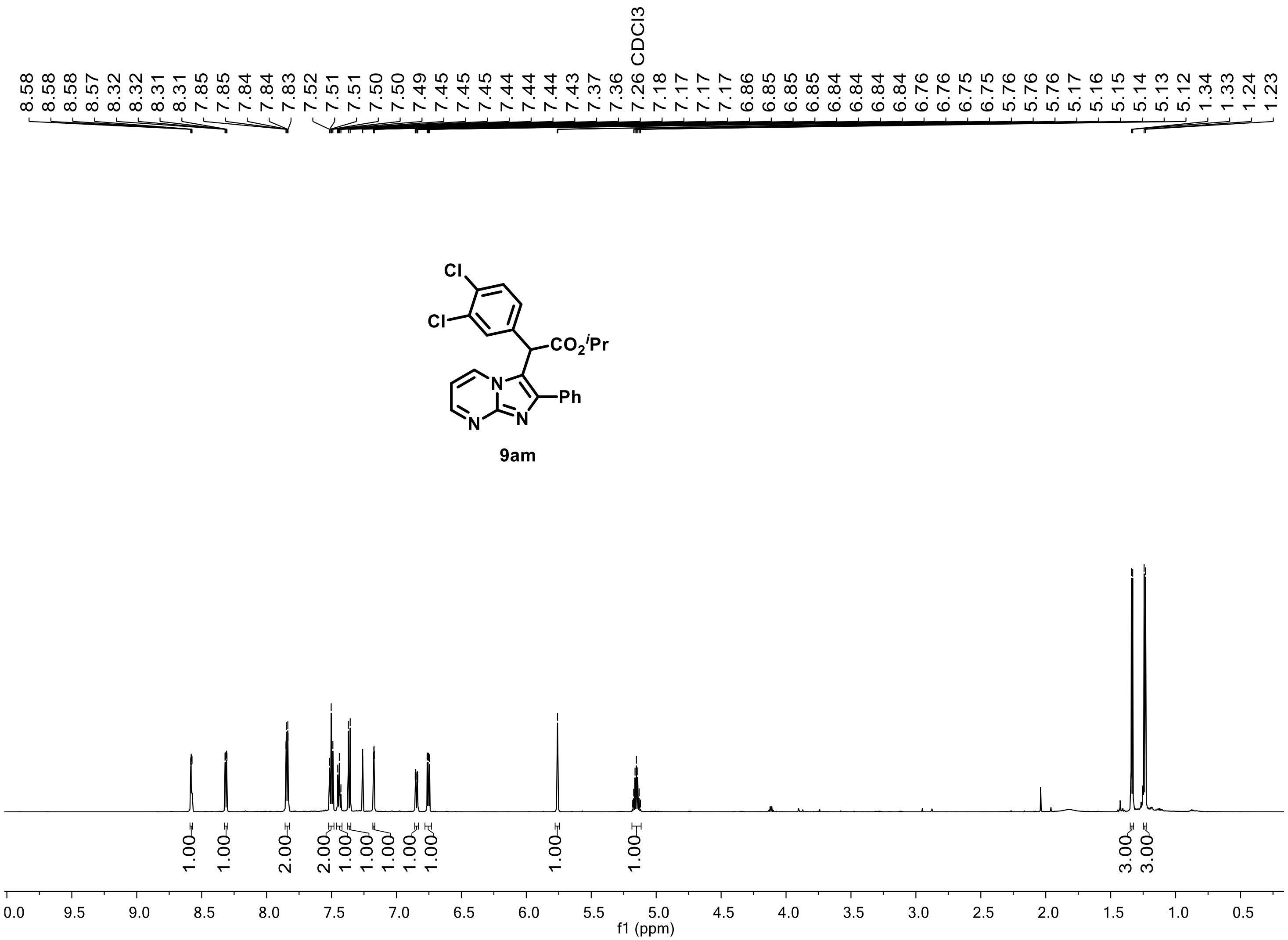

Molecule 9am: $^{13}C\{^{1}H\}$ NMR (150 MHz, $CDCl_3$)

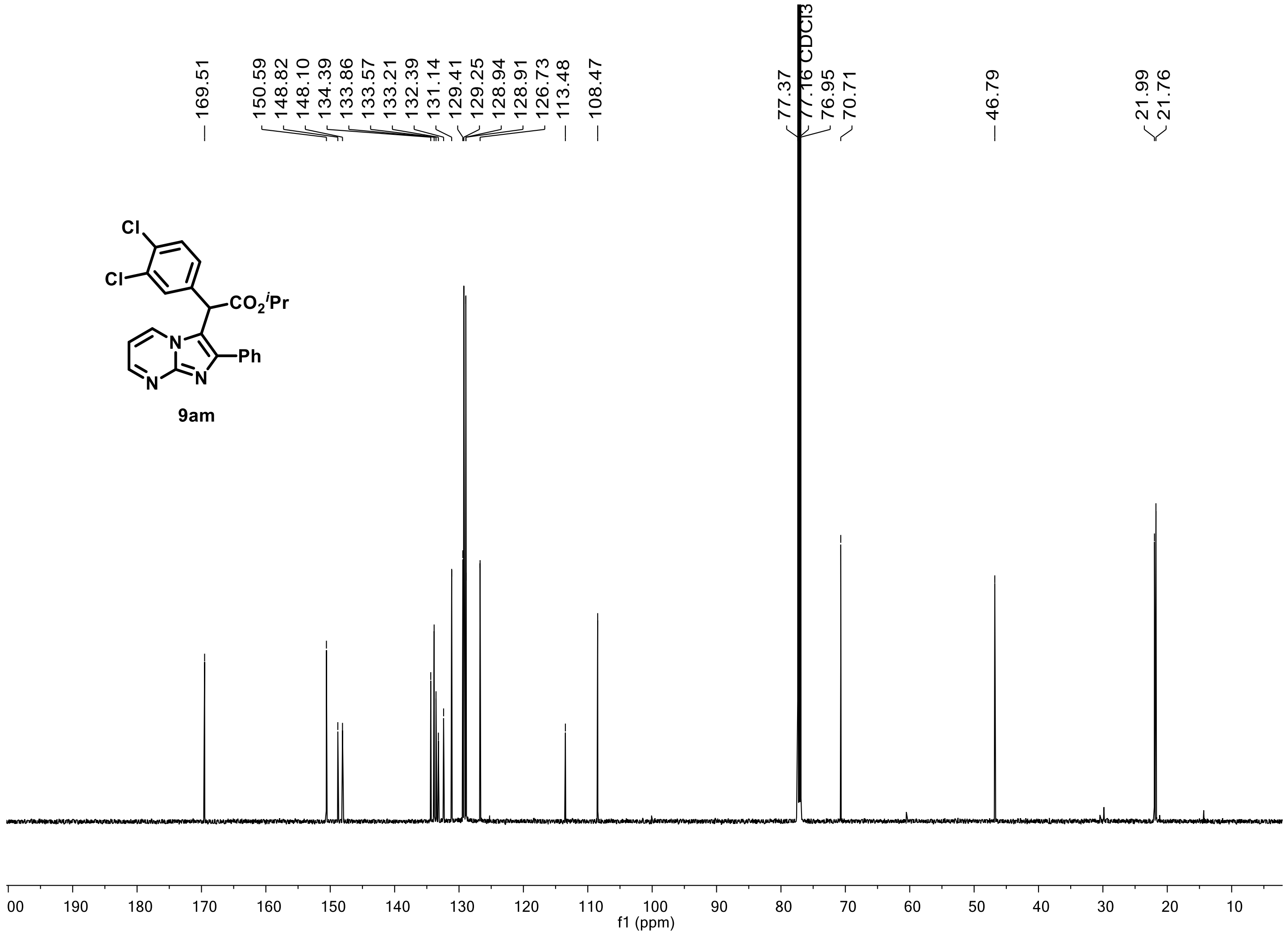

Molecule 9lm: ^{1}H NMR (400 MHz, $CDCl_3$)

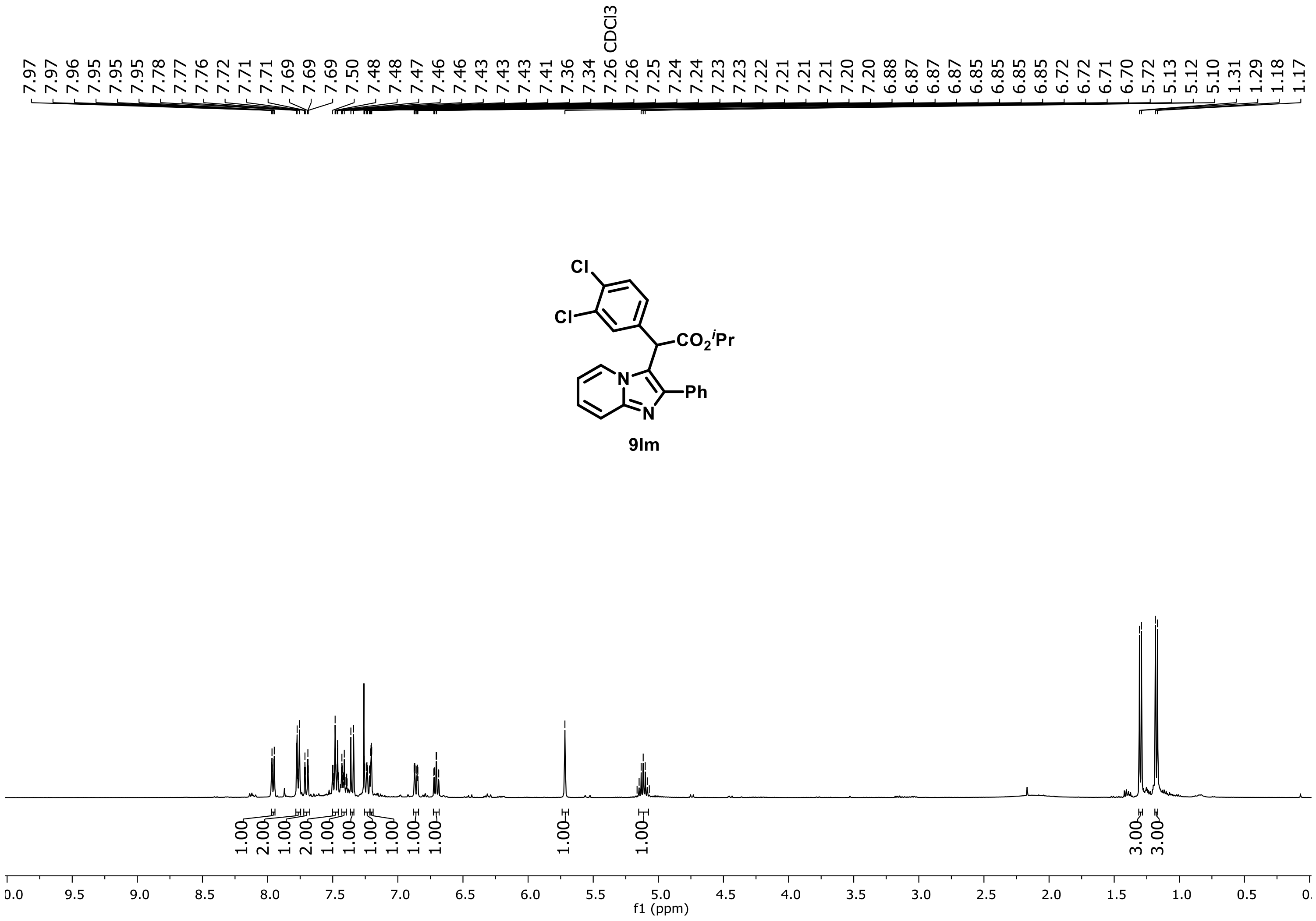

Molecule 9lm: $^{13}C\{^{1}H\}$ NMR (100 MHz, $CDCl_3$)

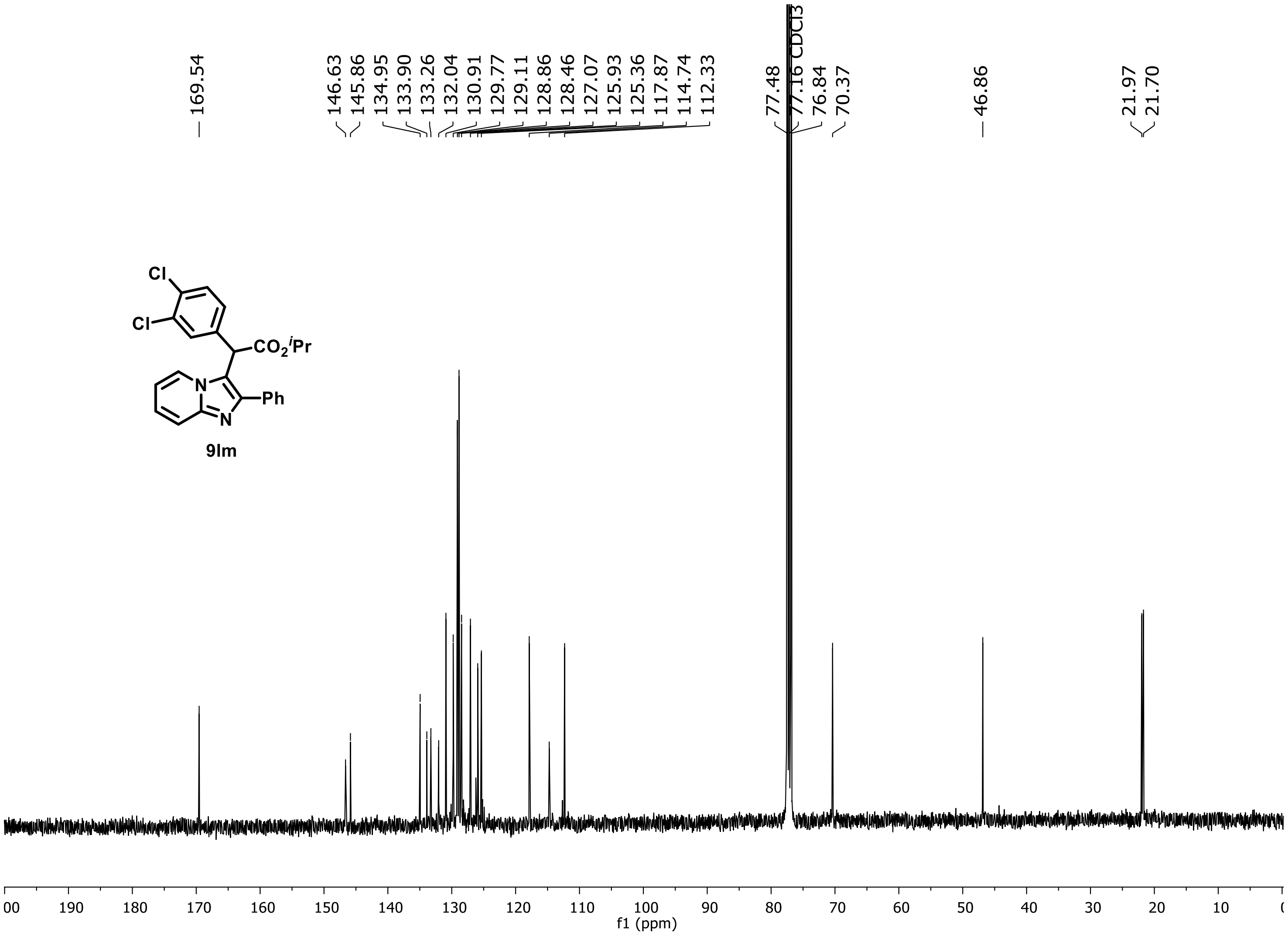

Molecule 9la: ^{1}H NMR (600 MHz, $CDCl_3$)

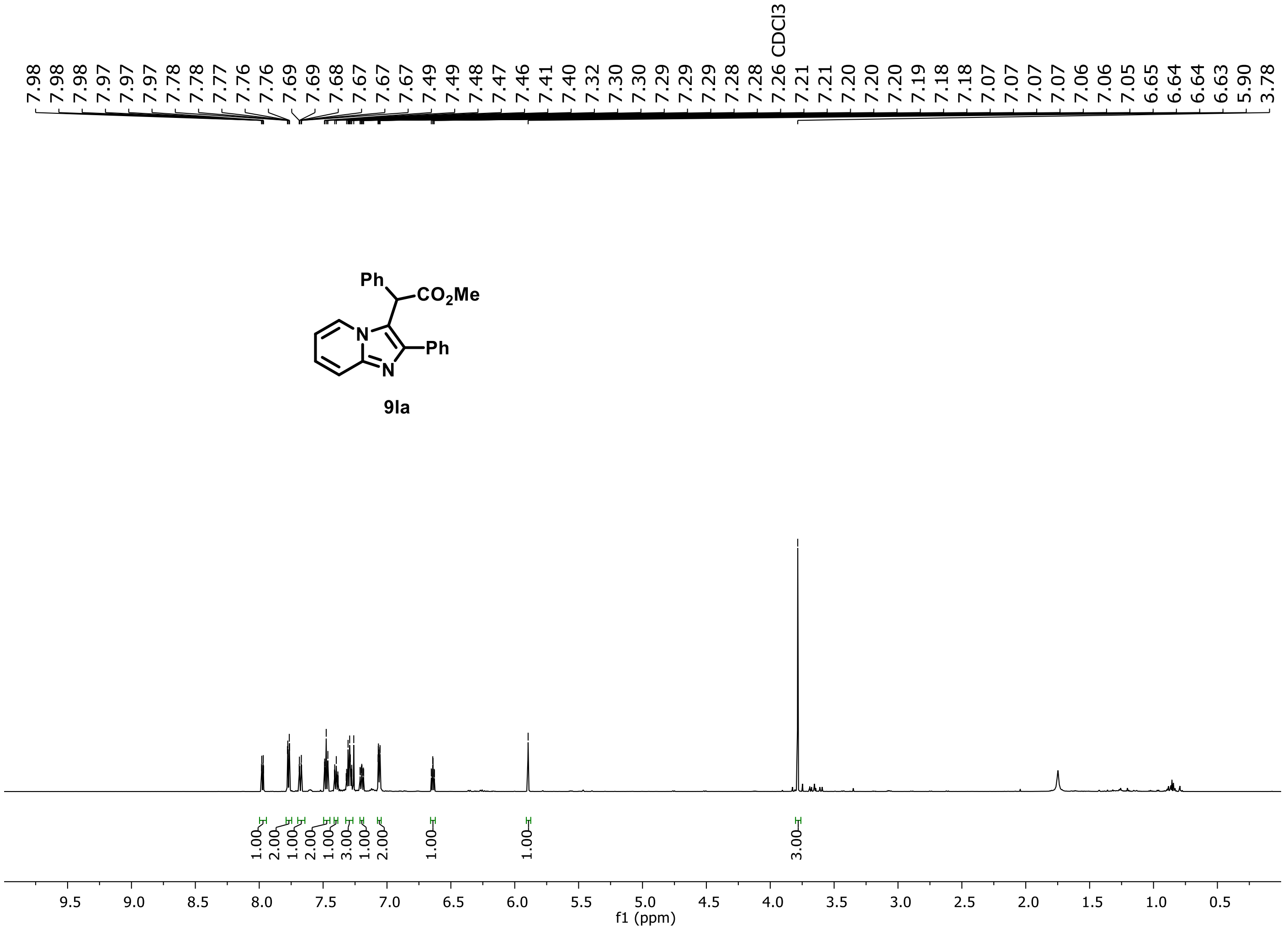

Molecule 9la: $^{13}C\{^{1}H\}$ NMR (150 MHz, $CDCl_3$)

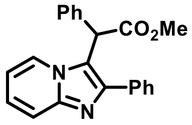

9la

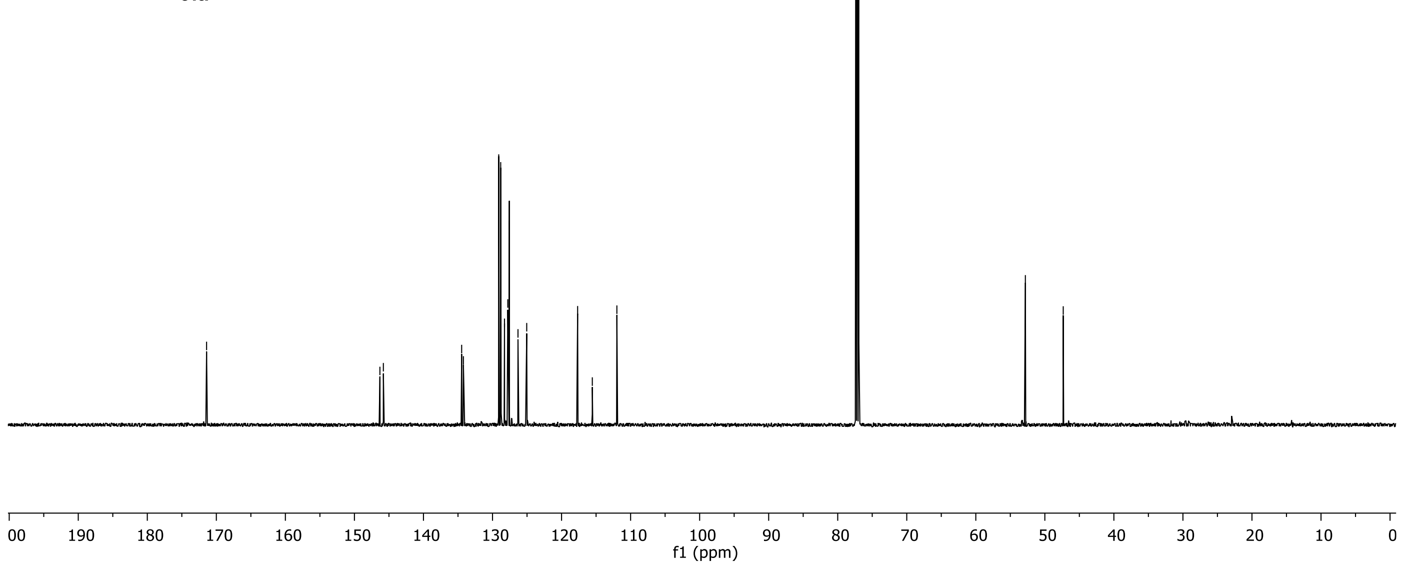

Molecule 9ma: ^{1}H NMR (600 MHz, $CDCl_3$)

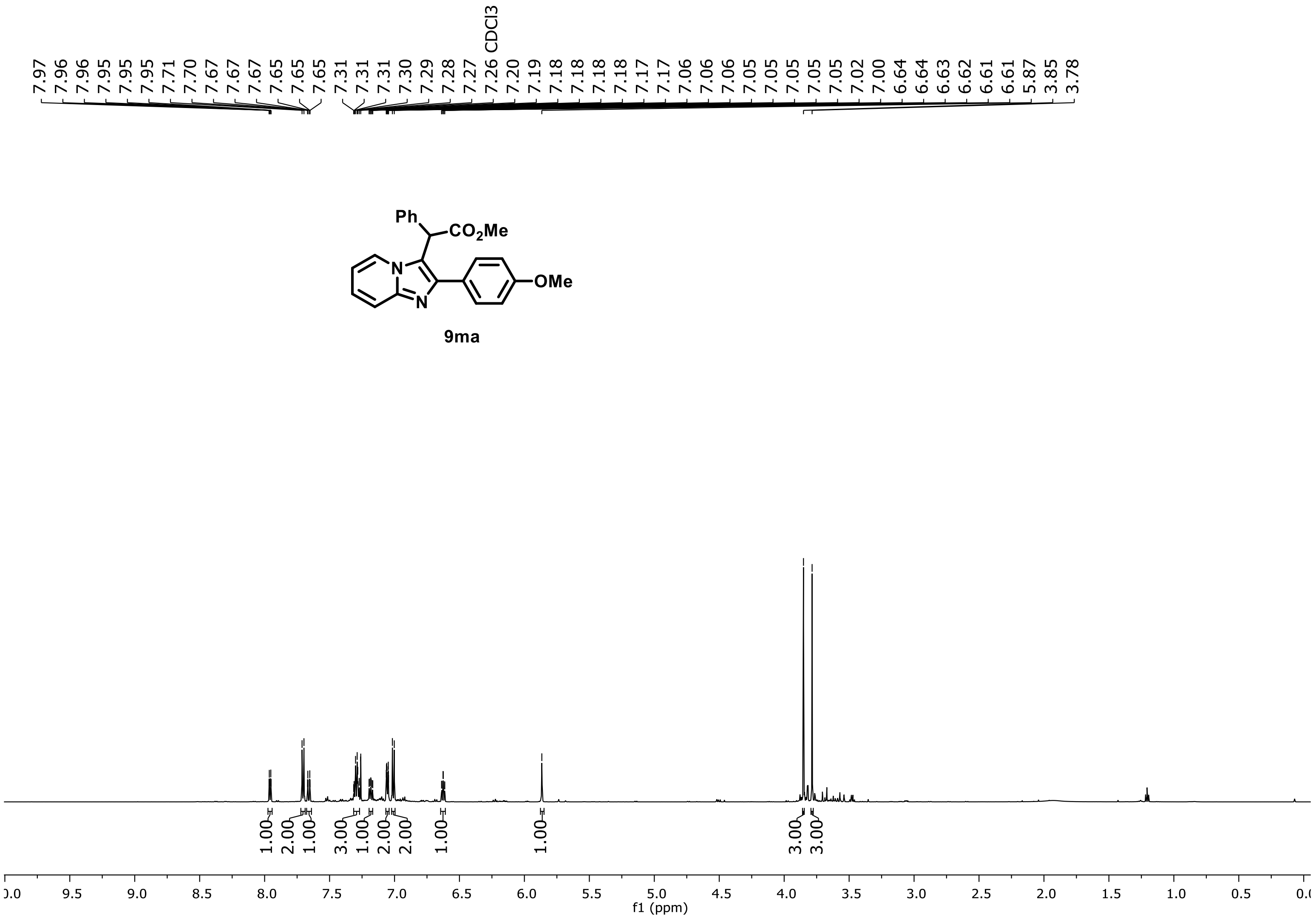

Molecule 9ma: $^{13}C\{^1H\}$ NMR (150 MHz, $CDCl_3$)

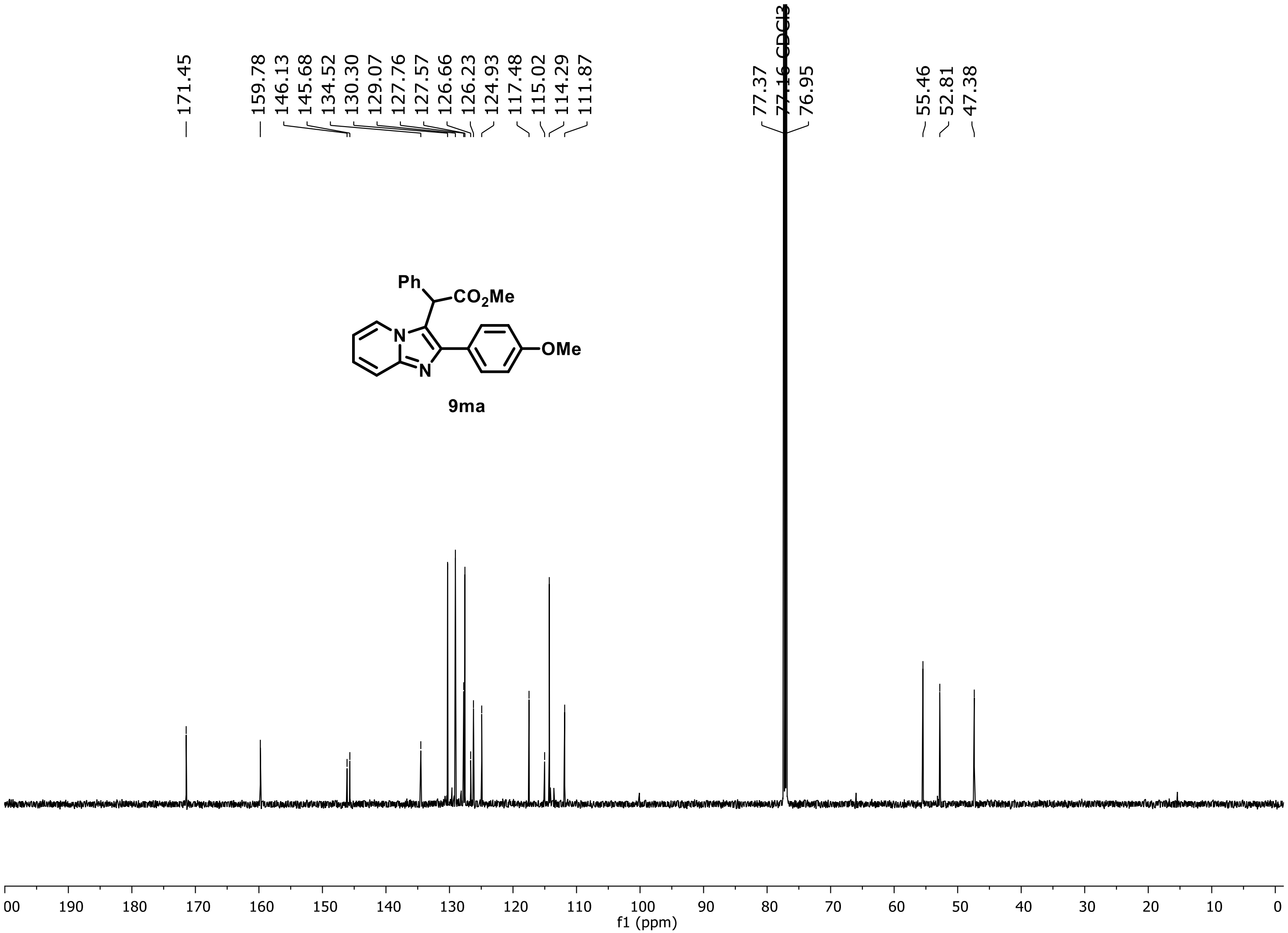

Molecule 9na: ^{1}H NMR (600 MHz, $CDCl_3$)

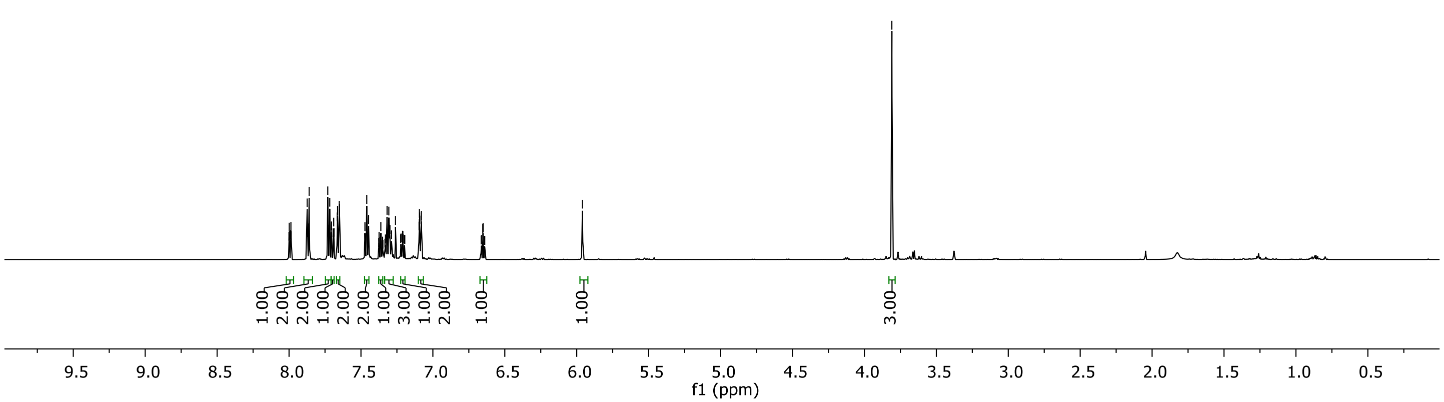

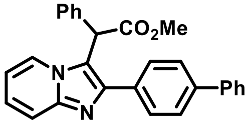

9na

Molecule 9na: $^{13}C\{^1H\}$ NMR (150 MHz, $CDCl_3$)

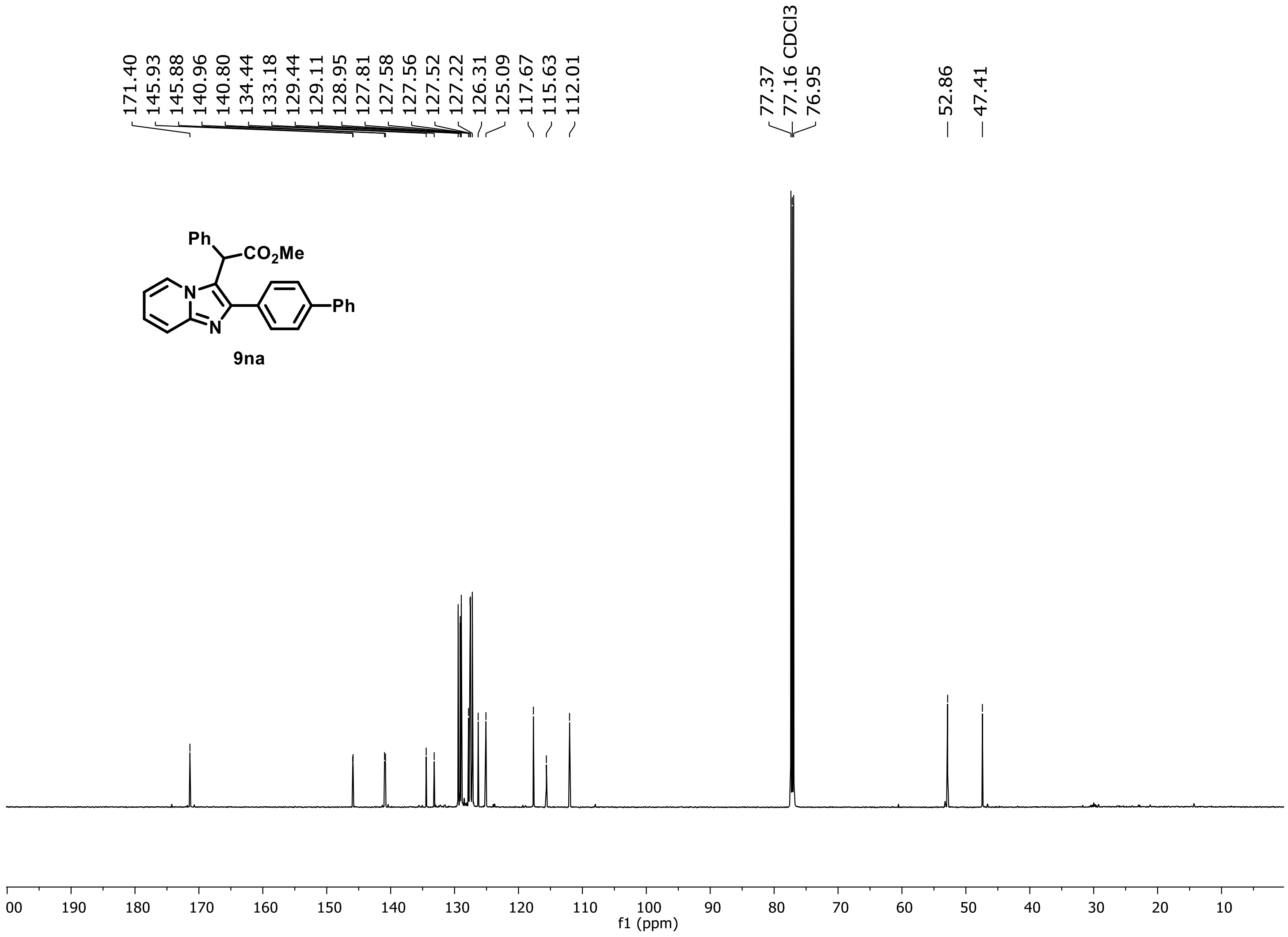

Molecule 9lf: ^{1}H NMR (500 MHz, $CDCl_3$)

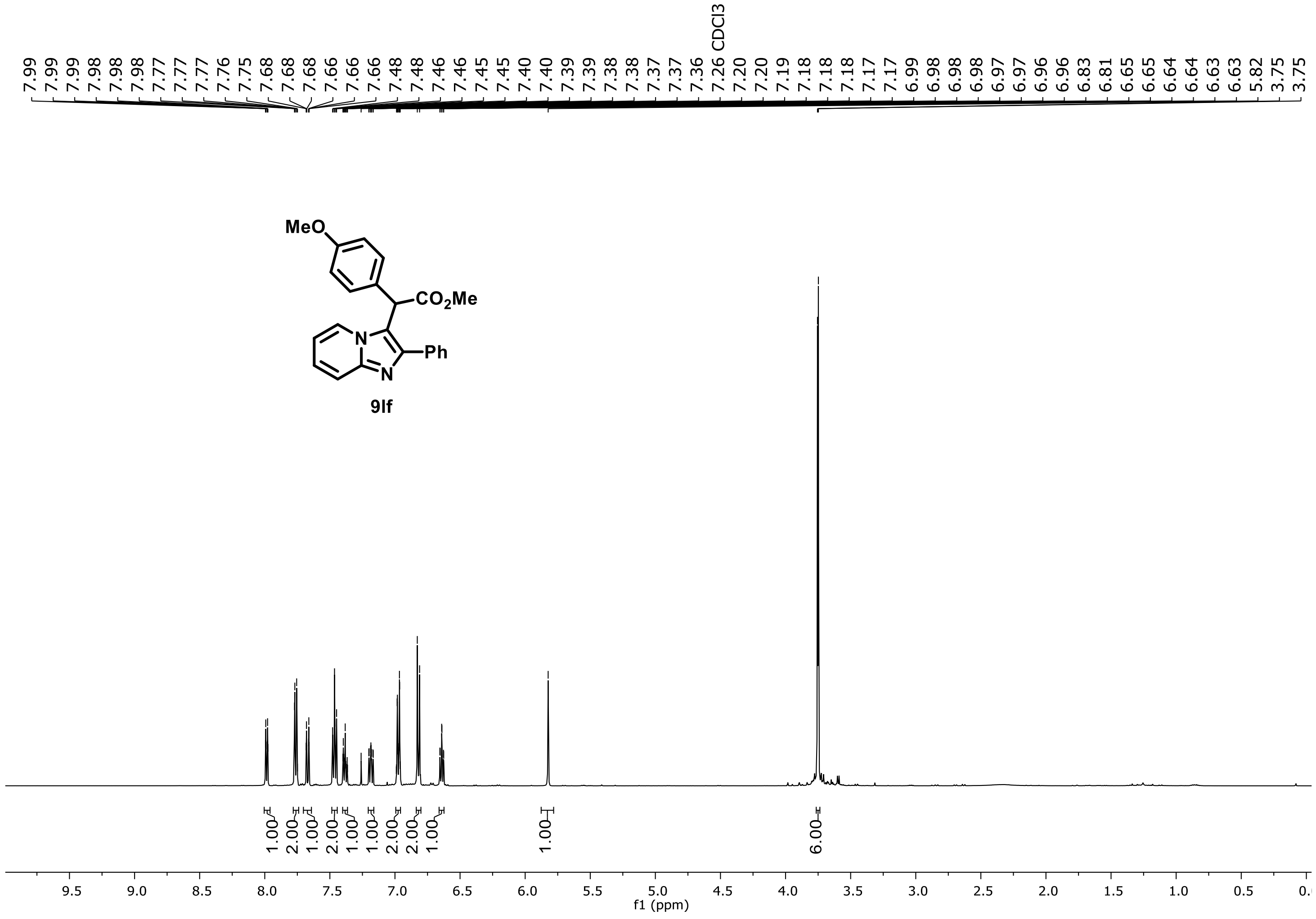

Molecule 9lf: $^{13}C\{^{1}H\}$ NMR (125 MHz, $CDCl_3$)

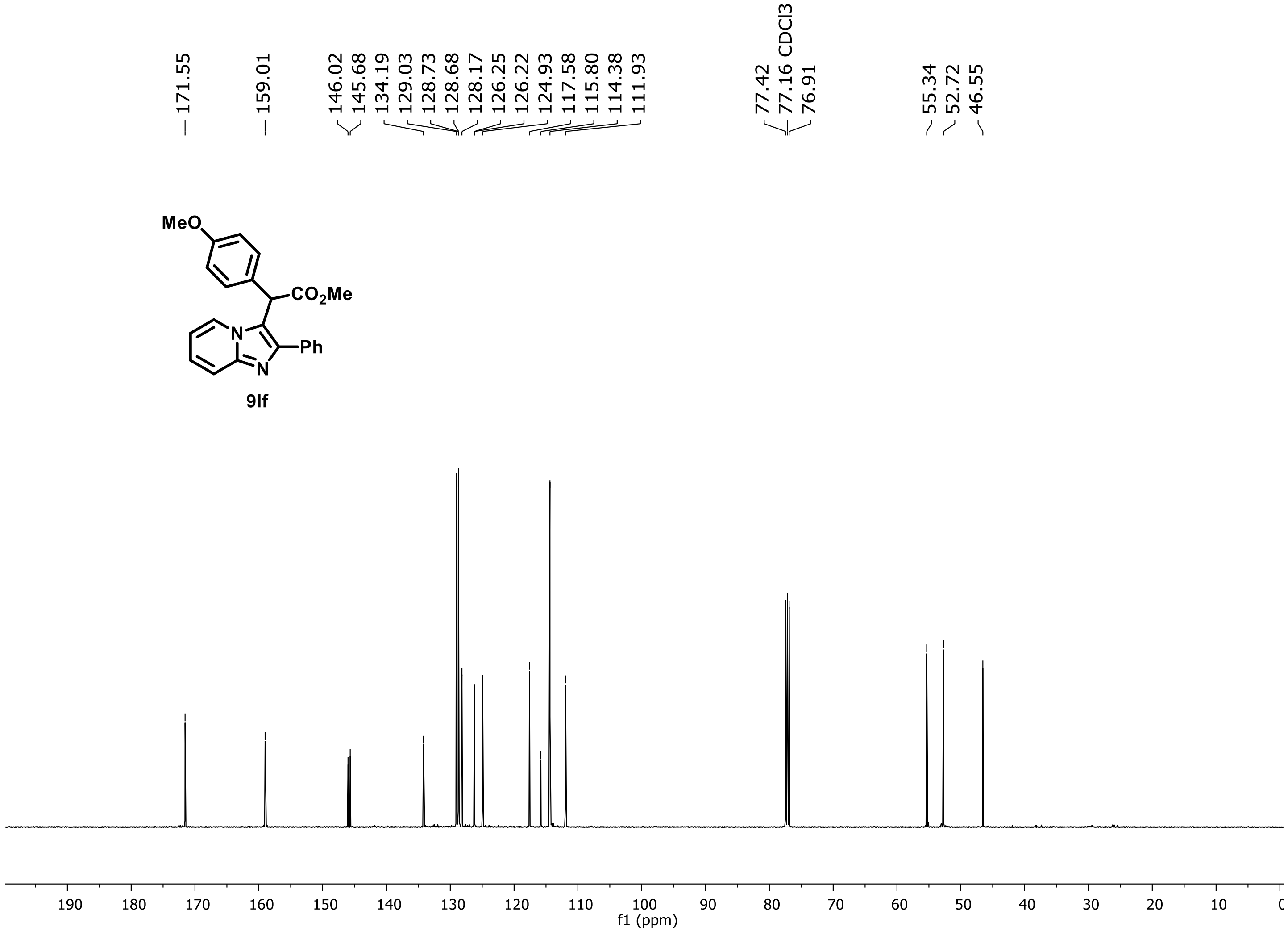

Molecule 9lk: ^{1}H NMR (500 MHz, $CDCl_3$)

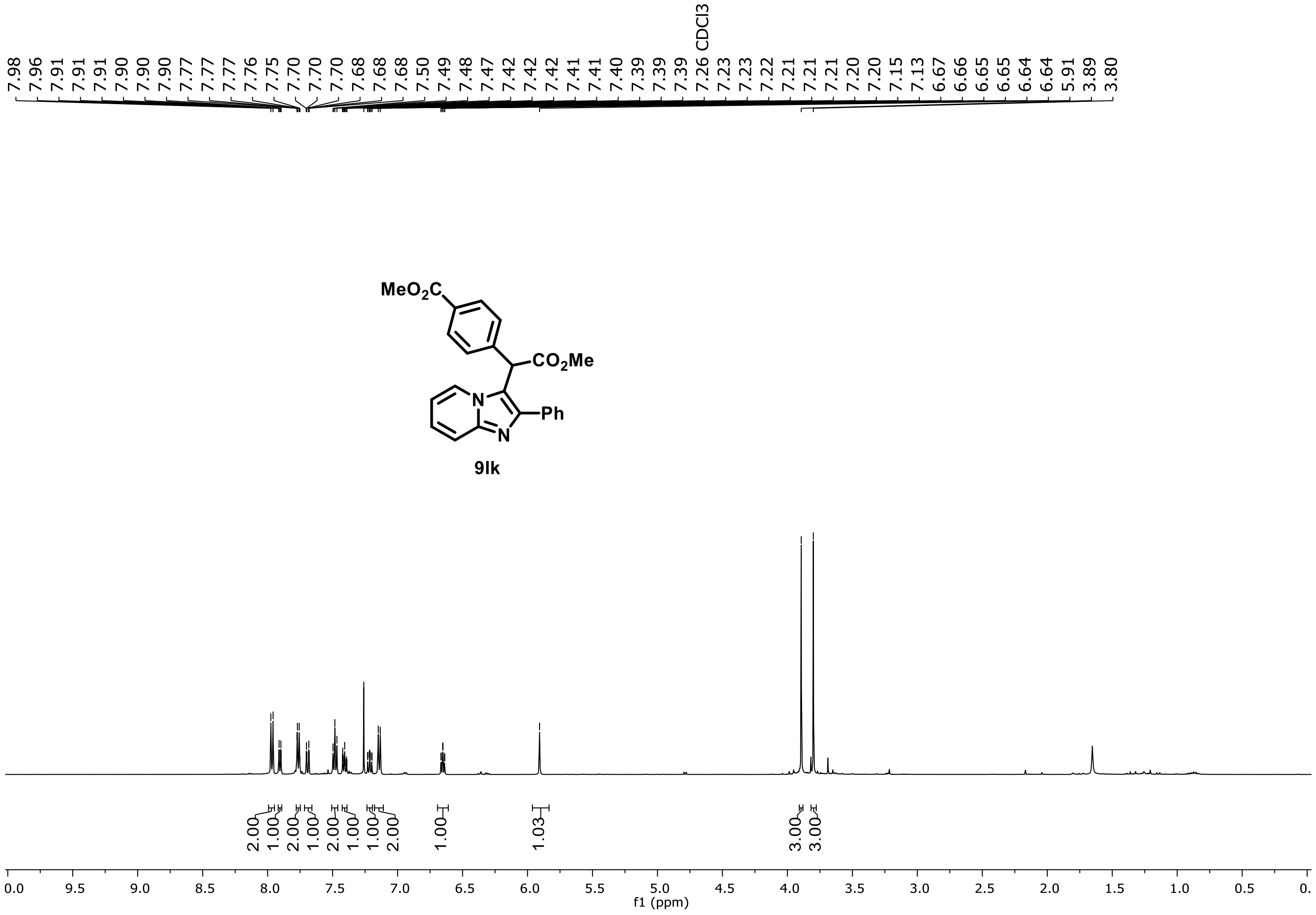

Molecule 9lk: $^{13}C\{^{1}H\}$ NMR (125 MHz, $CDCl_3$)

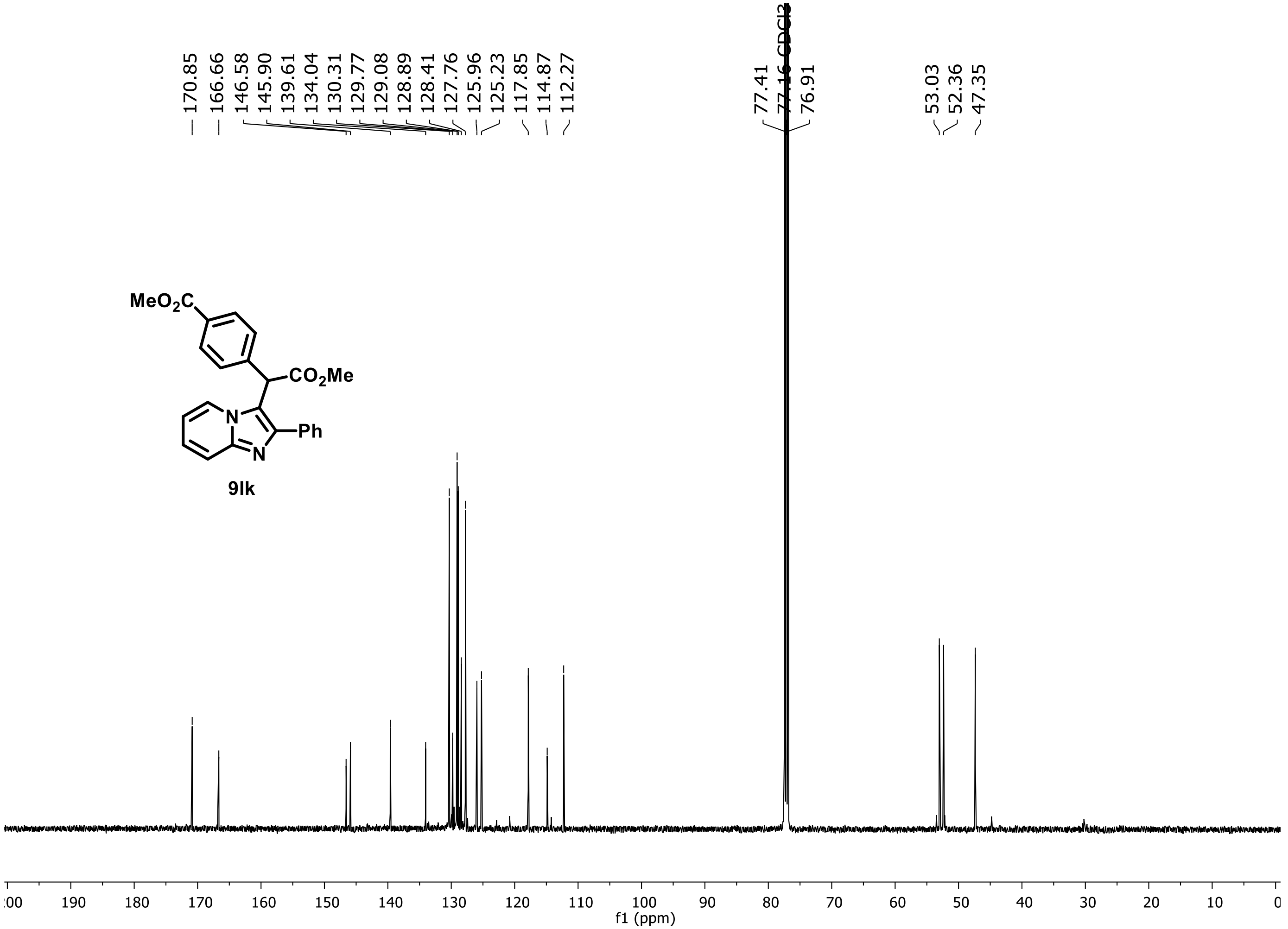

9lk

Crude reaction mixture of reaction 7p + 12 → 13 with 1,3,5-TMB as IS: ^{1}H NMR (300 MHz, $CDCl_3$)

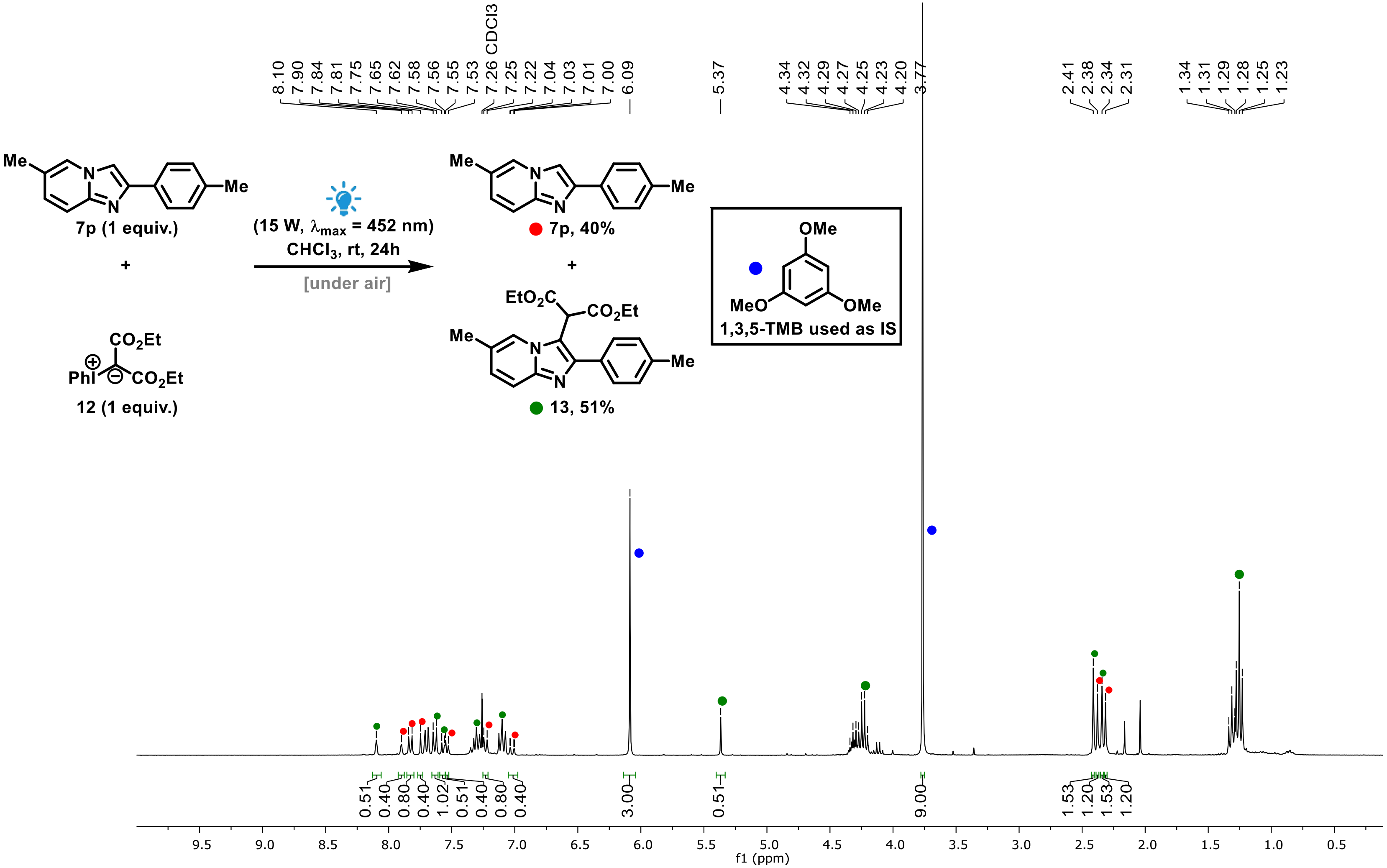

Molecule 14: ^{1}H NMR (500 MHz, $CDCl_3$)

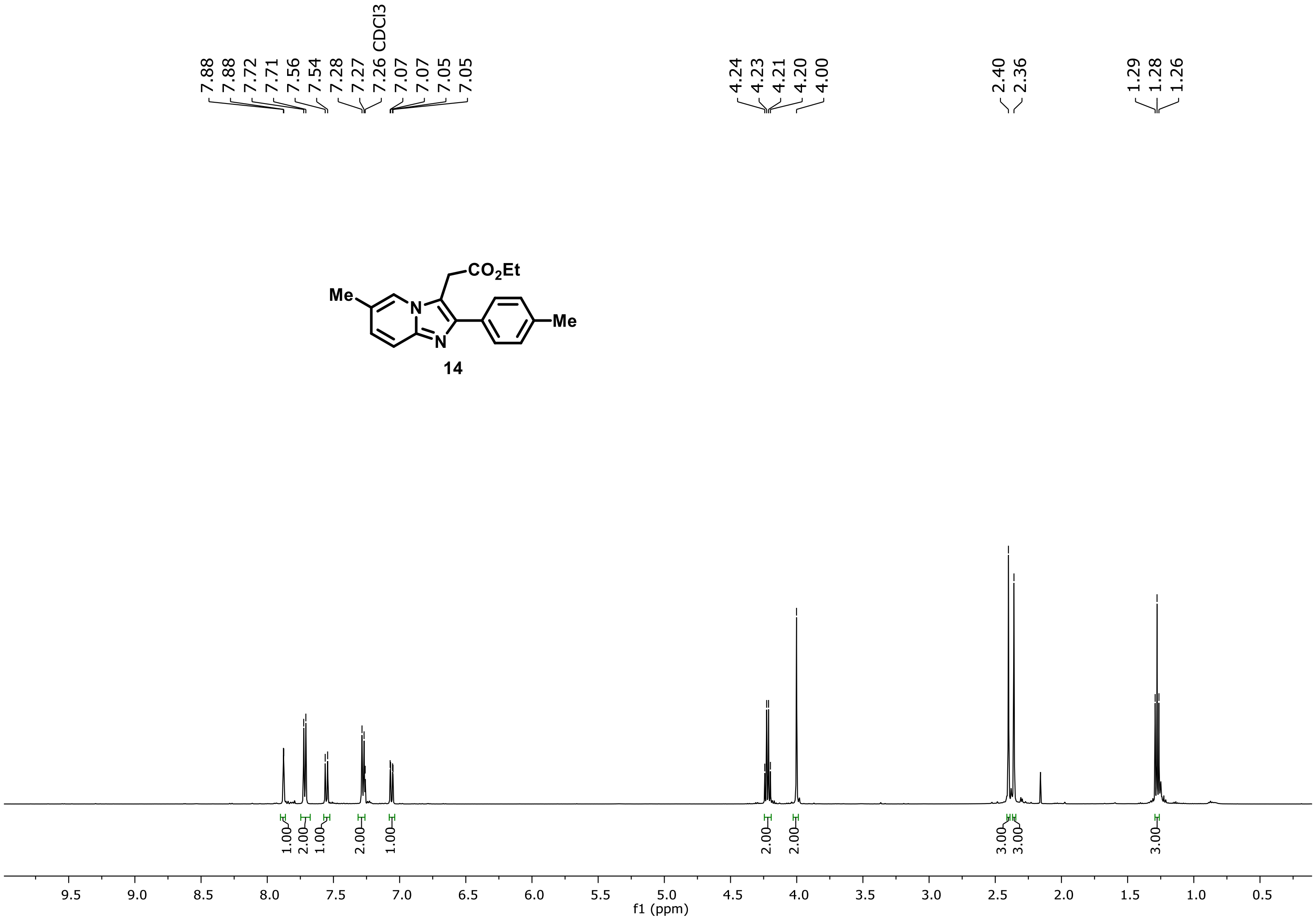

Molecule 14: $^{13}C\{^{1}H\}$ NMR (125 MHz, $CDCl_3$)

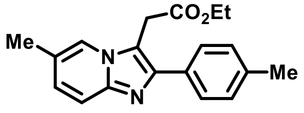

14

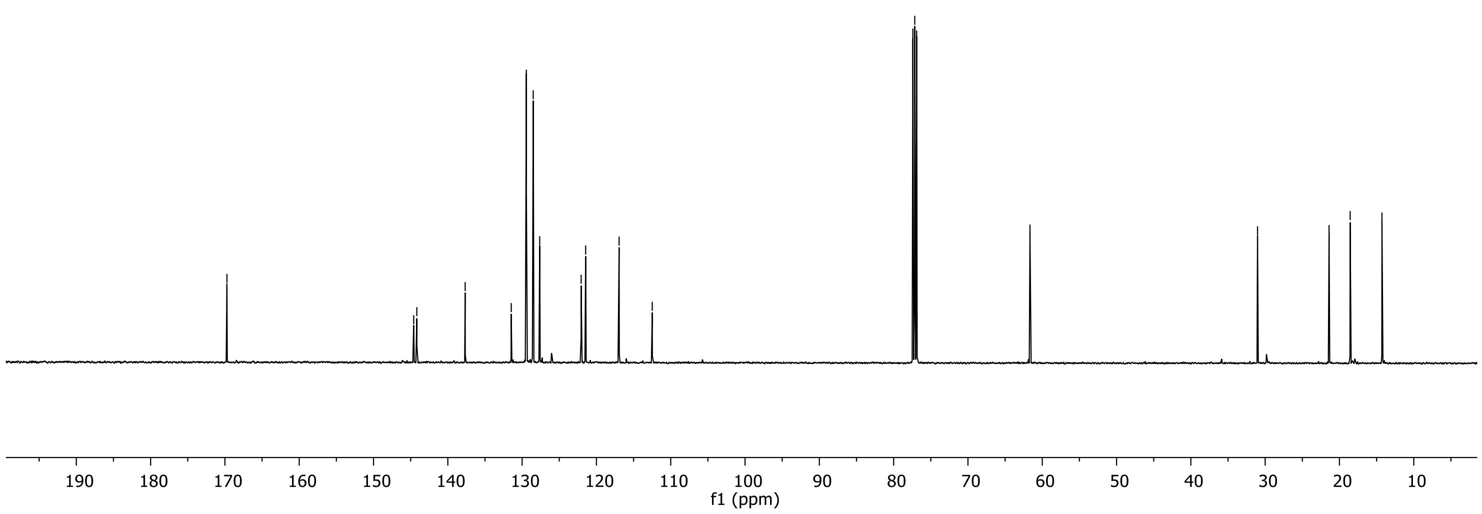

Supplement: Supplementary file 1 — Supporting Information [file ASIA-20-e00455-s001.pdf]
